# Cyaphide generation at an aluminium(i) center: a useful precursor for phosphorus-containing heterocycles

**DOI:** 10.1039/d6sc03023h

**Published:** 2026-05-05

**Authors:** Artyom Yakubenko, Álvaro García-Romero, Stephanie J. Urwin, Israel Fernández, Jose M. Goicoechea

**Affiliations:** a Department of Chemistry, Indiana University 800 East Kirkwood Avenue Bloomington Indiana 47405 USA jgoicoec@iu.edu; b EaStCHEM School of Chemistry, University of Edinburgh David Brewster Road Edinburgh EH9 3FJ UK; c Departamento de Química Orgánica and Centro de Innovación en Química Avanzada (ORFEO-CINQA), Universidad Complutense de Madrid, Facultad de Ciencias Químicas Madrid 28040 Spain israel@quim.ucm.es

## Abstract

The synthesis of an aluminium(iii) cyaphido complex, accessed through the formal oxidative addition of PCOSi^*i*^Pr_3_ at an aluminium(i) metal center, is reported. Reaction of Al(^Dipp^NacNac) (^Dipp^NacNac = CH{C(CH_3_)N(Dipp)}_2_; Dipp = 2,6-di(*iso*-propyl)phenyl) with PCOSi^*i*^Pr_3_ affords the aluminium(iii) complex Al(^Dipp^NacNac)(OSi^*i*^Pr_3_)(CP) in moderate (42%) isolated yield. Formation of this compound is accompanied by the concomitant formation of two isomeric side-products: a four-membered metallacycle Al(^Dipp^NacNac)[*κ*^2^-P(O)CSi^*i*^Pr_3_], and the phospha-aluminirene Al(^Dipp^NacNac)(*η*^2^-PCOSi^*i*^Pr_3_). The reactivity of Al(^Dipp^NacNac)(OSi^*i*^Pr_3_)(CP) is governed by the enhanced covalency of the Al–CP bond relative to magnesium(ii) cyaphido complexes, and the steric protection offered to the cyaphide moiety by the β-diketiminato and siloxide ligands. Despite the diminished reactivity of this compound when compared to more ionic complexes of the cyaphide ion, the C≡P triple bond partakes in [2 + 1], [2 + 3] and [2 + 4] cyclization reactions with Ni(COD)_2_, organic azides, and 2,3-dimethyl-butadiene, respectively. These reactions can be used to access phosphorus-containing heterocycles that can be readily detached from the aluminium(iii) platform using iodation and transmetallation strategies.

## Abstract 2

The synthesis of an aluminium(iii) cyaphido complex, accessed through the formal oxidative addition of PCOSi^*i*^Pr_3_ at an aluminium(i) metal center, is reported.

## Graphical abstract

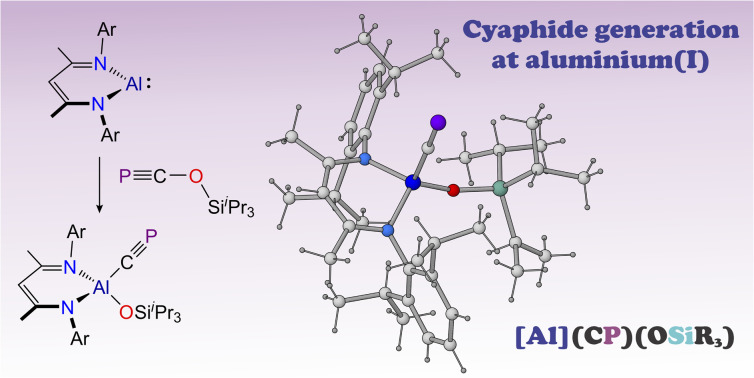

## Introduction

**Fig. 1:**
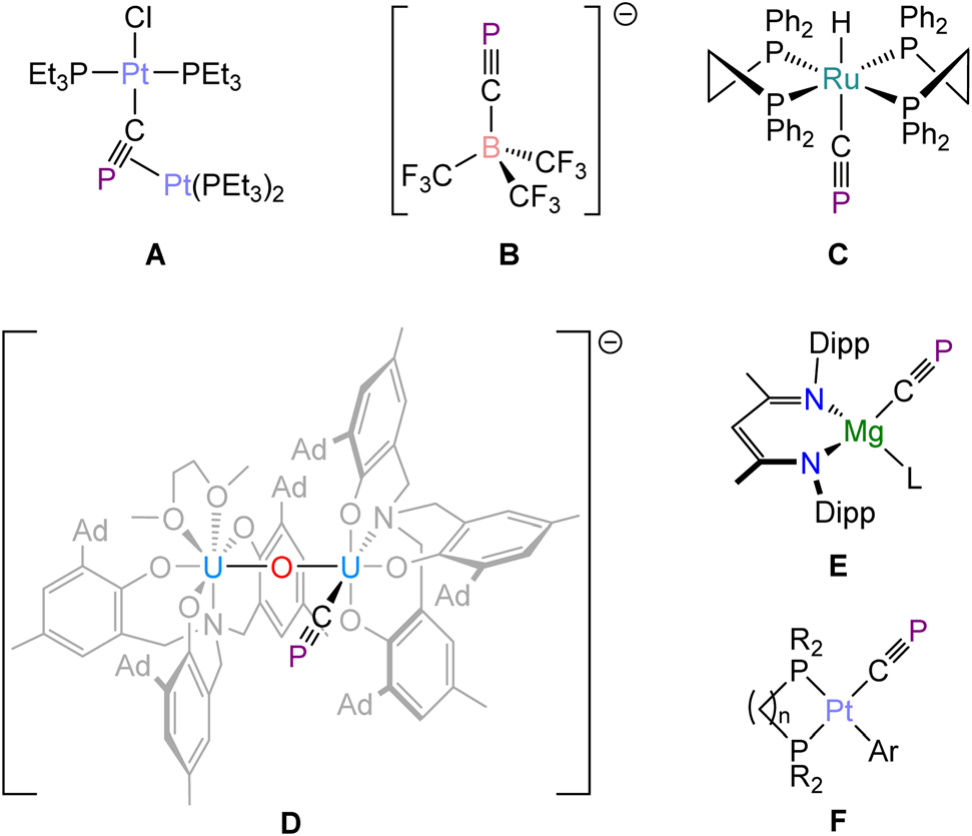
Selected complexes of the cyaphide ligand. L = dioxane, THF, I^*i*^Pr, IMes; R = ^*i*^Pr, Cy; Ar = Mes, Trip.

The cyaphide ion (C≡P^−^) is an underexplored phosphorus-containing analogue of cyanide (C≡N^−^).^1^ Until recently, metal cyaphido complexes were extremely rare due to the lack of a unified method for their synthesis. The first of these compounds, the bimetallic complex Pt(PEt_3_)_2_Cl(µ–C≡P)Pt(PEt_3_)_2_ (Fig. 1; A), was reported by Angelici and co-workers in 1992.^2,3^ This complex was synthesized using a metallophospha-alkene precursor, *trans*-Pt(PEt_3_)_2_Cl[C(Cl)=PMes], and is believed to involve the formation of a terminal cyaphide complex, *trans*-Pt(PEt_3_)_2_Cl(C≡P), as an intermediate. In 2004, Lehmann and colleagues were able to access the anionic borate, [B(CF_3_)_3_CP]^−^ (B) by the reaction of an acyl–halide complex [B(CF_3_)_3_C(O)X]^−^ (X = Cl, Br) with K[P(SiMe_3_)_2_].^4^ This compound can be understood to be a Lewis acid–base adduct of a borane with a cyaphide ion. Limited follow-up reactivity studies have been reported for these landmark molecules.^5^ The first isolable metal complex featuring a terminal cyaphido ligand was reported two years later by Grützmacher. This species, the ruthenium(ii) cyaphido complex *trans*-Ru(dppe)_2_H(CP) (C; dppe = 1,2-bis(diphenylphosphino)-ethane), was accessed by desilylation of the cationic *κ*^1^-phosphaalkyne compound *trans*-[Ru(dppe)_2_H(PCSiPh_3_)]^+^.^6^ This platform has been explored by Crossley and co-workers for the synthesis of an array of metal complexes with which to probe the *trans*-influence of the cyaphide ligand.^7–10^ In 2017, Meyer and co-workers reported the synthesis of a bimetallic uranium(iv) species featuring a terminal cyaphide ligand, {U[N(CH_2_ArO)_3_](DME)}(µ–O){U[N(CH_2_ArO)_3_](CP)} (D),^11^ which was accessed by reaction of sodium phosphaethynolate, NaPCO,^12^ with two equivalents of the uranium(iii) complex U[N(CH_2_ArO)_3_](DME) (Ar = 3-(1-adamantyl)-5-methyl-phenyl; DME = dimethoxyethane). This synthesis illustrates that reductive C–O cleavage of phosphaethynolates can be used to access metal cyaphido compounds. We tested this hypothesis by reacting PCOSi^*i*^Pr_3_ ^13^ with Jones' strongly reducing (and highly oxophilic) magnesium(i) complex [Mg(^Dipp^NacNac)]_2_,^14^ which was found to afford an equimolar mixture of Mg(^Dipp^NacNac)(diox)(CP) (E) and Mg(^Dipp^NacNac)(diox)(OSi^*i*^Pr_3_).^15^ Since then, we have shown that E acts as a Grignard reagent allowing for cyaphide transfer in a number of salt–metathesis reactions, and access to a range of metal compounds containing the CP moiety.^16–23^

In a recent report, Müller, Jones and co-workers demonstrated that platinum(0) complexes are able to photolytically activate the C(sp)–C(sp^2^) bond of aryl-phosphaalkynes, specifically PCMes and PCTripp (Mes = 2,4,6-trimethylphenyl; Tripp = 2,4,6-tri(isopropyl)phenyl), to afford platinum(ii) cyaphido complexes (F).^24^ In this elegant study, the authors showed that such oxidative addition reactions are thermodynamically uphill and reversible. Upon heating, the cyaphido complexes reverted back to platinum(ii) π-complexes of the phosphaalkyne. This inspired us to explore whether other single-site oxidative addition reactions are viable, in order to increase the atom-economy of reductive bond cleavage reactions such as those described above. We reasoned that the aluminium(i) carbenoid Al(^Dipp^NacNac)^25,26^ should have comparable reductive capacity to magnesium(i) and uranium(iii) complexes, and that it might be used to cleave the C–O bond in PCOSi^*i*^Pr_3_. The coordinatively unsaturated metal centre would also be capable of bonding to both the anionic species generated (*i.e.*, the cyaphide ion and the tri(isopropyl)siloxide ion). The results of these studies are reported.

## Results and discussion

### Cyaphide generation through single-site oxidative addition

**Scheme 1:**
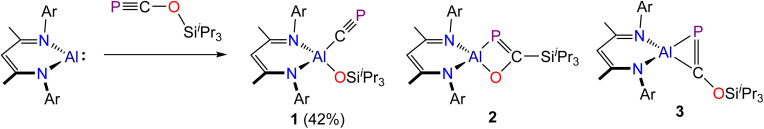
Synthesis of 1–3. Ar = Dipp (2,6-diisopropylphenyl). Isolated yield of 1 in parentheses.

Addition of a hexane solution of PCOSi^*i*^Pr_3_ to Al(^Dipp^NacNac) at room temperature results in a colour change of the reaction mixture from light-yellow to dark-brown over the course of 15 minutes. The ^31^P{^1^H} NMR spectrum of the crude reaction mixture exhibits three singlet phosphorus resonances at 128.3, 164.4 and 344.9 ppm (in a 6 : 3 : 1 ratio), indicating the presence of three phosphorus-containing compounds, all of which were later determined to be constitutional isomers (1–3, respectively; Scheme 1). Running these reactions at low temperature (−100 °C) reveals the presence of only compounds 1 and 2 in the crude reaction mixtures.

Fractional crystallization from such reaction mixtures allowed us to structurally authenticate all the species present in solution. A compositionally pure sample of Al(^Dipp^NacNac)(OSi^*i*^Pr_3_)(CP) (1) can be isolated in moderate yield (up to 42%) from the first crop of crystals obtained by storing these hexane solutions at −35 °C. Single crystal X-ray diffraction confirmed formation of the target compound (*vide infra*). Redissolution of these crystals in C_6_D_6_ revealed a single singlet resonance in the ^31^P{^1^H} NMR spectrum at 128.3 ppm, which is notably up-field relative to the chemical shift of more ionic cyaphido complexes such as Mg(^Dipp^NacNac)(diox)(CP) (^31^P{^1^H}: 177.2 ppm).^15^ A broad resonance corresponding to the cyaphide carbon atom was observed in the ^13^C{^1^H} NMR spectrum at 230.96 ppm, which is also up-field relative to that of the magnesium complex (^13^C{^1^H}: 270.97 ppm; ^1^*J*_C–P_ = 34.0 Hz). No ^13^C–^31^P coupling was observed in this spectrum, presumably because the carbon atom is bonded to a quadrupolar ^27^Al nucleus (*S* = 5/2; 100% natural abundance). The ^1^H NMR spectrum of 1 is consistent with the presence of a single β-diketiminato and tri(isopropyl)siloxide ligand environment. The IR spectrum of 1 reveals a band at 1372 cm^−1^ arising from the C≡P stretching mode, also consistent with previously reported values for related complexes.

**Fig. 2:**
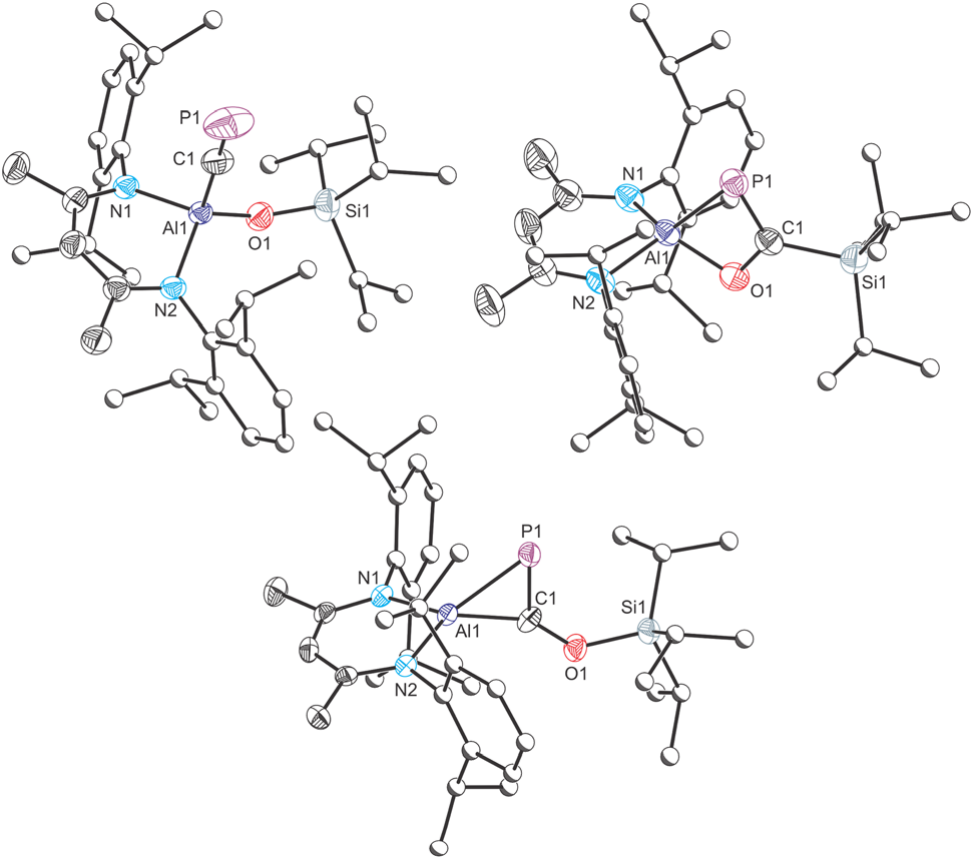
Molecular structures of 1 (top left), 2 (top right) and 3 (bottom) as determined by single crystal X-ray diffraction. Thermal ellipsoids set at 50% probability level; hydrogen atoms omitted for clarity. Carbon atoms of Dipp and ^*i*^Pr groups are depicted as spheres of arbitrary radius. Selected interatomic distances [Å] and angles [°] 1: Al1–C1 1.962(3), C1–P1 1.549(3), Al1–O1 1.702(2), Al1–C1–P1 176.66(18); 2: Al1–P1 2.309(2), Al1–C1 2.292(3), Al1–O1 1.788(2), C1–P1 1.735(3), C1–O1 1.381(4), C1–Si1 1.886(3), Al1–P1–C1 67.34(10), Al1–O1–C1 91.73(17), P1–C1–O1 119.6(2); 3: Al1–P1 2.287(2), Al1–C1 1.885(3), C1–P1 1.724(3), C1–O1 1.362(3), Al1–C1–P1 78.51(11), Al1–P1–C1 53.87(10), C1–Al1–P1 47.61(10).

The molecular structure of 1, as determined by single crystal X-ray diffraction (Fig. 2, top left), reveals the characteristic pseudo-tetrahedral coordination mode typical of aluminium(iii) complexes. The C≡P bond length of 1, 1.549(3) Å, is consistent with those found for other metal cyaphido complexes (*e.g.* Mg(^Dipp^NacNac)(diox)(CP): 1.553(2) Å). The M−C≡P bond angle, 176.7(2)°, is slightly deviated from linearity, which we attribute to the steric demands of both the ^Dipp^NacNac and OSi^*i*^Pr_3_ ligands.

Isolation of the supernatant solution from the mixture that afforded 1 and subsequent recrystallization afforded a second crop of crystals: a mixture of colourless and dark red crystals. The latter were mounted and characterized by single-crystal X-ray diffraction, revealing the formation of compound 3 (Fig. 2, bottom). This compound is an isomer of 1, which has not undergone reductive cleavage of the C–O bond of PCOSi^*i*^Pr_3_, but rather a [2 + 1]-cyclometallation reaction. Such phospha-aluminirenes have previously been observed by Stephan on reaction of Al(^Dipp^NacNac) with phosphaalkynes such as PC^*t*^Bu and PCAd (Ad = adamantyl).^27^

The structure of 3 reveals a three-membered cyclic ring in which the aluminium centre binds to both the phosphorus and carbon atoms of the siloxy-phosphaalkyne with bond distances of 2.287(2) and 1.885(3) Å, respectively. These distances are comparable to those of related phosphaaluminirenes.^27^ The P1–C1 bond in 3, 1.724(3) Å, is notably longer than that observed for 1 (1.549(3) Å), consistent with the formal description of the former as a double bond, while the latter can be considered a triple bond. These data compare well with the predicted values for P–C double (1.69 Å) and triple bonds (1.54 Å).^28,29^

Dissolution of these dark-red crystals in C_6_D_6_ revealed a major singlet resonance in ^31^P{^1^H} NMR spectrum at 344.9 ppm, originating from 3, along with minor resonances at 128.3 and 164.4 ppm, corresponding to 1 and 2, respectively. Phospha-aluminirenes obtained by reaction of PC^*t*^Bu and PCAd with Al(^Dipp^NacNac) exhibit singlet resonances at 514.3 and 517.1 ppm, respectively. The more shielded resonance of 3 may arise from the π-donating character of the siloxy group. Also worth noting is that the Si1⋯P1 interatomic distance in the structure of 3 is approx. 3.5 Å which may also contribute to the observed difference in chemical shifts. Over the course of several days at room temperature, the resonance arising from 3 diminishes in intensity giving rise to 1 and 2.

One final crop of off-white crystals could be obtained from the mother liquor of the reaction mixture when left to slowly evaporate at −35 °C. Analysis of this sample by single-crystal X-ray diffraction reveals a solid-solution of two species, the four-membered metallacycle 2 (Fig. 2, top right), which co-crystallizes with 1. The structural motif present in 2 has been previously reported in separate studies by Roper,^30,31^ Cummins^32^ and Inoue.^33^ There are four molecules present in the asymmetric unit of this sample. Only one of these can be modelled as a disorder-free molecule of 2. This species reveals a short P=C bond (1.735(3) Å) comparable to that of 3 (1.724(3) Å) and other related species such as Inoue's silicon-containing heterocyclic compound (1.732(3) and 1.733(3) Å).^33^ The ^31^P{^1^H} NMR spectrum of this sample displays an enrichment in the concentration of 2 (observed at 164.4 ppm) which is accompanied by the minor resonances at 128.3 and 344.9 ppm, corresponding to 1 and 3, respectively.

### Computational studies: mechanism and bonding

**Fig. 3:**
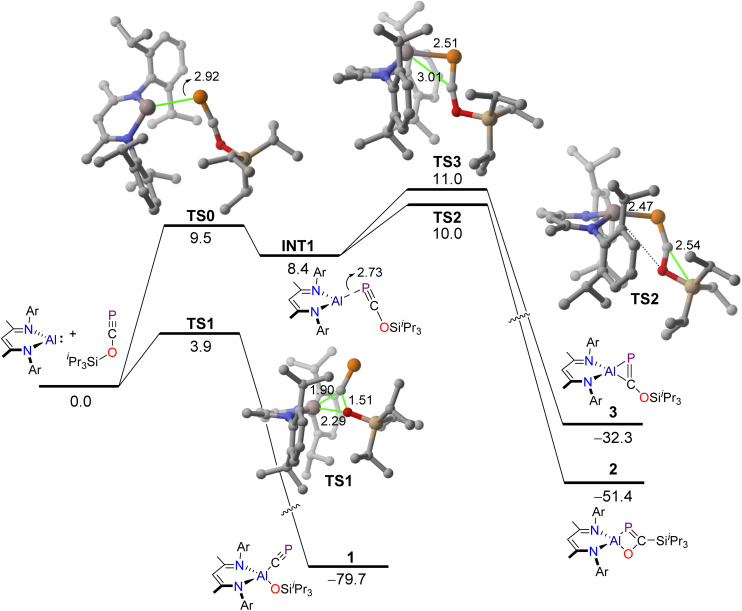
Computed reaction profiles for the formation of compounds 1–3. Relative free energies (Δ*G*, at 298 K) and bond distances are given in kcal mol^−1^ and Ångstroms, respectively. All data have been computed at the PCM-BP86-D3/def2-TZVPP//PCM-BP86-D3/def2-SVP level. Hydrogen atoms omitted for clarity.

Density Functional Theory (DFT) calculations at the dispersion corrected PCM-BP86-D3/def2-TZVPP//PCM-BP86-D3/def2-SVP level were first carried out to gain more insight into the mechanisms governing the formation of the species 1–3 from the reaction of PCOSi^*i*^Pr_3_ and Al(^Dipp^NacNac). As shown by the computed reaction profile in Fig. 3, the formation of the title compound 1 derives from an oxidative addition reaction through the three-membered transition state TS1. This saddle point is associated with the rupture of the C–O bond in the PCOSi^*i*^Pr_3_ reagent with the concomitant formation of the Al–C(P) and Al–O bonds, and therefore strongly resembles the transition states previously computed for related oxidative additions of other σ-bonds mediated by aluminium(i) carbenoids.^34^ Interestingly, the computed activation barrier for this transformation is rather low (Δ*G*^‡^ = 3.9 kcal mol^−1^). This, together with the highly exergonic nature of the process (Δ*G* = −79.7 kcal mol^−1^), is reflected in the ease of the reaction, which is consistent with a process occurring at room temperature and even at −100 °C, as found experimentally (see above).

Isomeric compounds 2 and 3 are both formed from the same common species INT1, which lies 8.4 kcal mol^−1^ above the separate reactants and can be viewed as a fleeting intermediate where the phosphorus atom of PCOSi^*i*^Pr_3_ is weakly bonded to the aluminium(i) centre. The formation of these isomers is also strongly exergonic (Δ*G* = −51.4 kcal mol^−1^ and −32.3 kcal mol^−1^, respectively) and occurs *via* transition states TS2 and TS3, with low barriers as well (Δ*G*^‡^ = 10.0 kcal mol^−1^ and 11.0 kcal mol^−1^, respectively). While TS3 is associated with the formation of the new Al–C bond in a formal [2 + 1] reaction, TS2 is mainly associated with the migration of the Si^*i*^Pr_3_ moiety from the oxygen atom to the adjacent carbon atom.

The spectroscopic data commented above strongly suggest that the degree of covalency of the Al–CP bond in the title compound 1 is much more pronounced than that in more ionic cyaphido complexes such as Mg(^Dipp^NacNac)(diox)(CP), (E). To provide further quantitative support to this finding, we then compared the bonding situations in 1 and its Mg(ii) counterpart with the help of the Energy Decomposition Analysis (EDA) method (see computational details in the SI). To this end, the interaction between the *κ*^1^-cyaphide anion with cationic [(^Dipp^NacNac)Al(OSi^*i*^Pr_3_)]^+^ and [(^Dipp^NacNac)Mg(diox)]^+^ fragments was computed. From the data in Table 1, it becomes clear that the Al–CP interaction is clearly stronger than the Mg–CP interaction (Δ*E*_int_ = −178.7 kcal mol^−1^*vs.* −153.7 kcal mol^−1^, respectively), which is consistent with the corresponding computed NBO-Wiberg Bond Orders: 0.51 (1) > 0.19 (E). Partitioning of the Δ*E*_int_ term into its energy contributors indicates that the electrostatic term, Δ*E*_elstast_, which is a measure of the ionic bonding, dominates in both species, which is not surprising due to the charged nature of the considered fragments. Despite that, the relative contribution of the ionic bonding is clearly higher in the Mg(ii) complex than in 1 (74% *vs.* 66%), and consequently, the covalent bonding, measured by the Δ*E*_orb_ term, is higher in the Al(iii) compound (31% *vs.* 22%). Therefore, our EDA calculations nicely confirm the higher covalent nature of the Al–CP bond as compared to Mg–CP.

**Table 1:** EDA data (in kcal mol^−1^) for compounds 1 and E computed at the ZORA-BP86-D3/TZ2P//PCM-BP86-D3/def2-SVP level

	[Al^III^]–CP (1)	[Mg^II^]–CP (E)
Δ*E*_int_	−178.7	−153.7
Δ*E*_Pauli_	135.4	73.6
Δ*E*_elstast_^a^	−207.4 (66%)	−166.7 (74%)
Δ*E*_orb_^a^	−95.7 (31%)	−50.8 (22%)
Δ*E*_disp_^a^	−11.0 (3%)	−9.9 (4%)

a Percentages in parentheses refer to the relative contributions to the total attractive interactions Δ*E*_elstat_+ Δ*E*_orb_ + Δ*E*_disp_.

### Cyclization reactions

Organoaluminium complexes are typically less reactive than their organomagnesium analogues. This difference arises from the greater polarity of Mg–C bonds relative to Al–C bonds, as shown previously. Consequently, Grignard reagents exhibit vigorous reactivity towards electrophiles, whereas organoaluminium compounds often require elevated temperatures and prolonged reaction times to achieve similar transformations.^35^ This prompted us to explore the reactivity of 1 towards common laboratory reagents. Our studies show that many substrates that are known to react with Mg(^Dipp^NacNac)(diox)(CP) (E), such as electrophiles (*e.g.* aldehydes, ketones) and metal halides (*e.g.* Au(IDipp)Cl), are largely unreactive towards 1. We have observed no evidence of cyaphide group transfer thus far; however we are able to engage the C≡P triple bond in several cyclization reactions.

**Scheme 2:**
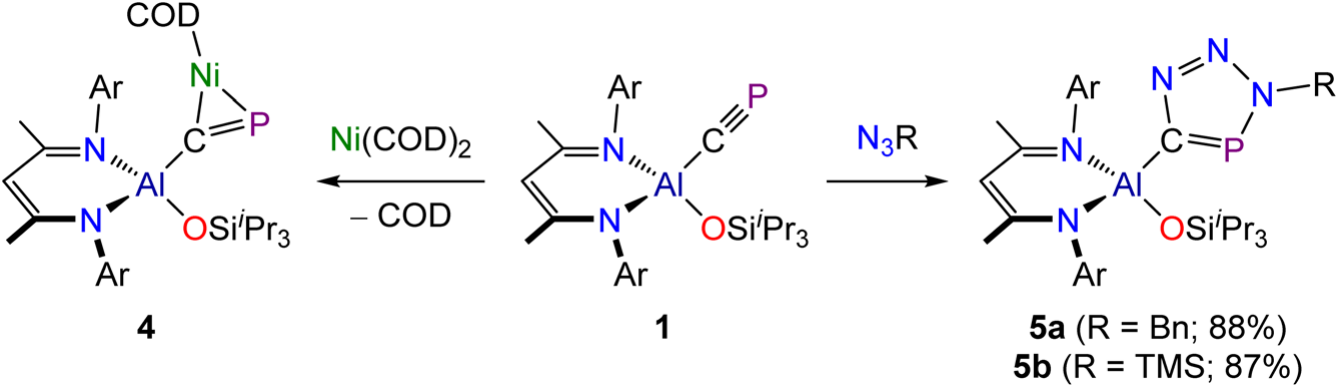
Synthesis of 4 and 5a/5b.

Phosphaalkynes (P≡C–R) have previously been investigated as effective precursors for [2 + 1] cycloaddition reactions. Reported examples primarily involve the use of organic carbenes^36^ and main-group element carbenes analogues.^27,37,38^ Some d-metal complexes have also been shown to participate in [2 + 1]-cyclometallation with phosphaalkynes.^39^ However, in selected cases, phosphaalkynes have been also shown to undergo oligomerization reactions upon coordination to the d-metal centres due to the high reactivity of the intermediate species and the lack of steric protection.^40^ To determine whether such cyclization reactions are possible on our aluminium platform, we reacted 1 with Ni(COD)_2_ (Scheme 2). Addition of one equivalent of Ni(COD)_2_ to a solution of 1 results in an immediate darkening of the solution and the quantitative formation of a new species as determined by NMR spectroscopy. The resulting compound, Al(^Dipp^NacNac)(OSi^*i*^Pr_3_)(µ_2_–CP)Ni(COD), 4, exhibits a singlet in its ^31^P{^1^H} NMR spectrum at 315.5 ppm and a broadened resonance at 257.58 ppm in its ^13^C{^1^H} spectrum. These values are comparable to those of other cyclometallated cyaphido compounds, *e.g.* (IDipp)Au(µ_2_–CP)Ni(MeI^*i*^Pr)_2_.^16^ In addition to the resonances assigned to 4, the ^1^H NMR spectrum of the reaction mixture also exhibits two resonances due to free COD which integrate in a 2 : 1 ratio (*δ*: 2.22 and 5.58 ppm). Compound 4 was found to slowly decompose in solution over the course of several days, however crystals of the compound could be obtained from a concentrated hexane solution.

**Fig. 4:**
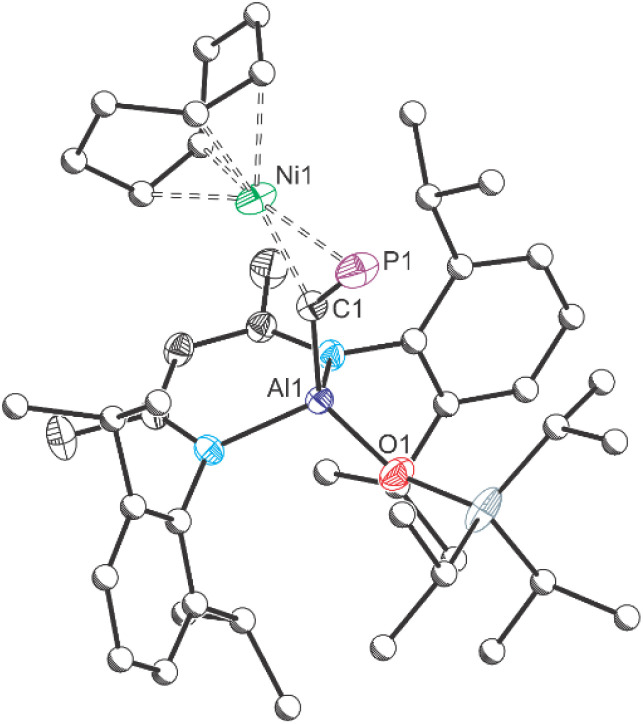
Molecular structure of 4 as determined by single crystal X-ray diffraction. Thermal ellipsoids set at 50% probability level; hydrogen atoms omitted for clarity. Carbon atoms of Dipp, ^*i*^Pr and COD groups are depicted as spheres of arbitrary radius. Selected interatomic distances [Å] and angles for one of the crystallographically unique molecules in the asymmetric unit [°]: Al1–C1 1.957(2)/1.958(2), C1–P1 1.628(2)/1.630(2), Al1–O1 1.709(2)/1.711(2), Ni1–C1, 1.936(3)/1.930(2), Ni1–P1, 2.172(1)/2.171(1); Al1–C1–P1 141.08(11)/140.18(12).

The crystal structure of 4 reveals a square planar coordination environment about the nickel centre with the cyaphide moiety bonded in a “side-on” *η*^2^-coordination mode to the metal (Fig. 4). The C–P bond length in 4, measured at 1.628(2) and 1.630(2) Å for the two molecules present in the asymmetric unit, is notable longer than that of 1 (1.549(3) Å), and suggests significant π-backdonation from the nickel centre to a π-antibonding orbital of the cyaphido ligand. The P–C–Al angle is 141.08(11) and 140.18(12)°, which markedly differs from the almost linear value, 176.5(2)°, observed for precursor 1. The C–Ni bond lengths are 1.936(2) and 1.930(2) Å and P–Ni distances are 2.172(1) and 2.171(1) Å within the triangular metallacyclo-phosphapropene unit.

Phosphalkynes are known to take part in [2 + 3] cycloaddition reactions with organic azides.^41^ Several metal cyaphido compounds have also been shown to afford metalated phosphatriazoles on reaction with azides.^19,20,24^ In an effort to establish whether the cyaphide moiety in 1 retains this reactivity, we reacted 1 with two azides (N_3_Bn and N_3_TMS; Bn = benzyl; TMS = trimethylsilyl). Both reactions quantitatively afford [2 + 3] cyclization products regio-selectively (Scheme 2), with the benzyl azide reaction proceeding rapidly (*i.e.* before NMR data could be acquired), while the reaction with trimethylsilyl azide required three days at room temperature. We attribute this difference to the greater steric demands of the N_3_TMS. The reactions were monitored by ^31^P{^1^H} NMR spectroscopy until full conversion to the products was observed. New singlet resonances appeared in the ^31^P NMR spectra of the reaction mixtures at 221.6 and 238.2 ppm for Al(^Dipp^NacNac)(OSi^*i*^Pr_3_)(CPN_3_R) where R = Bn (5a) and TMS (5b), respectively. Broad resonances were also observed in the ^13^C{^1^H} NMR spectra of these compounds at 192.14 and 189.12 ppm for the carbon atoms of the phosphatriazole rings in 5a and 5b, respectively. The ^1^H NMR spectra were consistent with the presence of a single β-diketiminato ligand, as evidenced by single resonances in the region expected for the γ-H of the ligand backbone (*δ*: 5.20 and 5.26 ppm for 5a and 5b, respectively).

**Fig. 5:**
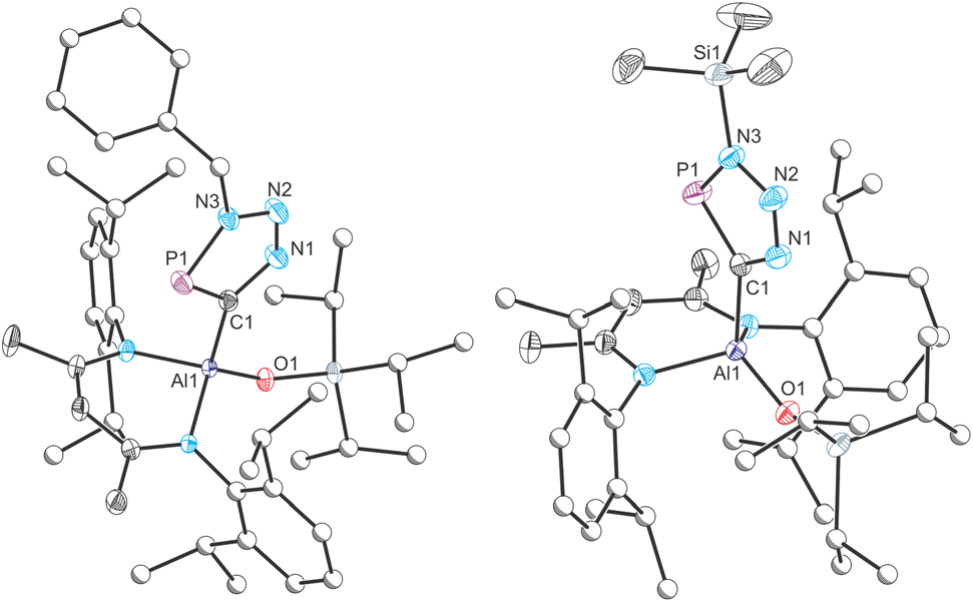
Molecular structures of 5a (left) and 5b (right) as determined by single crystal X-ray diffraction. Thermal ellipsoids set at 50% probability level; hydrogen atoms and solvent of crystallization omitted for clarity. Carbon atoms of Dipp, ^*i*^Pr and Bn groups are depicted as spheres of arbitrary radius. Selected interatomic distances [Å] and angles [°] 5a: Al1–C1 1.986(2), C1–P1 1.720(2), C1–N1 1.377(2), N1–N2 1.310(2), N2–N3 1.345(2), N3–P1 1.689(2), Al1–O1 1.699(1), Al1–C1–P1 125.28(8); 5b: Al1–C1 1.988(5), C1–P1 1.720(4), C1–N1 1.373(4), N1–N2 1.299(3), N2–N3 1.367(4), N3–P1 1.685(4), Al1–O1 1.704(2), Al1–C1–P1 126.5(3).

The structures of compounds 5a and 5b were determined by single-crystal X-ray diffraction (Fig. 5). Each reveals a planar five-membered CPN_3_ moiety, characteristic of other metallo-phosphatriazoles. Upon cyclization with azides, the C–P distance increases from 1.549(2) Å in 1 to *ca.* 1.720 Å in complexes 5a and 5b, consistent with the bond lengths reported for other triazaphospholes.^19,20,24^ The Al–C bond of 1.962(2) Å in 1 remains largely unchanged in 5a and 5b (*ca.* 1.987 Å).

**Scheme 3:**
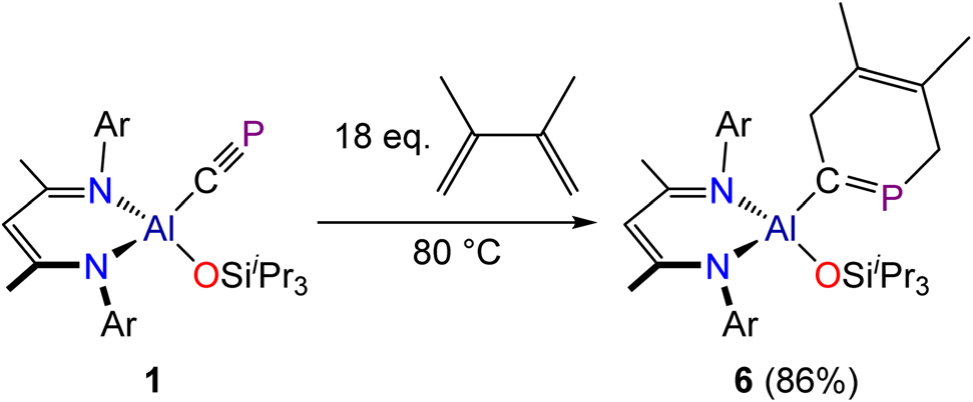
Synthesis of 6.

To establish the extent of viable cyclization reactions available to the cyaphide functional group, compound 1 was reacted with 2,3-dimethyl-1,3-butadiene. This reaction was found to proceed slowly, requiring forcing reaction conditions (80 °C) and a significant stoichiometric excess of 2,3-dimethyl-1,3-butadiene. We attribute this reduced reactivity to the steric crowding about the cyaphide moiety in 1, as more electron-rich but bulkier dienes, such as Danishefsky's diene (*trans*-1-methoxy-3-trimethylsilyloxy-buta-1,3-diene) were found to be unreactive towards 1. The reaction between 1 and 2,3-dimethyl-1,3-butadiene was found to afford a six-membered cyclic product, 6, which features a 1-phosphacyclo-hexa-1,4-diene core (Scheme 3). Upon completion of the reaction, the excess butadiene was removed under a dynamic vacuum and the product crystallized from a concentrated hexane solution at −35 °C. This is the first example of a [2 + 4] cyclization product obtained from a cyaphido complex. Related phospha-cyclohexadienes have been generated transiently by reaction of phosphaalkynes with butadienes, however coordination to a metal complex was required for their isolation.^42^

**Fig. 6:**
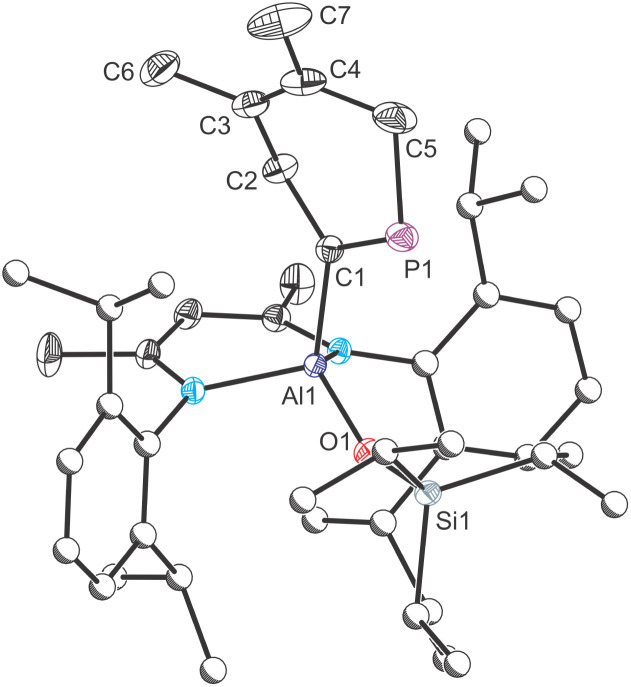
Molecular structure of 6 as determined by single crystal X-ray diffraction. Thermal ellipsoids set at 50% probability level; hydrogen atoms omitted for clarity. Carbon atoms of Dipp and ^*i*^Pr are depicted as spheres of arbitrary radius. Selected interatomic distances [Å] and angles [°]: Al1–C1 1.9814(16), C1–P1 1.6719(16), C1–C2 1.534(2), C2–C3 1.512(2), C3–C4 1.331(3), C4–C5 1.501(3), C5–P1 1.859(2), C3–C6 1.506(3), C4–C7 1.506(3), Al1–O1 1.7101(12), Al1–C1–P1 119.95(9).

Crystallographic analysis of 6 confirmed the formation of the [2 + 4]-cycloaddition product (Fig. 6). The Al1–C1 bond length was determined to be 1.982(2) Å which is slightly longer than that observed for compounds 5a and 5b, an observation we attribute to the greater steric bulk of the phospha-cyclohexadiene moiety. The C1=P1 bond length, measured at 1.672(2) Å, is consistent with a C=P double bond as observed for phospha-alkenes (1.61–1.71 Å).^28^ The C3–C4 bond length, 1.331(3) Å, is also as expected for a C=C double bond, while all the other bond lengths of the heterocycle adopt values consistent with single bonds. In the solid state, the six-membered heterocyclic fragment adopts a boat-like conformation (Fig. 6). The C1, P1, C3 and C4 atoms are located within the same plane, with some ring distortion arising due to the larger covalent radius of phosphorus atom. The dihedral angles between the C1–P1–C3–C4 plane and the P1–C5–C4 and C1–C2–C3 planes are 141.3° and 145.2°, respectively (see Fig. S81 in the SI). These values are in line with the folding angle of the central ring in 9,10-dihydroantracene, reported as 144.7°.^43^

The ^31^P NMR spectrum of a compositionally pure sample of 6 displays three multiplet resonances at 290.8, 284.0, 273.4 ppm, with the signal at 284.0 ppm being the predominant species (relative integrations: 0.05 : 1 : 0.1). The ^1^H–^31^P coupling constants range from 15 to 18 Hz for the three resonances. Interestingly, only two distinct sets of signals were detected by ^1^H NMR spectroscopy. The observed ratio of products remains constant even after several consecutive recrystallizations of 6 or under variable reaction conditions. Given the steric hindrance around the Al1–O1 and Al1–C1 bonds, we attribute this observation to the presence of a conformational equilibrium. The region between aliphatic and aromatic resonances in ^1^H NMR spectrum enables an unobstructed view of the reaction mixture. Integration displays a ratio of approximately 0.18 to 1.00 between the two observed sets of signals. A similar ratio is observed in ^31^P NMR spectrum, where the two minor resonances (290.8 and 273.4 ppm) integrate to a combined 0.15 relative to the major peak at 284.0 ppm. The experimental data suggests the presence of at least three conformers in solution, two of which exchange rapidly on the ^1^H NMR timescale, while being distinguishable in the ^31^P NMR spectrum. Further analysis of 6 using ^1^H–^1^H EXSY and variable temperature NMR experiments supports the presence of chemical exchange between the conformers (see Fig. S55 and S60 in the SI). A computational analysis of 6 allowed us to locate four distinct conformers on the potential energy surface (see Fig. S83 in the SI). Based on their calculated relative free energies, two of these species, 6-I and 6-II, both featuring boat-like six-membered rings, are nearly degenerate (ΔΔ*G* = 0.5 kcal mol^−1^). These have the same orientation of the six-membered heterocycle relative to the [(^Dipp^NacNac)Al(OSi^*i*^Pr_3_)]^+^ support, with the lowest energy conformer, 6-I, matching the structure determined by SXRD. The two remaining conformers, 6-III and 6-IV, result from the rotation around the Al–C bond in 6-I and 6-II and lie higher in energy relative to 6-I (ΔΔ*G* = 1.6 kcal mol^−1^ and 2.4 kcal mol^−1^, respectively).

**Fig. 7:**
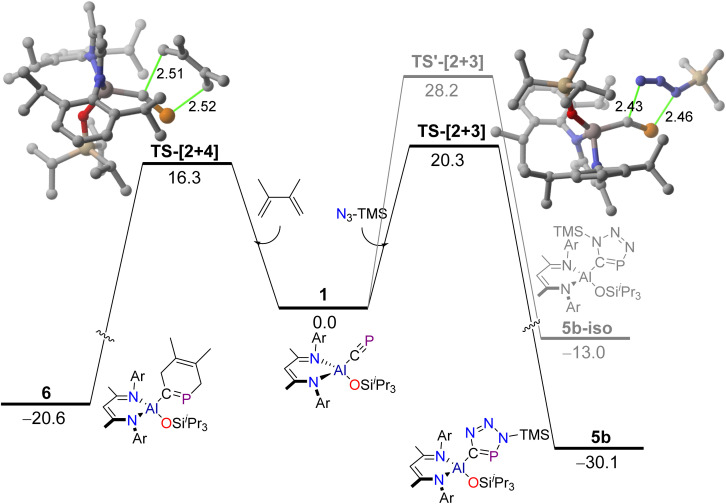
Computed reaction profiles for the formation of cycloadducts 5b and 6. Relative free energies (Δ*G*, at 298 K) and bond distances are given in kcal mol^−1^ and angstroms, respectively. All data have been computed at the PCM-BP86-D3/def2-TZVPP//PCM-BP86-D3/def2-SVP level. Hydrogen atoms omitted for clarity.

We also performed DFT calculations to explore the mechanisms involved in the above-described [2 + 3] and [2 + 4] cycloaddition reactions. As shown in Fig. 7, both processes proceed in a concerted manner through the corresponding five-membered or six-membered transition states with feasible activation barriers (Δ*G*^‡^ = 20.3 and 16.3 kcal mol^−1^, respectively) and in highly exergonic reactions (Δ*G* = −30.1 and −20.6 kcal mol^−1^). This is similar to related [2 + 3] cycloaddition reactions involving azides and cyaphide metal complexes, which also occur in a concerted manner and with complete regioselectivity.^44,45^ Indeed, our calculations confirm that the formation of the alternative regioisomer where the substituted nitrogen atom of the azide would bind the carbon atom of the cyaphide is both kinetically (ΔΔ*G*^‡^ = 7.9 kcal mol^−1^) and thermodynamically (ΔΔ*G* = 17.1 kcal mol^−1^) unfavoured, which is fully consistent with the exclusive formation of cycloadducts 5a and 5b (having the substituted nitrogen atom of the azide attached to the phosphorus atom).

### Heterocycle functionalization

**Scheme 4:**
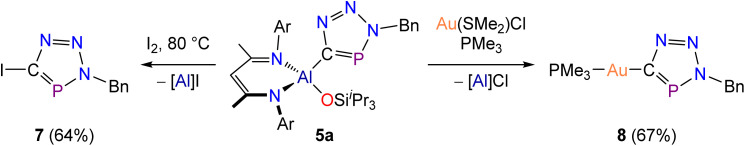
Synthesis of 7 and 8. [Al] = Al(^Dipp^NacNac)(OSi^*i*^Pr_3_).

**Fig. 8:**
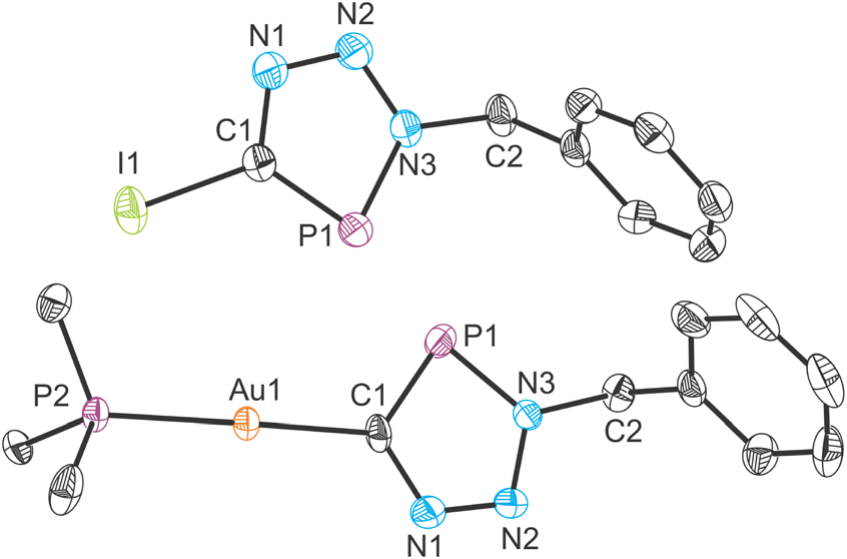
Molecular structures of 7 (top) and 8 (bottom) as determined by single crystal X-ray diffraction. Thermal ellipsoids set at 50% probability level; hydrogen atoms omitted for clarity. Selected interatomic distances [Å] and angles [°] 7: C1–I1 2.072(8), C1–P1 1.710(8), C1–N1 1.352(11), N1–N2 1.314(10), N2–N3 1.339(10), N3–P1 1.690(7), N3–C2 1.486(10); 8: C1–Au1 2.050(6), P2–Au1 2.289(2), C1–P1 1.730(7), C1–N1 1.358(9), N1–N2 1.315(8), N2–N3 1.343(8), N3–P1 1.688(6), N3–C2 1.481(8).

The utility of the aforementioned cyclization reactions is limited if the resulting heterocycles cannot be detached from the Al(^Dipp^NacNac)(OSi^*i*^Pr_3_) platform. This prompted us to explore the reactivity of 5a towards iodination and transmetallation reactions as a proof-of-concept. Thus, a mixture of 5a with elemental iodine was heated for 1 hour at 80 °C, which resulted in quantitative consumption of the metallo-phosphatrizole compound and formation of the iodo-phosphatriazole 7 (Scheme 4, left). The functionalized heterocycle 7 exhibits nearly identical solubility to that of the by-product Al(^Dipp^NacNac)(OSi^*i*^Pr_3_)I (identified by ^1^H NMR spectroscopy), making the recrystallization impractical. Therefore, a successful separation was achieved by column chromatography on SiO_2_ using benzene as the eluent. The isolated yield of 7 was 64%. The ^31^P{^1^H} NMR spectrum of 7 reveals a singlet resonance at 187.8 ppm, which is consistent with values reported for related iodo-derivatized triazaphospholes.^19^ The crystal structure of 7 features the planar heterocyclic fragment with a C–I bond distance of 2.076(4) Å (Fig. 8, top).

As an alternative functionalization strategy, transmetallation was achieved *via* the reaction of 5a with gold(i) complex Au(SMe_2_)Cl (Scheme 4, right). Immediate transfer of the heterocyclic moiety was confirmed by the appearance of a ^31^P{^1^H} resonance of 205.1 ppm corresponding to Au(SMe_2_)(CPN_3_Bn). However, isolation of this compound proved challenging due to its light and vacuum sensitivity. Thus, in order to stabilize the gold(i) complex, we performed a ligand exchange reaction with PMe_3_. The resulting vacuum stable complex, Au(PMe_3_)(CPN_3_Bn), 8, displays two ^31^P{^1^H} NMR resonances at 201.5 and 3.4 ppm (^2^*J*_P–P_ = 35 Hz) corresponding to the triazaphosphole and the trimethylphosphine, respectively.

The crystal structure of 8 exhibits a linear geometry, which is characteristic of gold(i) complexes (Fig. 8, bottom). The crystal structure features two unique molecules in the asymmetric unit. The Au–C bond length is at 2.050(6) and 2.042(7) Å, while the Au–P bond is determined to be 2.289(2) and 2.287(2) Å. The C–Au–P angles are 175.7(2) and 178.6(2)°. In addition, the crystal structure of 7 reveals a short Au⋯Au interaction, with the distance between the two gold centres measured at 3.274(1) Å (see SI). This is relatively long when compared to reported distances for aurophilic interactions which range from 2.7 to 3.3 Å.^46,47^

Due to the limited scope of available phosphaalkynes, triazaphospholes formed *via* reactions with organic azides are not easily accessible. The structural diversity of triazaphospholes can only be tuned by varying the employed azide reagents. Compounds 7 and 8 enable a new strategic pathway for the synthesis of functionalized triazaphospholes allowing for control over both *exo*-cyclic groups.

## Conclusions

We show that the synthesis of a novel aluminium cyaphide complex – Al(^Dipp^NacNac)(OSi^*i*^Pr_3_)(CP) (1) – is possible *via* the single-site oxidative addition of the C–O bond in PCOSi^*i*^Pr_3_ at an aluminum(i) metal centre. This process is accompanied by two competing side-reactions, both of which involve the formation of a common intermediate in which the phosphorus lone-pair of PCOSi^*i*^Pr_3_ interacts with the electrophilic aluminium(i) centre. Formation of a P–O bond, with concomitant silyl-group migration to the carbon atom, affords the four-membered metallacycle Al(^Dipp^NacNac)[*κ*^2^-P(O)CSi^*i*^Pr_3_] (2). Alternatively, the intermediate can form a P–C bond, giving rise to the formal [2 + 1] cyclometallation product Al(^Dipp^NacNac)(*η*^2^-PCOSi^*i*^Pr_3_) (3). Compound 1 differs from other related organometallic cyaphido complexes in both the low polarity of the Al–C bond (which imparts it with properties typically associated with phospha-alkynes) and by the extreme steric protection of the cyaphide moiety offered by the β-diketiminato and siloxide ligands.

Despite this diminished reactivity, 1 was shown to undergo quantitative conversion into a [2 + 1] cyclometallation product upon reaction with Ni(COD)_2_. High-yielding protocols for the [2 + 3] and [2 + 4] cycloadditions with organic azides and 2,3-dimethyl-1,3-butadiene, respectively, were also developed and found to proceed concertedly. Finally, the functionalization of the triazaphosphole complex 5a was achieved *via* both the iodination and the transmetallation onto the gold(i) metal centre. Taken together, these observations show that cyaphido complex 1 provides a sterically protected, highly stable platform for the construction and functionalization of phosphorus-containing heterocycles.

## Author contributions

Conceptualization: A. Y. and J. M. G.; experimental work: A. Y. and S. J. U.; X-ray crystallography: A. Y., S. J. U., A. G.-R. and J. M. G.; computational modelling: I. F.; writing – original draft: A. Y., I. F. and J. M. G.; writing & editing: all authors; supervision: J. M. G.; funding acquisition: I. F. and J. M. G.

## Conflicts of interest

There are no conflicts to declare.

## Supplementary Material

SC-017-D6SC03023H-s001

SC-017-D6SC03023H-s002

## Data Availability

CCDC 2531460 (1), 2531461 (2), 2531462 (3), 2531463 (4), 2531464 (5a), 2531465 (5b·hex), 2531466 (6), 2531467 (7) and 2531468 (8) contain the supplementary crystallographic data for this paper.^48*a–i*^ The data supporting this article have been included as part of the supplementary information (SI). Supplementary information: experimental procedures, analytical data for all novel compounds (NMR, IR and HRMS spectra), single-crystal X-ray diffraction collection and refinement details, and computational details. See DOI: https://doi.org/10.1039/d6sc03023h.

## References

[cit1] Görlich T., Coburger P., Yang E. S., Goicoechea J. M., Grützmacher H., Müller C. (2023). Angew. Chem., Int. Ed..

[cit2] Jun H., Young Jr. V. G., Angelici R. J. (1992). J. Am. Chem. Soc..

[cit3] Jun H., Angelici R. J. (1994). Organometallics.

[cit4] Finz M., Bernhardt E., Willner H., Lehmann C. W. (2004). Angew. Chem., Int. Ed..

[cit5] Konze W. V., Young V. G., Angelici R. J. (1999). Organometallics.

[cit6] Cordaro J. G., Stein D., Rüegger H., Grützmacher H. (2006). Angew. Chem., Int. Ed..

[cit7] Trathen N., Leech M. C., Crossley I. R., Greenacre V. K., Roe S. M. (2014). Dalton Trans..

[cit8] Leech M. C., Crossley I. R. (2018). Dalton Trans..

[cit9] Levis M. C., Pearce K. G., Crossley I. R. (2019). Inorg. Chem..

[cit10] Levis M. C., Helm M. L., Turner J. F. C., Crossley I. R. (2024). Chem.–Eur. J..

[cit11] Hoerger C. J., Heinemann F. W., Louyriac E., Maron L., Grützmacher H., Meyer K. (2017). Organometallics.

[cit12] Goicoechea J. M., Grützmacher H. (2018). Angew. Chem., Int. Ed..

[cit13] Heift D., Benkő Z., Grützmacher H. (2014). Dalton Trans..

[cit14] Green S. P., Jones C., Stasch A. (2007). Science.

[cit15] Wilson D. W. N., Urwin S. J., Yang E. S., Goicoechea J. M. (2021). J. Am. Chem. Soc..

[cit16] Yang E. S., Goicoechea J. M. (2022). Angew. Chem., Int. Ed..

[cit17] Yang E. S., Wilson D. W. N., Goicoechea J. M. (2023). Angew. Chem., Int. Ed..

[cit18] Yang E. S., Combey E., Goicoechea J. M. (2023). Chem. Sci..

[cit19] Yang E. S., Mapp A., Taylor A., Beer P. D., Goicoechea J. M. (2023). Chem.–Eur. J..

[cit20] Mapp A., Wilmore J., Beer P. D., Goicoechea J. M. (2023). Angew. Chem., Int. Ed..

[cit21] Yang E. S., García-Romero A., Hu C., Fletcher J., Thomas C. M., Goicoechea J. M. (2024). J. Am. Chem. Soc..

[cit22] Wannipurage D. C., Yang E. S., Chivington A. D., Fletcher J., Ray D., Yamamoto N., Pink M., Goicoechea J. M., Smith J. M. (2024). J. Am. Chem. Soc..

[cit23] Yang E. S., Goicoechea J. M. (2025). Chem. Commun..

[cit24] Görlich T., Frost D. S., Boback N., Coles N. T., Dittrich B., Müller P., Jones W. D., Müller C. (2021). J. Am. Chem. Soc..

[cit25] Cui C., Roesky H. W., Schmidt H.-G., Noltemeyer M., Hao H., Cimpoesu F. (2000). Angew. Chem., Int. Ed..

[cit26] Zhong M., Sinhababu S., Roesky H. W. (2020). Dalton Trans..

[cit27] Liu L. L., Zhou J., Cao L. L., Stephan D. W. (2019). J. Am. Chem. Soc..

[cit28] Pyykkö P., Atsumi M. (2009). Chem.–Eur. J..

[cit29] Pyykkö P., Reidel S., Patzscke M. (2005). Chem.–Eur. J..

[cit30] Bohle D. S., Rickard C. E. F., Roper W. R. (1985). J. Chem. Soc., Chem. Commun..

[cit31] Bohle D. S., Clark G. R., Rickard C. E. F., Roper W. R. (1988). J. Organomet. Chem..

[cit32] Figueroa J. S., Cummins C. C. (2004). J. Am. Chem. Soc..

[cit33] Breit N. C., Szilvási T., Inoue S. (2014). Chem.–Eur. J..

[cit34] García-Rodeja Y., Bickelhaupt F. M., Fernández I. (2016). Chem.–Eur. J..

[cit35] Organometallics in Synthesis, ed. M. Schlosser, John Wiley & Sons, Inc., Hoboken, NJ, USA, 2013

[cit36] Wagner O., Ehle M., Regitz M. (1989). Angew. Chem., Int. Ed..

[cit37] Schäfer A., Weidenbruch M., Saak W., Pohl S. (1987). Angew. Chem., Int. Ed..

[cit38] Cowley A. H., Hall S. W., Nunn C. M., Power J. M. (1988). J. Chem. Soc., Chem. Commun..

[cit39] Mansell S. M., Green M., Russell C. A. (2012). Dalton Trans..

[cit40] Hitchcock P. B., Maah M. J., Nixon J. F. (1986). J. Chem. Soc., Chem. Commun..

[cit41] Rösch W., Facklam T., Regitz M. (1987). Tetrahedron.

[cit42] Mack A., Pierron E., Allspach T., Bergsträßer U., Regitz M. (1998). Synthesis.

[cit43] Herbstein F. H., Kapon M., Reisner G. M. (1986). Acta Crystallogr. B.

[cit44] González-Pinardo D., Goicoechea J. M., Fernández I. (2024). Chem.–Eur. J..

[cit45] González-Pinardo D., Fernández I. (2025). Inorg. Chem..

[cit46] Schmidbaur H., Schier A. (2012). Chem. Soc. Rev..

[cit47] Coburger P., Hadlington T. J. (2025). Z. Anorg. Allg. Chem..

[cit48] (a) CCDC 2531460: Experimental Crystal Structure Determination, 2026, 10.5517/ccdc.csd.cc2qz60y

