# Supplementary material for: Cyaphide generation at an aluminium(i) center: a useful precursor for phosphorus-containing heterocycles
Source: Chem Sci. 2026 May 5;17(24):12101–10. doi: 10.1039/d6sc03023h (PMC13170228; doi:10.1039/d6sc03023h)
Supplement: SC-017-D6SC03023H-s001 [file SC-017-D6SC03023H-s001.pdf]

Supplementary Information (SI) for Chemical Science.
This journal is © The Royal Society of Chemistry 2026

Contents

1. Experimental procedures 2

1.1. Materials and methods 2

1.2. Synthesis, spectroscopic and analytical data 3

2. NMR and IR spectra 16

3. High-resolution mass spectra 53

4. Single crystal X-ray data 56

5. Computational Details 79

6. References 121

1. Experimental procedures

1.1. Materials and methods

All experiments were carried out under an inert atmosphere of argon using standard Schlenk line techniques or under nitrogen using glovebox techniques (MBraun UNIlab glovebox maintained at < 0.1 ppm H_2O and < 0.1 ppm O_2). Glassware was dried at 80 °C prior to use. Benzene, toluene, dioxane, THF, diethyl ether and hexane were purified using a Pure Process Technology (PPT) solvent purification system (SPS). C_6D_6 and d_8-toluene were distilled over sodium/benzophenone. All dry solvents were stored under argon in gas-tight ampoules over 3 Å activated molecular sieves. $^{i}Pr_3SiOTf$ was dried over sieves. Silica was kept for three days at 80 °C and dried under dynamic vacuum prior to use. $AlCl_3$ was sublimed prior to use. All other chemicals were used as purchased. The synthesis of the following precursors was done according to previously reported procedures: $[Na(dioxane)_2]PCO$,[1] (^{Dipp}NacNac)H,[2] Na(^{Dipp}NacNac)[3] and KCp*.[4]

NMR spectra were acquired on a Bruker 500 MHz Avance Neo or a Varian 500 MHz Inova NMR spectrometer. Chemical shifts (δ) are reported in parts per million (ppm). ^{1}H and ^{13}C NMR spectra are referenced to TMS using residual protio-solvent resonance (^{1}H NMR C_6D_6: δ = 7.16 ppm, ^{13}C NMR C_6D_6: δ = 128.06 ppm; ^{1}H NMR d_8-toluene: δ = 2.08 ppm). ^{29}Si NMR spectra were externally referenced to TMS. ^{31}P NMR spectra were externally referenced to an 85% solution of H_3PO_4 in H_2O. High-resolution mass spectra were recorded on a Thermo Q-Exactive Plus (APCI-Orbitrap, positive ion mode) instrument at the Mass Spectrometry Facility of the Department of Chemistry of Indiana University. IR spectra were acquired on a Thermo Scientific Nicolet Summit FTIR spectrometer with a diamond ATR stage. X-ray diffraction data were collected with a Bruker

D8 Venture diffractometer equipped with a PhotonII detector and IμS sources, using Mo K_α and Cu K_α irradiation sources.

1.2. Synthesis, spectroscopic and analytical data

Synthesis of [AlCp*]₄. The following procedure is a modified protocol originally published by Fischer and coworkers.[5] Diethyl ether (100 mL) was added to $AlCl_3$ (2.868 g, 21.4 mmol) while stirring. A 1M solution of $LiAlH_4$ in Et_2O (7.2 mL, 7.2 mmol) was added dropwise, and the solution was stirred for 1 hour at room temperature. The resulting $AlCl_2H$ solution was filtered using a cannula onto KCp^* (10 g, 57.4 mmol). The mixture was stirred at room temperature for 4 hours and filtered by cannula again. The filtrate was concentrated under dynamic vacuum to give a yellowish solid of $Cp^*{}_2AlH$. The solid was heated at 110 °C under argon for 30 minutes and subsequently heated at the same temperature for additional 30 minutes under the dynamic vacuum, affording a pale yellow powder of $[AlCp^*]_4$. The powder was washed with hexane (3 × 5 mL) and dried under dynamic vacuum. Yield: 84% (2.92 g, 18 mmol).

^{1}H NMR (C_6D_6, 500 MHz, 25 °C): δ (ppm) 1.90 (s, 15H, $C_5(CH_3)_5$). The characterization data is in agreement with previously reported work.[5]

Synthesis of Al(^{Dipp}NacNac). The following procedure is a modified protocol originally published by Kretschmer and coworkers.[3] Toluene (70 mL) was added to the mixture of $[AlCp^*]_4$ (1 g, 1.54 mmol) and (^{Dipp}NacNac)Na (3.26 g, 7.39 mmol). The resulting suspension was stirred at 95 °C for 9.5 hours. The precipitate, $NaCp^*$, was removed by filtration with a cannula, and the dark-red filtrate was kept at room temperature overnight. The solution was concentrated to approximately 20% of its original volume and stored at 3 °C overnight, resulting in crystallization of a red solid.

The crystals were separated by filtration using a cannula and dried under dynamic vacuum. Toluene (20 mL) was added to the solid and the resulting mixture was stirred for about 10 minutes until red crystals dissolved. The formed suspension was filtered to separate the yellow residue and yield a transparent dark-red solution. The solution was kept at –35 °C overnight to yield dark red crystals. The solution was removed by decantation, and the crystals were dried under dynamic vacuum to yield pure Al(^{Dipp}NacNac). Yield: 22% (598 mg, 1.35 mmol).

^{1}H NMR (C_6D_6, 500 MHz, 25 °C): δ (ppm) 7.22 – 7.15 (m, overlaps with C_6D_5H, prevents integration; Dipp ArH), 5.18 (s, 1H, NacNac γ-H), 3.17 (sept, 4H, $^{3}J_{H-H}$ = 6.9 Hz, Dipp CH(CH_3)$_2$), 1.66 (s, 6H, NacNac NCCH_3), 1.38 (d, 12H, $^{3}J_{H-H}$ = 6.9 Hz, Dipp CH(CH_3)$_2$), 1.13 (d, 12H, $^{3}J_{H-H}$ = 6.9 Hz, Dipp CH(CH_3)$_2$). The characterization data is in agreement with previously reported work.[3]

Method 1. Synthesis and isolation of [Al(^{Dipp}NacNac)(OSi^{i}Pr$_3$)(CP)] (1), [Al(^{Dipp}NacNac)(PC(Si^{i}Pr$_3$)O)] (2) and [Al(^{Dipp}NacNac)(PCOSi^{i}Pr$_3$)] (3). A solution of ^{i}Pr$_3$SiOTf (332 mg, 1.08 mmol) in hexane (10 mL) was added to [Na(dioxane)$_2$]PCO (340 mg, 1.30 mmol). The resulting yellowish suspension was stirred overnight to form iPr$_3$SiOCP. The precipitate was removed by filtration, and the filtrate was transferred directly onto crystals of Al(^{Dipp}NacNac) (385 mg, 0.87 mmol). The resulting dark-brown solution was stirred for 30 minutes. The solution was concentrated to approximately 8 mL under dynamic vacuum and stored at −35°C overnight affording beige crystals of the pure **1** (199 mg, 0.30 mmol, 35% yield). The supernatant after crystallization of **1** was concentrated to approximately 50% of its volume and stored at −35°C overnight. The resulting mixture of crystals was separated from supernatant and dried under dynamic vacuum. The bright red crystals of **2** were handpicked from the mixture, rinsed with 0.5

mL of hexane and dried under dynamic vacuum. The resulting crystals were used for the analysis without further purification (11.2 mg, 0.017 mmol, 2% yield). The supernatant after the second crystallization was used without further purification for the characterization of species **3**.

Method 2. Synthesis and isolation of [Al(^{Dipp}NacNac)(OSi^{i}Pr$_3$)(CP)] (1). A solution of ^{i}Pr$_3$SiOTf (196 mg, 0.64 mmol) in toluene (3 mL) was added to [Na(dioxane)$_2$]PCO (196 mg, 0.75 mmol). The resulting yellowish suspension was stirred overnight to form ^{i}Pr$_3$SiOCP. The precipitate was removed by filtration, and the filtrate was transferred into one of the legs of λ-cell, while crystals of Al(^{Dipp}NacNac) (190 mg, 0.43 mmol) were placed to the other one. The λ-cell contents were cooled to −100 °C in an acetone bath, mixed and stirred for 3 hours, allowing the reaction mixture and the acetone cooling bath to naturally warm-up. The solution was put through a layer of silica (4.5 × 0.7 × 0.7 cm); the silica was washed with toluene (5 × 1 mL). The combined solution in toluene was evaporated under dynamic vacuum. The formed residue was redissolved in hexane, and the resulting solution was kept at −35 °C overnight affording the first crop of beige crystals of the pure product (98 mg, 0.15 mmol). The supernatant was concentrated to approximately 50% of its original volume and the solution was stored at −35 °C overnight to yield a second crop of crystals (20.5 mg, 0.03 mmol). Yield: 42% (combined, 118.5 mg, 0.18 mmol).

[Al(^{Dipp}NacNac)(OSi^{i}Pr$_3$)(CP)] (1) – spectroscopic data

^{1}H NMR (500 MHz, C_6D_6, 25 °C): δ (ppm) 7.21 – 7.13 (m, overlaps with C_6D_5H, prevents integration; Dipp Ar*H*), 7.10 (dd, $^3J_{H-H}$ = 7.4, 1.9 Hz, 2H; Dipp Ar*H*), 4.99 (s, 1H; NacNac γ-*H*), 4.07 (sept, $^3J_{H-H}$ = 6.7 Hz, 2H; Dipp C*H*(CH$_3$)$_2$), 3.23 (sept, $^3J_{H-H}$ = 6.9 Hz, 2H; Dipp C*H*(CH$_3$)$_2$), 1.57 (d, $^3J_{H-H}$ = 6.6 Hz, 6H; Dipp CH(C*H*$_3$)$_2$), 1.51 (s, 6H; NacNac NCC*H*$_3$), 1.37 (d, $^3J_{H-H}$ = 6.8

Hz, 6H; Dipp CH(CH_3)$_2$), 1.28 (d, $^3J_{H–H}$ = 6.8 Hz, 6H; Dipp CH(CH_3)$_2$), 1.02 (d, $^3J_{H–H}$ = 6.8 Hz, 6H; Dipp CH(CH_3)$_2$), 0.87 (m, 21H; overlapping Si[CH(CH_3)$_2$]$_3$ and Si[CH(CH$_3$)$_2$]$_3$).

^{13}C{^{1}H} NMR (126 MHz, C_6D_6, 25 °C): δ (ppm) 230.96 (br s., *C*P), 171.63 (NacNac N*C*CH$_3$), 145.47 (Dipp *ortho*-*C*^{i}Pr), 143.24 (Dipp *ortho*-*C*^{i}Pr), 141.13 (Dipp *ipso*-*C*), 127.53 (Dipp Ar*C*H), 125.35 (Dipp Ar*C*H), 124.25 (Dipp Ar*C*H), 98.90 (NacNac γ-*C*H), 29.06 (Dipp *C*H(CH$_3$)$_2$), 28.21 (Dipp *C*H(CH$_3$)$_2$), 28.05 (Dipp CH(*C*H$_3$)$_2$), 24.72 (Dipp CH(*C*H$_3$)$_2$), 24.40 (Dipp CH(*C*H$_3$)$_2$), 24.30 (Dipp CH(*C*H$_3$)$_2$), 23.93 (NacNac NC*C*H$_3$), 18.58* (Si[CH(*C*H$_3$)$_2$]$_3$), 14.45* (Si[*C*H(CH$_3$)$_2$]$_3$). *Assignment based on the pattern observed for the other compounds

^{31}P{^{1}H} NMR (202 MHz, C_6D_6, 25 °C): δ (ppm) 128.31.

^{29}Si{^{1}H} NMR (99 MHz, C_6D_6, 25 °C): δ (ppm) 2.13.

HRMS (APCI): m/z [M+H]$^+$ calcd for $C_{39}H_{63}AlN_2OPSi^+$: 661.4257; found: 661.4276.

IR: 1372 cm^{-1}

[Al(^{Dipp}NacNac)(PC(Si^{i}Pr$_3$)O)] (2) – spectroscopic data

^{1}H NMR (500 MHz, C_6D_6, 25 °C): δ (ppm) 7.21 – 7.04 (m, overlaps with C$_6$D$_5$*H*, prevents integration; Dipp Ar*H*), 4.99 (s, 1H; NacNac γ-*H*), 3.33 (sept, $^3J_{H–H}$ = 6.7 Hz, 2H; Dipp C*H*(CH$_3$)$_2$), 3.18 (sept, $^3J_{H–H}$ = 6.8 Hz, 2H; Dipp C*H*(CH$_3$)$_2$), 1.57 (s, 6H; NacNac NCCH_3), 1.49−1.50 (two overlapping d, $^3J_{H–H}$ = 6.70, 6.73 Hz, 12H; Dipp CH(CH_3)$_2$), 1.23 (sept, $^3J_{H–H}$ = 7.4 Hz, 3H; Si[C*H*(CH$_3$)$_2$]$_3$), 1.13 (d, $^3J_{H–H}$ = 6.9 Hz, 6H; Dipp CH(CH_3)$_2$), 1.03−0.98 (two overlapping d, $^3J_{H–H}$ = 6.7 Hz, 7.5 Hz, 24H; Dipp CH(CH_3)$_2$ and Si[CH(CH_3)$_2$]$_3$).

^{13}C{^{1}H} NMR (126 MHz, C_6D_6, 25 °C): δ (ppm) 255.66 (d, $^1J_{C–P}$ = 105.48 Hz; P*C*(Si^{i}Pr$_3$)O), 171.38 (NacNac N*C*CH$_3$), 144.78 (Dipp *ortho*-*C*^{i}Pr), 144.27 (Dipp *ortho*-*C*^{i}Pr), 138.07 (Dipp *ipso*-*C*), 128.35 (Dipp Ar*C*H), 124.92 (Dipp Ar*C*H), 124.45 (Dipp Ar*C*H), 98.29 (NacNac

γ-*C*H), 29.05 (Dipp *C*H(CH_3)$_2$), 29.00 (Dipp *C*H(CH_3)$_2$), 25.97 or 25.91 (Dipp CH($\mathit{C}H_3$)$_2$), 25.26 (Dipp CH($\mathit{C}H_3$)$_2$), 24.95 (Dipp CH($\mathit{C}H_3$)$_2$), 24.59 (Dipp C($\mathit{C}H_3$)$_2$), 23.24 (NacNac NC$\mathit{C}H_3$), 19.13 (Si[CH($\mathit{C}H_3$)$_2$]$_3$), 11.90 (Si[*C*H(CH_3)$_2$]$_3$).

^{31}P{^{1}H} NMR (202 MHz, C_6D_6, 25 °C): δ (ppm) 164.44.

^{29}Si{^{1}H} NMR (99 MHz, C_6D_6, 25 °C): δ (ppm) −2.04 (d, $^2J_{\text{Si–P}}$ = 20.7 Hz).

[Al(^{Dipp}NacNac)(PCOSi^{i}Pr$_3$)] (3) – spectroscopic data

^{1}H NMR (500 MHz, C_6D_6, 25 °C): δ (ppm) 7.17 – 7.01 (m, 6H; Dipp Ar*H*), 4.89 (s, 1H; NacNac γ-*H*), 3.54 (sept, $^3J_{\text{H–H}}$ = 6.4 Hz, 2H; Dipp C*H*(CH_3)$_2$), 3.08 (sept, $^3J_{\text{H–H}}$ = 6.3 Hz, 2H; Dipp C*H*(CH_3)$_2$), 1.58 – 1.47 (m, 21H; overlapping signals of NacNac NCCH_3, Si[C*H*(CH_3)$_2$]$_3$ and two Dipp CH(CH_3)$_2$), 1.13 (d, $^3J_{\text{H–H}}$ = 6.7 Hz, 6H; Dipp CH(CH_3)$_2$), 1.08 (d, $^3J_{\text{H–H}}$ = 7.3 Hz, 6H; Dipp CH(CH_3)$_2$), 1.05 (d, $^3J_{\text{H–H}}$ = 7.5 Hz, 18H; Si[CH(CH_3)$_2$]$_3$).

^{13}C{^{1}H} NMR (126 MHz, C_6D_6, 25 °C): δ (ppm) 286.86 (d, $^1J_{\text{C–P}}$ = 120.45 Hz; P*C*(OSi^{i}Pr$_3$)), 172.62 (NacNac N*C*CH$_3$), 144.30 (Dipp *ortho*-*C*^{i}Pr), 143.51 (Dipp *ortho*-*C*^{i}Pr), 138.66 (Dipp *ipso*-*C*), 127.53 (Dipp Ar*C*H), 124.72 (Dipp Ar*C*H), 124.45 (Dipp Ar*C*H), 96.71 (NacNac γ-*C*H), 29.14 (Dipp *C*H(CH_3)$_2$), 25.05 (Dipp CH($\mathit{C}H_3$)$_2$), 25.00 (Dipp CH($\mathit{C}H_3$)$_2$), 24.54 (Dipp CH($\mathit{C}H_3$)$_2$), 23.49 (NacNac NC$\mathit{C}H_3$), 18.65 (Si[CH($\mathit{C}H_3$)$_2$]$_3$), 13.00 (Si[*C*H(CH_3)$_2$]$_3$).

^{31}P{^{1}H} NMR (202 MHz, C_6D_6, 25 °C): δ (ppm) 344.89.

^{29}Si{^{1}H} NMR (99 MHz, C_6D_6, 25 °C): δ (ppm) 16.66.

[Al(^{Dipp}NacNac)(OSi^{i}Pr$_3$)(CPNi{COD})] (4). The compound was generated *in situ* by adding a solution of **1** (18.1 mg, 0.027 mmol) in C_6D_6 (circa. 0.5 mL) to Ni(COD)$_2$ (8 mg, 0.029 mmol). No further purification was performed. Quantitative conversion to product **4** was observed by ^{1}H

and $^{31}P\{^{1}H\}$ NMR spectroscopy. Compound **4** slowly decomposes over the course of several days. Despite multiple attempts, isolation of **4** as a compositionally pure solid was not possible.

^{1}H NMR (500 MHz, C_6D_6, 25 °C): *δ* (ppm) 7.16 – 7.11 (m, overlaps with C_6D_5*H*, prevents integration; Dipp Ar*H*), 5.79 – 5.64 (m, 3H; Ni-bound COD C*H*), 5.58 (s, 4H; 'free' COD C*H*), 5.00 (s, 1H; NacNac γ-*H*), 4.30 (s, 1H; Ni-bound COD C*H*), 3.42 (sept, 2H, $^{3}J_{H–H}$ = 6.7 Hz; Dipp C*H*(CH_3)$_2$), 3.16 (sept, 2H, $^{3}J_{H–H}$ = 6.6 Hz; Dipp C*H*(CH_3)$_2$), 2.21 (s, 8H; 'free' COD CH_2), 2.13 – 1.94 (m, 8H; Ni-bound COD CH_2), 1.56 (s, 6H; NacNac NCCH_3), 1.47 (d, 6H, $^{3}J_{H–H}$ = 6.9 Hz; Dipp CH(CH_3)$_2$), 1.24 – 1.15 (m, 9H, Si[C*H*(CH_3)$_2$]$_3$) overlapping with Dipp CH(CH_3)$_2$), 1.09 (d, 12H, $^{3}J_{H–H}$ = 6.7 Hz; Dipp CH(CH_3)$_2$), 0.93 (d, 18H, $^{3}J_{H–H}$ = 7.5 Hz; Si[CH(CH_3)$_2$]$_3$).

$^{13}C\{^{1}H\}$ NMR (126 MHz, C_6D_6, 25 °C): *δ* (ppm) 257.58 (br. s., Al–*C*P), 169.87 (NacNac N*C*CH$_3$), 145.15 (Dipp *ortho*-*C*^{i}Pr), 143.52 (Dipp *ortho*-*C*^{i}Pr), 141.85 (Dipp *ipso*-*C*), 128.83 ('free' COD *C*H), 127.30 (Dipp Ar*C*H), 125.43 (Dipp Ar*C*H), 124.37 (Dipp Ar*C*H), 98.18 (Ni-bound COD *C*H), 98.11 (NacNac γ-*C*H), 93.30 (Ni-bound COD *C*H), 89.72 (Ni-bound COD *C*H), 30.88 (Ni-bound COD *C*H$_2$), 30.20 (Ni-bound COD *C*H$_2$), 29.11 (Dipp *C*H(CH_3)$_2$), 28.40 ('free' COD *C*H$_2$), 27.09 (Dipp CH(*C*H$_3$)$_2$), 26.32 (Dipp *C*H(CH_3)$_2$), 25.49 (Dipp CH(*C*H$_3$)$_2$), 25.01 (Dipp CH(*C*H$_3$)$_2$), 24.55 (Dipp CH(*C*H$_3$)$_2$), 24.18 (NacNac NC*C*H$_3$), 19.31 (Si[CH(*C*H$_3$)$_2$]$_3$), 15.43 (Si[*C*H(CH_3)$_2$]$_3$).

$^{31}P\{^{1}H\}$ NMR (202 MHz, C_6D_6, 25 °C): *δ* (ppm) 315.53.

$^{29}Si\{^{1}H\}$ NMR (99 MHz, C_6D_6, 25 °C): *δ* (ppm) 0.97.

[Al(^{Dipp}NacNac)(OSi^{i}Pr$_3$)(CPN$_3$Bn)] (5a). A solution of **1** (60 mg, 0.091 mmol) in toluene (circa 1 mL) was placed into an NMR tube equipped with a J. Young gas-tight valve. A 0.5 M solution of BnN_3 in CH_2Cl_2 (0.22 mL, 0.110 mmol) was added to the NMR tube via syringe. The resulting

solution was carefully shaken for 1 minute. The completion of reaction was determined by $^{31}P\{^1H\}$ NMR spectroscopy. All volatiles were removed under a dynamic vacuum. The remaining white solid was dissolved in hexane (circa 10 mL) and the resulting solution was stored at −35 °C overnight to crystallize the first crop of the product (53.5 mg, 0.067 mmol). The mother liquor was concentrated to approximately 3 mL and stored at −35 °C overnight to yield the second crop of the product (10.2 mg, 0.013 mmol). Yield: 88% (combined, 63.7 mg, 0.080 mmol).

^{1}H NMR (500 MHz, C_6D_6, 25 °C): δ (ppm) 7.20 (dd, $^3J_{H-H}$ = 6.9, $^4J_{H-H}$ = 1.5 Hz, 2H; Dipp Ar*H*), 7.15 – 6.98 (m, 9H, overlapping Dipp Ar*H* and $CH_2C_6H_5$), 5.49 (d, $^3J_{H-P}$ = 5.1 Hz, 2H; C*H*$_2C_6H_5$), 5.20 (s, 1H; NacNac γ-*H*), 3.47 (sept, $^3J_{H-H}$ = 6.8 Hz, 2H; Dipp C*H*$(CH_3)_2$), 2.92 (sept, $^3J_{H-H}$ = 6.7 Hz, 2H; Dipp C*H*$(CH_3)_2$), 1.54 (s, 6H; NacNac NCC*H*$_3$), 1.46 (d, $^3J_{H-H}$ = 6.8 Hz, 6H; Dipp CH(C*H*$_3$)$_2$), 1.12 (sept, $^3J_{H-H}$ = 7.7 Hz, 3H; Si[C*H*$(CH_3)_2$]$_3$) overlapping with 1.08 (d, $^3J_{H-H}$ = 6.7 Hz, 6H; Dipp CH(C*H*$_3$)$_2$), 0.94 (d, $^3J_{H-H}$ = 7.5 Hz, 18H; Si[CH(C*H*$_3$)$_2$]$_3$), 0.90 (d, $^3J_{H-H}$ = 6.8 Hz, 6H; Dipp CH(C*H*$_3$)$_2$), 0.72 (d, $^3J_{H-H}$ = 6.7 Hz, 6H; Dipp CH(C*H*$_3$)$_2$).

$^{13}C\{^1H\}$ NMR (126 MHz, C_6D_6, 25 °C): δ (ppm) 192.70 (br. s., Al–*C*P), 171.78 (NacNac N*C*CH$_3$), 145.20 (Dipp *ortho*-*C*^{i}Pr), 143.34 (Dipp *ortho*-*C*^{i}Pr), 141.55 (Dipp *ipso*-*C*), 139.03 (Ph quat. *C*), 128.81 (Dipp or Ph Ar*C*H), 128.49 (Dipp Ar*C*H), 128.35 (Dipp or Ph Ar*C*H), 127.38 (Dipp or Ph Ar*C*H), 125.15 (Dipp or Ph Ar*C*H), 124.24 (Dipp or Ph Ar*C*H), 99.90 (d; NacNac γ-*C*H), 54.77 (d, $^2J_{C-P}$ = 11.4 Hz; *C*H$_2C_6H_5$), 29.26 (Dipp *C*H$(CH_3)_2$), 27.92 (Dipp *C*H$(CH_3)_2$), 25.21 (Dipp CH(*C*H$_3$)$_2$), 24.91 (Dipp CH(*C*H$_3$)$_2$), 24.23 (br.s., two merged Dipp CH(*C*H$_3$)$_2$ groups), 24.16 (NacNac NC*C*H$_3$), 18.97 (Si[CH(*C*H$_3$)$_2$]$_3$), 15.17 (Si[*C*H$(CH_3)_2$]$_3$).$^{31}P\{^1H\}$ NMR (202 MHz, C_6D_6, 25°C): δ (ppm) 221.60.

$^{29}Si\{^1H\}$ NMR (99 MHz, C_6D_6, 25 °C): δ (ppm) 1.54.

HRMS (APCI): m/z $[M+H]^+$ calcd for $C_{46}H_{70}AlN_5OPSi^+$: 794.4897; found: 794.4886.

[Al(^{Dipp}NacNac)(OSi^{i}Pr$_3$)(CPN$_3$SiMe$_3$)] (5b). A solution of **1** (15.9 mg, 0.024 mmol) in C_6D_6 (circa 0.5 mL) was placed into an NMR tube equipped with a J. Young gas-tight valve. A 0.048 M solution of $TMSN_3$ in toluene (0.6 mL, 0.029 mmol) was added to the NMR tube using a syringe. The resulting solution was carefully shaken and kept at room temperature for three days. The reaction completion was monitored by $^{31}P\{^1H\}$ NMR spectroscopy. All volatiles were removed under a dynamic vacuum. The remaining white solid was dissolved in hexane (circa 2 mL) and the resulting solution was stored at −35° C over three days to crystallize the first crop of the product (8.7 mg, 0.011 mmol). The mother liquor was concentrated to approximately 1 mL and stored at −35 ° C for over three days to yield the second crop of the product (7.5 mg, 0.010 mmol). Yield: 87% (combined, 16.2 mg, 0.021 mmol)

^{1}H NMR (500 MHz, C_6D_6, 25 °C): δ (ppm) 7.17 – 7.09 (m, overlaps with C_6D_5H, prevents integration; Dipp Ar*H*), 7.02 (dd, $^3J_{H-H}$ = 6.8, $^4J_{H-H}$ = 2.5 Hz, 2H; Dipp Ar*H*), 5.26 (s, 1H; NacNac γ-*H*), 3.53 (sept, $^3J_{H-H}$ = 6.7 Hz, 2H; Dipp C*H*(CH$_3$)$_2$), 2.92 (sept, $^3J_{H-H}$ = 6.6 Hz, 2H; Dipp C*H*(CH$_3$)$_2$), 1.59 (s, 6H; NacNac NCC*H*$_3$), 1.48 (d, $^3J_{H-H}$ = 6.7 Hz, 6H; Dipp CH(C*H*$_3$)$_2$), 1.17 (sept, $^3J_{H-H}$ = 7.3 Hz, 3H; Si[C*H*(CH$_3$)$_2$]$_3$), 1.10 (d, $^3J_{H-H}$ = 6.9 Hz, 6H; Dipp CH(C*H*$_3$)$_2$), 0.96 (d, $^3J_{H-H}$ = 7.5 Hz, 18H; Si[CH(C*H*$_3$)$_2$]$_3$), 0.92 (d, $^3J_{H-H}$ = 6.7 Hz, 6H; Dipp CH(C*H*$_3$)$_2$), 0.71 (d, $^3J_{H-H}$ = 6.7 Hz, 6H; Dipp CH(C*H*$_3$)$_2$), 0.48 (s, 9H; Si(C*H*$_3$)$_3$).

$^{13}C\{^1H\}$ NMR (126 MHz, C_6D_6, 25 °C): δ (ppm) 189.80 (br. s., Al–*C*P), 171.72 (NacNac N*C*CH$_3$), 145.10 (Dipp *ortho*-*C*^{i}Pr), 143.40 (Dipp *ortho*-*C*^{i}Pr), 141.63 (Dipp *ipso*-*C*), 127.38 (Dipp Ar*C*H), 125.17 (Dipp Ar*C*H), 124.25 (Dipp Ar*C*H), 99.90 (NacNac γ-*C*H), 29.30 (Dipp *C*H(CH$_3$)$_2$), 27.89 (Dipp *C*H(CH$_3$)$_2$), 25.10 (Dipp CH(*C*H$_3$)$_2$), 24.90 (Dipp CH(*C*H$_3$)$_2$), 24.39 (Dipp CH(*C*H$_3$)$_2$),

24.21 (Dipp CH(CH_3)$_2$ and NacNac NCCH_3), 19.04 Si[CH(CH_3)$_2$]$_3$), 15.23 Si[CH(CH_3)$_2$]$_3$), 0.88 (Si(CH_3)$_3$).

$^{31}P\{^1H\}$ NMR (202 MHz, C_6D_6, 25 °C): δ (ppm) 238.19.

$^{29}Si\{^1H\}$ NMR (99 MHz, C_6D_6, 25 °C): δ (ppm) 1.60 ($^iPr_3SiO$). The resonance corresponding to the CPN_3SiMe_3 fragment was not observed possibly due to the coupling to the quadrupolar ^{14}N nucleus ($S = 1$).

HRMS (APCI): m/z $[M+H]^+$ calcd for $C_{42}H_{72}AlN_5OPSi_2^+$: 776.4823; found: 776.4814.

[Al(^{Dipp}NacNac)(OSi^{i}Pr$_3$)(C$_7$PH$_{10}$)] (6). A solution of **1** (15.9 mg, 0.024 mmol) in C_6D_6 (circa 0.5 mL) was placed into an NMR tube equipped with a J. Young gas-tight valve. 2,3-dimethyl-1,3-butadiene (0.05 mL, 0.440 mmol) was added to the NMR tube using a syringe. The resulting solution was carefully shaken and heated at 80 °C for 1 day. The reaction completion was determined by ^{31}P NMR spectroscopy. All volatiles were removed under a dynamic vacuum. The remaining white solid was dissolved in hexane (circa 2 mL) and the resulting solution was stored at −35 °C overnight to crystallize the first crop of the product (10 mg, 0.0135 mmol). The mother liquor was concentrated to approximately 0.5 mL and stored at −35 °C for over three days to yield the second crop of the product (5.4 mg, 0.0072 mmol). Yield: 86% (combined, 15.4 mg, 0.0207 mol)

^1H NMR* (500 MHz, C_6D_6, 25 °C): δ (ppm) 7.20 – 7.09 (m, overlaps with C_6D_5H, prevents integration; Dipp ArH), 4.95 (s, 1H; NacNac γ-H), 3.71 (d, 2H, $^3J_{P–H}$ = 17.1 Hz; CH_2– (Al)=P–CH$_2$), 3.38 (sept, 2H, $^3J_{H–H}$ = 6.8 Hz; Dipp CH(CH_3)$_2$), 3.01 (sept, 2H, $^3J_{H–H}$ =7.0 Hz; Dipp CH(CH_3)$_2$), 2.36 (s, 2H; CH$_2$–C(Al)=P–CH_2), 1.89 (s, 3H; heterocyclic CH-CH_3), 1.77 (s, 3H; heterocyclic CH-CH_3), 1.55 (s, 6H; NacNac NCCH_3), 1.45 (d, 6H, $^3J_{H–H}$ = 6.7 Hz; Dipp

CH(CH_3)$_2$), 1.21 (d, 6H, $^3J_{H–H}$ = 6.6 Hz; Dipp CH(CH_3)$_2$), 1.19 – 1.14 (m, 3H; Si[CH(CH$_3$)$_2$]$_3$), 1.12 (d, 6H, $^3J_{H–H}$ = 6.7 Hz; Dipp CH(CH_3)$_2$), 1.07 (d, 6H, $^3J_{H–H}$ = 6.7 Hz; Dipp CH(CH_3)$_2$), 0.92 (d, 18H, $^3J_{H–H}$ = 7.5 Hz; Si[CH(CH_3)$_2$]$_3$). *Only the signals of major conformer are listed.

^{13}C{^{1}H} NMR* (126 MHz, C_6D_6, 25 °C): δ (ppm) 211.74 (br. s., Al–CP), 171.84 (NacNac NCCH$_3$), 145.00 (Dipp *ortho*-C^iPr), 143.37 (Dipp *ortho*-C^iPr), 141.81 (Dipp *ipso*-C), 127.36 (Dipp ArCH), 126.32 (d, $^2J_{C\text{-}P}$ = 12.4 Hz; heterocyclic quat. C=C), 125.39 (Dipp ArCH), 124.40 (Dipp ArCH), 123.56 (heterocyclic quat. C=C), 99.21 (NacNac γ-CH), 45.26 (d, $^2J_{C\text{-}P}$ = 11.4 Hz; CH$_2$–C(Al)=P–CH$_2$), 37.95 (d, $^1J_{C\text{-}P}$ = 49.1 Hz; CH$_2$–C(Al)=P–CH$_2$), 29.20 (Dipp CH(CH$_3$)$_2$), 27.04 (Dipp CH(CH$_3$)$_2$), 26.16 (Dipp CH(CH$_3$)$_2$), 25.62 (Dipp CH(CH$_3$)$_2$), 25.13 (Dipp CH(CH$_3$)$_2$), 24.32 (Dipp CH(CH$_3$)$_2$), 24.29 (NacNac NCCH$_3$), 21.13 (heterocyclic CH$_3$), 20.70 (heterocyclic CH$_3$), 19.36** (Si[CH(CH$_3$)$_2$]$_3$), 15.75** (Si[CH(CH$_3$)$_2$]$_3$). *Only the signals of major conformer are listed. **Assignment based on the pattern observed for the other compounds.

^{31}P NMR (202 MHz, C_6D_6, 25 °C): δ (ppm) 290.84 (t, $^3J_{P–H}$ = 18.1 Hz; minor conformer), 283.95 (t, $^3J_{P–H}$ = 15.2 Hz; major conformer), 273.40 (t, $^3J_{P–H}$ = 17.4 Hz; minor conformer).

^{29}Si{^{1}H} NMR (99 MHz, C_6D_6, 25 °C): δ (ppm) 0.21.

HRMS (APCI): m/z [M+H]$^+$ calcd for $C_{45}H_{73}AlN_2OPSi^+$: 743.5040; found: 743.5042.

ICPN$_3$Bn (7). A solution of **5a** (26 mg, 0.033 mmol) in C_6D_6 (circa 0.5 mL) was placed into an NMR tube equipped with a J. Young gas-tight valve. A 0.1 M solution of elemental iodine in toluene (0.33 mL, 0.033 mmol) was added to the NMR tube using a syringe. The resulting solution was carefully shaken and heated at 80 °C for 1 hour. The reaction completion was determined by ^{31}P{^{1}H} NMR spectroscopy. All volatiles were removed under dynamic vacuum. The crude product was purified by the column chromatography on SiO_2 using benzene as eluent. The fraction

with $R_f \sim 0.4$ was collected. The solvent was removed under dynamic vacuum, and the residue was recrystallized from a toluene/hexane mixture. The first crop of colorless crystals was isolated and dried under dynamic vacuum (4.2 mg, 0.014 mmol). The mother liquor was concentrated and stored at −35 °C overnight to isolate a second crop of crystals (2.1 mg, 0.007 mmol). Yield: 63.5% (combined, 6.3 mg, 0.021 mmol)

^{1}H NMR (500 MHz, C_6D_6, 25 °C): δ (ppm) 6.97 – 6.89 (m, 3H, overlapping *meta*- and *para*-C_6H_5), 6.80 (dd, $^3J_{H\text{-}H}$ = 7.3, $^4J_{H\text{-}H}$ = 2.1 Hz, 2H; *ortho*-C_6H_5), 4.92 (d, $^3J_{H\text{–}P}$ = 6.7 Hz, 2H; C$H_2$$C_6H_5$).

^{13}C{^{1}H} NMR (126 MHz, C_6D_6, 25°C): δ (ppm) 136.53 (Ph *ipso*-Ar*C*), 129.08 (*para*- or *meta*-Ar*C*H), 128.72 (Ph *para*- or *meta*-Ar*C*H), 128.48 (Ph *ortho*- Ar*C*H), 121.69 (d, $^1J_{C\text{-}P}$ = 81.1 Hz; I*C*PN_3), 55.49 (d, $^2J_{C\text{-}P}$ = 11.4 Hz; *C*$H_2C_6H_5$).

^{31}P{^{1}H} NMR (202 MHz, C_6D_6, 25 °C): δ (ppm) 187.78.

HRMS (APCI): m/z $[M+H]^+$ calcd for $C_8H_8N_3IP^+$: 303.9495; found: 303.9501.

[Au(PMe$_3$)(CPN$_3$Bn)] (8). A solution of **5a** (23.1 mg, 0.029 mmol) in C_6D_6 (0.5 mL) was added to Me_2S·AuCl (8.1 mg, 0.027 mmol). The resulting clear light-yellow solution was transferred to an NMR tube equipped with a J. Young gas-tight valve and left at room temperature for 30 minutes. A 0.05 M solution of PMe_3 in toluene (0.66 mL, 0.033 mmol) was added using a syringe. The resulting white suspension was stirred overnight at room temperature to form a clear solution. Volatiles were removed under dynamic vacuum. The resulting white precipitate was washed with hexane (5 × 2 mL) and dried under dynamic vacuum. Yield: 67% (8.7 mg, 0.019 mmol)

^{1}H NMR (500 MHz, C_6D_6, 25 °C): δ (ppm) 6.87 (d, $^3J_{H\text{–}H}$ = 7.7, 2H; *ortho*-C_6H_5), 6.83 – 6.64 (m, 3H; overlapping *meta*- and *para*-C_6H_5), 5.28 (d, $^3J_{H\text{–}P}$ = 4.8 Hz, 2H, C$H_2$$C_6H_5$), 0.28 (d, $^2J_{H\text{–}P}$ = 9.7 Hz, 9H, P(CH_3)$_3$).

^{13}C NMR (126 MHz, C_6D_6, 25 °C): δ (ppm) 213.81 (dd, $^{1}J_{C-P}$ = 132.8 Hz, $^{2}J_{C-P}$ = 86.0 Hz; *C*PN$_3$−Bn), 139.03 (Ph *ipso*- Ar*C*), 128.77 (Ph *para*- or *meta*-Ar*C*H), 128.35 (Ph *ortho*- Ar*C*H), 127.76 (Ph *para*- or *meta*- Ar*C*H), 55.36 (d, $^{2}J_{C-P}$ = 10.2 Hz; *C*H$_2$Ph), 14.79 (d, $^{2}J_{C-P}$ = 32.6 Hz; P(*C*H$_3$)$_3$).

$^{31}P\{^{1}H\}$ NMR (202 MHz, C_6D_6, 25 °C): δ (ppm) 201.45 (d, $^{2}J_{P-P}$ = 31.1 Hz; C*P*N$_3$−Bn), 3.44 (d, $^{2}J_{P-P}$ = 37.7 Hz, *P*Me$_3$).

HRMS (APCI): m/z $[M+H]^+$ calcd for $C_{11}H_{17}N_3AuP_2^+$: 450.0558; found: 450.0565.

^{1}H NMR data for Al(^{Dipp}NacNac)(OSi^{i}Pr$_3$)I

^{1}H NMR (500 MHz, C_6D_6, 25 °C): δ (ppm) 7.18 (dd, $^{3}J_{H-H}$ = 7.7, $^{4}J_{H-H}$ = 1.9 Hz, 2H; Dipp ArH), 7.16 – 7.13 (m, overlaps with C_6D_5H, prevents integration; Dipp ArH), 7.09 (dd, $^{3}J_{H-H}$ = 7.4, $^{4}J_{H-H}$ = 1.9 Hz, 2H; Dipp ArH), 5.08 (s, 1H; NacNac γ-H), 3.96 (sept, $^{3}J_{H-H}$ = 6.7 Hz, 2H; Dipp C*H*(CH$_3$)$_2$), 3.30 (sept, $^{3}J_{H-H}$ = 6.7 Hz, 2H; Dipp C*H*(CH$_3$)$_2$), 1.54 (d, $^{3}J_{H-H}$ = 6.6 Hz, 6H; Dipp CH(C*H*$_3$)$_2$), 1.51 (s, 6H; NacNac NCC*H*$_3$), 1.40 (d, $^{3}J_{H-H}$ = 6.8 Hz, 6H; Dipp CH(C*H*$_3$)$_2$), 1.20 (d, $^{3}J_{H-H}$ = 6.8 Hz, 6H; Dipp CH(C*H*$_3$)$_2$), 0.96 (d, $^{3}J_{H-H}$ = 6.8 Hz, 6H; Dipp CH(C*H*$_3$)$_2$), 0.92 – 0.88 (m, 3H; Si[C*H*(CH$_3$)$_2$]$_3$), 0.86 (d, $^{3}J_{H-H}$ = 6.3 Hz, 18H; Si[CH(C*H*$_3$)$_2$]$_3$).

^{1}H NMR data for Al(^{Dipp}NacNac)(OSi^{i}Pr$_3$)Cl

^{1}H NMR (500 MHz, C_6D_6, 25 °C): δ (ppm) 7.10 – 6.92 (m, overlaps with C_6D_5H and **7**, prevents integration; Dipp ArH), 6.06 (s, 1H; NacNac γ-H), 3.30 (sept, $^{3}J_{H-H}$ = 6.8 Hz, 2H; Dipp C*H*(CH$_3$)$_2$), 3.25 (sept, $^{3}J_{H-H}$ = 6.8 Hz, 2H; Dipp C*H*(CH$_3$)$_2$), 1.88 (s, 6H; NacNac NCC*H*$_3$), 1.40 (d, $^{3}J_{H-H}$ =

6.8 Hz, 6H; Dipp CH(CH_3)$_2$), 1.08 (d, $^3J_{H-H}$ = 6.7 Hz, 6H; Dipp CH(CH_3)$_2$), 1.00 (d, $^3J_{H-H}$ = 6.8 Hz, 6H; Dipp CH(CH_3)$_2$), 0.94 (sept, $^3J_{H-H}$ = 7.5 Hz, 3H; Si[CH(CH_3)$_2$]$_3$), 0.76 (d, $^3J_{H-H}$ = 7.5 Hz, 18H; Si[CH(CH_3)$_2$]$_3$), 0.24 (d, $^3J_{H-H}$ = 6.8 Hz, 6H; Dipp CH(CH_3)$_2$).

2. NMR and IR spectra

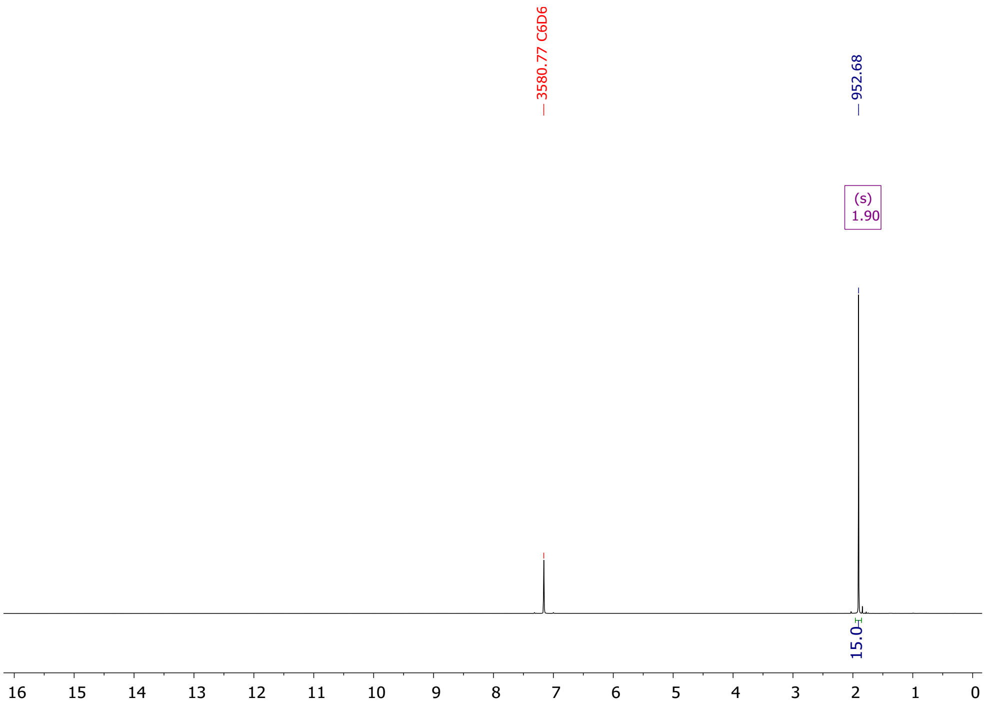

Figure S1. ^{1}H NMR spectrum of $[AlCp^{*}]_4$ (500 MHz, C_6D_6, 25°C).

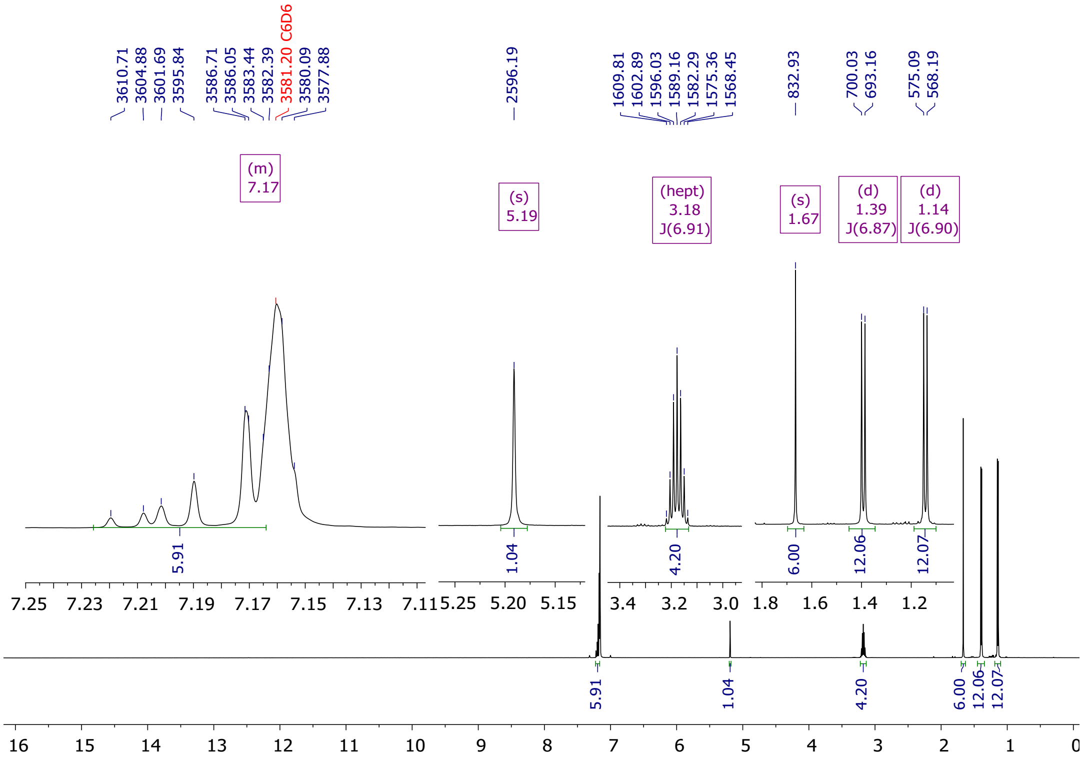

Figure S2. ^{1}H NMR spectrum of Al(^{DIPP}NacNac) (500 MHz, C_6D_6, 25°C).

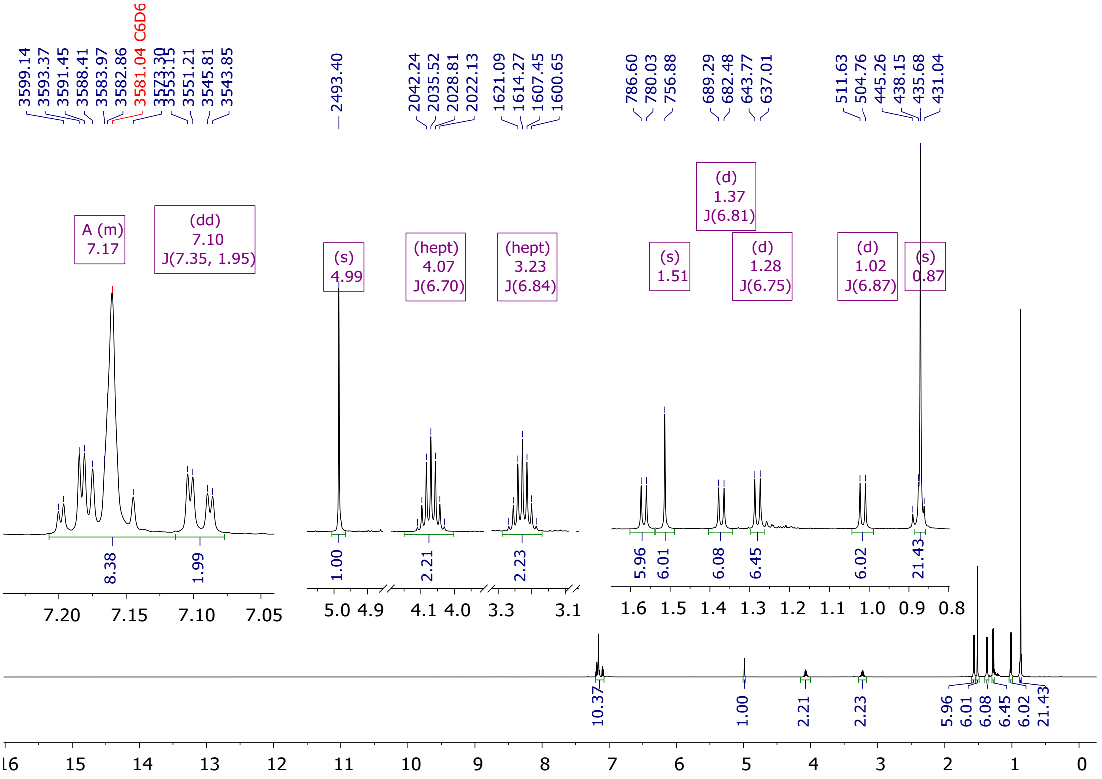

Figure S3. ^{1}H NMR spectrum of **1** (500 MHz, C_6D_6, 25°C).

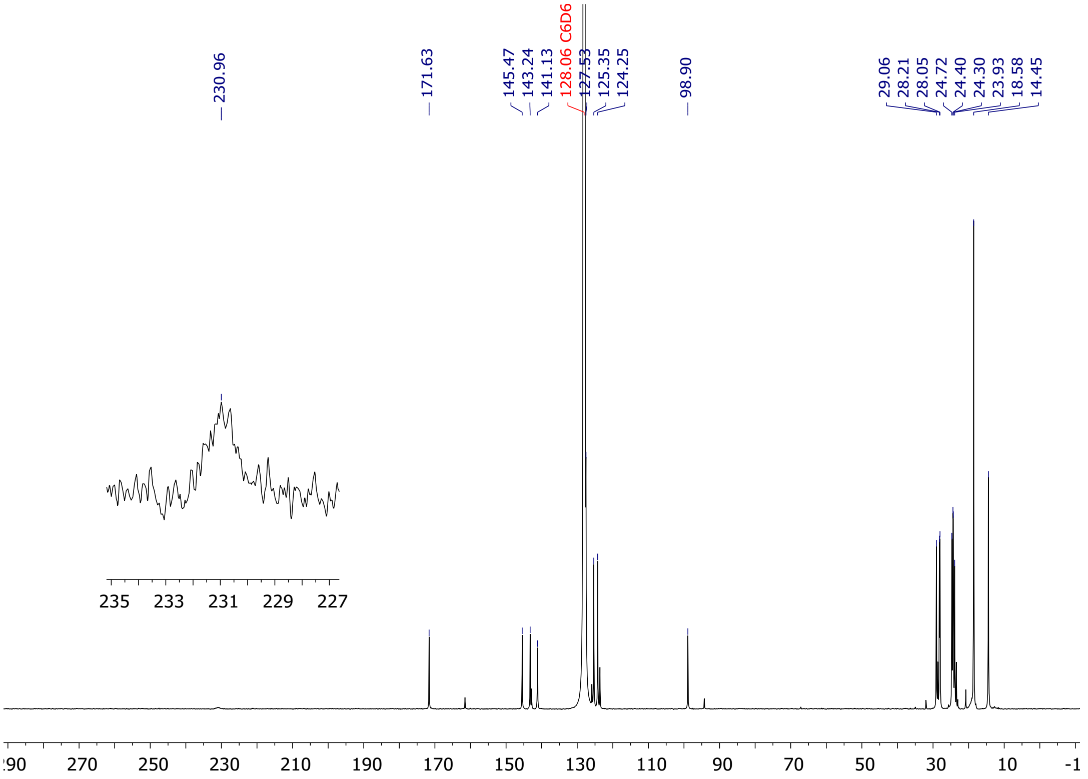

Figure S4. $^{13}C\{^{1}H\}$ NMR spectrum of **1** (126 MHz, C_6D_6, 25°C).

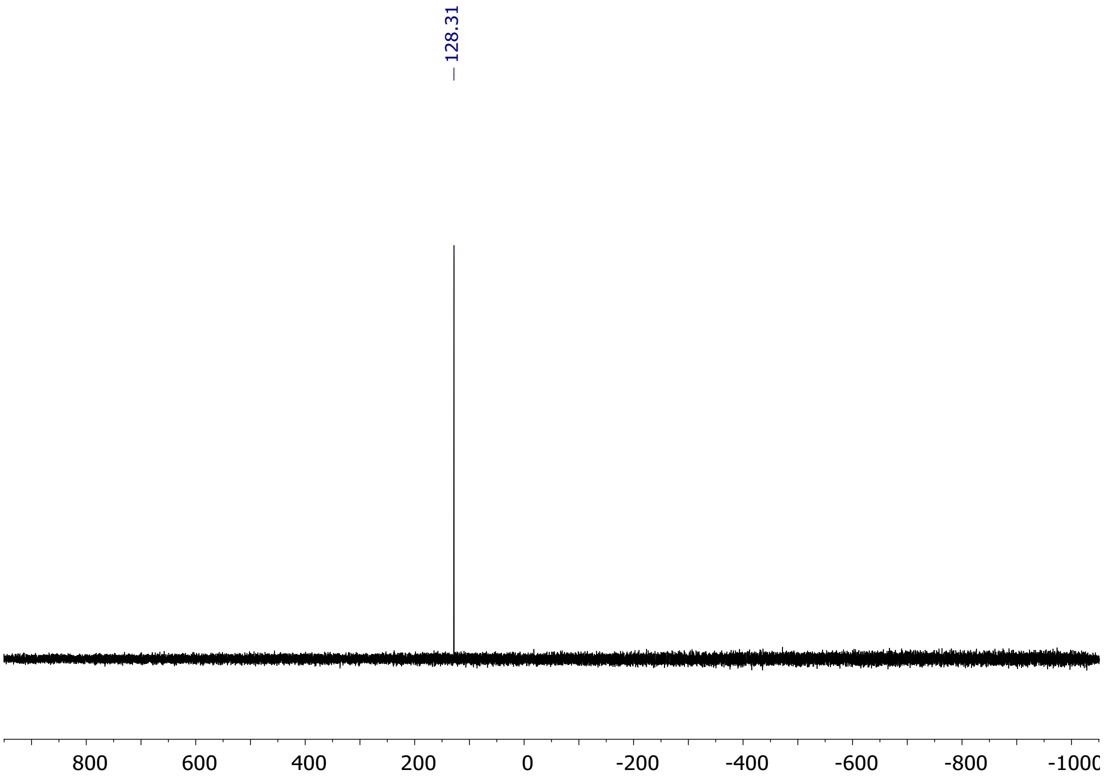

Figure S5. $^{31}P\{^1H\}$ NMR spectrum of **1** (202 MHz, C_6D_6, 25°C).

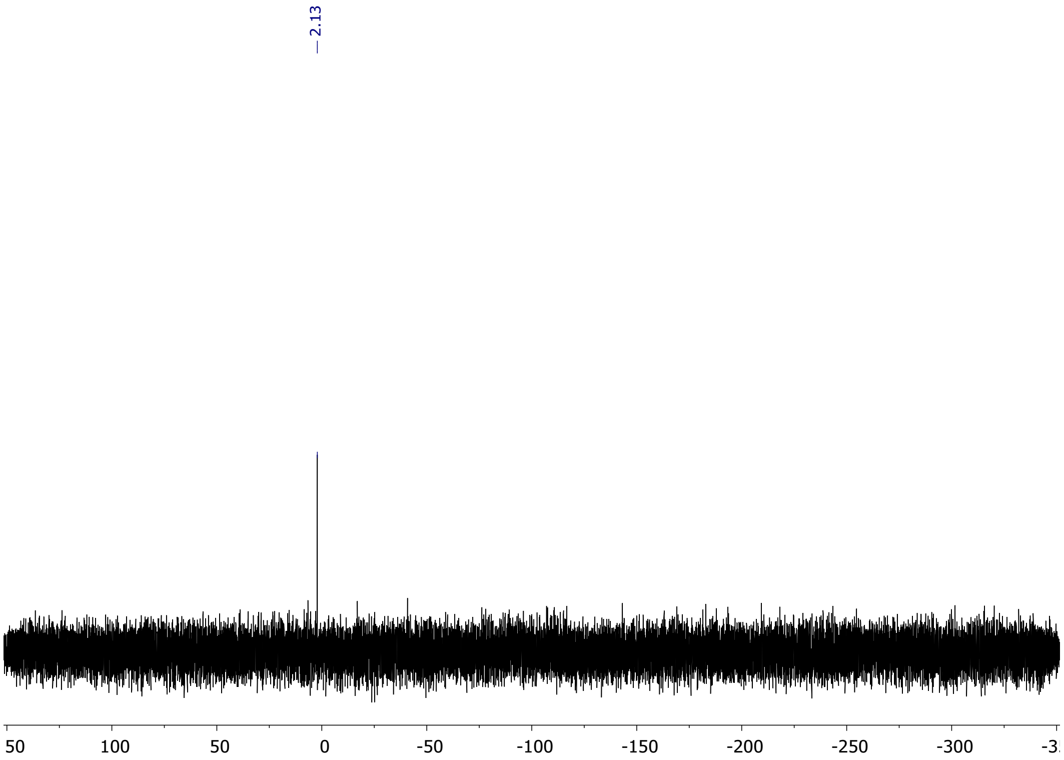

Figure S6. $^{29}Si\{1H\}$ NMR spectrum of **1** (99 MHz, C_6D_6, 25°C).

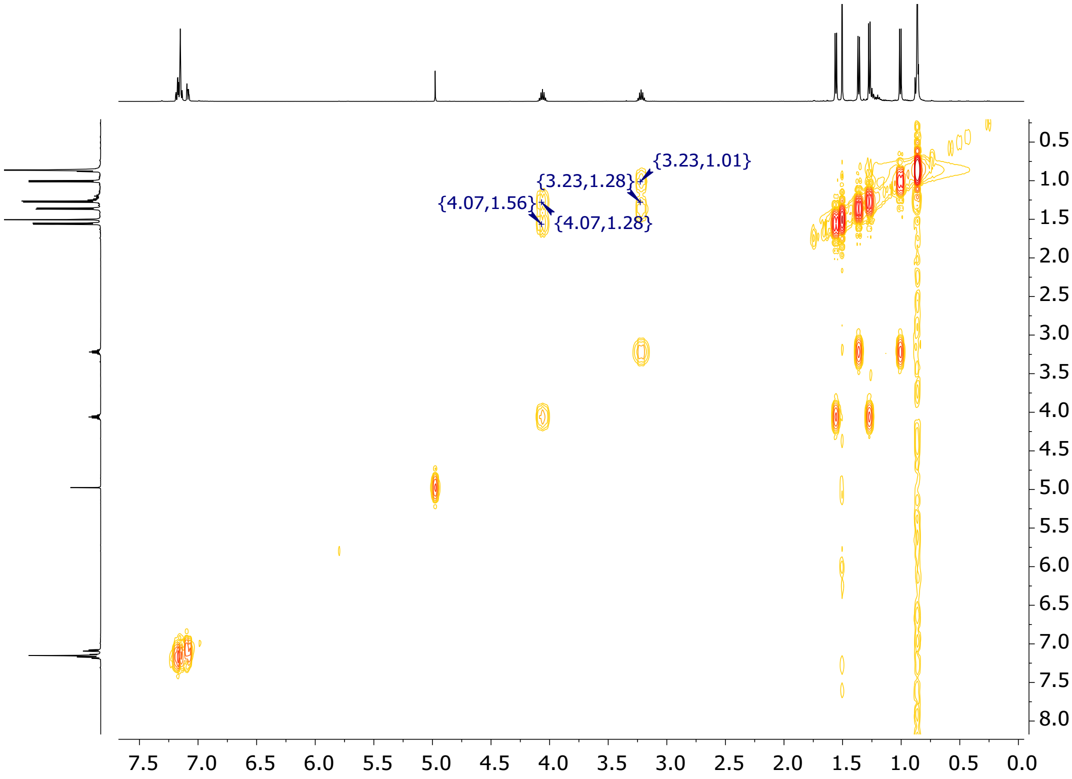

Figure S7. ^{1}H–^{1}H COSY NMR spectrum of **1** (500 MHz, C_6D_6, 25°C).

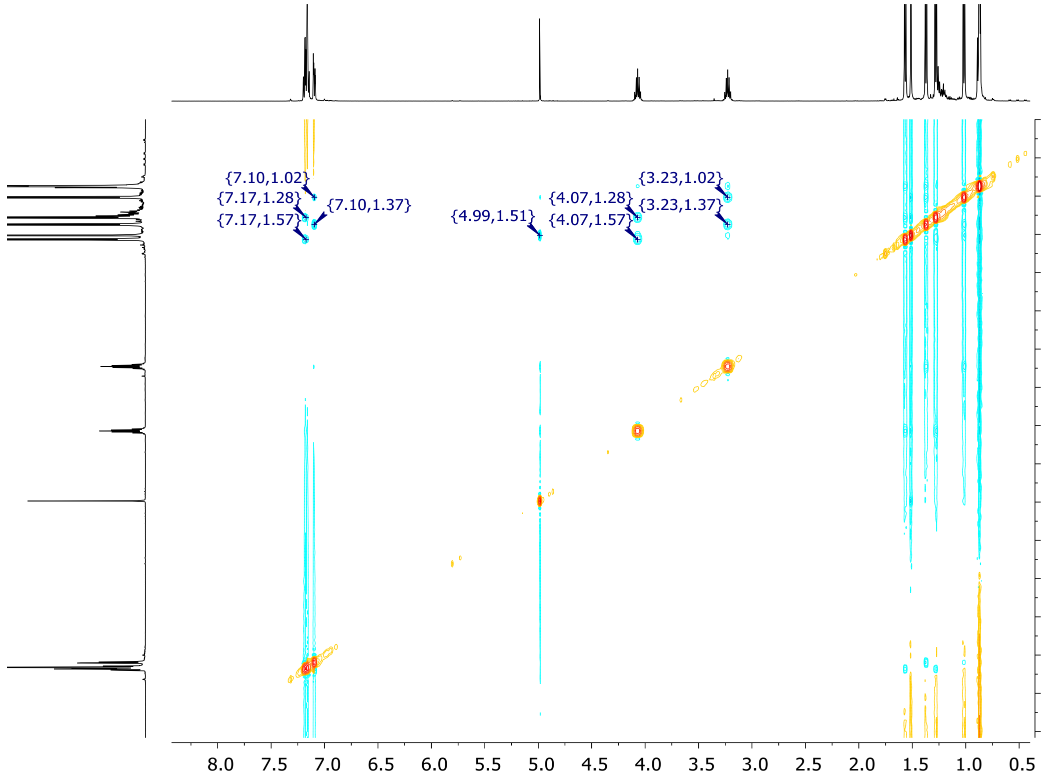

Figure S8. ^{1}H–^{1}H NOESY NMR spectrum of **1** (500 MHz, C_6D_6, 25°C).

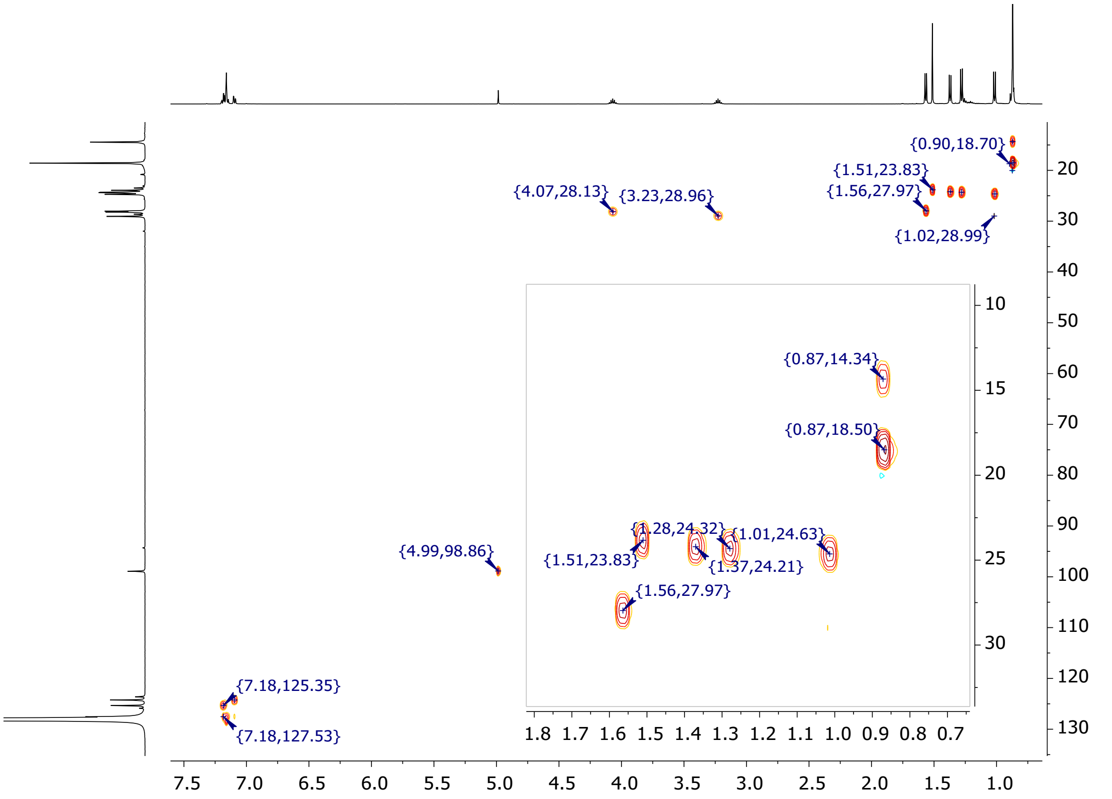

Figure S9. ^{1}H–$^{13}C\{^{1}H\}$ HSQC NMR spectrum of **1** (500 & 126 MHz, C_6D_6, 25°C).

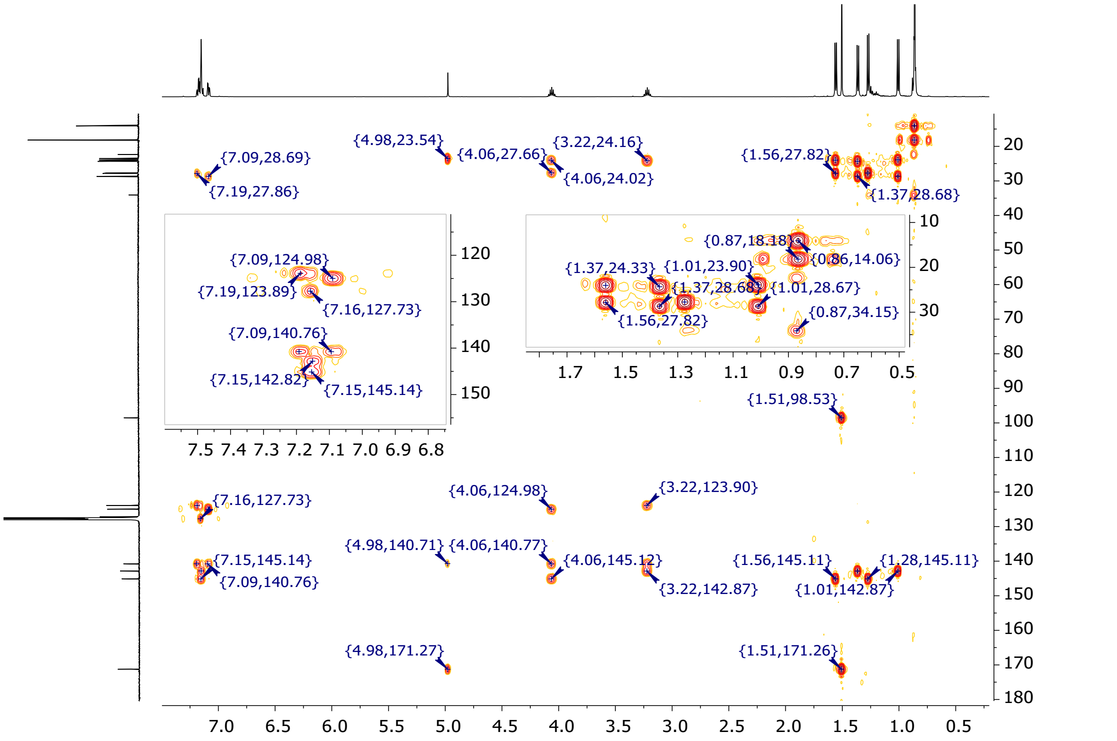

Figure S10. ^{1}H–$^{13}C\{^{1}H\}$ HMBC NMR spectrum of **1** (500 & 126 MHz, C_6D_6, 25°C).

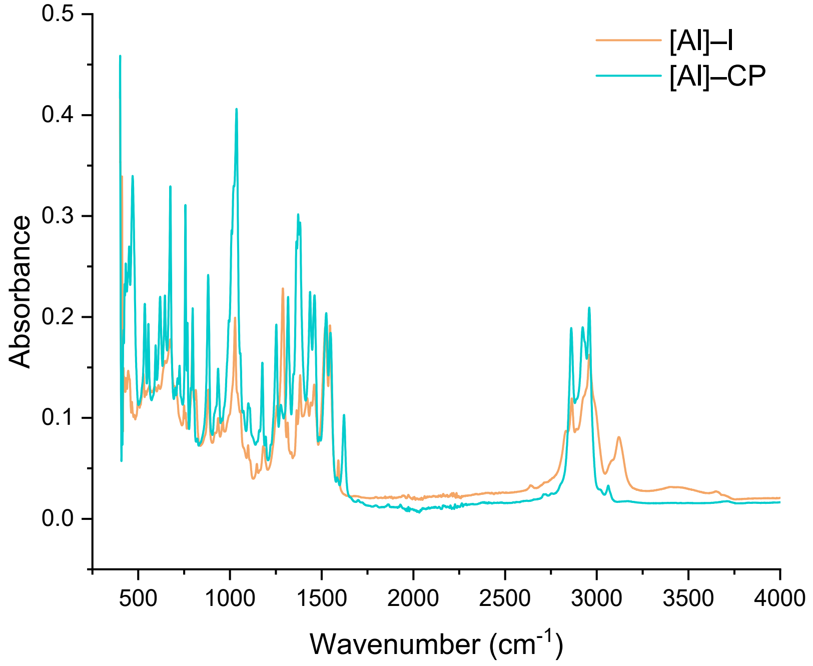

Figure S11. IR spectrum of **1** (25°C).

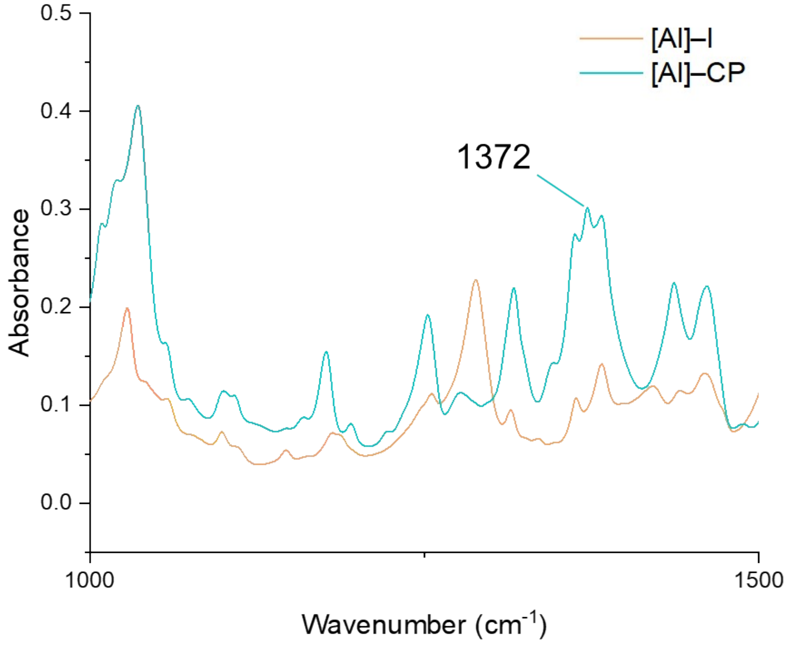

Figure S12. IR spectrum of **1** (25°C, 1000–1500 cm^{-1} insert).

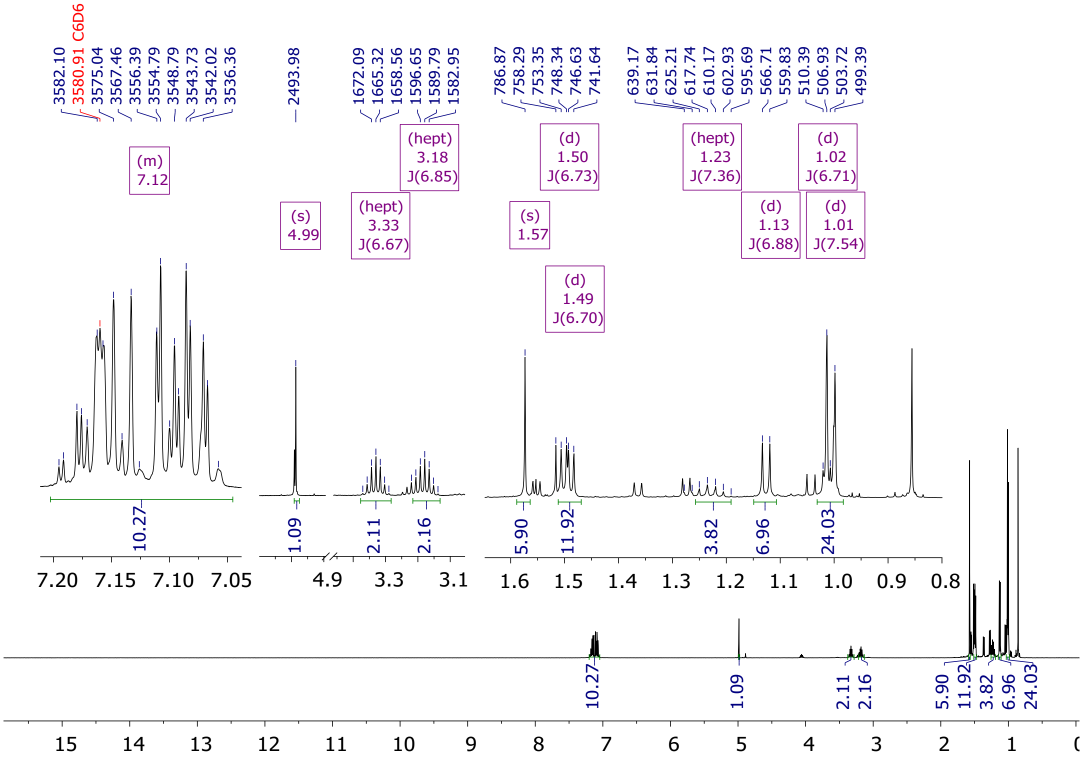

Figure S13. ^{1}H NMR spectrum of **2** (500 MHz, C_6D_6, 25°C).

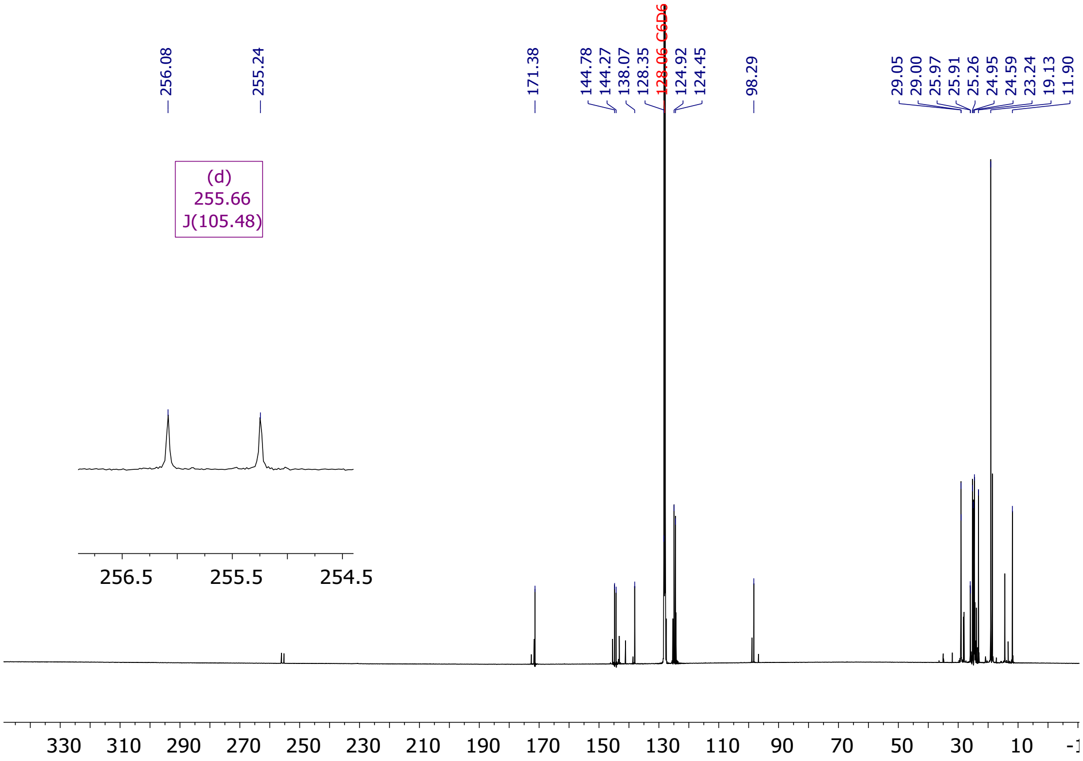

Figure S14. $^{13}C\{^{1}H\}$ NMR spectrum of **2** (126 MHz, C_6D_6, 25°C).

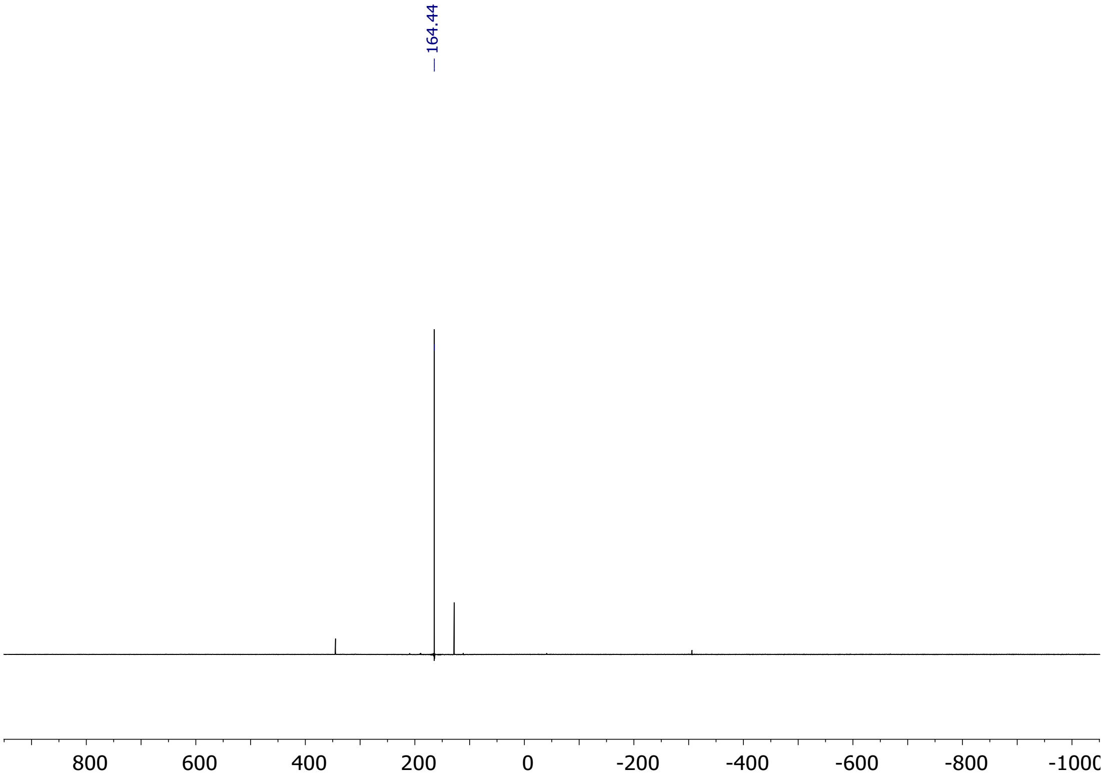

Figure S15. $^{31}P\{^1H\}$ NMR spectrum of **2** (202 MHz, C_6D_6, 25°C).

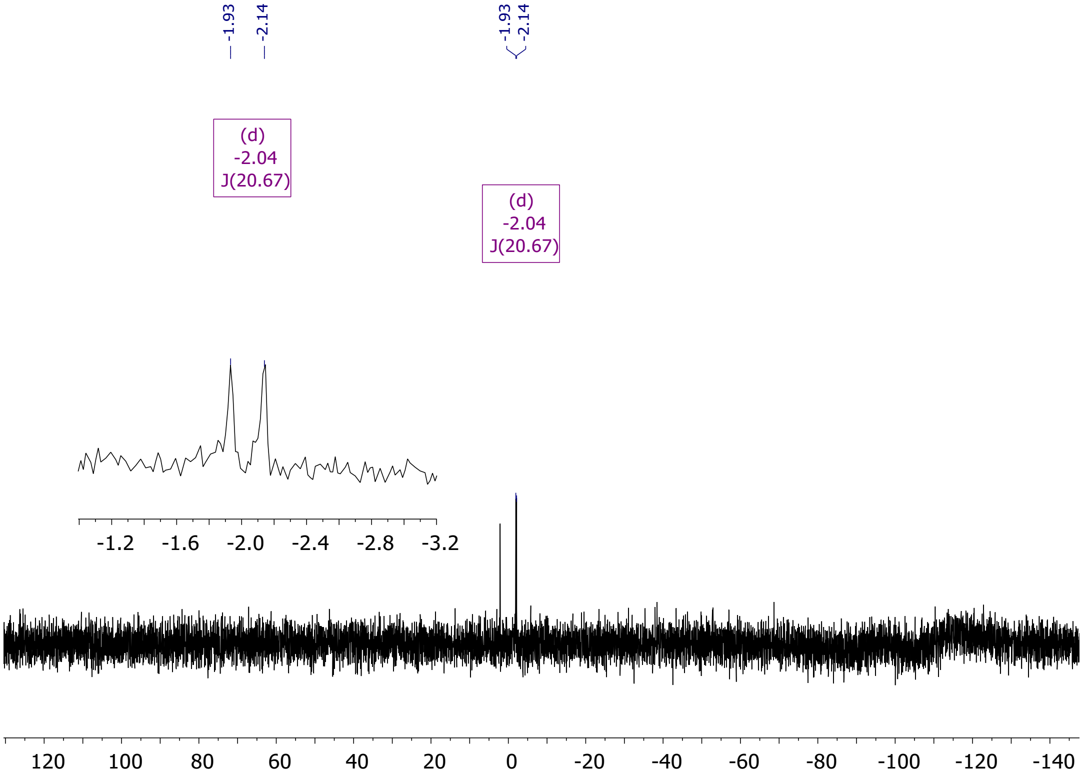

Figure S16. $^{29}Si\{^1H\}$ NMR spectrum of **2** (99 MHz, C_6D_6, 25°C).

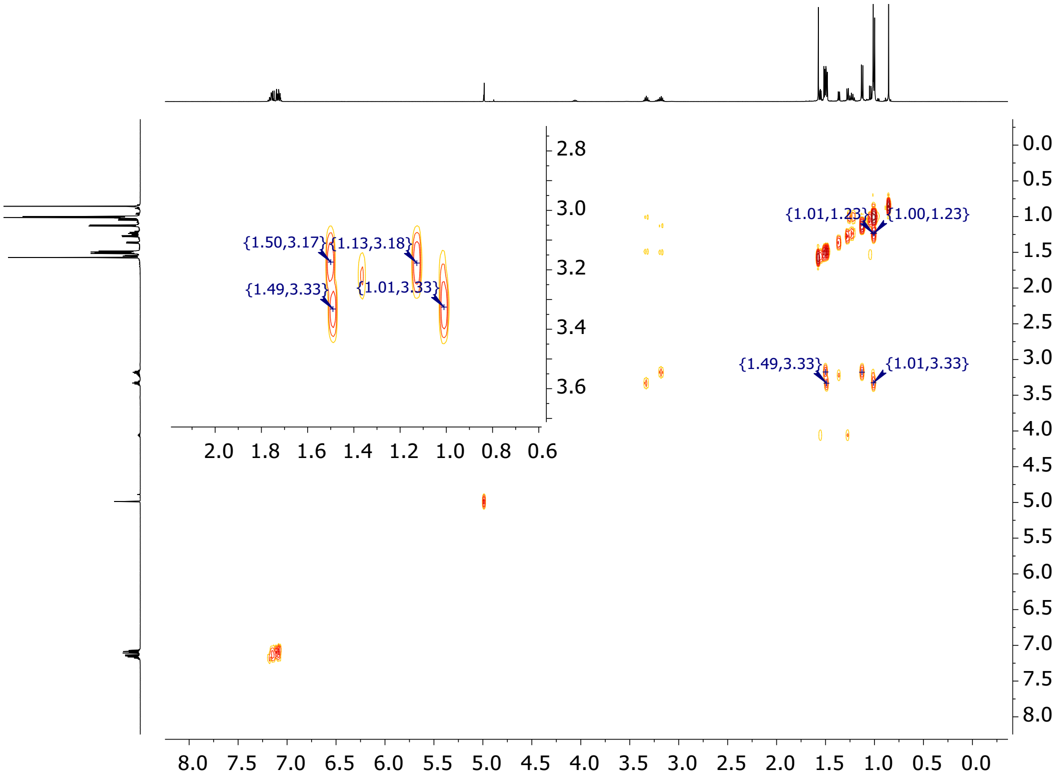

Figure S17. ^1H–^1H COSY NMR spectrum of **2** (500 MHz, C_6D_6, 25°C).

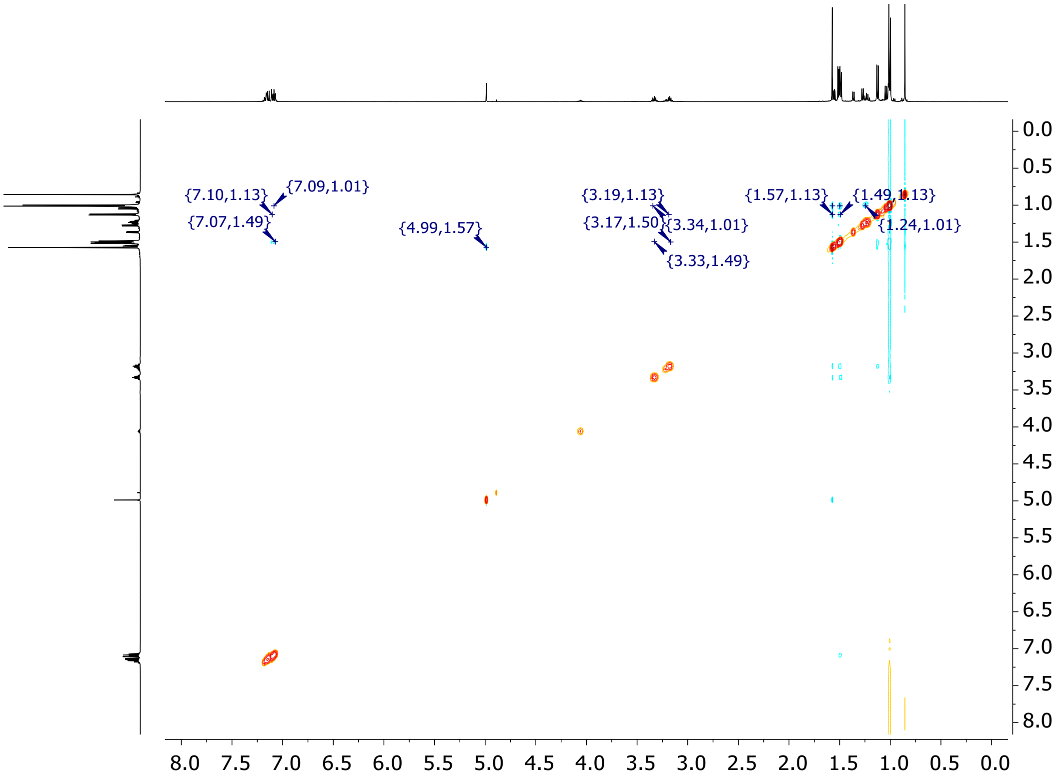

Figure S18. ^1H–^1H NOESY NMR spectrum of **2** (500 MHz, C_6D_6, 25°C).

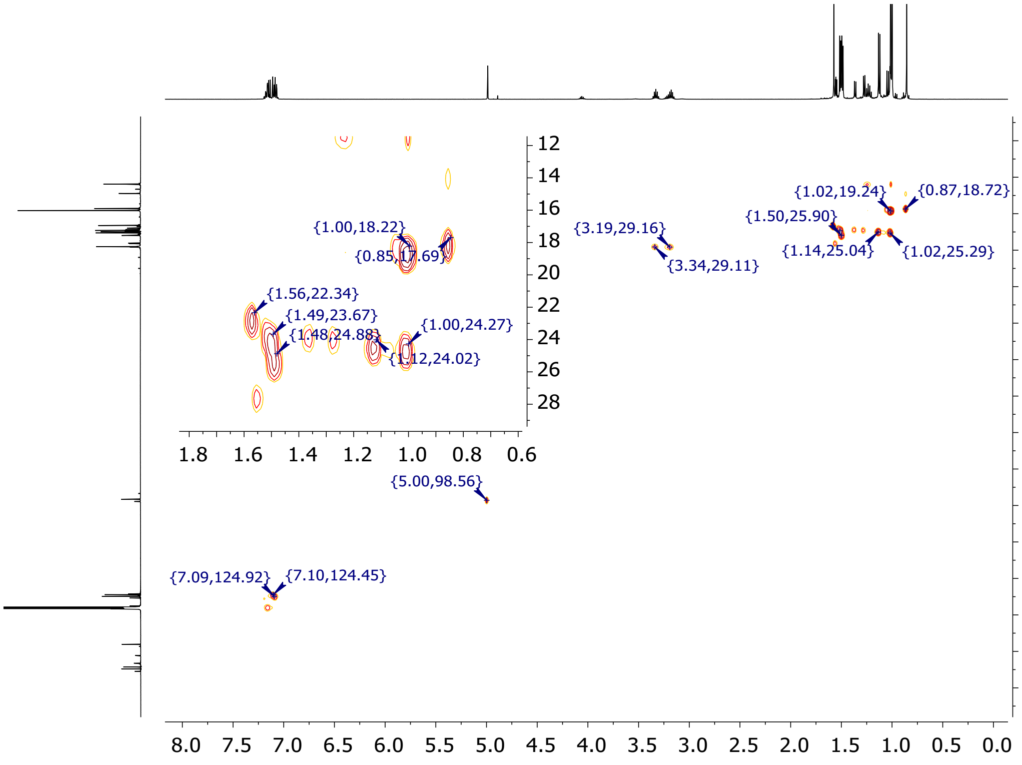

Figure S19. ^{1}H–$^{13}C\{^{1}H\}$ HSQC NMR spectrum of **2** (500 & 126 MHz, C_6D_6, 25°C).

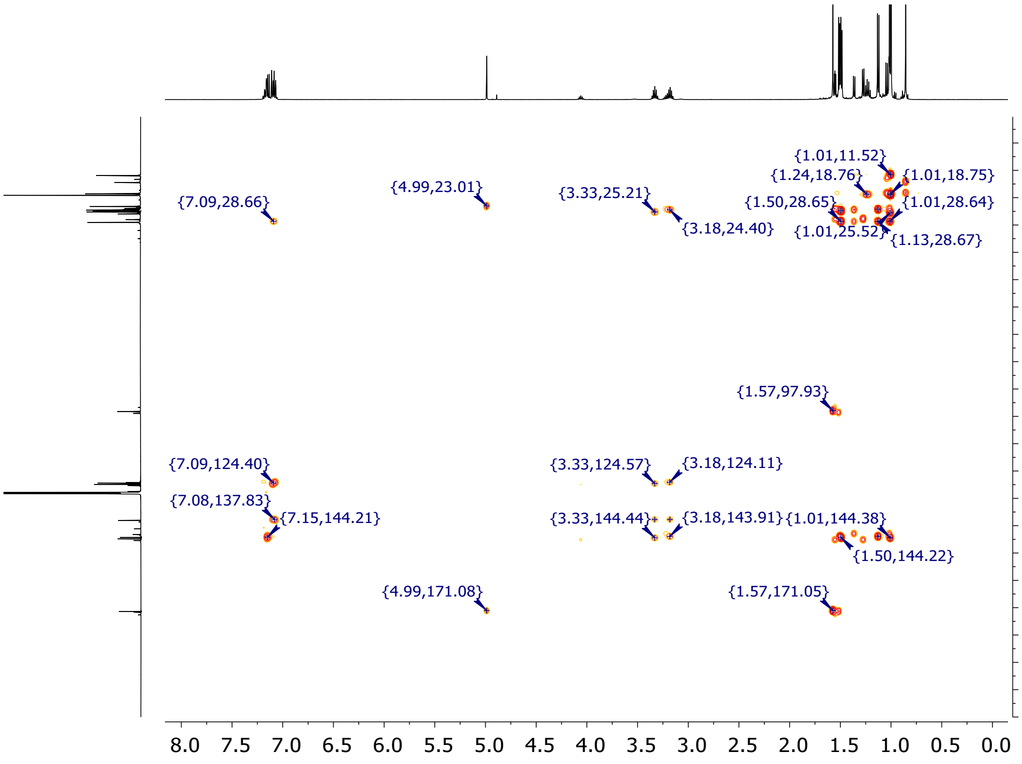

Figure S20. ^{1}H–$^{13}C\{^{1}H\}$ HMBC NMR spectrum of **2** (500 & 126 MHz, C_6D_6, 25°C).

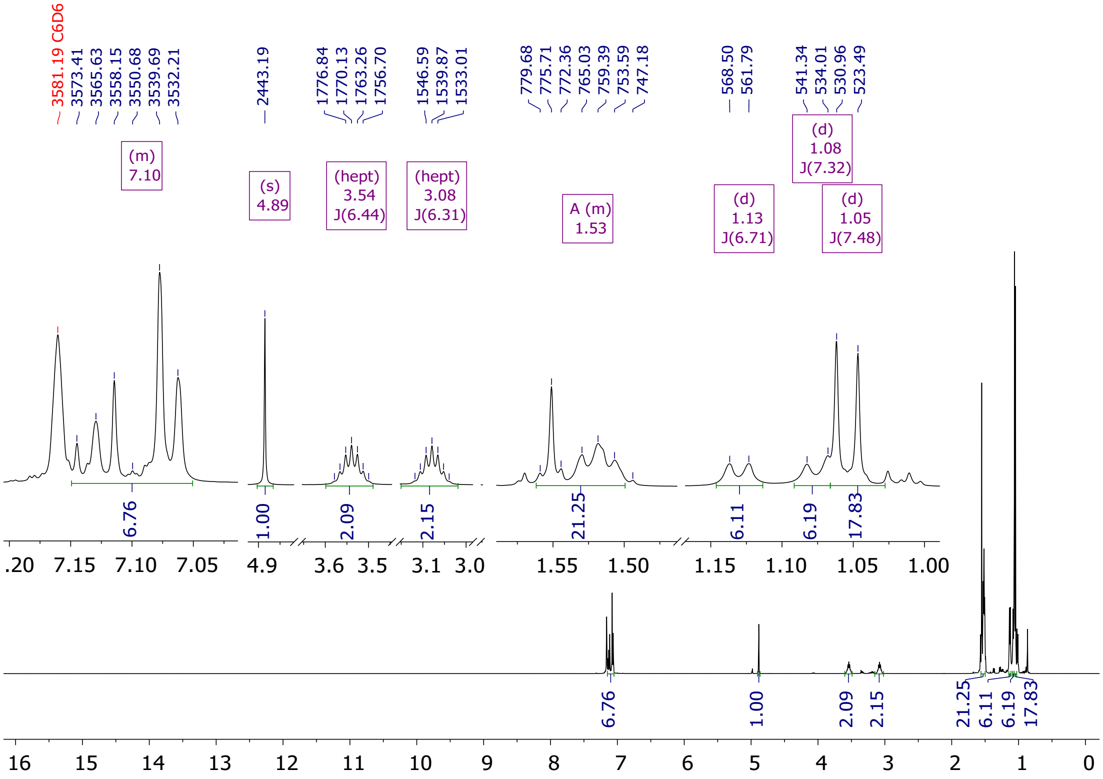

Figure S21. ^{1}H NMR spectrum of **3** (500 MHz, C_6D_6, 25°C).

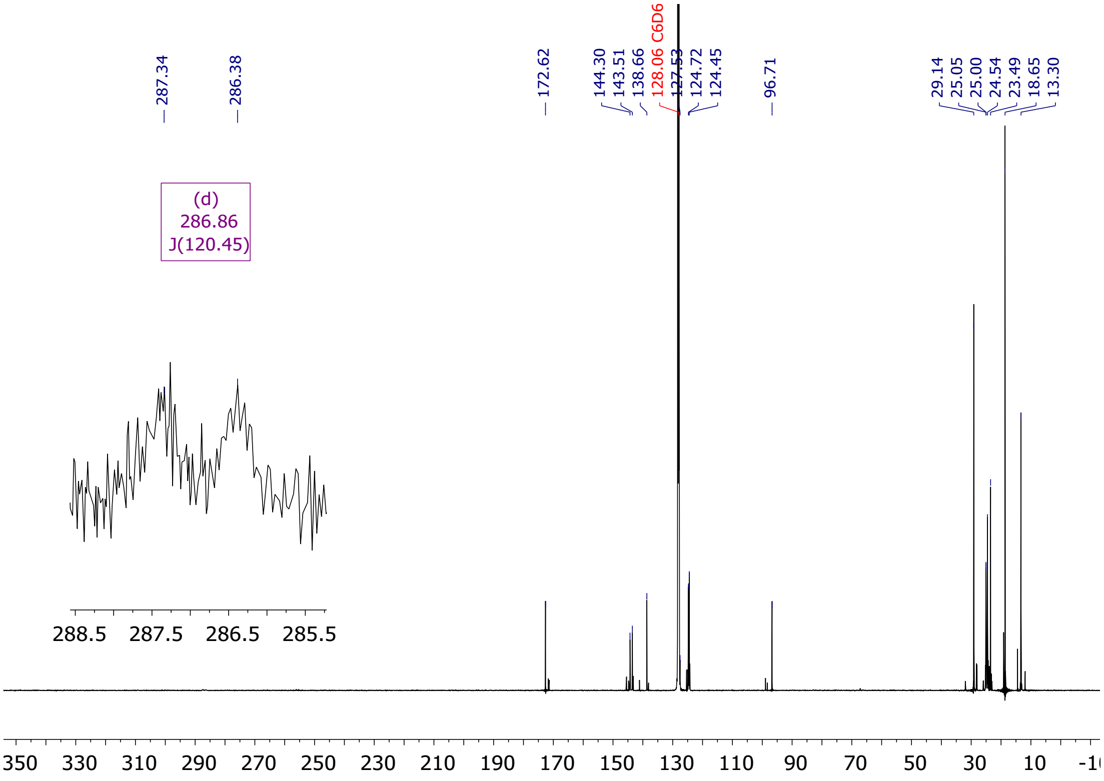

Figure S22. $^{13}C\{^{1}H\}$ NMR spectrum of **3** (126 MHz, C_6D_6, 25°C).

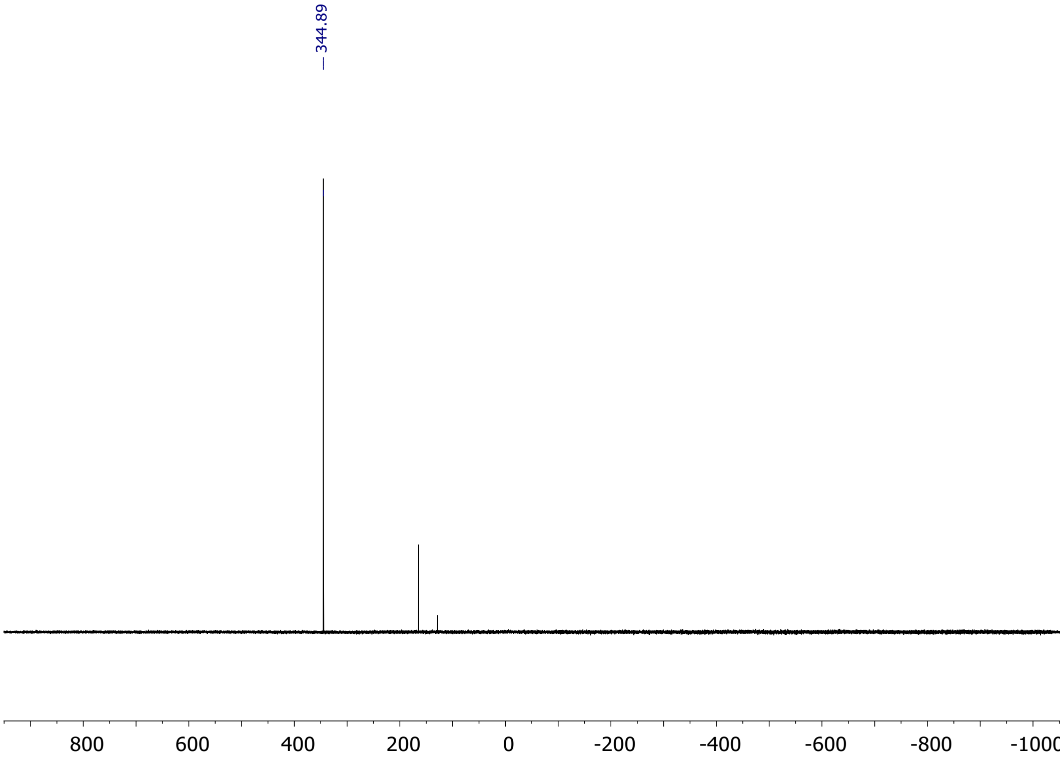

Figure S23. $^{31}P\{^1H\}$ NMR spectrum of **3** (202 MHz, C_6D_6, 25°C).

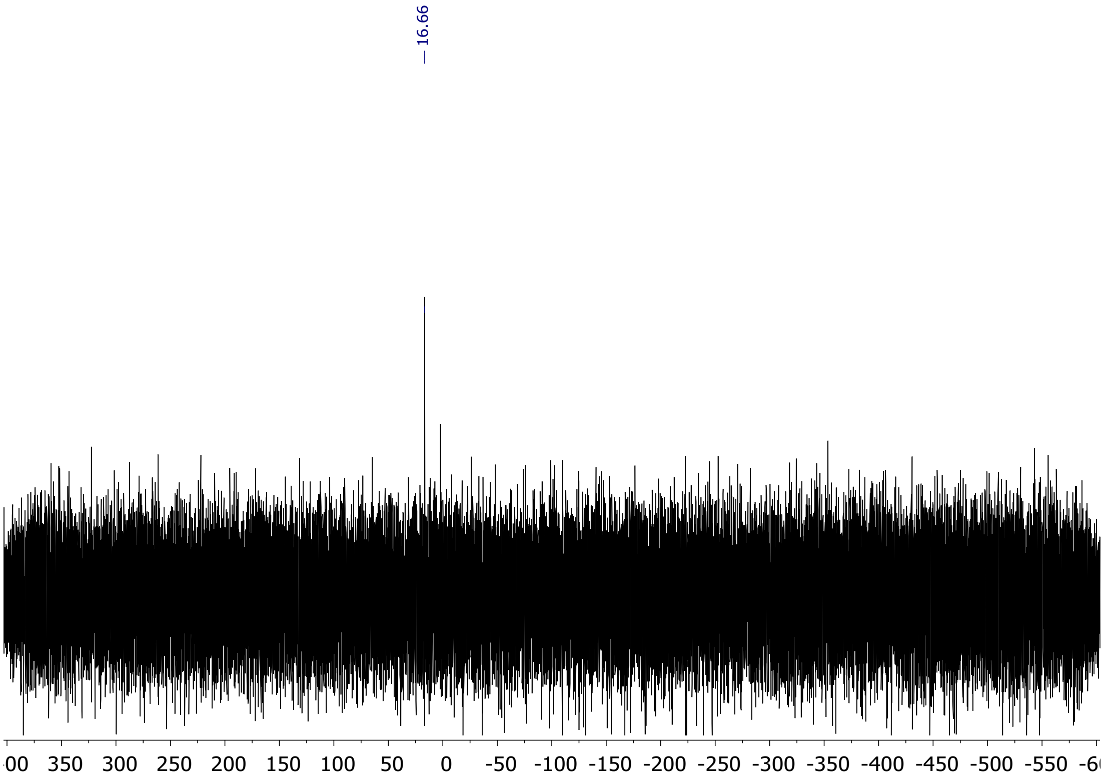

Figure S24. $^{29}Si\{1H\}$ NMR spectrum of **3** (99 MHz, C_6D_6, 25°C).

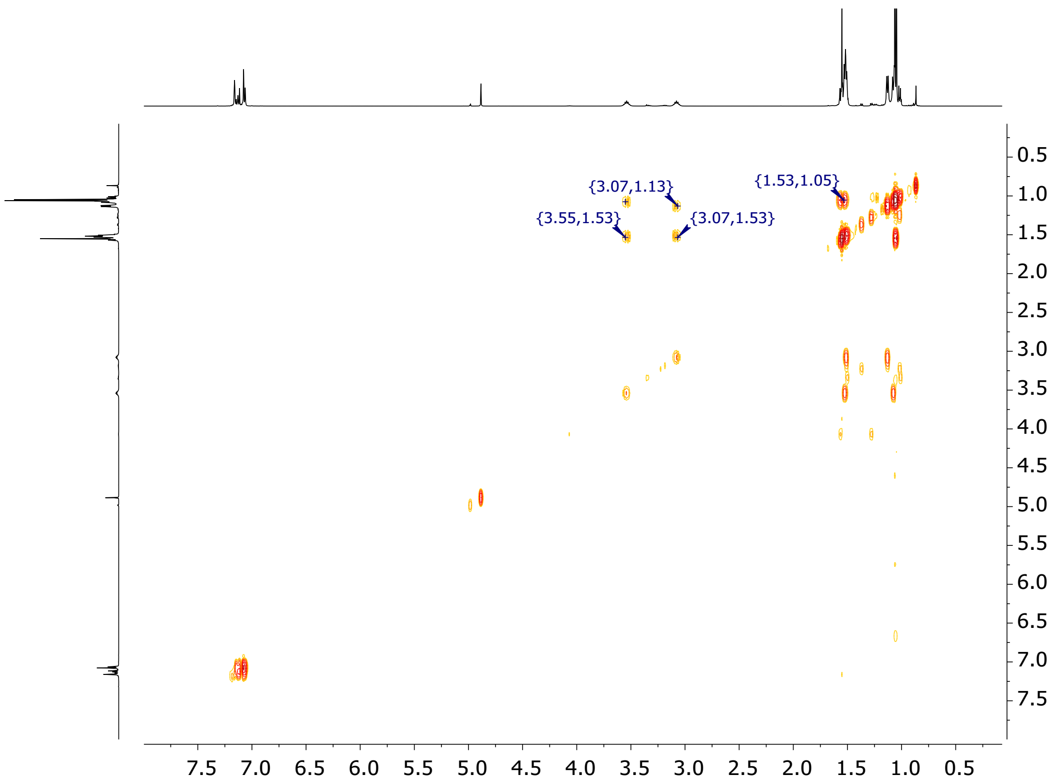

Figure S25. ^{1}H–^{1}H COSY NMR spectrum of **3** (500 MHz, C_6D_6, 25°C).

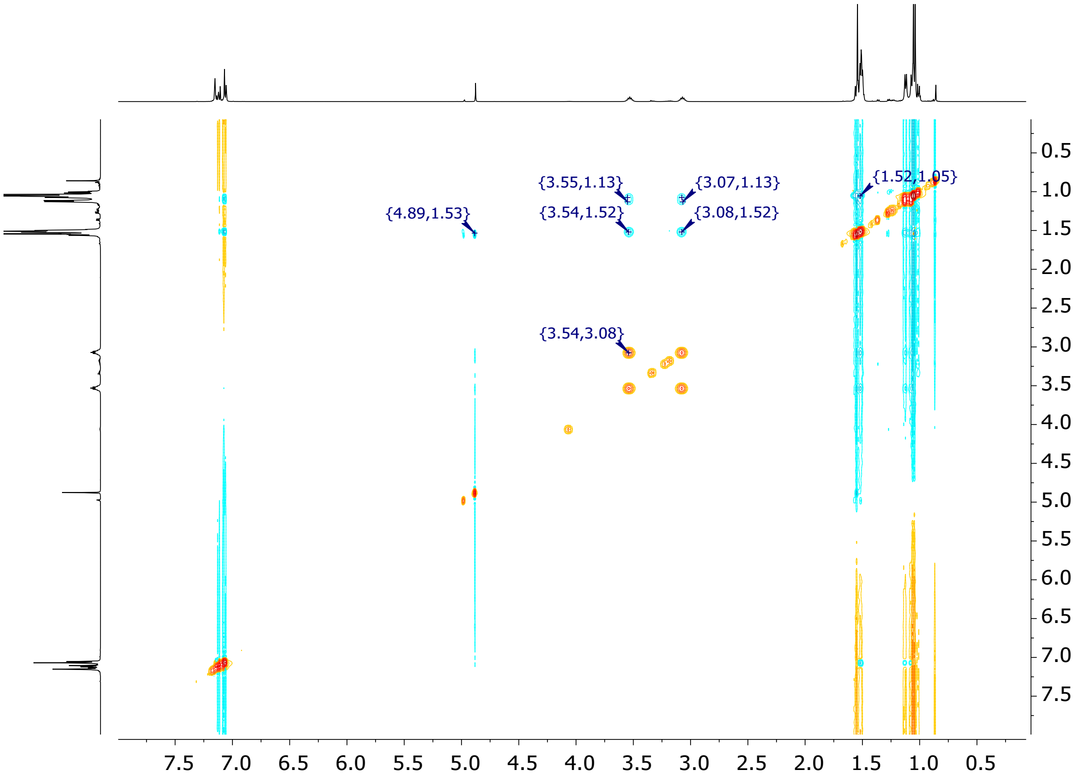

Figure S26. ^{1}H–^{1}H NOESY NMR spectrum of **3** (500 MHz, C_6D_6, 25°C).

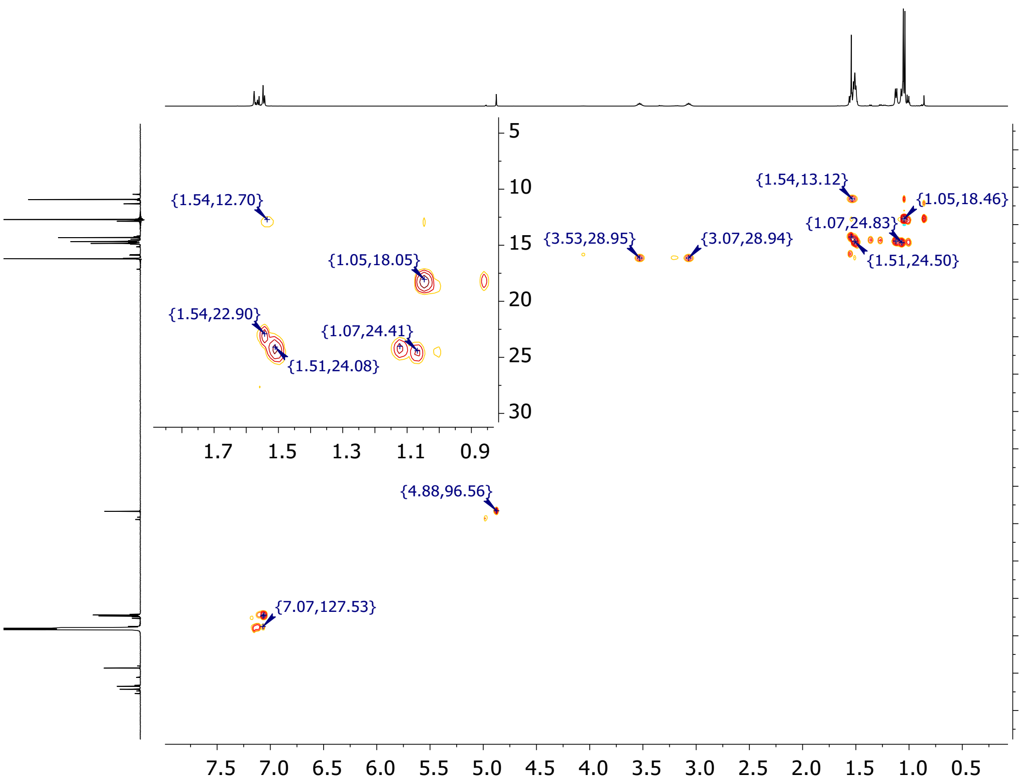

Figure S27. ^{1}H–$^{13}C\{^{1}H\}$ HSQC NMR spectrum of **3** (500 & 126 MHz, C_6D_6, 25°C).

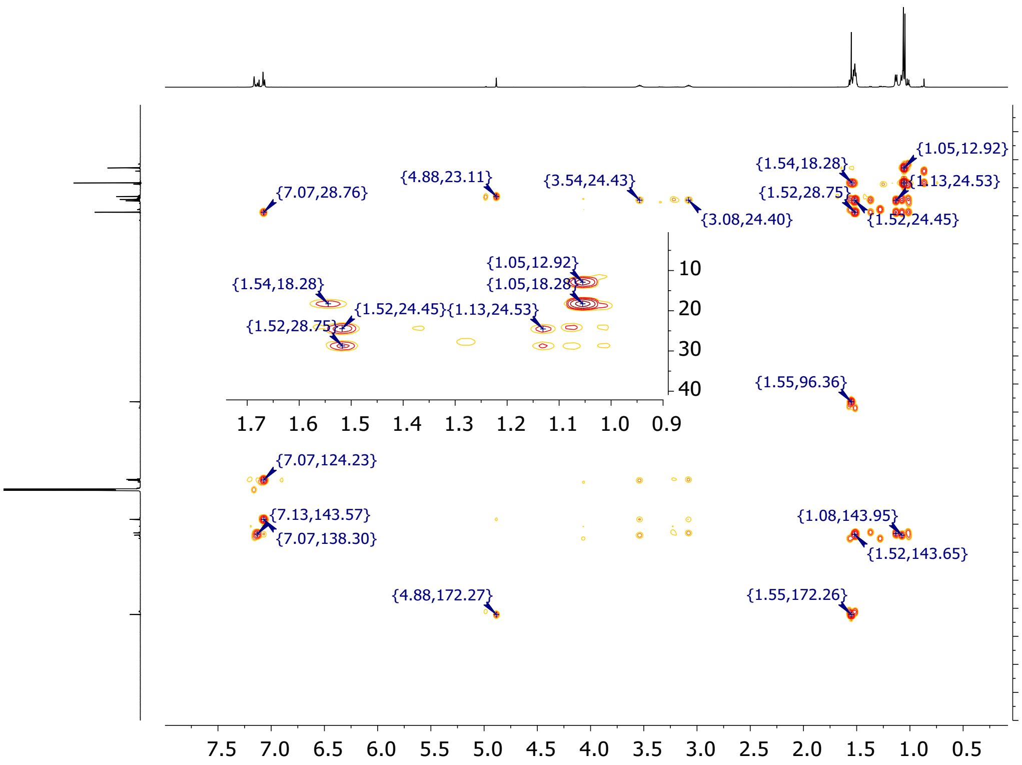

Figure S28. ^{1}H–$^{13}C\{^{1}H\}$ HMBC NMR spectrum of **3** (500 & 126 MHz, C_6D_6, 25°C).

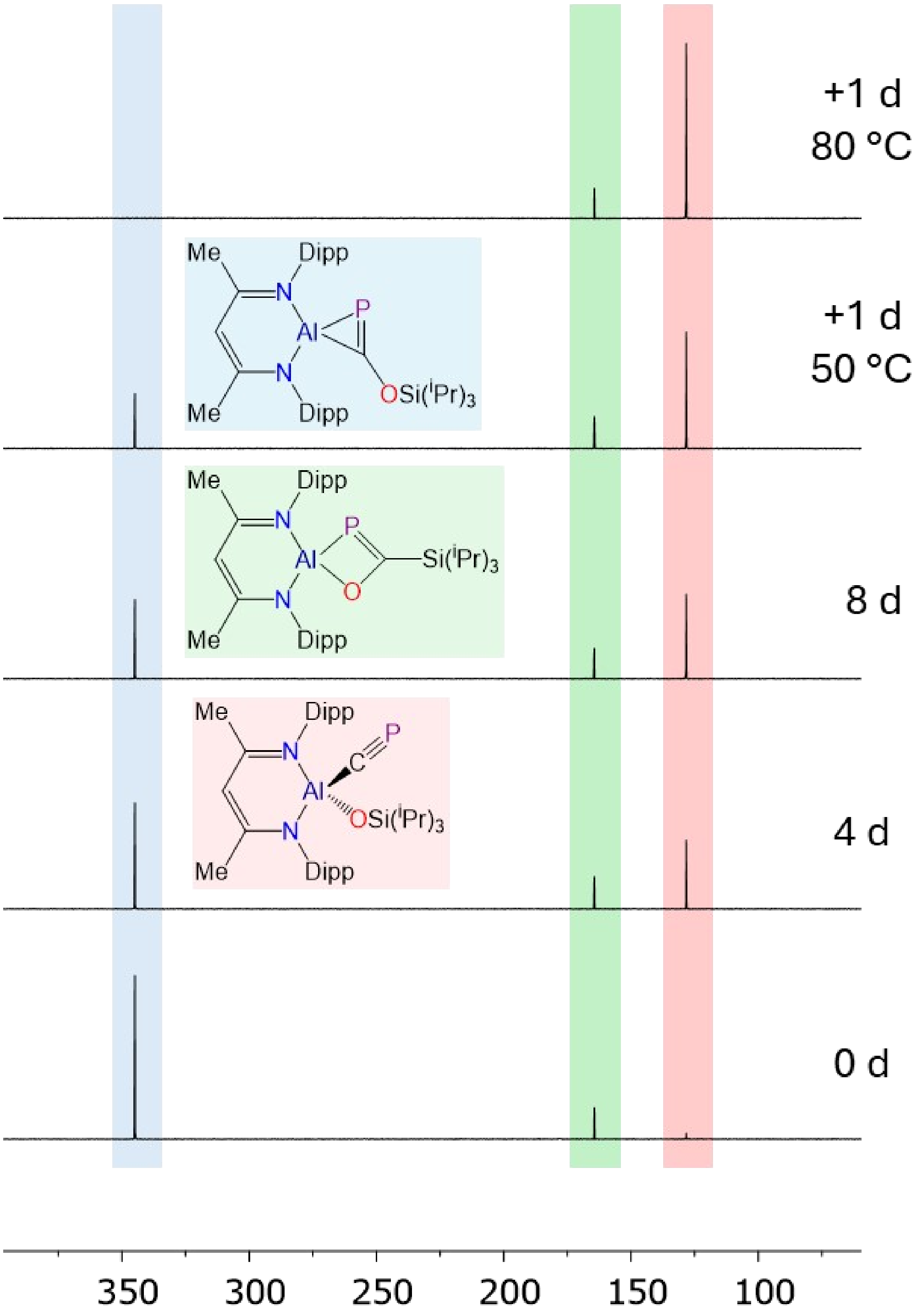

Figure S29. Decomposition of **3** (^{31}P NMR spectrum; 202 MHz, C_6D_6, 25°C).

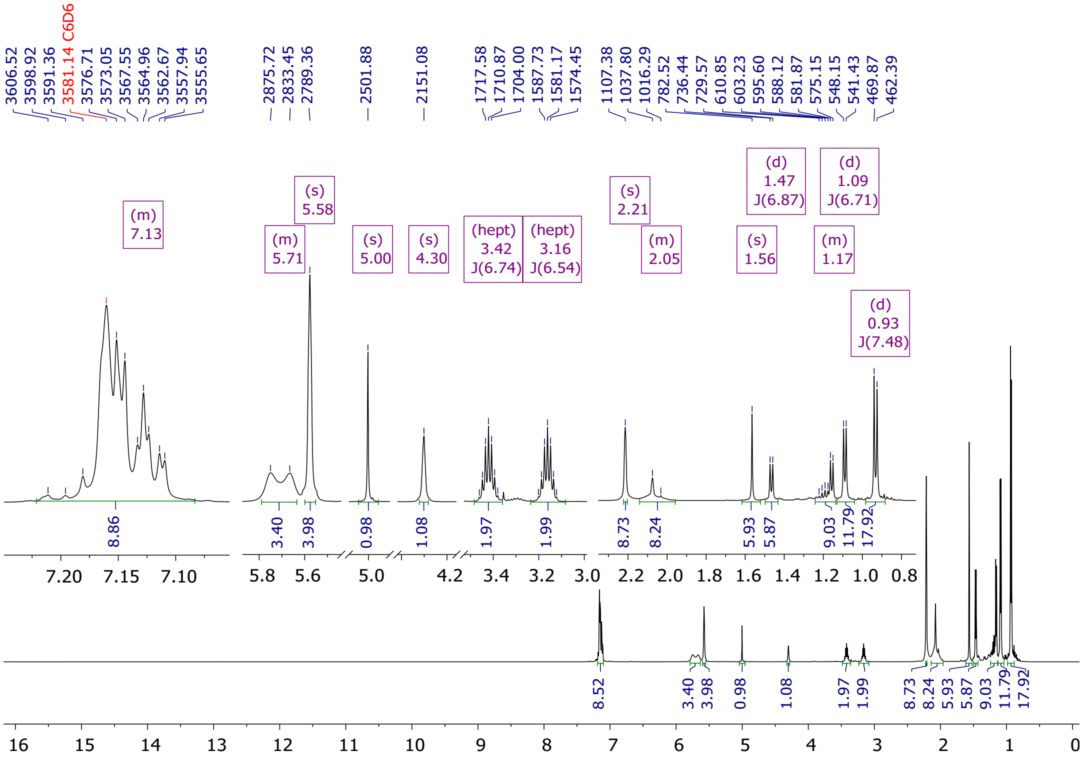

Figure S30. ^{1}H NMR spectrum of **4** (500 MHz, C_6D_6, 25°C).

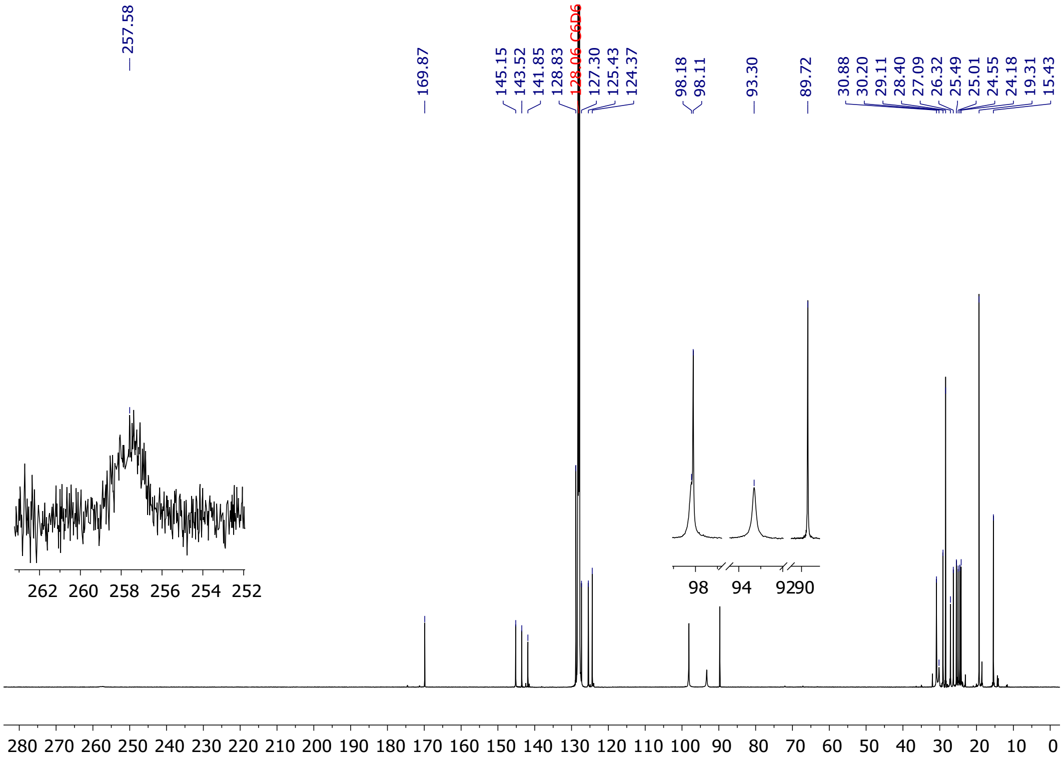

Figure S31. $^{13}C\{^{1}H\}$ NMR spectrum of **4** (126 MHz, C_6D_6, 25°C).

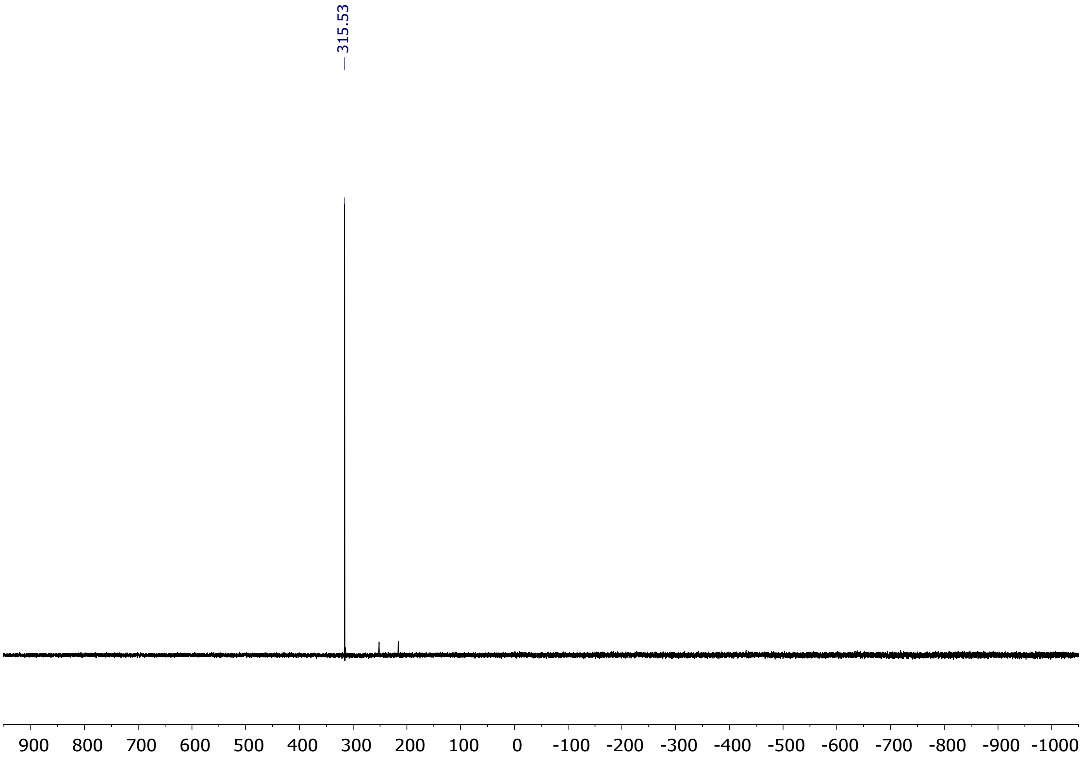

Figure S32. $^{31}P\{^1H\}$ NMR spectrum of **4** (202 MHz, C_6D_6, 25°C).

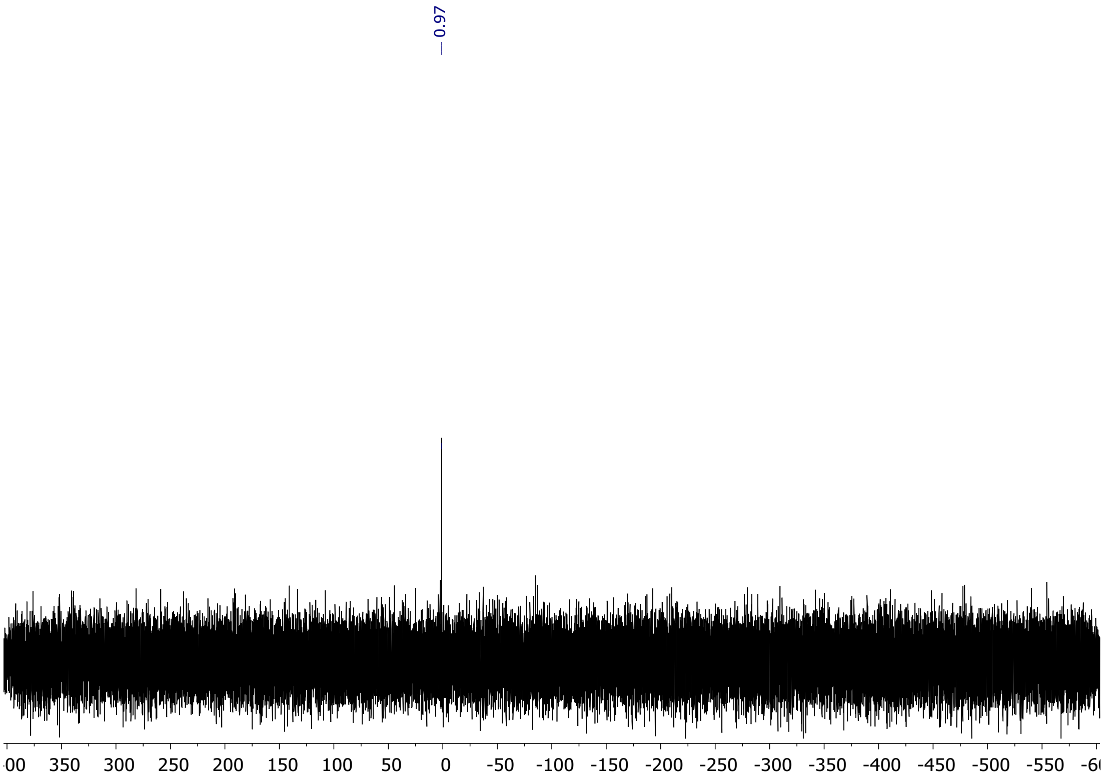

Figure S33. $^{29}Si\{1H\}$ NMR spectrum of **4** (99 MHz, C_6D_6, 25°C).

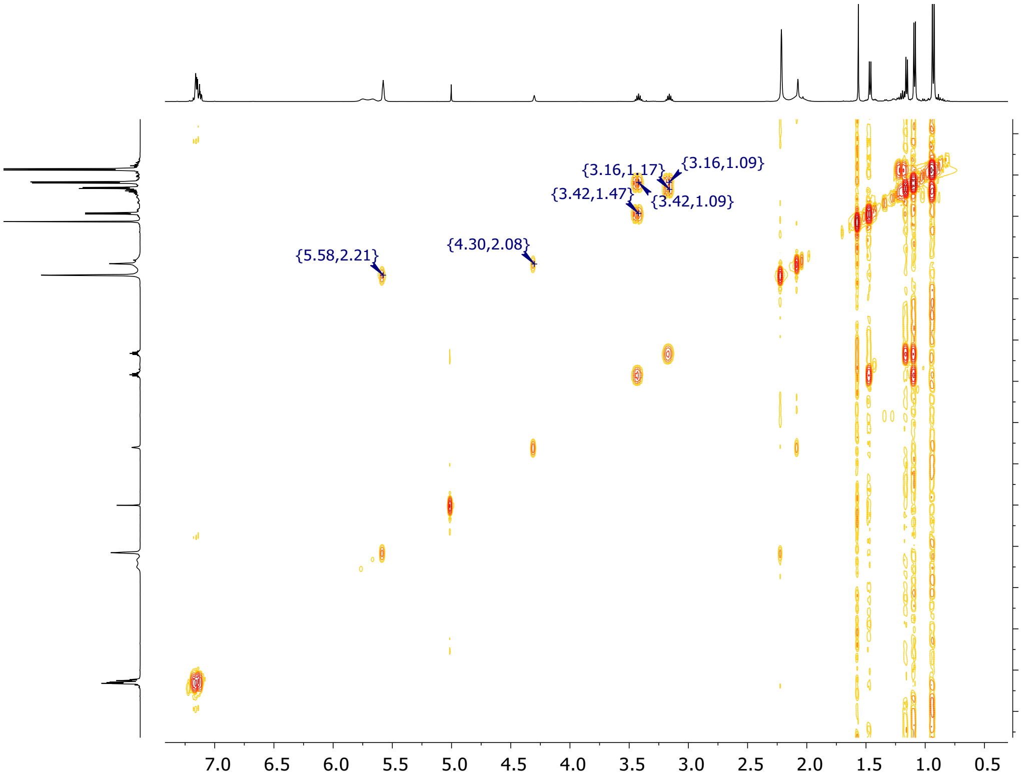

Figure S34. ^{1}H–^{1}H COSY NMR spectrum of **4** (500 MHz, C_6D_6, 25°C).

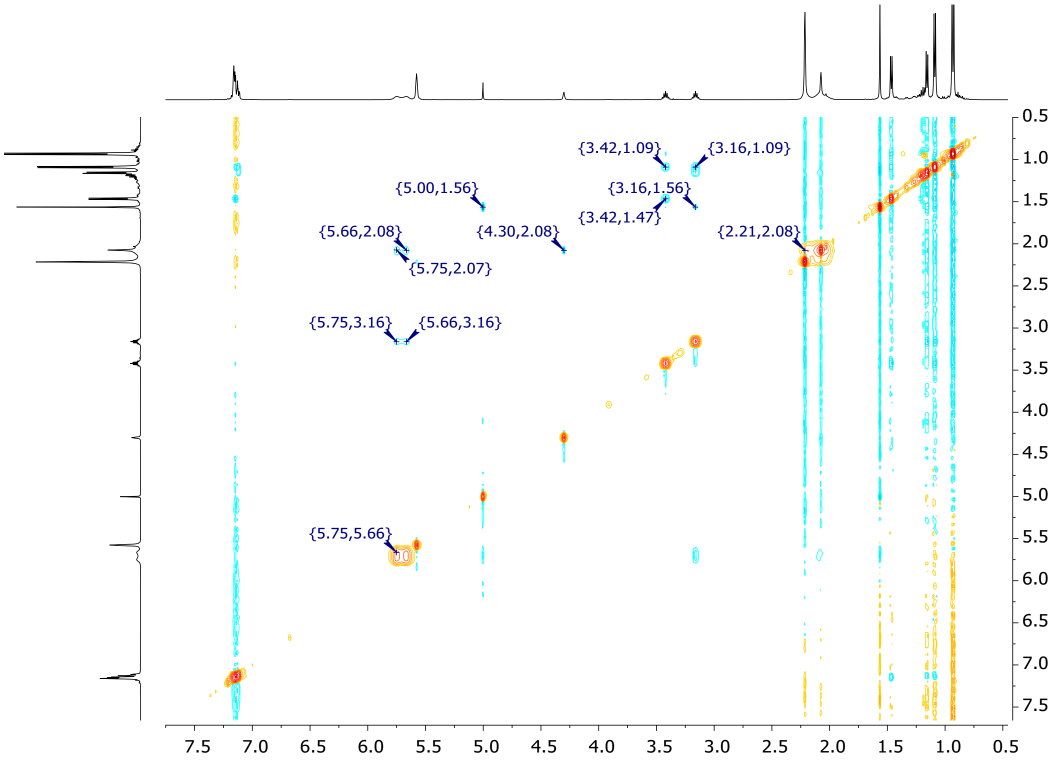

Figure S35. ^{1}H–^{1}H NOESY NMR spectrum of **4** (500 MHz, C_6D_6, 25°C).

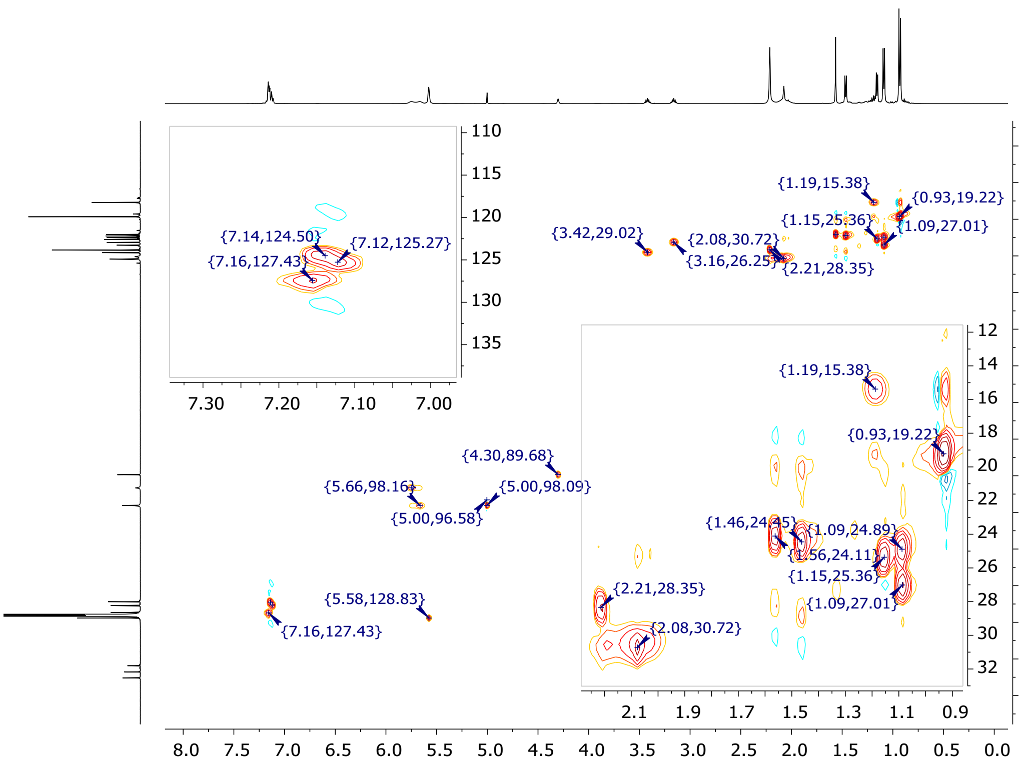

Figure S36. $^1H–^{13}C\{^1H\}$ HSQC NMR spectrum of **4** (500 & 126 MHz, C_6D_6, 25°C).

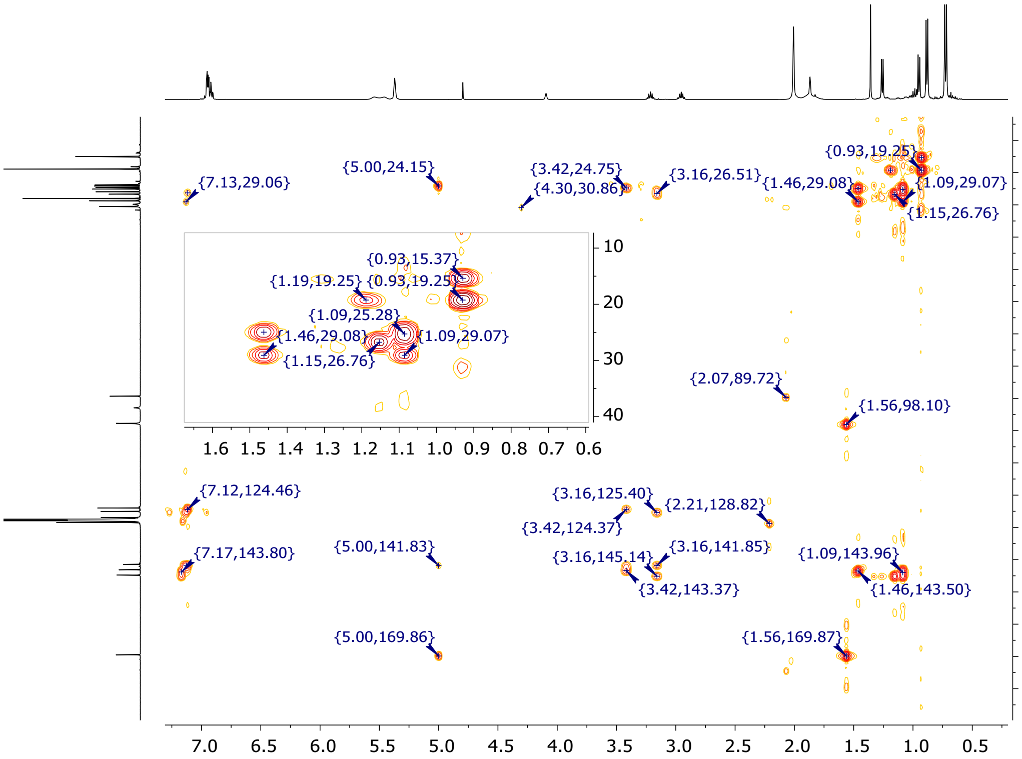

Figure S37. $^1H–^{13}C\{^1H\}$ HMBC NMR spectrum of **4** (500 & 126 MHz, C_6D_6, 25°C).

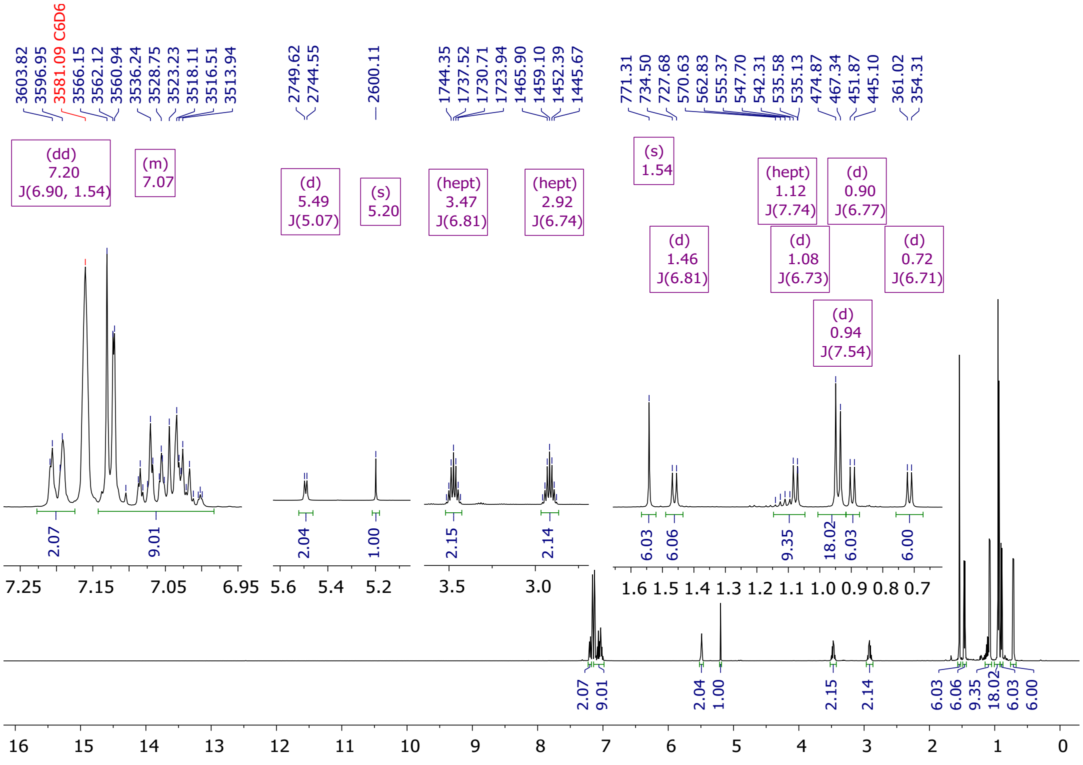

Figure S38. ^{1}H NMR spectrum of **5a** (500 MHz, C_6D_6, 25°C).

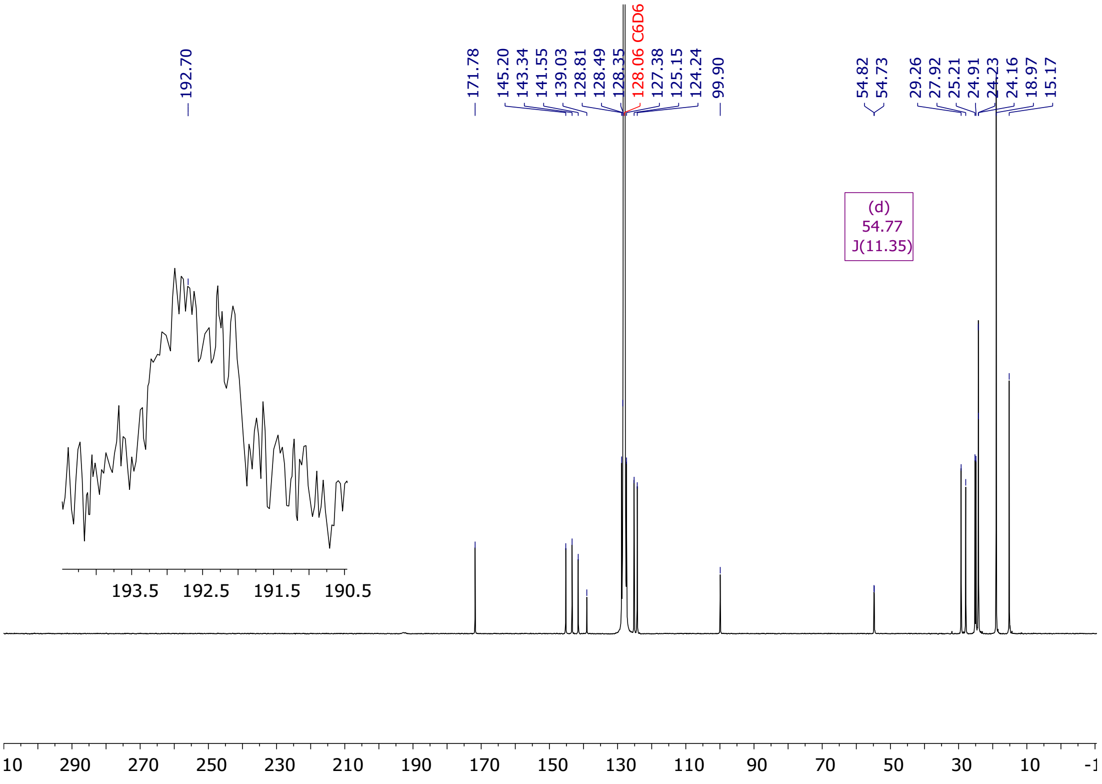

Figure S39. $^{13}C\{^{1}H\}$ NMR spectrum of **5a** (126 MHz, C_6D_6, 25°C).

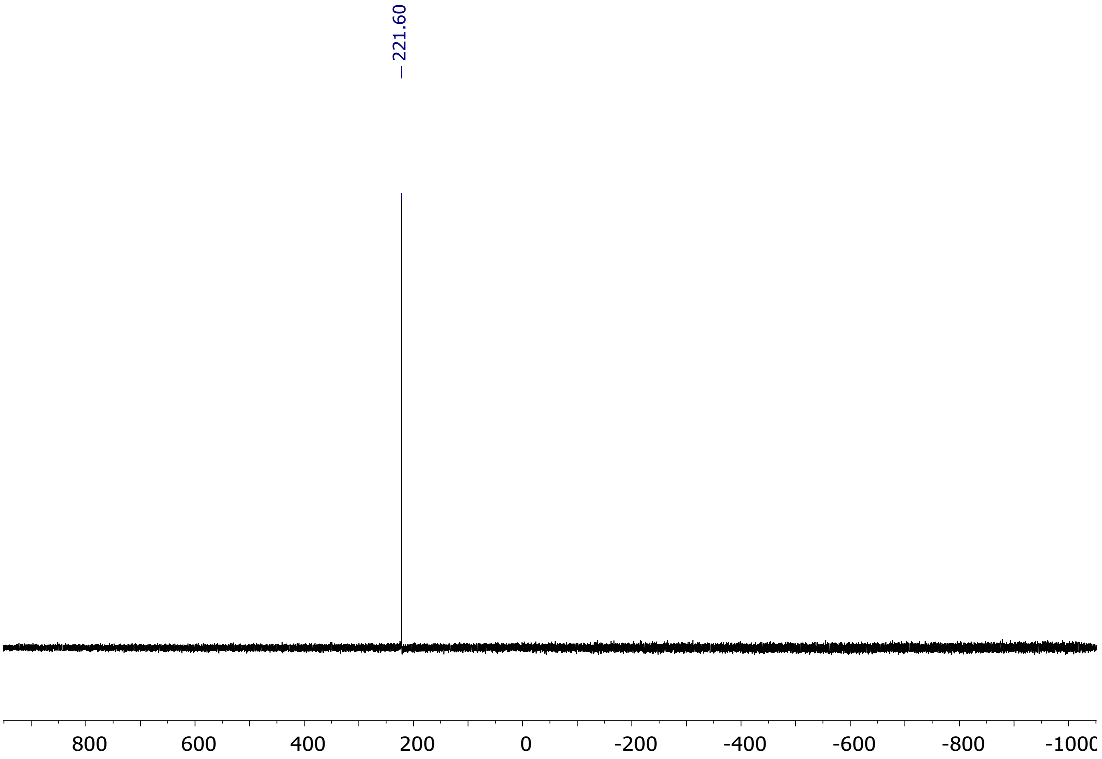

Figure S40. $^{31}P\{^{1}H\}$ NMR spectrum of **5a** (202 MHz, C_6D_6, 25°C).

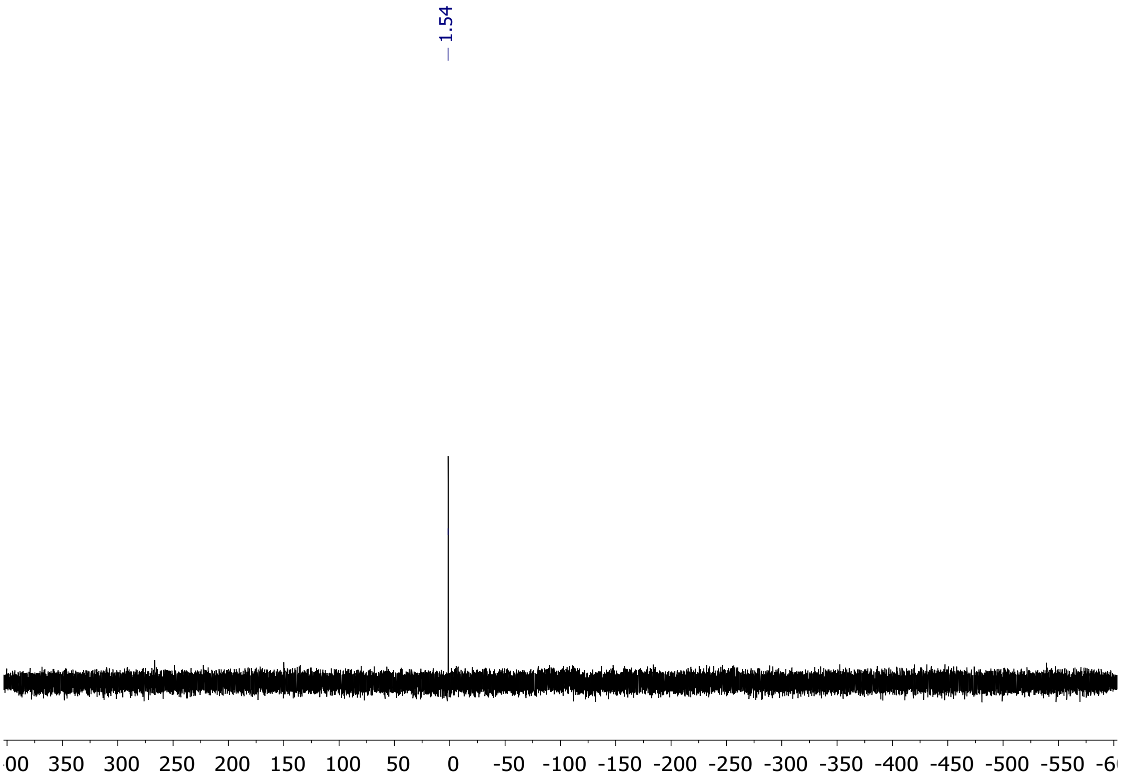

Figure S41. $^{29}Si\{1H\}$ NMR spectrum of **5a** (99 MHz, C_6D_6, 25°C).

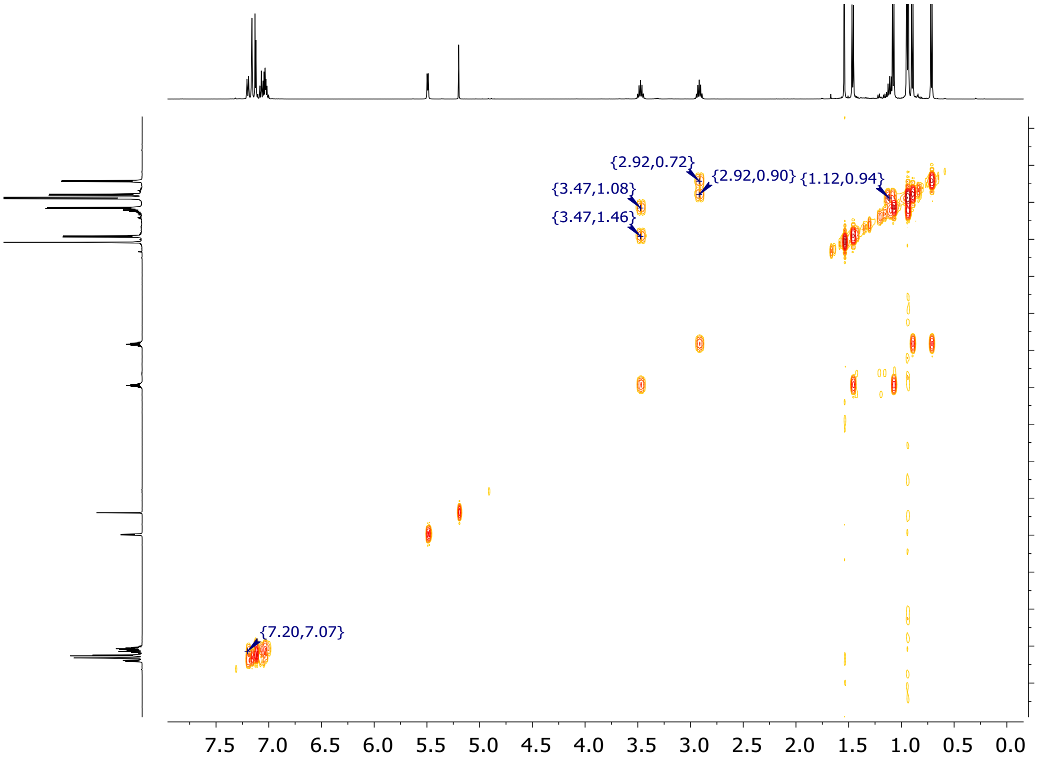

Figure S42. ^1H–^1H COSY NMR spectrum of **5a** (500 MHz, C_6D_6, 25°C).

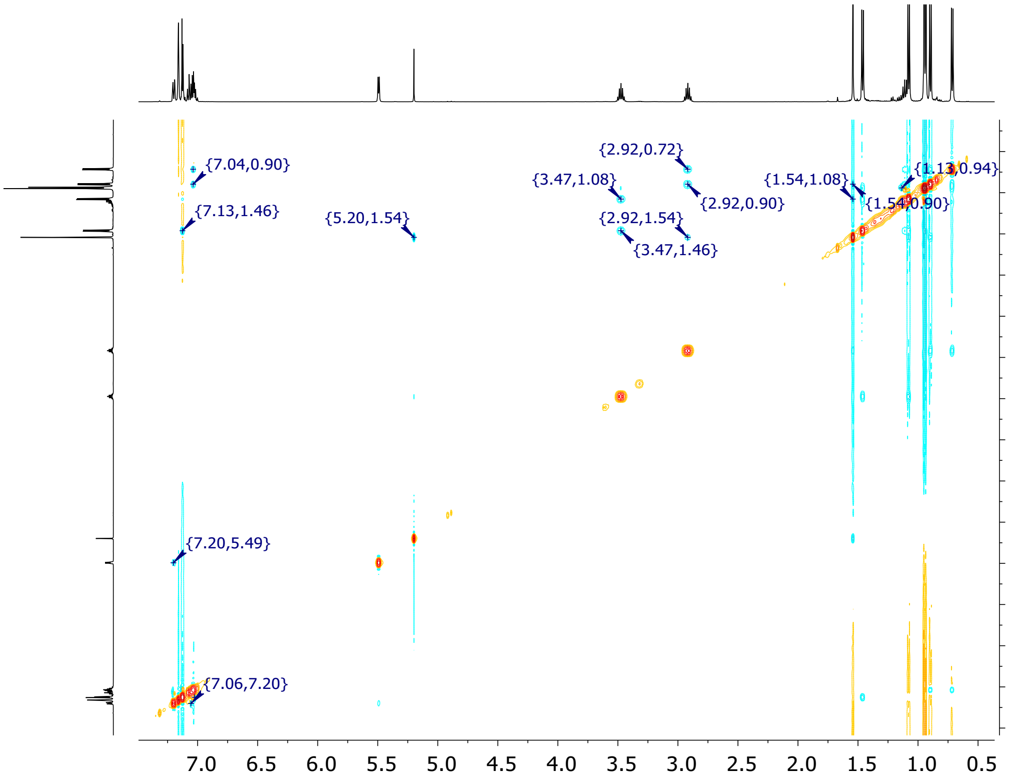

Figure S43. ^1H–^1H NOESY NMR spectrum of **5a** (500 MHz, C_6D_6, 25°C).

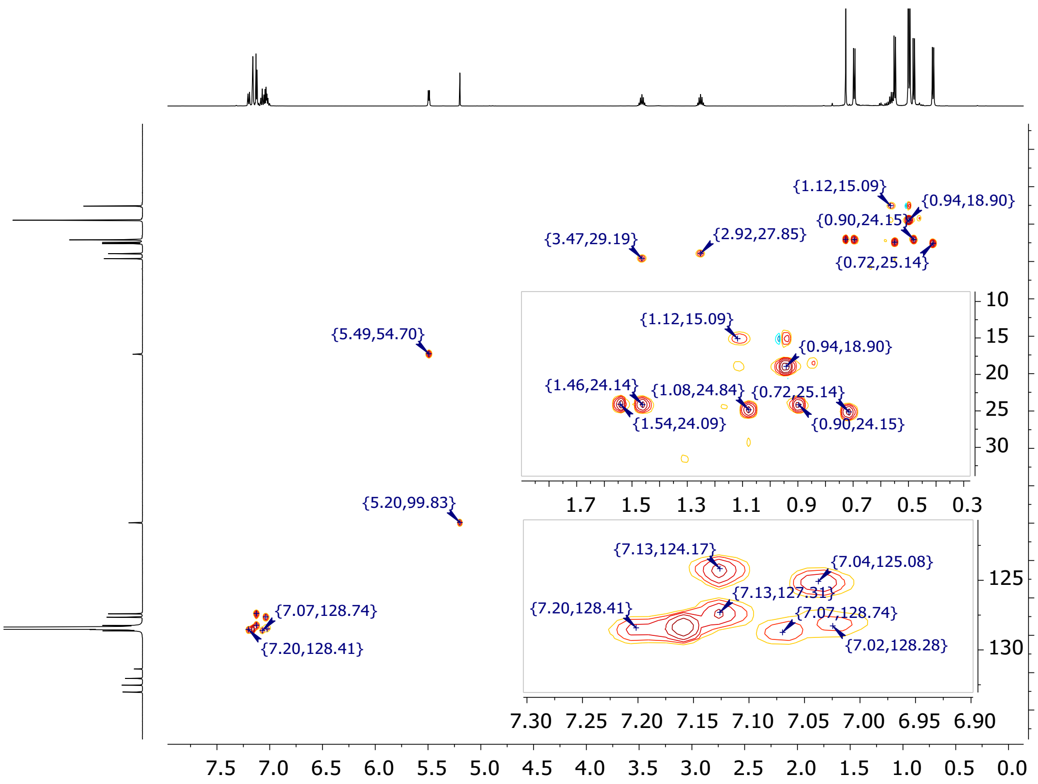

Figure S44. ^{1}H–$^{13}C\{^{1}H\}$ HSQC NMR spectrum of **5a** (500 & 126 MHz, C_6D_6, 25°C).

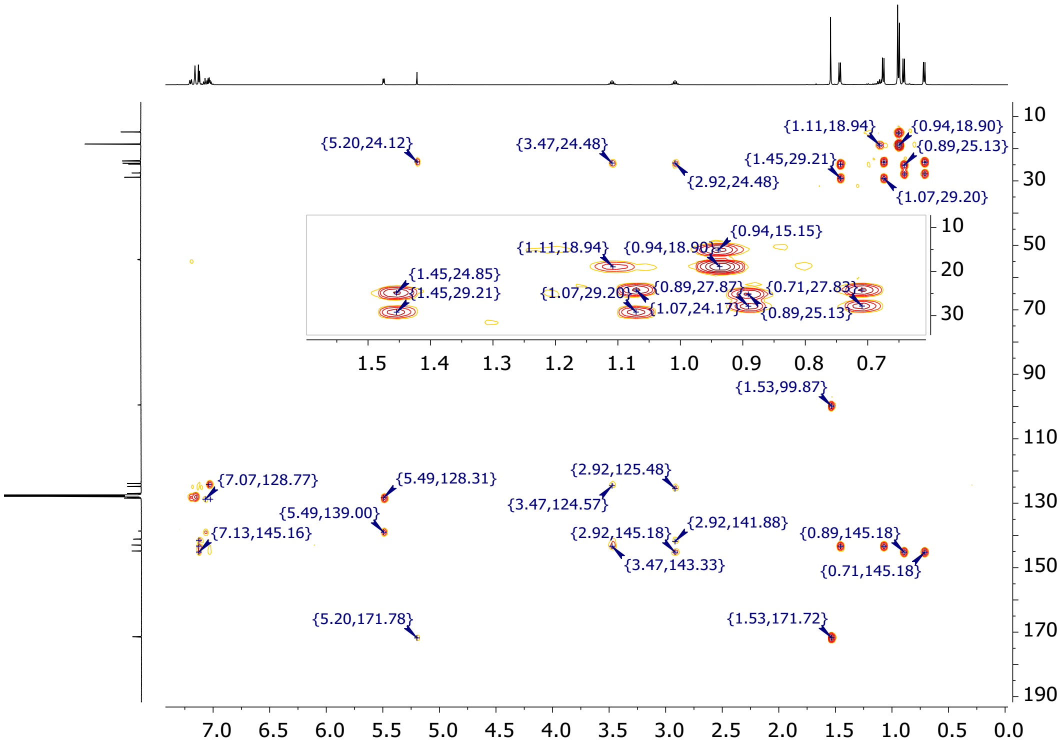

Figure S45. ^{1}H–$^{13}C\{^{1}H\}$ HMBC NMR spectrum of **5a** (500 & 126 MHz, C_6D_6, 25°C).

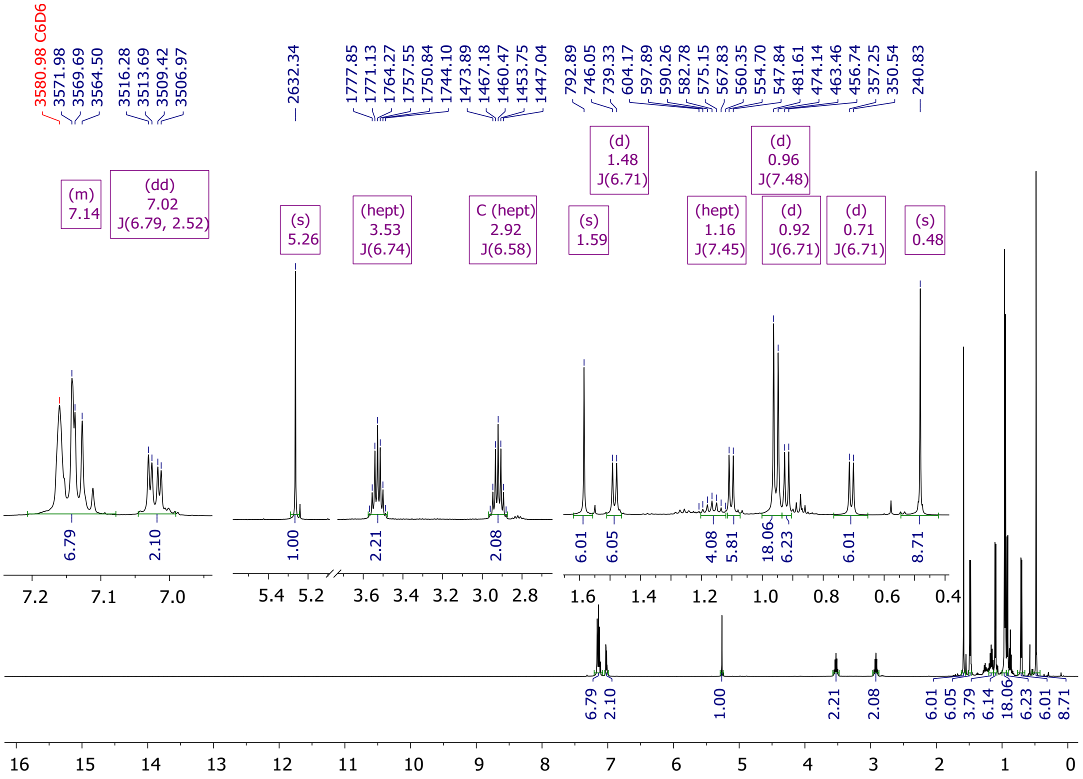

Figure S46. ^{1}H NMR spectrum of **5b** (500 MHz, C_6D_6, 25°C).

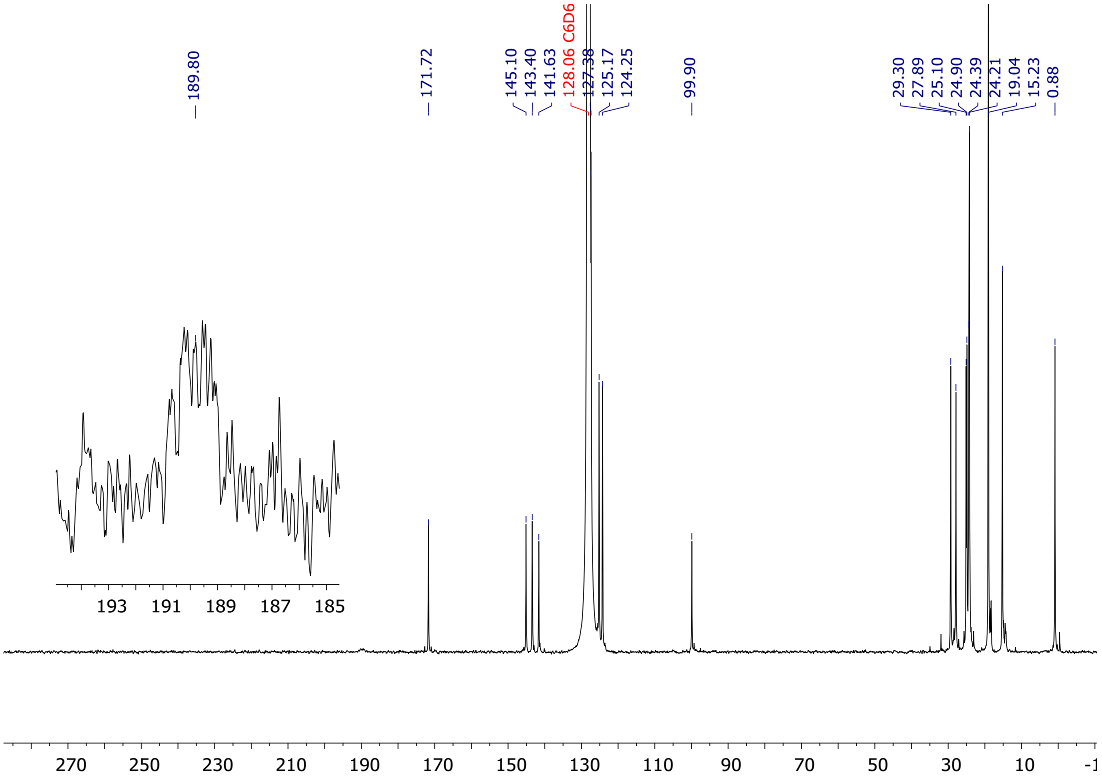

Figure S47. $^{13}C\{^{1}H\}$ NMR spectrum of **5b** (126 MHz, C_6D_6, 25°C).

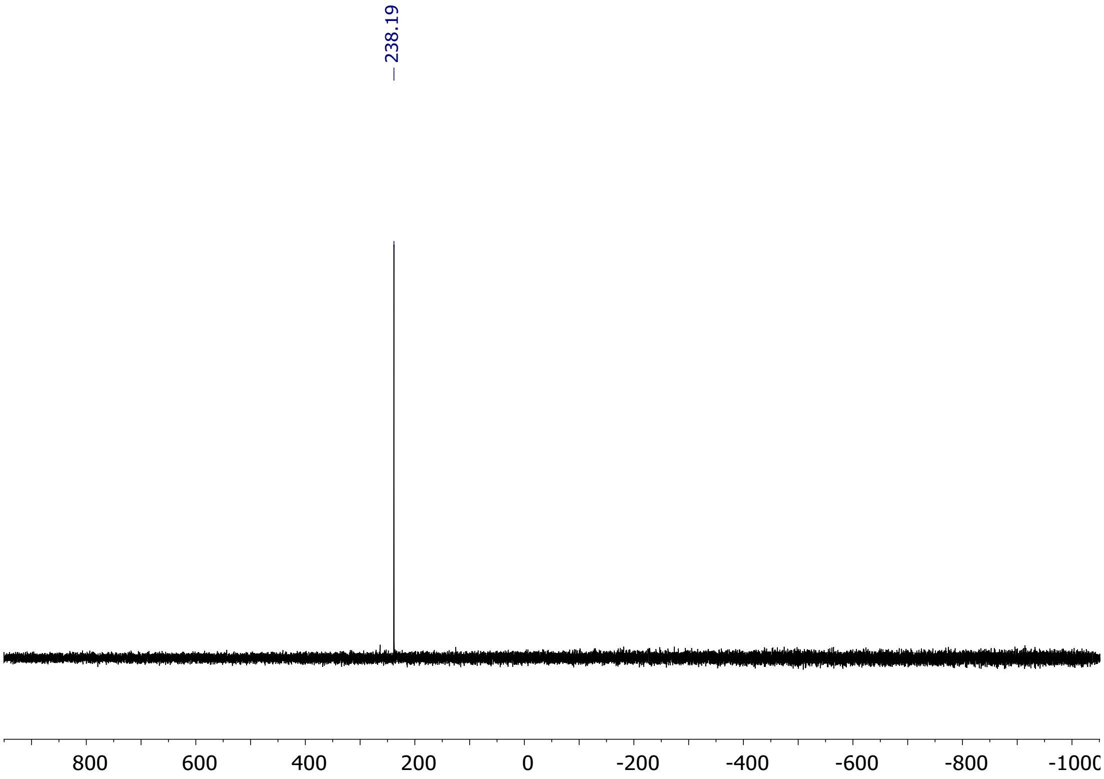

Figure S48. $^{31}P\{^{1}H\}$ NMR spectrum of **5b** (202 MHz, C_6D_6, 25°C).

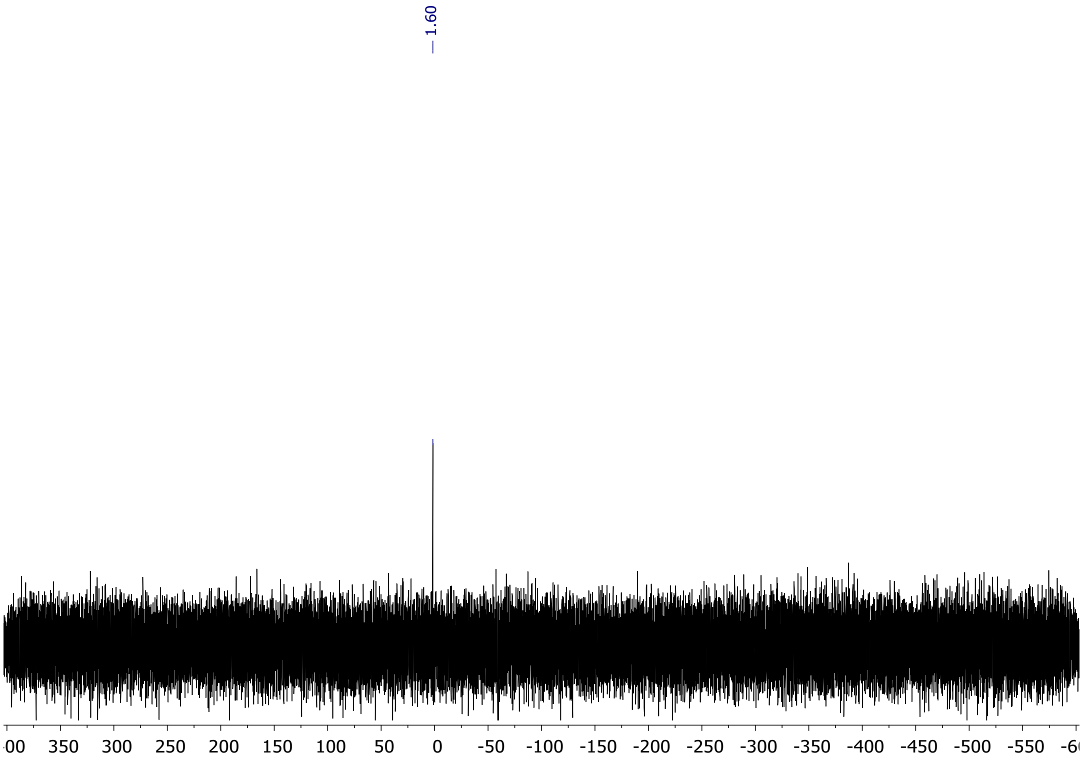

Figure S49. $^{29}Si\{1H\}$ NMR spectrum of **5b** (99 MHz, C_6D_6, 25°C).

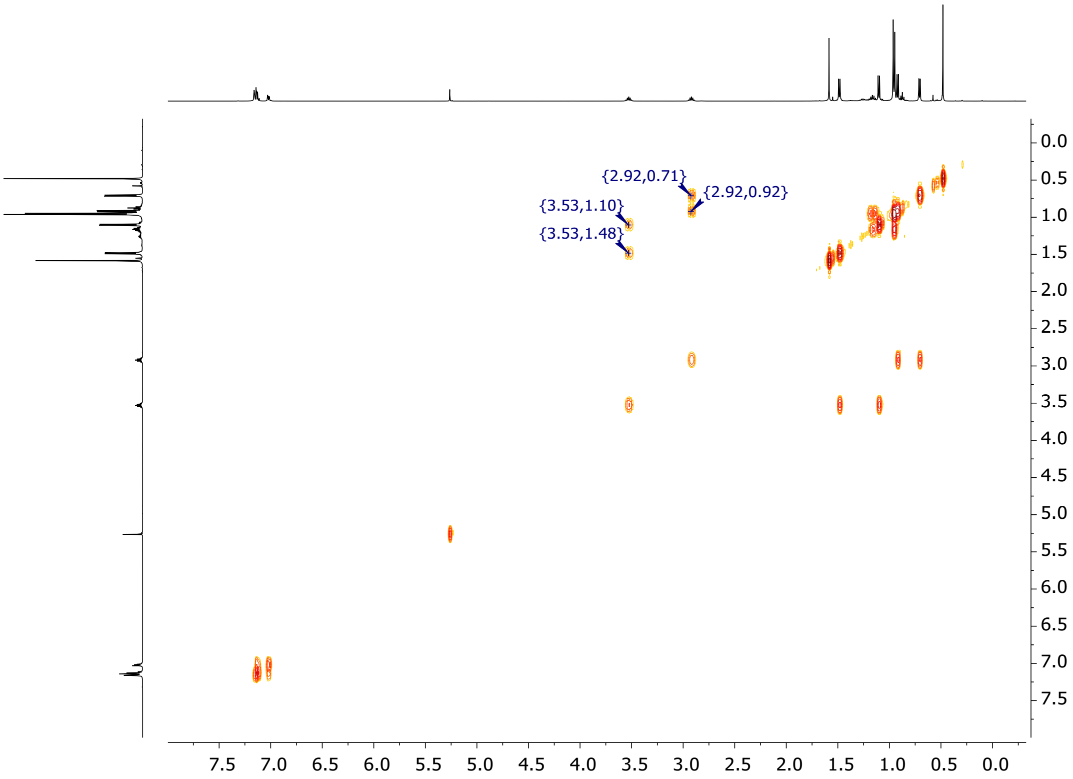

Figure S50. ^1H–^1H COSY NMR spectrum of **5b** (500 MHz, C_6D_6, 25°C).

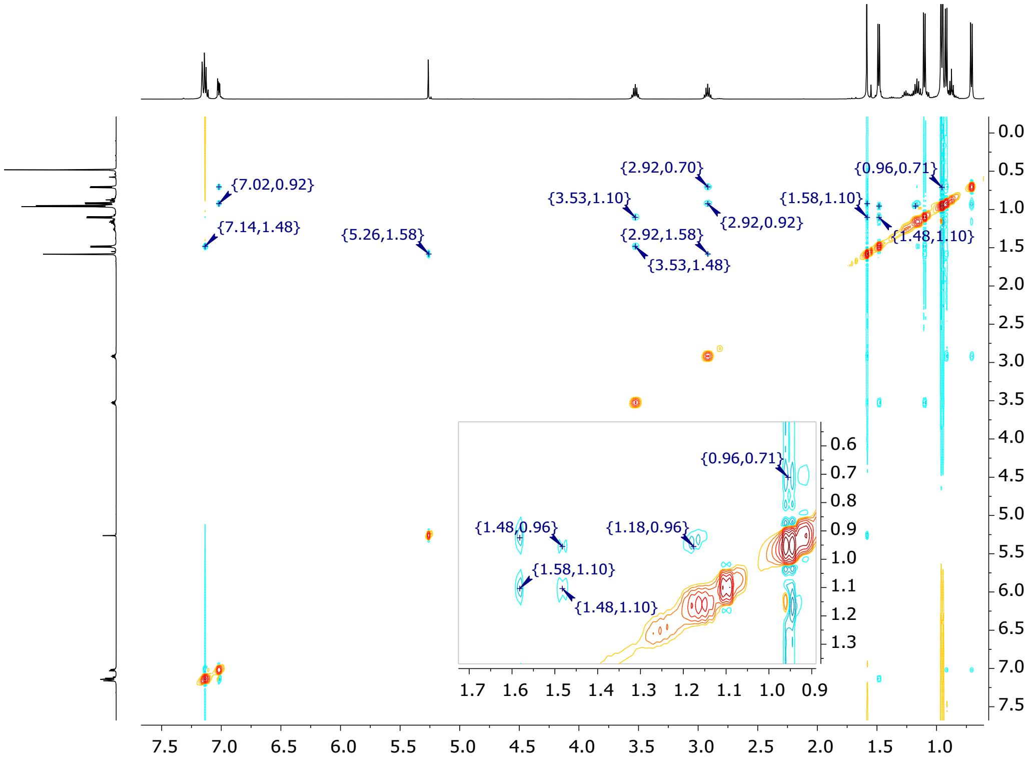

Figure S51. ^1H–^1H NOESY NMR spectrum of **5b** (500 MHz, C_6D_6, 25°C).

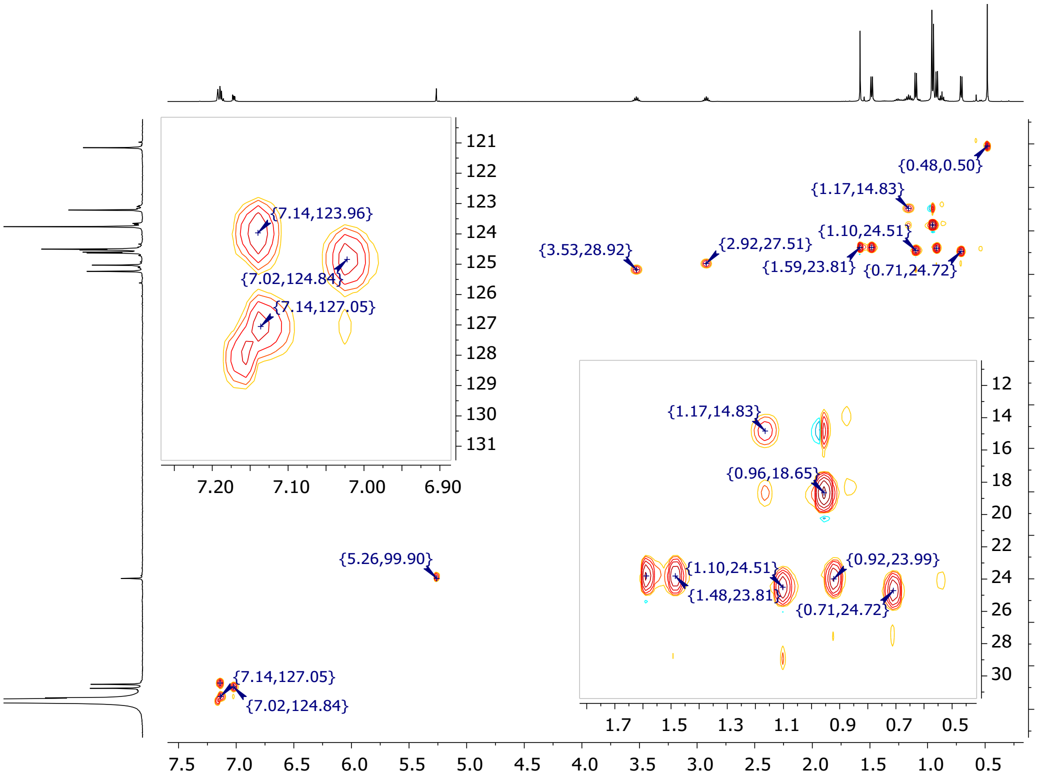

Figure S52. ^{1}H–$^{13}C\{^{1}H\}$ HSQC NMR spectrum of **5b** (500 & 126 MHz, C_6D_6, 25°C).

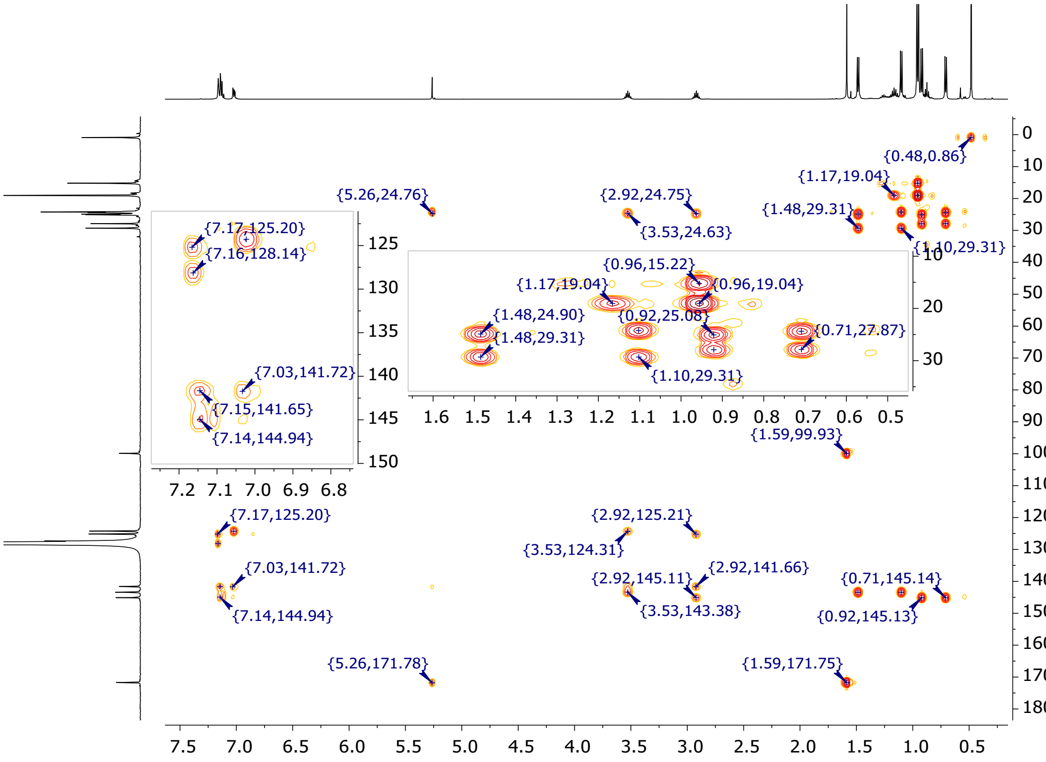

Figure S53. ^{1}H–$^{13}C\{^{1}H\}$ HMBC NMR spectrum of **5b** (500 & 126 MHz, C_6D_6, 25°C).

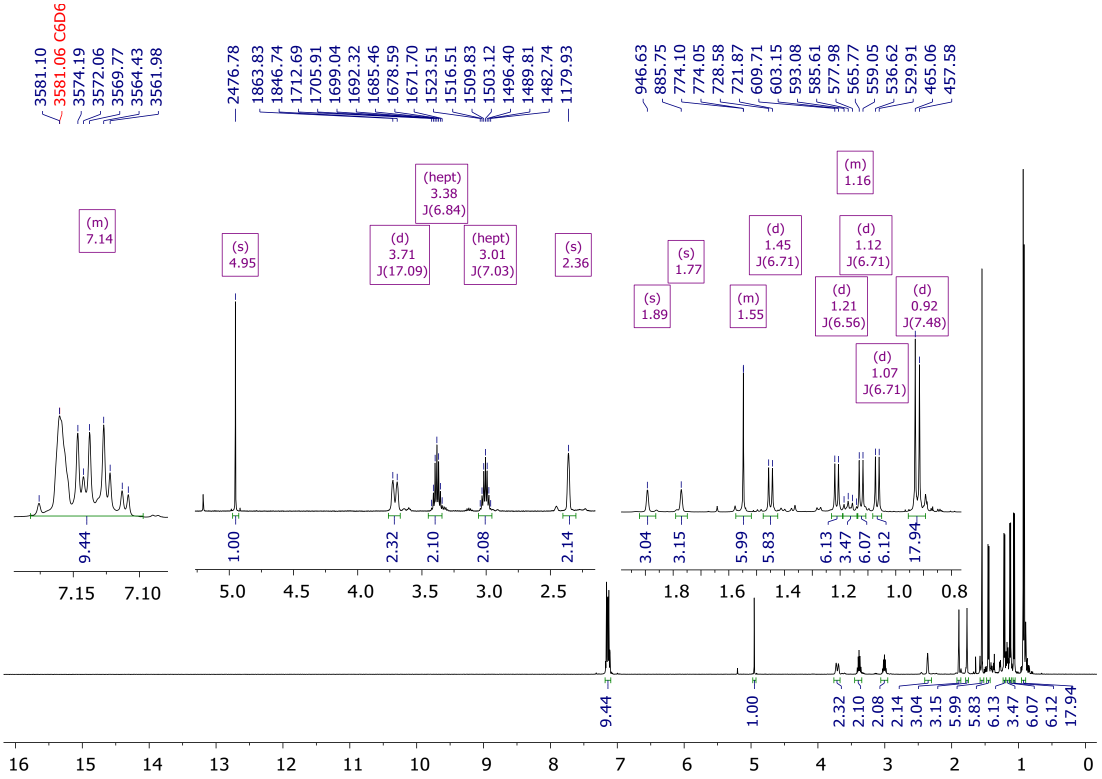

Figure S54. ^1H NMR spectrum of **6** (500 MHz, C_6D_6, 25°C).

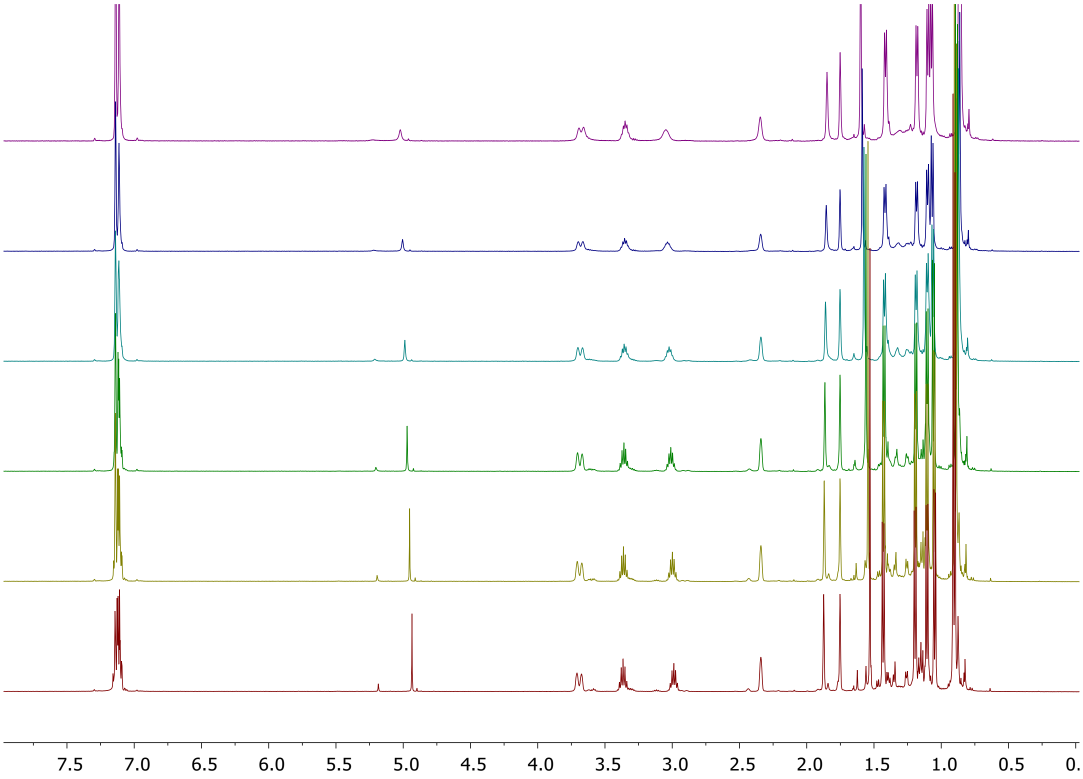

Figure S55. VT ^1H NMR spectra of **6** (500 MHz, C_6D_6, 25°C to 75°C).

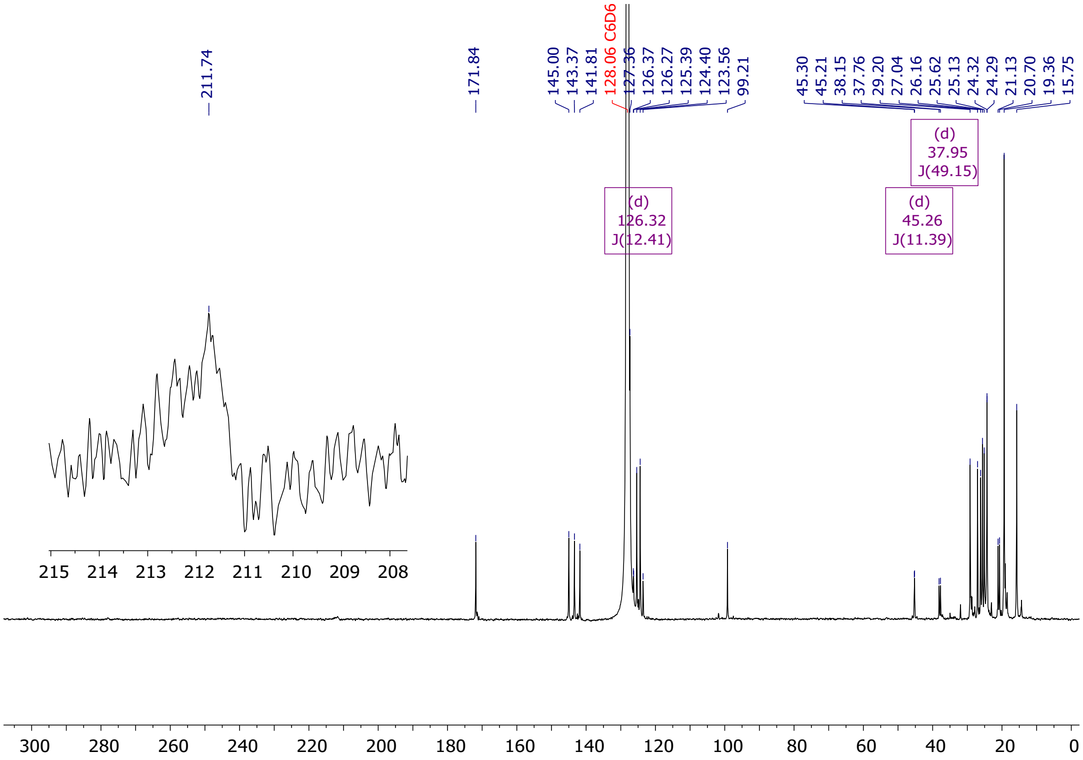

Figure S56. $^{13}C\{^{1}H\}$ NMR spectrum of **6** (126 MHz, C_6D_6, 25°C).

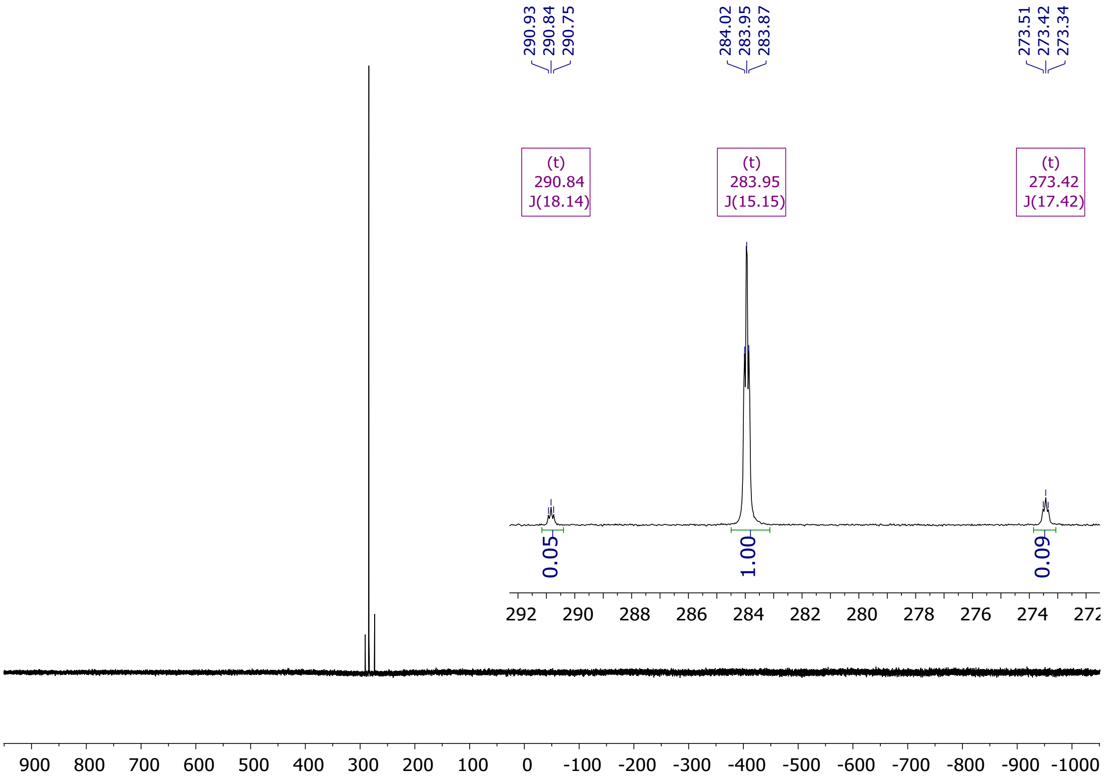

Figure S57. ^{31}P NMR spectrum of **6** (202 MHz, C_6D_6, 25°C).

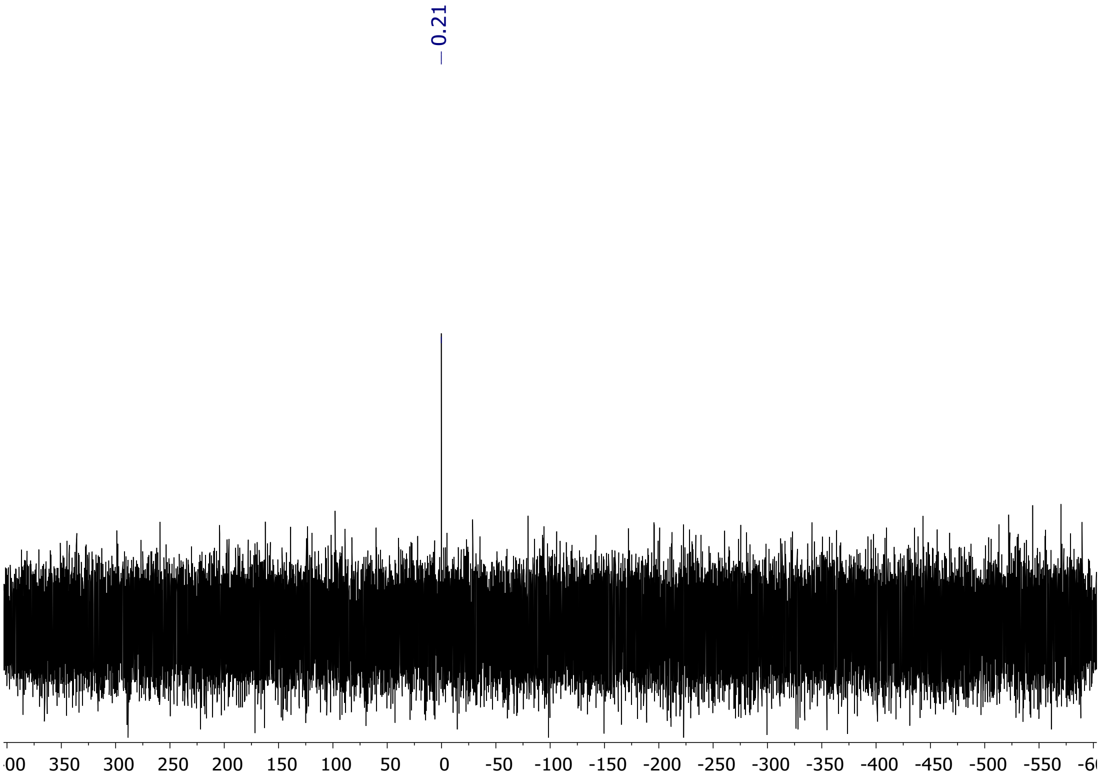

Figure S58. $^{29}Si\{^{1}H\}$ NMR spectrum of **6** (99 MHz, C_6D_6, 25°C).

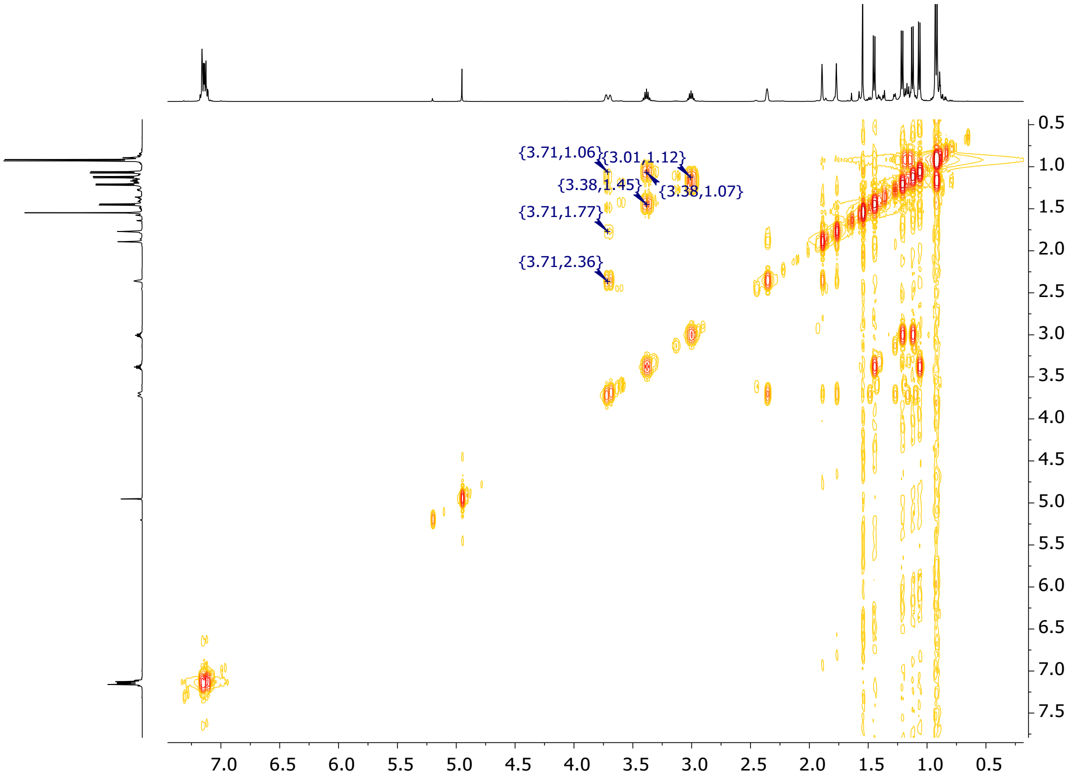

Figure S59. ^{1}H–^{1}H COSY NMR spectrum of **6** (500 MHz, C_6D_6, 25°C).

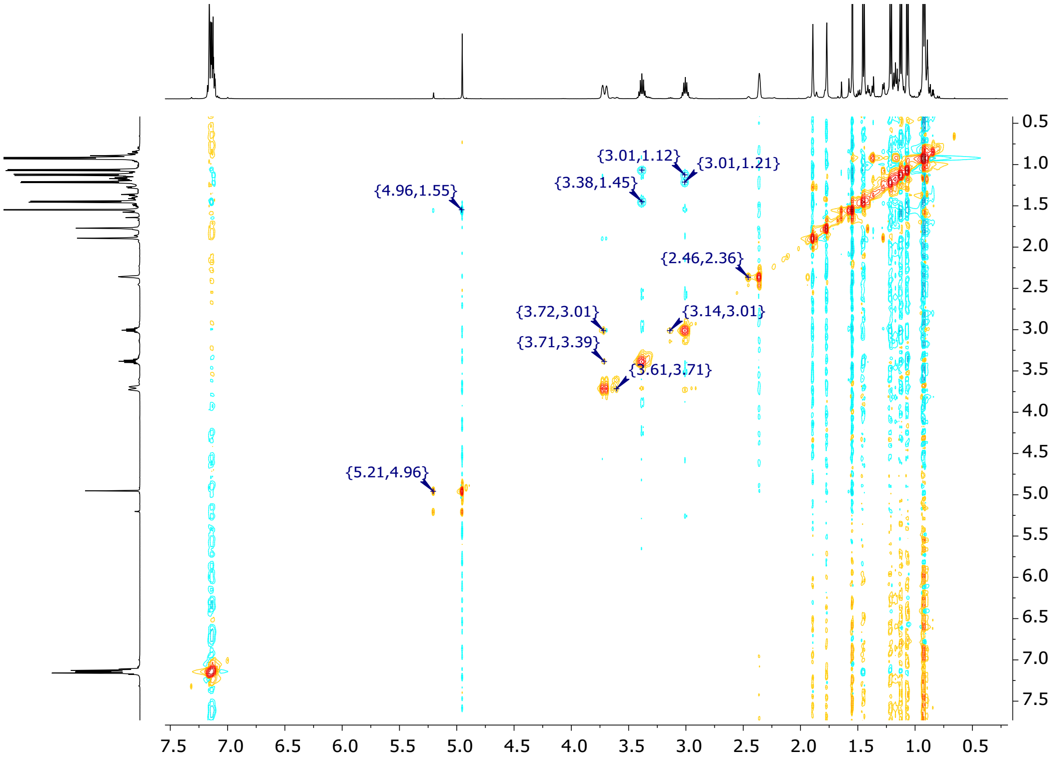

Figure S60. ^{1}H–^{1}H NOESY NMR spectrum of **6** (500 MHz, C_6D_6, 25°C).

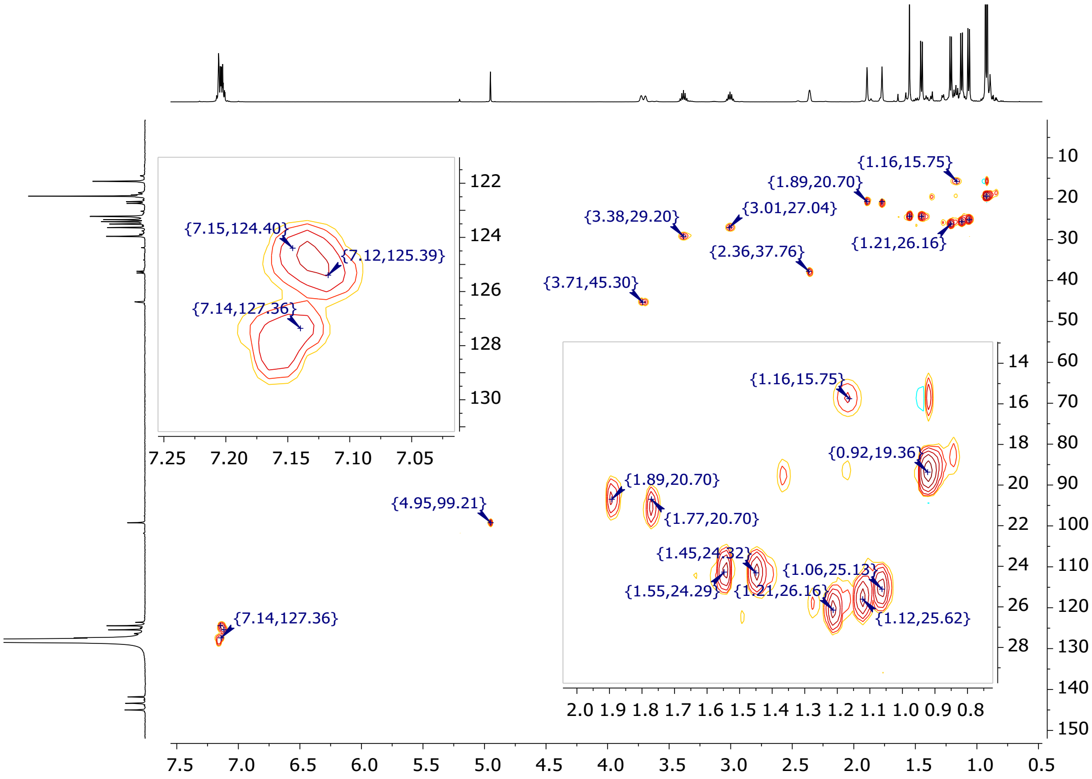

Figure S61. ^{1}H–$^{13}C\{^{1}H\}$ HSQC NMR spectrum of **6** (500 & 126 MHz, C_6D_6, 25°C).

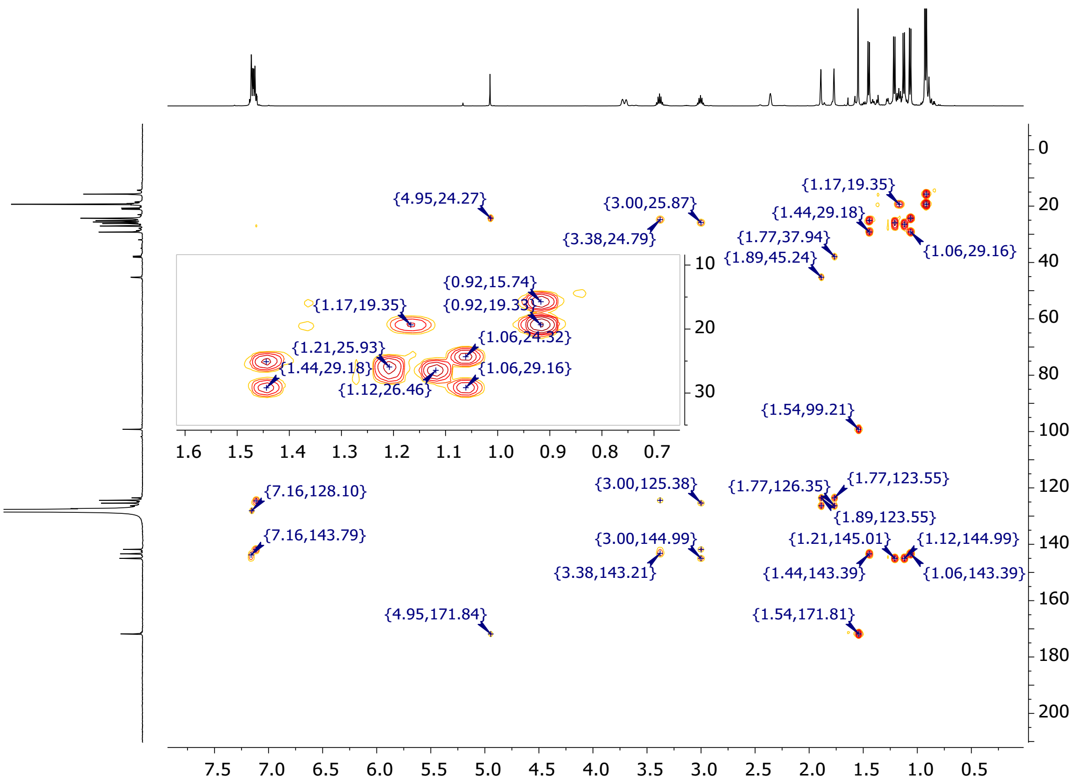

Figure S62. ^{1}H–$^{13}C\{^{1}H\}$ HMBC NMR spectrum of **6** (500 & 126 MHz, C_6D_6, 25°C).

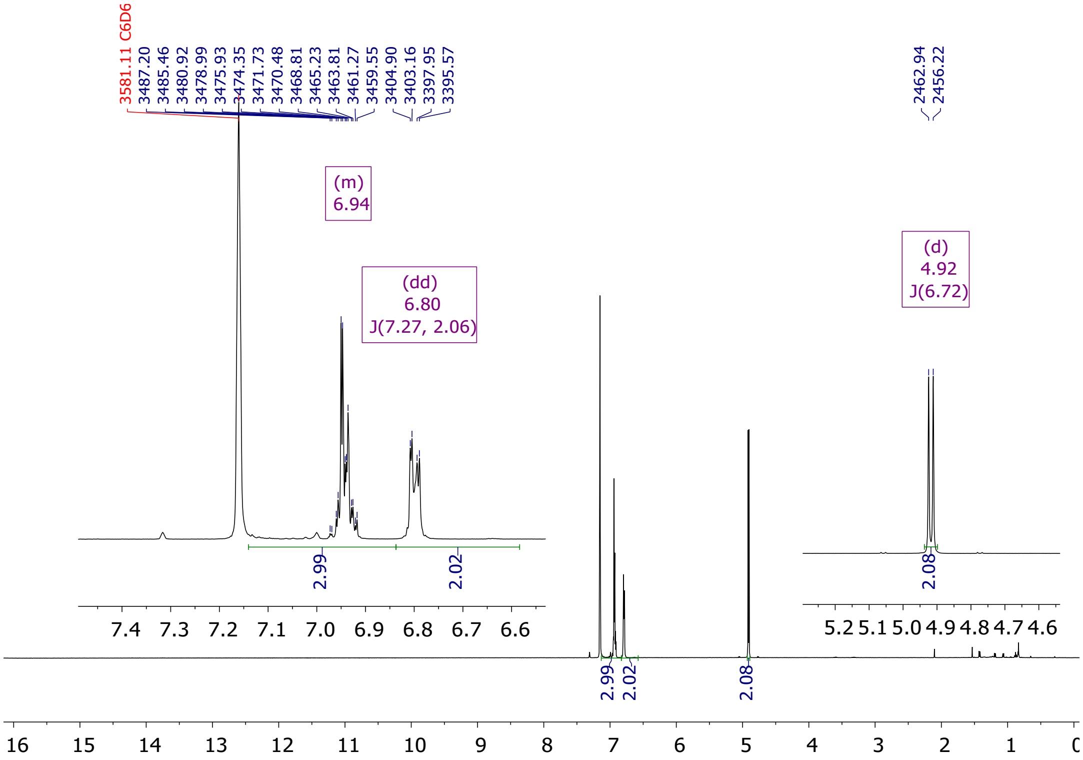

Figure S63. ^{1}H NMR spectrum of **7** (500 MHz, C_6D_6, 25°C).

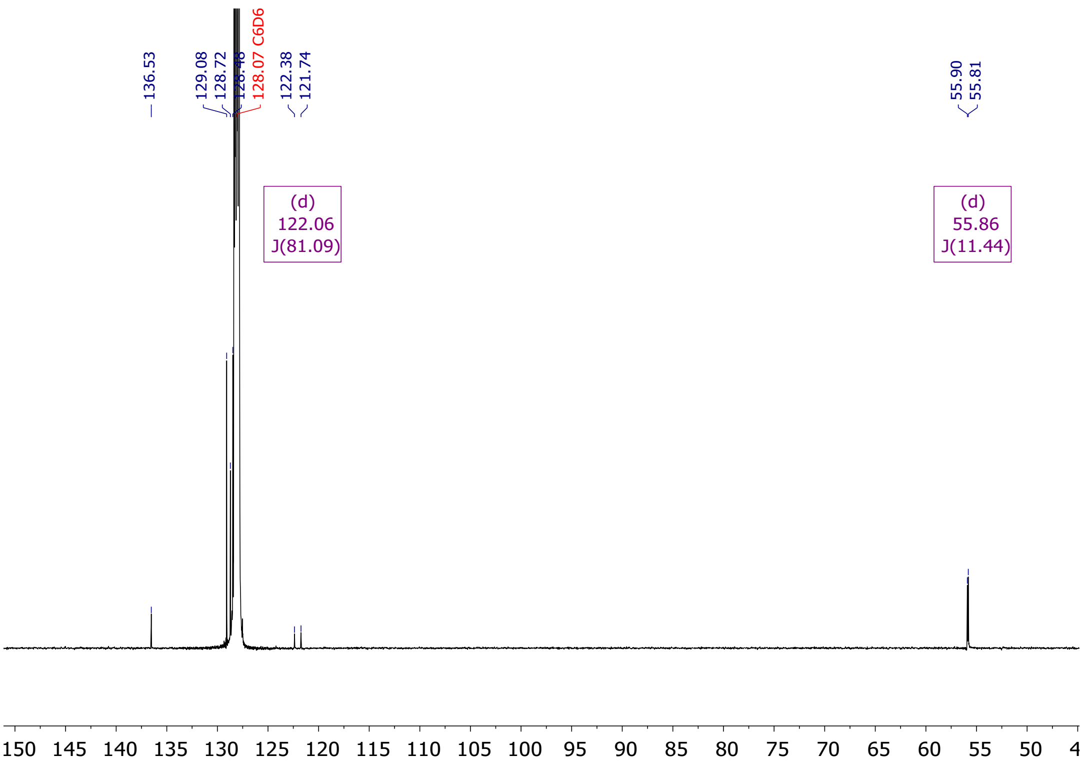

Figure S64. $^{13}C\{^1H\}$ NMR spectrum of **7** (126 MHz, C_6D_6, 25°C).

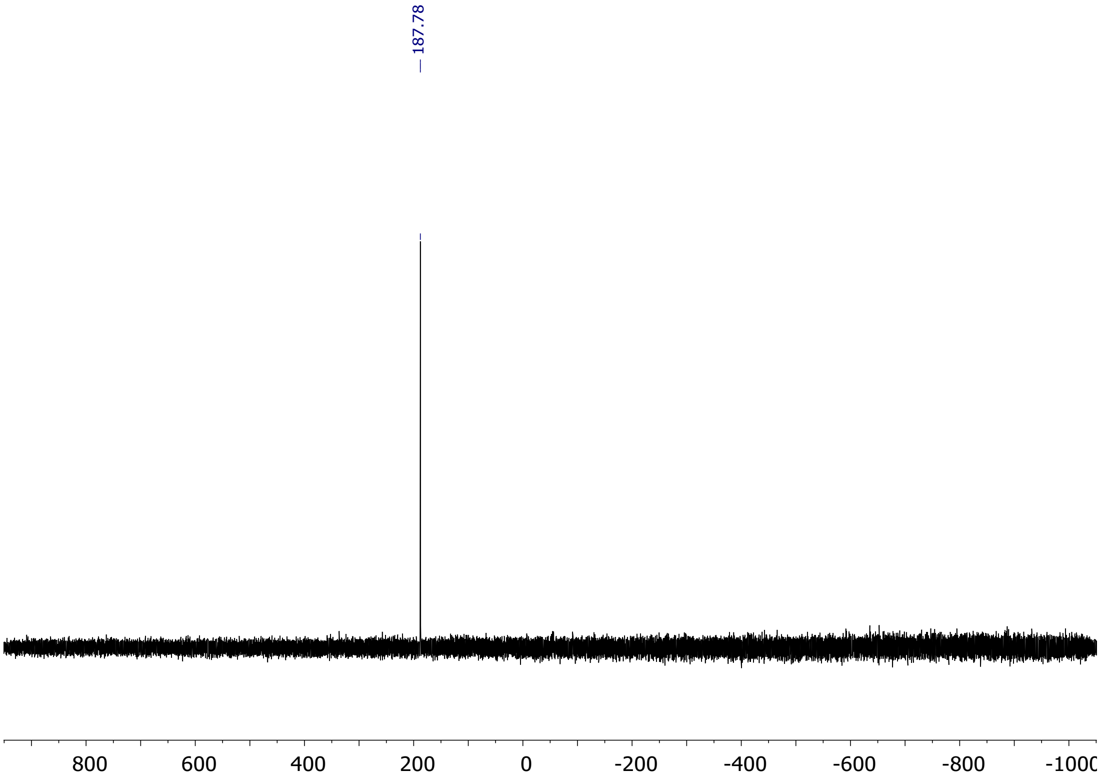

Figure S65. $^{31}P\{^1H\}$ NMR spectrum of **7** (202 MHz, C_6D_6, 25°C).

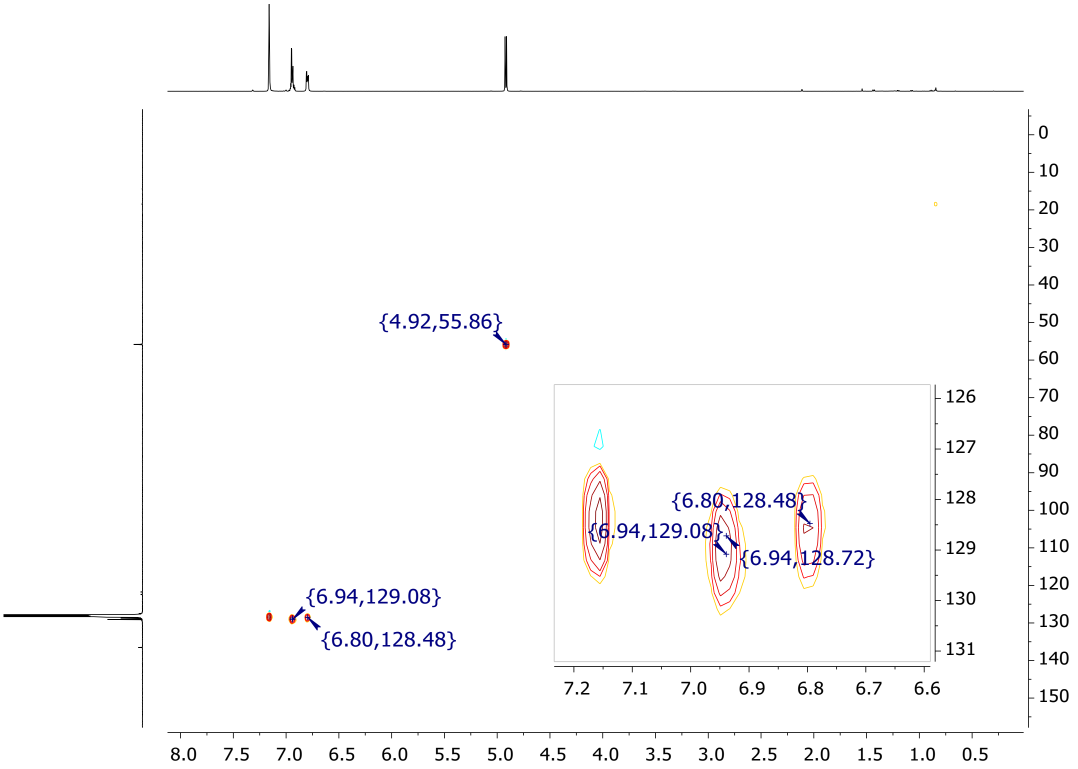

Figure S66. ^{1}H–$^{13}C\{^{1}H\}$ HSQC NMR spectrum of **7** (500 & 126 MHz, C_6D_6, 25°C).

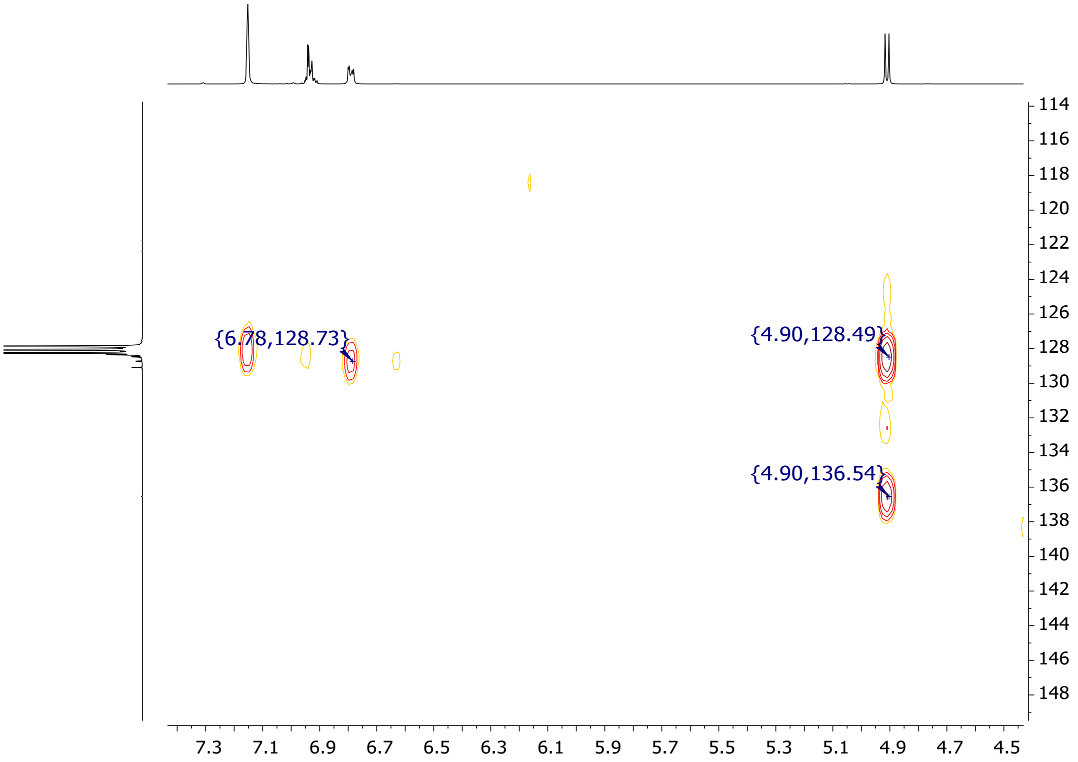

Figure S67. ^{1}H–$^{13}C\{^{1}H\}$ HMBC NMR spectrum of **7** (500 & 126 MHz, C_6D_6, 25°C).

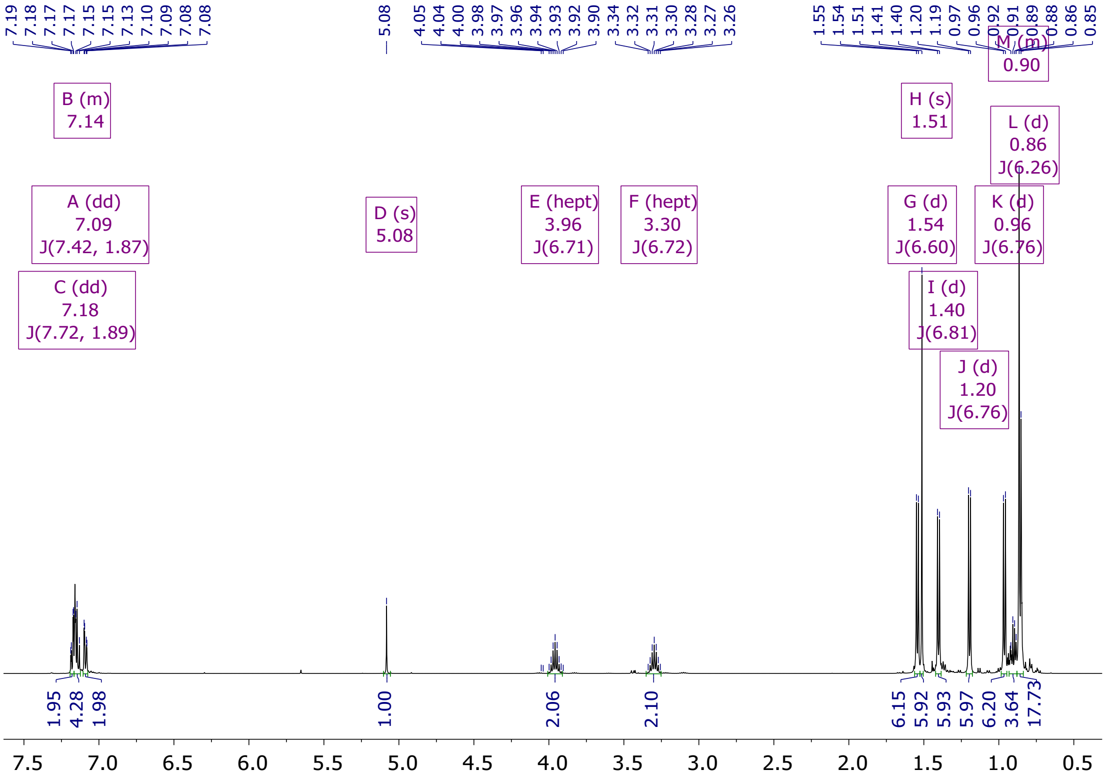

Figure S68. ^{1}H NMR spectrum of Al(^{Dipp}NacNac)(OSi^{i}Pr$_3$)I (500 MHz, C_6D_6, 25°C).

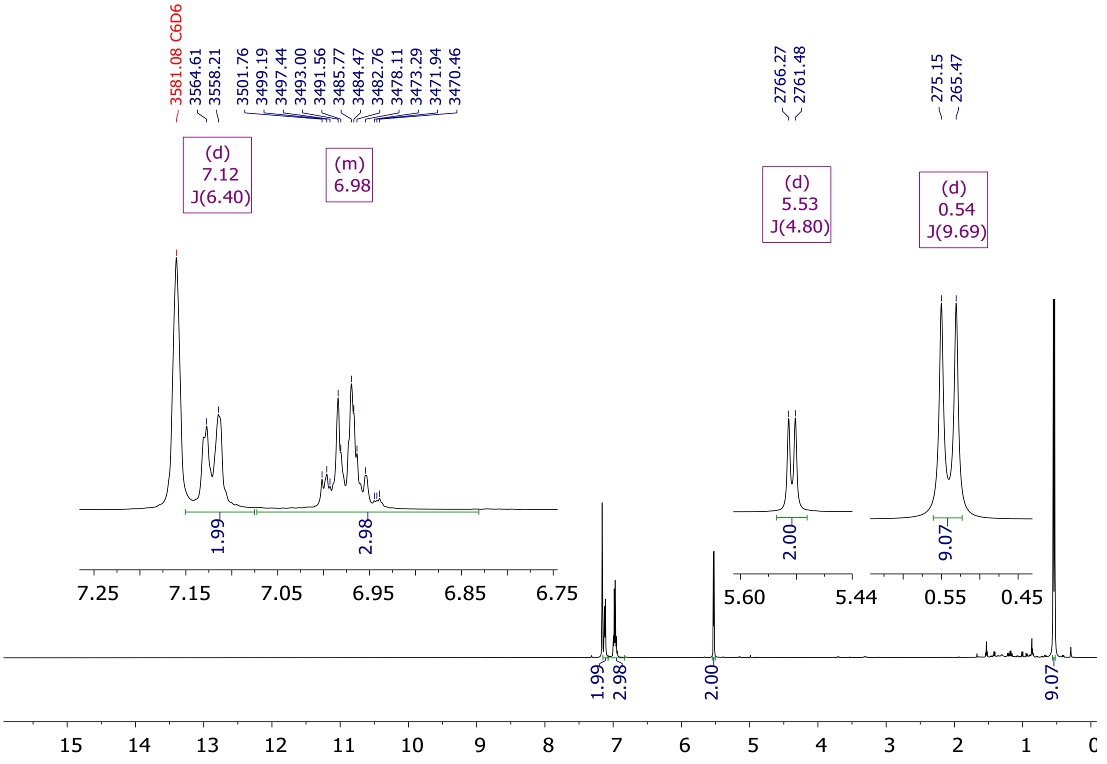

Figure S69. ^{1}H NMR spectrum of **8** (500 MHz, C_6D_6, 25°C).

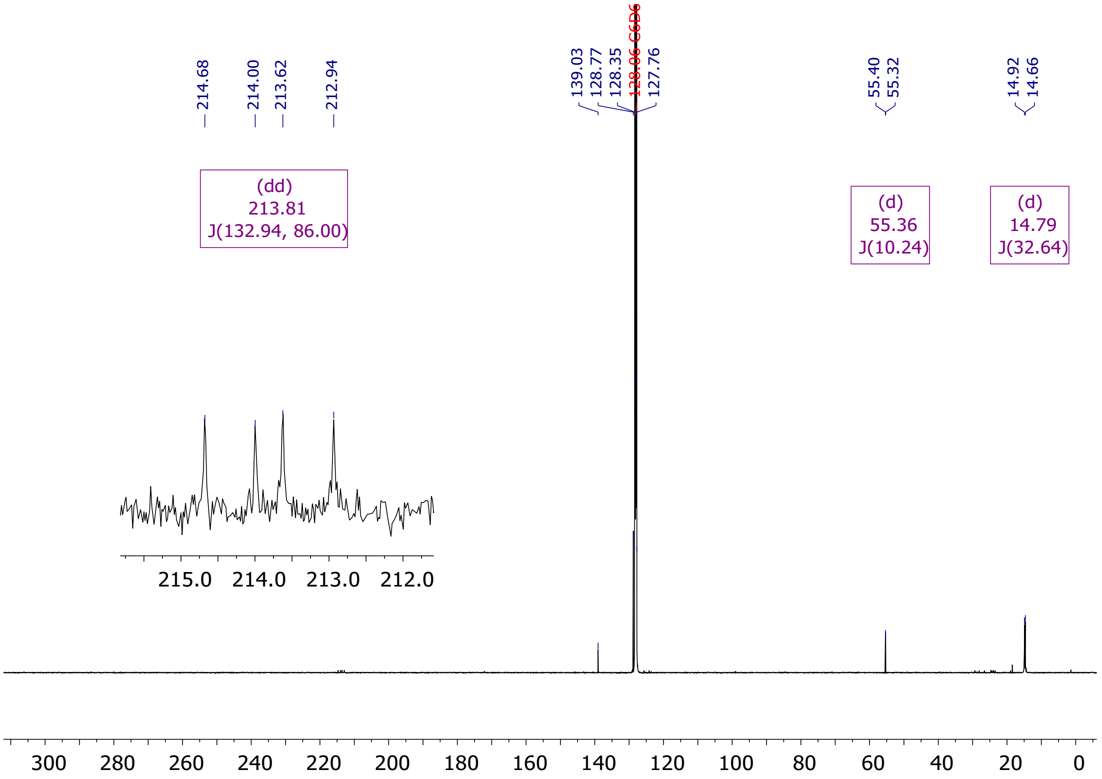

Figure S70. $^{13}C\{^1H\}$ NMR spectrum of **8** (126 MHz, C_6D_6, 25°C).

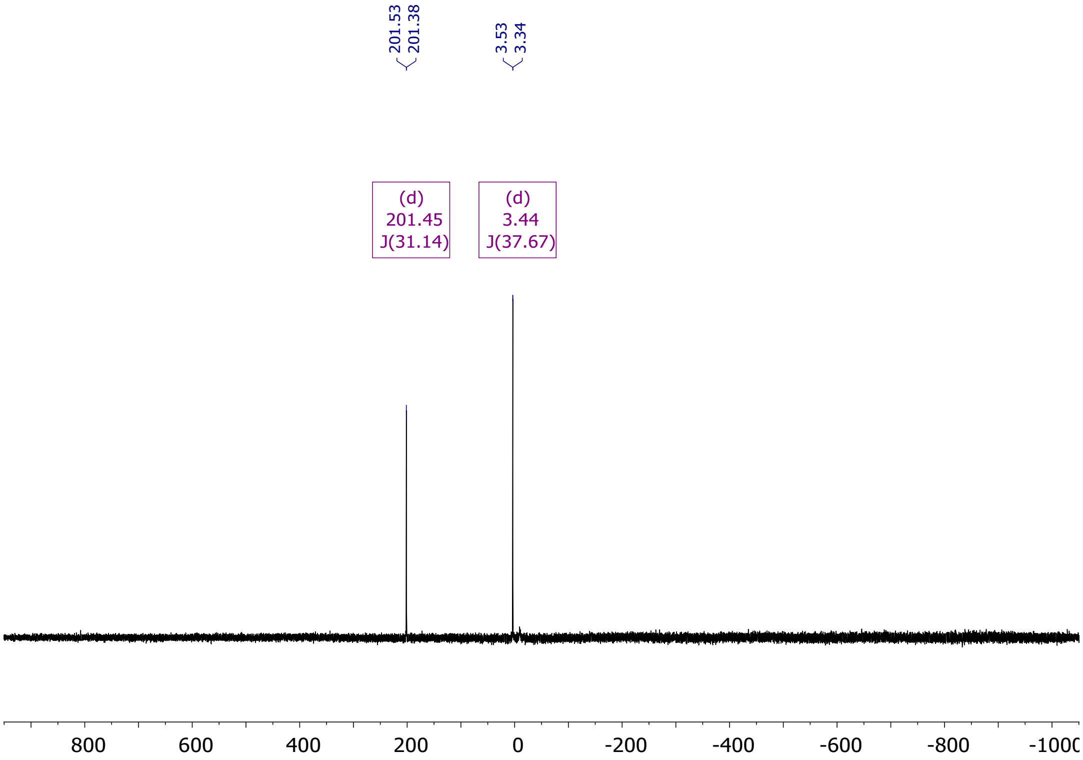

Figure S71. $^{31}P\{^1H\}$ NMR spectrum of **8** (202 MHz, C_6D_6, 25°C).

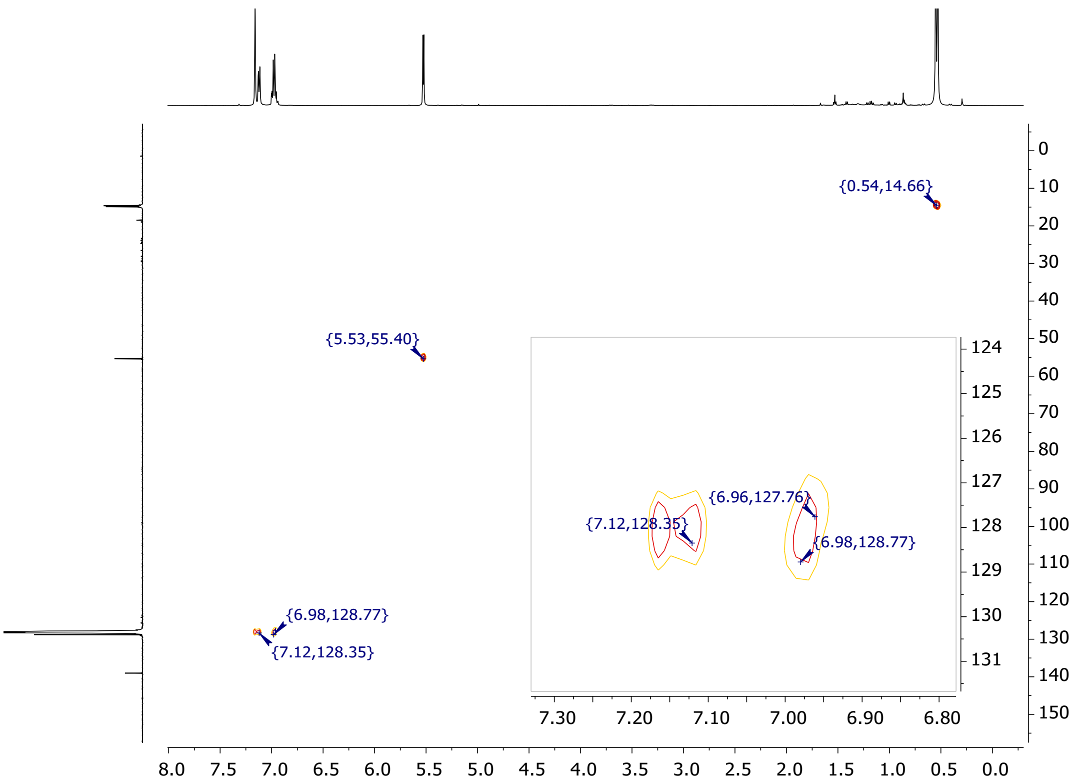

Figure S72. ^{1}H–$^{13}C\{^{1}H\}$ HSQC NMR spectrum of **8** (500 & 126 MHz, C_6D_6, 25°C).

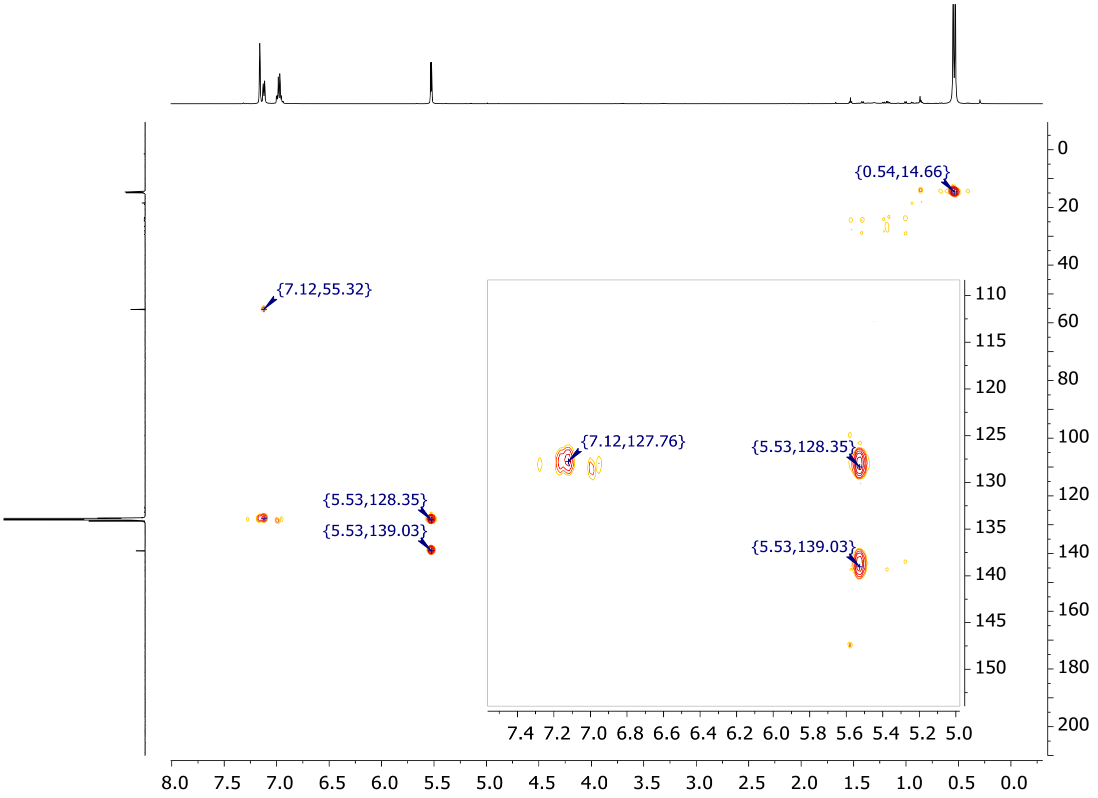

Figure S73. ^{1}H–$^{13}C\{^{1}H\}$ HMBC NMR spectrum of **8** (500 & 126 MHz, C_6D_6, 25°C).

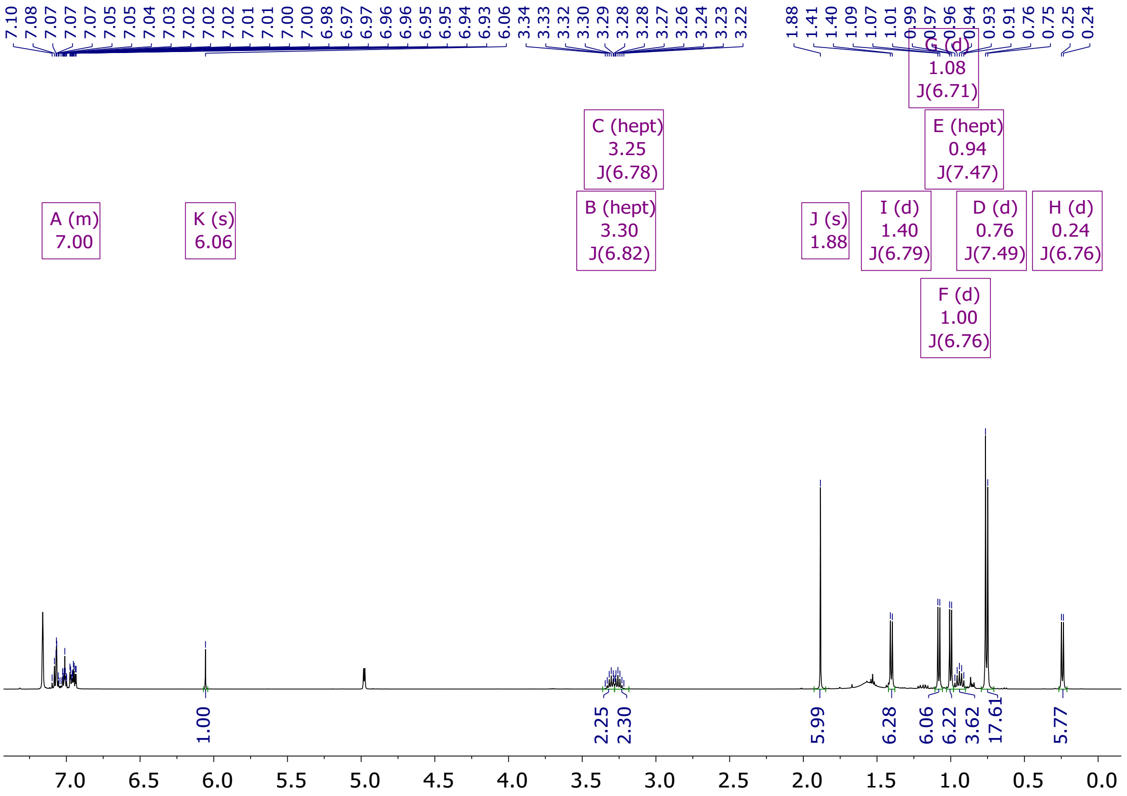

Figure S74. ^{1}H NMR spectrum of the crude reaction mixture of **5a** and AuCl(SMe_2) (500 MHz, C_6D_6, 25°C).

3. High-resolution mass spectra

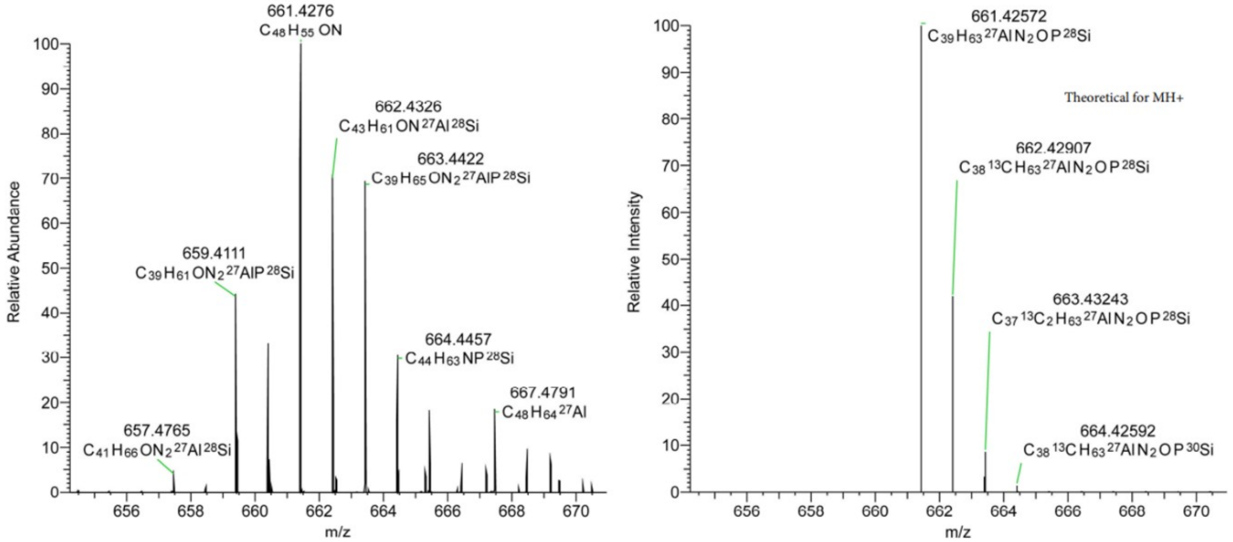

Figure S75. HRMS (APCI, positive mode) of **1** showing the $[M+H]^+$ peak at *m/z* 661.4276 (calc. 661.42572) (left) and simulation (right).

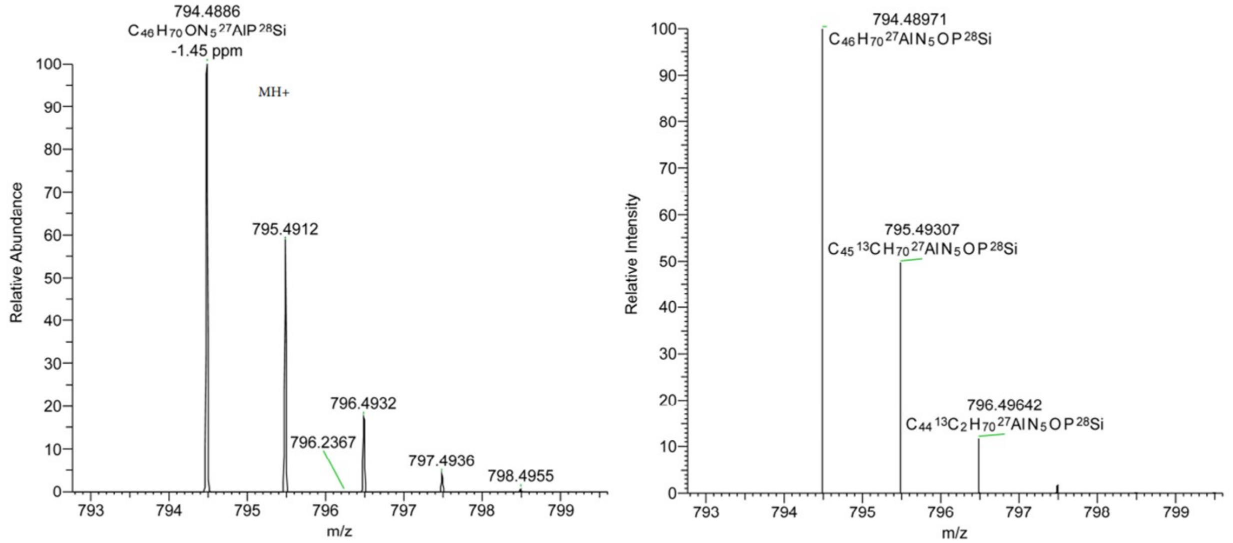

Figure S76. HRMS (APCI, positive mode) of **5a** showing the $[M+H]^+$ peak at *m/z* 794.4886 (calc. 794.48971) (left) and simulation (right).

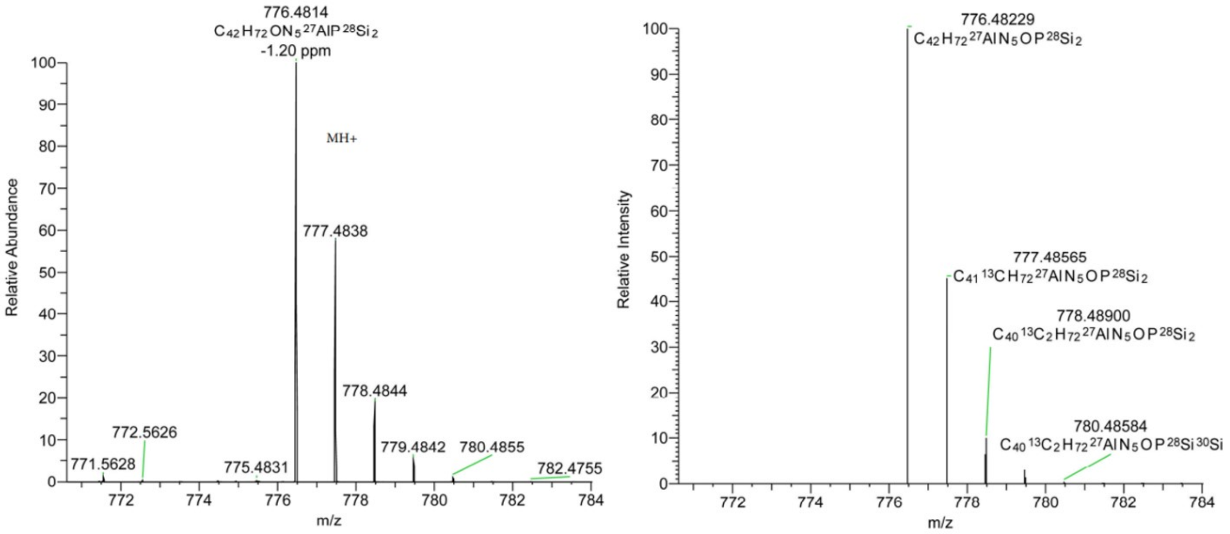

Figure S77. HRMS (APCI, positive mode) of **5b** showing the $[M+H]^+$ peak at *m/z* 776.4814 (calc. 776.48229) (left) and simulation (right).

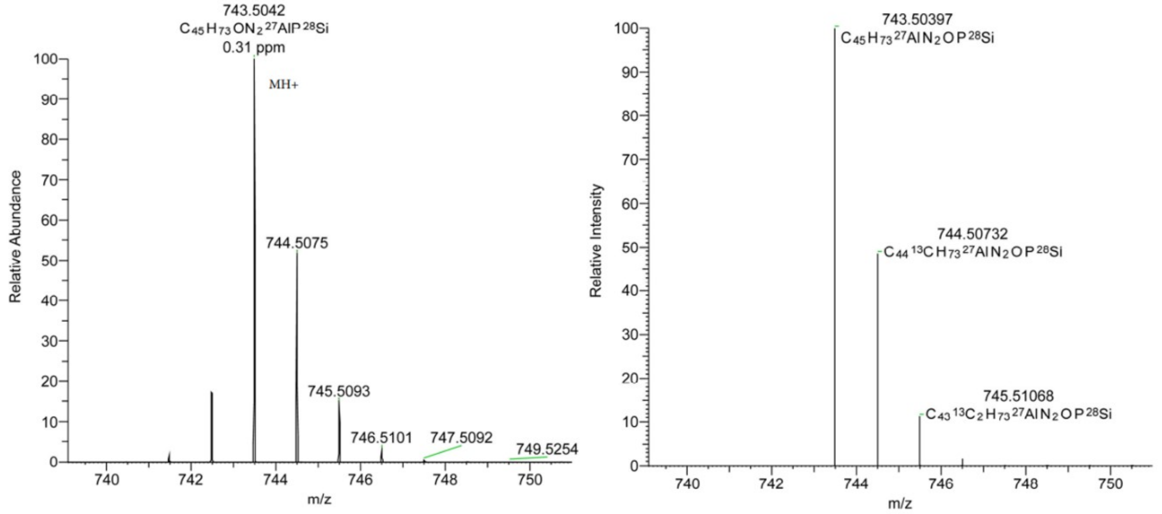

Figure S78. HRMS (APCI, positive mode) of **6** showing the $[M+H]^+$ peak at *m/z* 743.5042 (calc. 743.50397) (left) and simulation (right).

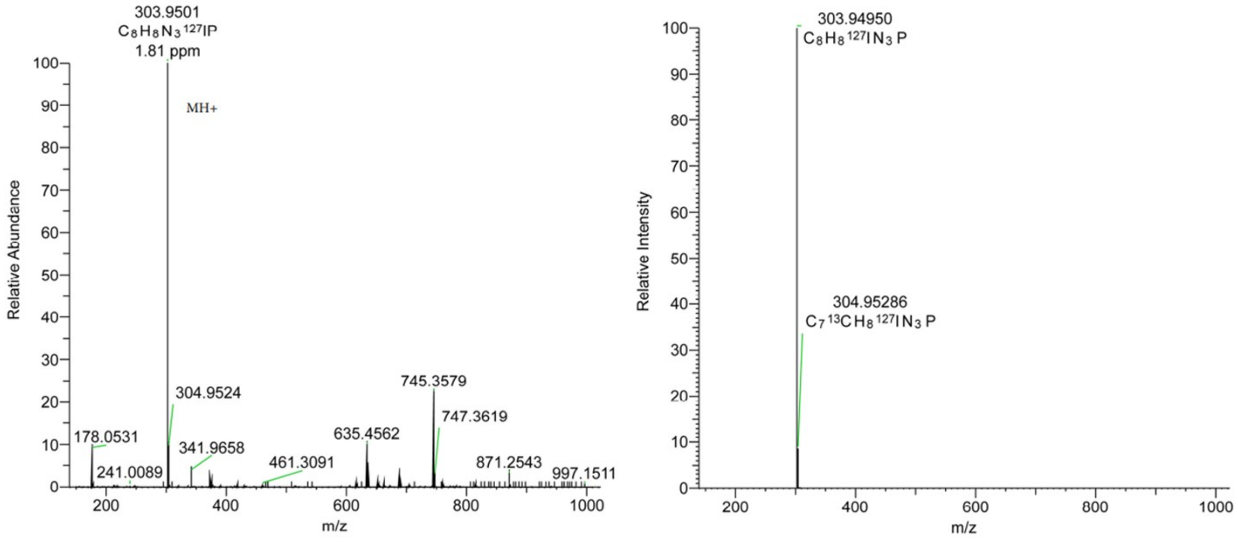

Figure S79. HRMS (APCI, positive mode) of **7** showing the $[M+H]^+$ peak at *m/z* 303.9501 (calc. 303.94950) (left) and simulation (right).

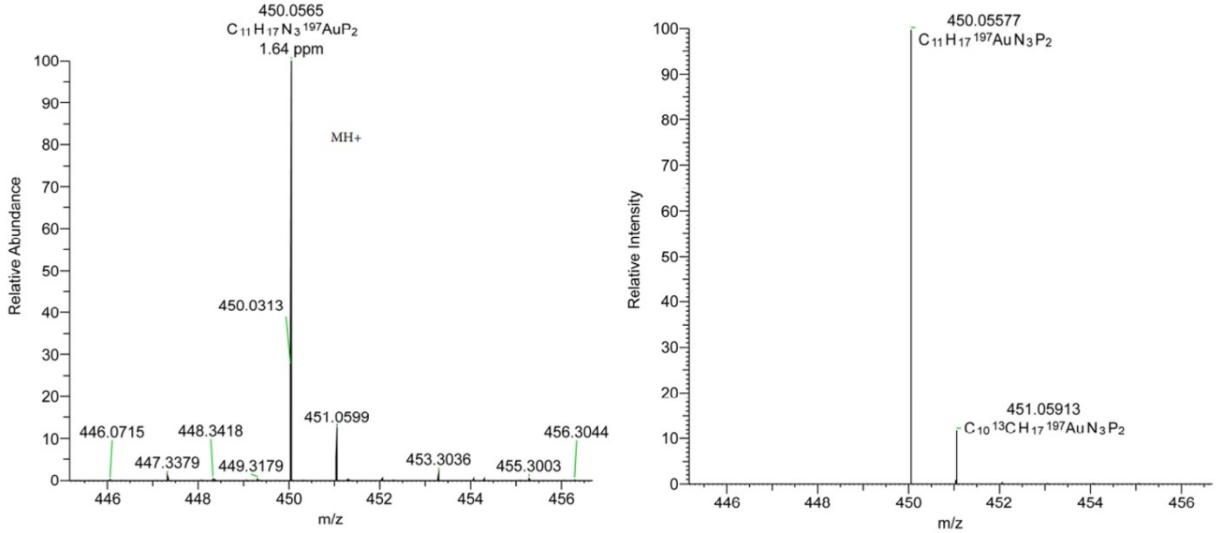

Figure S80. HRMS (APCI, positive mode) of **8** showing the $[M+H]^+$ peak at *m/z* 450.0565 (calc. 450.05577) (left) and simulation (right).

4. Single crystal X-ray data

Table S1. Crystallographic data and refinement details for **1** (CCDC 2531460).

Empirical formula	$C_{39}H_{62}AlN_2OPSi$
Formula weight	660.94
Crystal color, shape, size	red needle, 0.034 × 0.042 × 0.142 mm^3
Temperature	173(2) K
Wavelength	0.71073 Å (Mo K_α)
Crystal system, space group	Monoclinic, $P2_1/n$
Unit cell dimensions	a = 10.3918(7) Å α = 90°
	b = 19.2180(14) Å β = 94.649(2)°
	c = 20.1992(13) Å γ = 90°
Volume	4020.7(5) $Å^3$
Z	4
Density (calculated)	1.092 mg/m^3
Absorption coefficient	0.150 mm^{-1}
F(000)	1440
Data collection	
Diffractometer	Venture D8, Bruker
Source	Iμ3.0, Incoatec
Detector	Photon III
Theta range for data collection	2.023 to 28.322°
Index ranges	$-13 \leq h \leq 13$, $-25 \leq k \leq 25$, $-26 \leq l \leq 26$
Reflections collected	148222
Independent reflections	9994 [R_{int} = 0.0607; R_{sigma} = 0.0237]
Observed Reflections [I >2σ(I)]	7586
Completeness to theta = 25.242°	99.9%
Solution and Refinement	
Absorption correction	Multi-scan
Max. and min. transmission	0.7457 and 0.7128
Solution	Intrinsic methods
Refinement method	Full-matrix least-squares on F^2
Weighting scheme	$w = [\sigma^2F_o^2 + AP^2 + BP]^{-1}$, with
	$P = (F_o^2 + 2 F_c^2)/3$, A = 0.1107, B = 3.1576
Data / restraints / parameters	9994 / 0 / 416
Goodness-of-fit on F^2	1.036
Final R indices [I >2σ(I)]	R_1 = 0.0675, wR_2 = 0.1920
R indices (all data)	R_1 = 0.0872, wR_2 = 0.2151
Largest diff. peak and hole	1.223 and –0.824 e·$Å^{-3}$

Goodness-of-fit = $[\Sigma[w(F_o^2 - F_c^2)^2]/N_{observns} - N_{params})]^{1/2}$, all data. $R_1 = \Sigma(|F_o| - |F_c|) / \Sigma |F_o|$. $wR_2 = [\Sigma[w(F_o^2 - F_c^2)^2] / \Sigma [w(F_o2)^2]]^{1/2}$.

4.1. SXRD collection and refinement information for 1 (CCDC: 2531460)

A red, needle-shaped specimen of $C_{39}H_{62}AlN_2OPSi$, IUMSC 25042, approximate dimensions 0.085 × 0.132 × 0.218 mm^3, was placed on a Kapton mount with inert oil for crystal structure determination. The X-ray intensity data were measured on a Bruker D8 Venture KAPPA diffractometer equipped with a microfocus sealed tube (λ = 0.71073 Å) and a multilayer mirror monochromator.

Data collection. The data collection was performed using 1° ω and φ scans, frame times of 2 and 65 s, and a detector distance of 40 mm. Overall, 1395 frames were collected with a total exposure time of 17.05 hours. The frames were integrated with the SAINT V8.41 package using a narrow-frame algorithm.[6] The integration of the data using a monoclinic unit cell yielded 148222 reflections to a maximum θ angle of 28.30° (0.75 Å resolution), of which 9994 were independent (average redundancy 14.83, completeness = 99.9%, R_{int} = 6.07%, R_{sig} = 2.37%) and 7586 (75.9%) were greater than $2\sigma(F^2)$. The final cell constants of a = 10.3918(7) Å, b = 19.2180(14) Å, c = 20.1992(13) Å, α = 90°, β = 94.649(2)°, γ = 90°, volume = 4020.7(5) Å^3, are based upon the refinement of the XYZ-centroids of 9719 reflections above 20 $\sigma(I)$ with 2.28° < 2θ < 28.18°. Data were corrected for absorption effects using the Multi-Scan method in SADABS 2016/2. The calculated minimum and maximum transmission coefficients (based on crystal size) are 0.713 and 0.746.[7] Additional crystal and refinement information can be found in the tables.

Structure solution and refinement. The space group $P2_1/n$ (14) was determined based on intensity statistics and systematic absences. The structure was solved by SHELXS-97 and refined with full-matrix least squares / difference Fourier cycles using SHELXL-2019/2; Z = 4 for the formula unit $C_{39}H_{62}AlN_2OPSi$.[8,9] Non-hydrogen atoms were refined with anisotropic displacement parameters. The hydrogen atoms were placed in ideal positions and refined as riding atoms with relative isotropic displacement parameters. The final anisotropic full-matrix least-squares refinement on F^2 with 416 variables against 9994 data points and converged at R_1 = 6.75%, for the observed data and wR_2 = 21.51% for all data. The goodness-of-fit on F^2 was 1.04. The largest peak in the final difference electron density synthesis was 1.22 e$^-$/Å^3 and the deepest hole was −0.82 e$^-$/Å^3 with an RMS deviation of 0.063 e$^-$/Å^3. On the basis of the final model, the calculated density was 1.09 g/cm^3 and $F(000)$, 1440 e$^-$.

Table S2. Crystallographic data and refinement details for **2** (CCDC 2531461).

Empirical formula	$C_{39}H_{62}AlN_2OPSi$
Formula weight	660.94
Crystal color, shape, size	colorless block, 0.059 × 0.084 × 0.244 mm^3
Temperature	153(2) K
Wavelength	1.54184 (Cu K_α)
Crystal system, space group	Triclinic, *P*–1
Unit cell dimensions	a = 20.587(8) Å α = 62.123(17)°
	b = 21.285(9) Å β = 81.90(2)°
	c = 21.877(8) Å γ = 71.758(19)°
Volume	8048(6) $Å^3$
Z	8
Density (calculated)	1.091 mg/m^3
Absorption coefficient	1.319 mm^{-1}
F(000)	2880
Data collection	
Diffractometer	Venture D8, Bruker
Source	Iµ3.0, Incoatec
Detector	Photon III
Theta range for data collection	2.260 to 67.099°
Index ranges	$-24 \leq h \leq 24$, $-25 \leq k \leq 25$, $-26 \leq l \leq 26$
Reflections collected	110714
Independent reflections	28363 [R_{int} = 0.0576; R_{sigma} = 0.0508]
Observed Reflections [I >2σ(I)]	21821
Completeness to theta = 67.099°	98.96%
Solution and Refinement	
Absorption correction	Multi-scan
Max. and min. transmission	0.7528 and 0.6102
Solution	Intrinsic methods
Refinement method	Full-matrix least-squares on F^2
Weighting scheme	$w = [\sigma^2F_o^2 + AP^2 + BP]^{-1}$, with
	$P = (F_o^2 + 2 F_c^2)/3$, A = 0.1202, B = 7.5912
Data / restraints / parameters	28363 / 51 / 1716
Goodness-of-fit on F^2	1.022
Final R indices [I >2σ(I)]	R_1 = 0.0738, wR_2 = 0.2075
R indices (all data)	R_1 = 0.0914, wR_2 = 0.2216
Largest diff. peak and hole	1.129 and –0.516 e·$Å^{-3}$

Goodness-of-fit = $[\Sigma[w(F_o^2 - F_c^2)^2]/N_{observns} - N_{params})]^{1/2}$, all data. $R_1 = \Sigma(|F_o| - |F_c|) / \Sigma |F_o|$. $wR_2 = [\Sigma[w(F_o^2 - F_c^2)^2] / \Sigma [w(F_o2)^2]]^{1/2}$.

4.2. SXRD collection and refinement information for 2 (CCDC: 2531461)

A colourless, block shaped specimen of $C_{39}H_{62}AlN_2OPSi$, IUMSC 26011, approximate dimensions 0.059 × 0.084 × 0.244 mm^3, was used for the X-ray crystallographic analysis. The X-ray intensity data of 26011 were measured on a Bruker D8 KAPPA diffractometer system equipped with a microfocus sealed tube (λ = 1.54184 Å) and a multilayer mirror monochromator. The specimen was held at 152 K during the measurement with a Cryostream low temperature device.

Data collection. The data collection was performed using 1° ω and φ scans, frame times of 1 and 25 s, and a detector distance of 40 mm. A total of 3018 frames were collected. The total exposure time was 18.56 hours. The frames were integrated with the SAINT V8.41 package using a narrow-frame algorithm.[6] The integration of the data using a triclinic unit cell yielded a total of 110714 reflections to a maximum θ angle of 66.60° (0.84 Å resolution), of which 28363 were independent (average redundancy 3.90, completeness = 98.6%, R_{int} = 5.76%, R_{sig} = 5.08%) and 21821 (76.9%) were greater than $2\sigma(F^2)$. The final cell constants of a = 20.587(8) Å, b = 21.285(9) Å, c = 21.877(8) Å, volume = 8048(6) $Å^3$, are based upon the refinement of the XYZ-centroids of 1552 reflections above 20 $\sigma(I)$ with 27.66° < 2θ < 75.50°. Data were corrected for absorption effects using the Multi-Scan method in SADABS 2016/2. The calculated minimum and maximum transmission coefficients (based on crystal size) are 0.610 and 0.753.[7]

Structure solution and refinement. The structure was solved by XT, VERSION 2018/2 and refined using the SHELXL-2019/2 Software, in the space group *P*1 (2), with Z = 8 for the formula unit $C_{39}H_{62}AlN_2OPSi$.[8,9] The final anisotropic full-matrix least-squares refinement on F^2 with 1716 variables against 28363 data points and 51 restraints converged at R_1 = 7.38%, for the observed data and wR_2 = 22.16% for all data. The goodness-of-fit on F^2 was 1.02. The largest peak in the final difference electron density synthesis was 1.13 $e^-/Å^3$ and the deepest hole was −0.52 $e^-/Å^3$ with an RMS deviation of 0.066 $e^-/Å^3$. On the basis of the final model, the calculated density was 1.09 g/cm^3 and $F(000)$, 2880 e^-.

Table S3. Crystallographic data and refinement details for **3** (CCDC 2531462).

Empirical formula	$C_{39}H_{62}AlN_2OPSi$
Formula weight	660.94

Crystal color, shape, size	red block, 0.042 × 0.060 × 0.106 mm^3
Temperature	173(2) K
Wavelength	0.71073 (Mo $K_α$)
Crystal system, space group	Triclinic, *P*–1
Unit cell dimensions	a = 8.6485(7) Å α = 89.688(2)°
	b = 11.5609(8) Å β = 88.070(2)°
	c = 19.9875(14) Å γ = 87.097(2)°
Volume	1994.7(3) $Å^3$
Z	2
Density (calculated)	1.100 mg/m^3
Absorption coefficient	0.151 $mm^{–1}$
F(000)	720
Data collection	
Diffractometer	Venture D8, Bruker
Source	Iμ3.0, Incoatec
Detector	Photon III
Theta range for data collection	2.034 to 25.099°
Index ranges	–10 ≤ *h* ≤ 10, –13 ≤ *k* ≤ 13, –0 ≤ *l* ≤ 23
Reflections collected	7365
Independent reflections	7365 [R_{int} = 0.0894; R_{sigma} = 0.0576]
Observed Reflections [I >2σ(I)]	6271
Completeness to theta = 25.099°	99.4%
Solution and Refinement	
Absorption correction	Multi-scan
Max. and min. transmission	0.994 and 0.984
Solution	Intrinsic methods
Refinement method	Full-matrix least-squares on F^2
Weighting scheme	w = $[σ^2F_o^2 + AP^2 + BP]^{–1}$, with
	P = $(F_o^2 + 2 F_c^2)/3$, A = 0.0557, B = 0.8183
Data / restraints / parameters	7365 / 0 / 407
Goodness-of-fit on F^2	1.051
Final R indices [I >2σ(I)]	R_1 = 0.0509, wR_2 = 0.1133
R indices (all data)	R_1 = 0.0670, wR_2 = 0.1210
Largest diff. peak and hole	0.299 and –0.281 e·$Å^{–3}$

Goodness-of-fit = $[Σ[w(F_o^2 − F_c^2)^2]/N_{observns} − N_{params})]^{1/2}$, all data. $R_1 = Σ(|F_o| − |F_c|) / Σ |F_o|$. $wR_2 = [Σ[w(F_o^2 − F_c^2)^2] / Σ [w(F_o2)^2]]^{1/2}$.

4.3. SXRD collection and refinement information for 3 (CCDC: 2531462)

A red, block-shaped specimen of $C_{39}H_{62}AlN_2OPSi$, IUMSC 25447, approximate dimensions 0.042 × 0.060 × 0.106 mm^3, was placed on a Kapton mount with inert oil for crystal structure determination. The X-ray intensity data were measured on a Bruker D8 Venture KAPPA

diffractometer equipped with a microfocus sealed tube (λ = 0.71073 Å) and a multilayer mirror monochromator.

Data collection. The data collection was performed using 0.5° ω and φ scans, frame times of 1 and 10 s, and a detector distance of 40 mm. Overall, 3385 frames were collected with a total exposure time of 6.38 hours. The frames were integrated with the SAINT V8.41 package using a narrow-frame algorithm.[6] The integration of the data using a triclinic unit cell yielded 65432 reflections to a maximum θ angle of 25.10° (0.84 Å resolution), of which 7365 were independent (average redundancy 1.00, completeness = 99.4%, R_{int} = 5.67%, R_{sig} = 5.76%) and 6271 (85.1%) were greater than $2\sigma(F^2)$. The final cell constants of a = 8.6485(7) Å, b = 11.5609(8) Å, c = 19.9875(14) Å, α = 89.688(2)°, β = 88.070(2)°, γ = 87.097(2)°, volume = 1994.7(3) Å^3, are based upon the refinement of the XYZ-centroids of 6983 reflections above 20 σ(I) with 2.54° < 2θ < 24.85°. Data were corrected for absorption effects using the Multi-Scan method in TWINABS Bruker. The calculated minimum and maximum transmission coefficients (based on crystal size) are 0.675 and 0.745.[7] Additional crystal and refinement information can be found in the tables.

Structure solution and refinement. The space group $P1$ (2) was determined based on intensity statistics and systematic absences. The structure was solved by XT, VERSION 2018/2 and refined with full-matrix least squares / difference Fourier cycles using SHELXL-2019/2; Z = 2 for the formula unit $C_{39}H_{62}AlN_2OPSi$.[8,9] Non-hydrogen atoms were refined with anisotropic displacement parameters. The hydrogen atoms were placed in ideal positions and refined as riding atoms with relative isotropic displacement parameters. The final anisotropic full-matrix least-squares refinement on F^2 with 407 variables against 7365 data points and converged at R_1 = 5.09%, for the observed data and wR_2 = 12.10% for all data. The goodness-of-fit on F^2 was 1.05. The largest peak in the final difference electron density synthesis was 0.30 e$^-$/Å^3 and the deepest hole was −0.28 e$^-$/Å^3 with an RMS deviation of 0.052 e$^-$/Å^3. On the basis of the final model, the calculated density was 1.10 g/cm^3 and F(000), 720 e$^-$.

Table S4. Crystallographic data and refinement details for **4** (CCDC 2531463).

Empirical formula	$C_{47}H_{74}AlN_2NiOPSi$
Formula weight	827.83
Crystal color, shape, size	yellow needle, 0.086 × 0.152 × 0.479 mm^3
Temperature	153(2) K
Wavelength	0.71073 (Mo K_α)
Crystal system, space group	Monoclinic, $P2_1/n$
Unit cell dimensions	a = 18.5159(11) Å α = 90°
	b = 12.9787(8) Å β = 96.210(2)°
	c = 39.504(2) Å γ = 90°
Volume	1994.7(3) Å^3
Z	8
Density (calculated)	1.165 mg/m^3
Absorption coefficient	0.524 mm^{-1}
F(000)	3584
Data collection	
Diffractometer	Venture D8, Bruker
Source	Iμ3.0, Incoatec
Detector	Photon III
Theta range for data collection	1.881 to 27.516°
Index ranges	$-24 \leq h \leq 24$, $-16 \leq k \leq 16$, $-51 \leq l \leq 46$
Reflections collected	297938
Independent reflections	21649 [R_{int} = 0.0616; R_{sigma} = 0.0267]
Observed Reflections [I >2σ(I)]	17282
Completeness to theta = 25.242°	99.9%
Solution and Refinement	
Absorption correction	Multi-scan
Max. and min. transmission	0.956 and 0.788
Solution	Intrinsic methods
Refinement method	Full-matrix least-squares on F^2
Weighting scheme	$w = [\sigma^2 F_o^2 + AP^2 + BP]^{-1}$, with
	$P = (F_o^2 + 2 F_c^2)/3$, A = 0.0667, B = 5.3430
Data / restraints / parameters	21649 / 0 / 1064
Goodness-of-fit on F^2	1.035
Final R indices [I >2σ(I)]	R_1 = 0.0439, wR_2 = 0.1155
R indices (all data)	R_1 = 0.0605, wR_2 = 0.1285
Largest diff. peak and hole	0.661 and –0.544 e·Å^{-3}

Goodness-of-fit = $[\Sigma[w(F_o^2 - F_c^2)^2]/N_{observns} - N_{params})]^{1/2}$, all data. $R_1 = \Sigma(|F_o| - |F_c|) / \Sigma |F_o|$. $wR_2 = [\Sigma[w(F_o^2 - F_c^2)^2] / \Sigma [w(F_o2)^2]]^{1/2}$.

4.4. SXRD collection and refinement information for 4 (CCDC: 2531463)

A yellow, needle shaped specimen of $C_{47}H_{74}AlN_2NiOPSi$, IUMSC 25212, approximate dimensions 0.086 × 0.152 × 0.479 mm^3, was used for the X-ray crystallographic analysis. The X-ray intensity data of 25212 were measured on a Bruker D8 KAPPA diffractometer system equipped with a microfocus sealed tube (λ = 0.71073 Å) and a multilayer mirror monochromator. The specimen was held at 152 K during the measurement with a Cryostream low temperature device.

Data collection. The data collection was performed using 0.5° ω and φ scans, frame times of 1 and 35 s, and a detector distance of 40 mm. A total of 3702 frames were collected. The total exposure time was 24.34 hours. The frames were integrated with the SAINT V8.41 package using a narrow-frame algorithm.[6] The integration of the data using a monoclinic unit cell yielded a total of 297938 reflections to a maximum θ angle of 27.58° (0.77 Å resolution), of which 21649 were independent (average redundancy 13.76, completeness = 99.9%, R_{int} = 6.16%, R_{sig} = 2.67%) and 17282 (79.8%) were greater than $2\sigma(F^2)$. The final cell constants of a = 18.5159(11) Å, b = 12.9787(8) Å, c = 39.504(2) Å, volume = 9437.6(10) $Å^3$, are based upon the refinement of the XYZ-centroids of 9949 reflections above 20 $\sigma(I)$ with 2.24° < 2θ < 25.81°. Data were corrected for absorption effects using the Multi-Scan method in SADABS 2016/2. The calculated minimum and maximum transmission coefficients (based on crystal size) are 0.692 and 0.746.[7]

Structure solution and refinement. The structure was solved by XT, VERSION 2018/2 and refined using the SHELXL-2019/2 Software, in the space group $P2_1/n$ (14), with Z = 4 for the formula unit $C_{47}H_{74}AlN_2NiOPSi$.[8,9] The final anisotropic full-matrix least-squares refinement on F^2 with 1064 variables against 21649 data points converged at R_1 = 4.39%, for the observed data and wR_2 = 12.85% for all data. The goodness-of-fit on F^2 was 1.03. The largest peak in the final difference electron density synthesis was 0.66 $e^-/Å^3$ and the deepest hole was −0.54 $e^-/Å^3$ with an RMS deviation of 0.159 $e^-/Å^3$. On the basis of the final model, the calculated density was 1.17 g/cm^3 and F(000), 3584 e^-.

Table S5. Crystallographic data and refinement details for **5a** (CCDC 2531464).

Empirical formula	$C_{46}H_{69}AlN_5OPSi$
Formula weight	794.10

Crystal color, shape, size	colorless block, 0.085 × 0.121 × 0.224 mm^3
Temperature	153(2) K
Wavelength	0.71073 (Mo K_α)
Crystal system, space group	Monoclinic, $P2_1/c$
Unit cell dimensions	a = 11.7670(4) Å α = 90°
	b = 17.4531(5) Å β = 100.2680(10)°
	c = 22.6120(8) Å γ = 90°
Volume	4569.5(3) $Å^3$
Z	4
Density (calculated)	1.154 mg/m^3
Absorption coefficient	0.145 mm^{-1}
F(000)	1720
Data collection	
Diffractometer	Venture D8, Bruker
Source	Iμ3.0, Incoatec
Detector	Photon III
Theta range for data collection	1.831 to 28.296°
Index ranges	$-15 \le h \le 15$, $-23 \le k \le 23$, $-30 \le l \le 30$
Reflections collected	187989
Independent reflections	11336 [R_{int} = 0.0598; R_{sigma} = 0.0209]
Observed Reflections [I >2σ(I)]	9486
Completeness to theta = 25.242°	100%
Solution and Refinement	
Absorption correction	Multi-scan
Max. and min. transmission	0.7457 and 0.7217
Solution	Intrinsic methods
Refinement method	Full-matrix least-squares on F^2
Weighting scheme	$w = [\sigma^2F_o^2 + AP^2 + BP]^{-1}$, with
	$P = (F_o^2 + 2 F_c^2)/3$, A = 0.0721, B = 1.7472
Data / restraints / parameters	11336 / 0 / 515
Goodness-of-fit on F^2	1.114
Final R indices [I >2σ(I)]	R_1 = 0.0420, wR_2 = 0.1202
R indices (all data)	R_1 = 0.0537, wR_2 = 0.1346
Largest diff. peak and hole	0.525 and –0.475 $e \cdot Å^{-3}$

Goodness-of-fit = $[\Sigma[w(F_o^2 - F_c^2)^2]/N_{observns} - N_{params})]^{1/2}$, all data. $R_1 = \Sigma(|F_o| - |F_c|) / \Sigma |F_o|$. $wR_2 = [\Sigma[w(F_o^2 - F_c^2)^2] / \Sigma [w(F_o2)^2]]^{1/2}$.

4.5. SXRD collection and refinement information for 5a (CCDC: 2531464)

A colourless, block shaped specimen of $C_{46}H_{69}MgN_5OPSi$, IUMSC 25132, approximate dimensions 0.085 × 0.121 × 0.224 mm^3, was used for the X-ray crystallographic analysis. The X-ray intensity data of 25132 were measured on a Bruker D8 KAPPA diffractometer system equipped with a microfocus sealed tube (λ = 0.71073 Å) and a multilayer mirror monochromator. The specimen was held at 152 K during the measurement with a Cryostream low temperature device..

Data collection. The data collection was performed using 0.5° ω and φ scans, frame times of 1 and 20 s, and a detector distance of 40 mm. A total of 3215 frames were collected. The total exposure time was 11.83 hours. The frames were integrated with the SAINT V8.41 package using a narrow-frame algorithm.[6] The integration of the data using a monoclinic unit cell yielded a total of 187989 reflections to a maximum θ angle of 28.46° (0.75 Å resolution), of which 11336 were independent (average redundancy 16.58, completeness = 100.0%, R_{int} = 5.98%, R_{sig} = 2.09%) and 9486 (83.7%) were greater than 2σ(F^2). The final cell constants of a = 11.7670(4) Å, b = 17.4531(5) Å, c = 22.6120(8) Å, volume = 4569.5(3) Å^3, are based upon the refinement of the XYZ-centroids of 9438 reflections above 20 σ(*I*) with 2.42° < 2θ < 28.23°. Data were corrected for absorption effects using the Multi-Scan method in SADABS 2016/2. The calculated minimum and maximum transmission coefficients (based on crystal size) are 0.722 and 0.746.[7]

Structure solution and refinement. The structure was solved by XT, VERSION 2018/2 and refined using the SHELXL-2019/2 Software, in the space group $P2_1/c$ (14), with Z = 4 for the formula unit $C_{46}H_{69}MgN_5OPSi$.[8,9] The final anisotropic full-matrix least-squares refinement on F^2 with 515 variables against 11336 data points converged at R_1 = 4.20%, for the observed data and wR_2 = 13.46% for all data. The goodness-of-fit on F^2 was 1.11. The largest peak in the final difference electron density synthesis was 0.53 e$^-$/Å^3 and the deepest hole was −0.47 e$^-$/Å^3 with an RMS deviation of 0.121 e$^-$/Å^3. On the basis of the final model, the calculated density was 1.15 g/cm^3 and *F*(000), 1720 e$^-$.

Table S6. Crystallographic data and refinement details for **5b**·hex (CCDC 2531465).

Empirical formula	$C_{48}H_{85}AlN_5OPSi_2$
Formula weight	862.33

Crystal color, shape, size	colorless plate, 0.202 × 0.263 × 0.564 mm^3
Temperature	153(2) K
Wavelength	0.71073 (Mo K_α)
Crystal system, space group	Monoclinic, $P2_1/c$
Unit cell dimensions	a = 14.9852(8) Å α = 90°
	b = 16.4874(8) Å β = 106.040(2)°
	c = 22.1867(9) Å γ = 90°
Volume	5268.2(4) $Å^3$
Z	4
Density (calculated)	1.087 mg/m^3
Absorption coefficient	0.151 mm^{-1}
F(000)	1888
Data collection	
Diffractometer	Venture D8, Bruker
Source	Iμ3.0, Incoatec
Detector	Photon III
Theta range for data collection	1.878 to 28.315°
Index ranges	$-19 \le h \le 19$, $-22 \le k \le 21$, $-28 \le l \le 29$
Reflections collected	193875
Independent reflections	13066 [R_{int} = 0.0955; R_{sigma} = 0.0422]
Observed Reflections [I >2σ(I)]	10677
Completeness to theta = 25.242°	99.9%
Solution and Refinement	
Absorption correction	Multi-scan
Max. and min. transmission	0.970 and 0.919
Solution	Intrinsic methods
Refinement method	Full-matrix least-squares on F^2
Weighting scheme	$w = [\sigma^2F_o^2 + AP^2 + BP]^{-1}$, with
	$P = (F_o^2 + 2 F_c^2)/3$, A = 0.0906, B = 1.6588
Data / restraints / parameters	13066 / 24 / 533
Goodness-of-fit on F^2	1.079
Final R indices [I >2σ(I)]	R_1 = 0.0498, wR_2 = 0.1445
R indices (all data)	R_1 = 0.0618, wR_2 = 0.1580
Largest diff. peak and hole	0.538 and –0.522 e·$Å^{-3}$

Goodness-of-fit = $[\Sigma[w(F_o^2 - F_c^2)^2]/N_{observns} - N_{params})]^{1/2}$, all data. $R_1 = \Sigma(|F_o| - |F_c|) / \Sigma |F_o|$. $wR_2 = [\Sigma[w(F_o^2 - F_c^2)^2] / \Sigma [w(F_o2)^2]]^{1/2}$.

4.6. SXRD collection and refinement information for 5b (CCDC: 2531465)

A colourless, plate shaped specimen of $C_{42}H_{71}AlN_4O_2PSi_2$, IUMSC 25222, approximate dimensions 0.202 × 0.263 × 0.564 mm^3, was used for the X-ray crystallographic analysis. The X-ray intensity data of 25222 were measured on a Bruker D8 KAPPA diffractometer system equipped with a microfocus sealed tube (λ = 0.71073 Å) and a multilayer mirror monochromator. The specimen was held at 152 K during the measurement with a Cryostream low temperature device.

Data collection. The data collection was performed using 0.5° ω and φ scans, frame times of 1 and 10 s, and a detector distance of 40 mm. A total of 3215 frames were collected. The total exposure time was 6.39 hours. The frames were integrated with the SAINT V8.41 package using a narrow-frame algorithm.[6] The integration of the data using a monoclinic unit cell yielded a total of 193875 reflections to a maximum θ angle of 28.40° (0.75 Å resolution), of which 13066 were independent (average redundancy 14.84, completeness = 99.9%, R_{int} = 9.55%, R_{sig} = 4.22%) and 10677 (81.7%) were greater than 2σ(F^2). The final cell constants of a = 14.9852(8) Å, b = 16.4874(8) Å, c = 22.1867(9) Å, volume = 5268.2(4) Å^3, are based upon the refinement of the XYZ-centroids of 9970 reflections above 20 σ(*I*) with 2.47° < 2θ < 28.26°. Data were corrected for absorption effects using the Multi-Scan method in SADABS 2016/2. The calculated minimum and maximum transmission coefficients (based on crystal size) are 0.639 and 0.746.[7]

Structure solution and refinement. The structure was solved by XT, VERSION 2018/2 and refined using the SHELXL-2019/2 Software, in the space group $P2_1/c$ (14), with Z = 4 for the formula unit $C_{42}H_{71}AlN_4O_2PSi_2$.[8,9] The final anisotropic full-matrix least-squares refinement on F^2 with 533 variables against 13066 data points 24 and 24 restraints converged at R_1 = 4.98%, for the observed data and wR_2 = 15.80% for all data. The goodness-of-fit on F^2 was 1.08. The largest peak in the final difference electron density synthesis was 0.54 e$^-$/Å^3 and the deepest hole was −0.52 e$^-$/Å^3 with an RMS deviation of 0.115 e$^-$/Å^3. On the basis of the final model, the calculated density was 1.09 g/cm^3 and *F*(000), 1888 e$^-$.

Table S7. Crystallographic data and structure refinement details for **6** (CCDC 2531466).

Empirical formula	$C_{45}H_{72}AlN_2OPSi$
Formula weight	743.08

Crystal color, shape, size	colorless block, 0.136 × 0.138 × 0.375 mm^3
Temperature	153(2) K
Wavelength	0.71073 (Mo K_{α})
Crystal system, space group	Monoclinic, $P2_1/n$
Unit cell dimensions	a = 12.3931(3) Å α = 90°
	b = 17.6838(5) Å β = 100.1620(10)°
	c = 20.4243(6) Å γ = 90°
Volume	4405.9(2) $Å^3$
Z	4
Density (calculated)	1.120 mg/m^3
Absorption coefficient	0.144 mm^{-1}
F(000)	1624
Data collection	
Diffractometer	Venture D8, Bruker
Source	Iμ3.0, Incoatec
Detector	Photon III
Theta range for data collection	1.793 to 30.530°
Index ranges	$-16 \leq h \leq 17, -25 \leq k \leq 25, -29 \leq l \leq 29$
Reflections collected	172648
Independent reflections	13482 [R_{int} = 0.0713; R_{sigma} = 0.0322]
Observed Reflections [I >2σ(I)]	10397
Completeness to theta = 25.242°	100%
Solution and Refinement	
Absorption correction	Multi-scan
Max. and min. transmission	0.981 and 0.948
Solution	Intrinsic methods
Refinement method	Full-matrix least-squares on F^2
Weighting scheme	$w = [\sigma^2F_o^2 + AP^2 + BP]^{-1}$, with
	$P = (F_o^2 + 2 F_c^2)/3$, A = 0.0909, B = 2.0497
Data / restraints / parameters	13482 / 0 / 460
Goodness-of-fit on F^2	1.121
Final R indices [I >2σ(I)]	R_1 = 0.0524, wR_2 = 0.1492
R indices (all data)	R_1 = 0.0759, wR_2 = 0.1747
Largest diff. peak and hole	0.902 and –1.022 e·$Å^{-3}$

Goodness-of-fit = $[\Sigma[w(F_o^2 - F_c^2)^2]/N_{observns} - N_{params})]^{1/2}$, all data. $R_1 = \Sigma(|F_o| - |F_c|) / \Sigma |F_o|$. $wR_2 = [\Sigma[w(F_o^2 - F_c^2)^2] / \Sigma [w(F_o2)^2]]^{1/2}$.

4.7. SXRD collection and refinement information for 6 (CCDC: 2531466)

A colourless, block shaped specimen of $C_{45}H_{72}AlN_2OPSi$, IUMSC 25156, approximate dimensions 0.136 × 0.138 × 0.375 mm^3, was used for the X-ray crystallographic analysis. The X-ray intensity data of 25156 were measured on a Bruker D8 KAPPA diffractometer system

equipped with a microfocus sealed tube (λ = 0.71073 Å) and a multilayer mirror monochromator. The specimen was held at 152 K during the measurement with a Cryostream low temperature device.

Data collection. The data collection was performed using 0.5° ω and φ scans, frame times of 1 and 10 s, and a detector distance of 40 mm. A total of 3004 frames were collected. The total exposure time was 5.49 hours. The frames were integrated with the SAINT V8.41 package using a narrow-frame algorithm.[6] The integration of the data using a monoclinic unit cell yielded a total of 172648 reflections to a maximum θ angle of 30.56° (0.70 Å resolution), of which 13482 were independent (average redundancy 12.81, completeness = 100.0%, R_{int} = 7.13%, R_{sig} = 3.22%) and 10397 (77.1%) were greater than 2σ(F^2). The final cell constants of a = 12.3931(3) Å, b = 17.6838(5) Å, c = 20.4243(6) Å, volume = 4405.9(2) Å^3, are based upon the refinement of the XYZ-centroids of 9250 reflections above 20 σ(*I*) with 2.40° < 2θ < 29.51°. Data were corrected for absorption effects using the Multi-Scan method in SADABS 2016/2. The calculated minimum and maximum transmission coefficients (based on crystal size) are 0.718 and 0.746.[7]

Structure solution and refinement. The structure was solved by XT, VERSION 2018/2 and refined using the SHELXL-2019/2 Software, in the space group $P2_1/n$ (14), with Z = 4 for the formula unit $C_{45}H_{72}AlN_2OPSi$.[8,9] The final anisotropic full-matrix least-squares refinement on F^2 with 460 variables against 13482 data points converged at R_1 = 5.24%, for the observed data and wR_2 = 17.47% for all data. The goodness-of-fit on F^2 was 1.12. The largest peak in the final difference electron density synthesis was 0.90 e$^-$/Å^3 and the deepest hole was −1.02 e$^-$/Å^3 with an RMS deviation of 0.261 e$^-$/Å^3. On the basis of the final model, the calculated density was 1.12 g/cm^3 and *F*(000), 1624 e$^-$.

Table S8. Crystallographic data and structure refinement details for **7** (CCDC 2531467).

Empirical formula	$C_8H_7IN_3P$	
Formula weight	303.04	
Crystal color, shape, size	colorless plate, 0.057 × 0.134 × 0.196 mm^3	
Temperature	173(2) K	
Wavelength	0.71073 (Mo $K_α$)	
Crystal system, space group	Triclinic, *P*–1	
Unit cell dimensions	a = 6.2162(5) Å	α = 88.024(3)°
	b = 8.1483(7) Å	β = 85.736(3)°

	c = 9.9156(9) Å	γ = 89.791(3)°
Volume	500.55(7) $Å^3$	
Z	2	
Density (calculated)	2.011 mg/m^3	
Absorption coefficient	3.314 mm^{-1}	
F(000)	288	
Data collection		
Diffractometer	Venture D8, Bruker	
Source	Iμ3.0, Incoatec	
Detector	Photon III	
Theta range for data collection	2.501 to 27.586°	
Index ranges	$-8 \le h \le 8, -10 \le k \le 10, -12 \le l \le 12$	
Reflections collected	18998	
Independent reflections	2304 [R_{int} = 0.0483; R_{sigma} = 0.0255]	
Observed Reflections [I >2σ(I)]	2135	
Completeness to theta = 25.242°	99.2%	
Solution and Refinement		
Absorption correction	Multi-scan	
Max. and min. transmission	0.834 and 0.563	
Solution	Intrinsic methods	
Refinement method	Full-matrix least-squares on F^2	
Weighting scheme	$w = [\sigma^2 F_o^2 + AP^2 + BP]^{-1}$, with<br>$P = (F_o^2 + 2 F_c^2)/3$, A = 0.0422, B = 8.3784	
Data / restraints / parameters	2304 / 0 / 118	
Goodness-of-fit on F^2	1.162	
Final R indices [I >2σ(I)]	R_1 = 0.0528, wR_2 = 0.1517	
R indices (all data)	R_1 = 0.0561, wR_2 = 0.1536	
Largest diff. peak and hole	2.825 and –1.593 e·$Å^{-3}$	

Goodness-of-fit = $[\Sigma[w(F_o^2 - F_c^2)^2]/N_{observns} - N_{params})]^{1/2}$, all data. $R_1 = \Sigma(|F_o| - |F_c|) / \Sigma |F_o|$. $wR_2 = [\Sigma[w(F_o^2 - F_c^2)^2] / \Sigma [w(F_o 2)^2]]^{1/2}$.

4.8. SXRD collection and refinement information for 7 (CCDC: 2531467)

A colourless, plate-shaped specimen of $C_8H_7IN_3P$, IUMSC 25302, approximate dimensions 0.057 × 0.134 × 0.196 mm^3, was placed on a Kapton mount with inert oil for crystal structure determination. The X-ray intensity data were measured on a Bruker D8 Venture KAPPA diffractometer equipped with a microfocus sealed tube (λ = 0.71073 Å) and a multilayer mirror monochromator.

Data collection. The data collection was performed using 0.5 ° ω and φ scans, frame times of 1, 20 s, and a detector distance of 40 mm. Overall, 3505 frames were collected with a total exposure

time of 13.45 hours. The frames were integrated with the SAINT V8.41 package using a narrow-frame algorithm.[6] The integration of the data using a triclinic unit cell yielded 37100 reflections to a maximum θ angle of 27.62° (0.77 Å resolution), of which 2315 were independent (average redundancy 8.21, completeness = 99.2%, R_{int} = 6.28%, R_{sig} = 2.55%) and 2135 (92.2%) were greater than $2\sigma(F^2)$. The final cell constants of a = 6.2162(5) Å, b = 8.1483(7) Å, c = 9.9156(9) Å, α = 88.024(3)°, β = 85.736(3)°, γ = 89.791(3)°, volume = 500.55(7) $Å^3$, are based upon the refinement of the XYZ-centroids of 6848 reflections above 20 $\sigma(I)$ with 2.50° < 2θ < 27.45°. Data were corrected for absorption effects using the Multi-Scan method in TWINABS Bruker. The calculated minimum and maximum transmission coefficients (based on crystal size) are 0.551 and 0.746.[7] Additional crystal and refinement information can be found in the tables.

Structure solution and refinement. The space group *P*1 (2) was determined based on intensity statistics and systematic absences. The structure was solved by SHELXT 2018/2 and refined with full-matrix least squares / difference Fourier cycles using SHELXL-2019/2; Z = 2 for the formula unit $C_8H_7IN_3P$.[8,9] Non-hydrogen atoms were refined with anisotropic displacement parameters. The hydrogen atoms were placed in ideal positions and refined as riding atoms with relative isotropic displacement parameters. The final anisotropic full-matrix least-squares refinement on F^2 with 118 variables against 2304 data points and converged at R_1 = 5.28%, for the observed data and wR_2 = 15.36% for all data. The goodness-of-fit on F^2 was 1.16. The largest peak in the final difference electron density synthesis was 2.83 $e^-/Å^3$ and the deepest hole was −1.59 $e^-/Å^3$ with an RMS deviation of 0.215 $e^-/Å^3$. On the basis of the final model, the calculated density was 2.01 g/cm^3 and *F*(000), 288 e^-.

Table S8. Crystallographic data and structure refinement details for **8** (CCDC 2531468).

Empirical formula	$C_{22}H_{32}Au_2N_6P_4$
Formula weight	898.35
Crystal color, shape, size	colorless plate, 0.092 × 0.206 × 0.316 mm^3
Temperature	153(2) K
Wavelength	0.71073 (Mo K_α)
Crystal system, space group	Monoclinic, $P2_1/n$
Unit cell dimensions	a = 17.7846(16) Å α = 90°
	b = 9.0274(8) Å β = 104.408(3)°
	c = 18.6839(16) Å γ = 90°
Volume	2905.3(4) Å^3
Z	4
Density (calculated)	2.054 mg/m^3
Absorption coefficient	10.330 mm^{-1}
F(000)	1696
Data collection	
Diffractometer	Venture D8, Bruker
Source	Iμ3.0, Incoatec
Detector	Photon III
Theta range for data collection	2.251 to 27.511°
Index ranges	$-23 \leq h \leq 23$, $-11 \leq k \leq 11$, $-24 \leq l \leq 24$
Reflections collected	61066
Independent reflections	6629 [R_{int} = 0.0610; R_{sigma} = 0.0321]
Observed Reflections [I >2σ(I)]	5847
Completeness to theta = 25.242°	99.4%
Solution and Refinement	
Absorption correction	Multi-scan
Max. and min. transmission	0.7456 and 0.4486
Solution	Intrinsic methods
Refinement method	Full-matrix least-squares on F^2
Weighting scheme	$w = [\sigma^2F_o^2 + AP^2 + BP]^{-1}$, with
	$P = (F_o^2 + 2 F_c^2)/3$, A = 0.0395, B = 18.3577
Data / restraints / parameters	6629 / 0 / 307
Goodness-of-fit on F^2	1.166
Final R indices [I >2σ(I)]	R_1 = 0.0358, wR_2 = 0.0911
R indices (all data)	R_1 = 0.0420, wR_2 = 0.0935
Largest diff. peak and hole	2.825 and –1.593 e·Å^{-3}

Goodness-of-fit = $[\Sigma[w(F_o^2 - F_c^2)^2]/N_{observns} - N_{params})]^{1/2}$, all data. $R_1 = \Sigma(|F_o| - |F_c|) / \Sigma |F_o|$. $wR_2 = [\Sigma[w(F_o^2 - F_c^2)^2] / \Sigma [w(F_o2)^2]]^{1/2}$.

4.9. SXRD collection and refinement information for 8 (CCDC: 2531468)

A colourless, plate shaped specimen of $C_{22}H_{32}Au_2N_6P_4$, IUMSC 25245, approximate dimensions 0.092 × 0.206 × 0.316 mm^3, was used for the X-ray crystallographic analysis. The X-ray intensity data of 25245 were measured on a Bruker D8 KAPPA diffractometer system equipped with a microfocus sealed tube (λ = 0.71073 Å) and a multilayer mirror monochromator. The specimen was held at 152 K during the measurement with a Cryostream low temperature device.

Data collection. The data collection was performed using 0.5 ° ω and φ scans, frame times of 1 and 5 s, and a detector distance of 40 mm. A total of 2495 frames were collected. The total exposure time was 2.20 hours. The frames were integrated with the SAINT V8.41 package using a narrow-frame algorithm.[6] The integration of the data using a monoclinic unit cell yielded a total of 61066 reflections to a maximum θ angle of 27.52° (0.77 Å resolution), of which 6629 were independent (average redundancy 9.21, completeness = 99.4%, Rint = 6.10%, Rsig = 3.21%) and 5847 (88.2%) were greater than $2\sigma(F^2)$. The final cell constants of a = 17.7846(16) Å, b = 9.0274(8) Å, c = 18.6839(16) Å, volume = 2905.3(4) Å^3, are based upon the refinement of the XYZ-centroids of 9643 reflections above 20 $\sigma(I)$ with 2.36° < 2θ < 27.47°. Data were corrected for absorption effects using the Multi-Scan method in SADABS 2016/2. The calculated minimum and maximum transmission coefficients (based on crystal size) are 0.449 and 0.746.[7]

Structure solution and refinement. The structure was solved by XT, VERSION 2018/2 and refined using the SHELXL-2019/2 Software, in the space group $P2_1/n$ (14), with Z = 4 for the formula unit $C_{22}H_{32}Au_2N_6P_4$.[8,9] The final anisotropic full-matrix least-squares refinement on F^2 with 307 variables against 6629 data points converged at R_1 = 3.58%, for the observed data and wR_2 = 9.35% for all data. The goodness-of-fit on F^2 was 1.17. The largest peak in the final difference electron density synthesis was 1.34 e$^-$/Å^3 and the deepest hole was −1.26 e$^-$/Å^3 with an RMS deviation of 0.216 e$^-$/Å^3. On the basis of the final model, the calculated density was 2.05 g/cm^3 and $F(000)$, 1696 e$^-$.

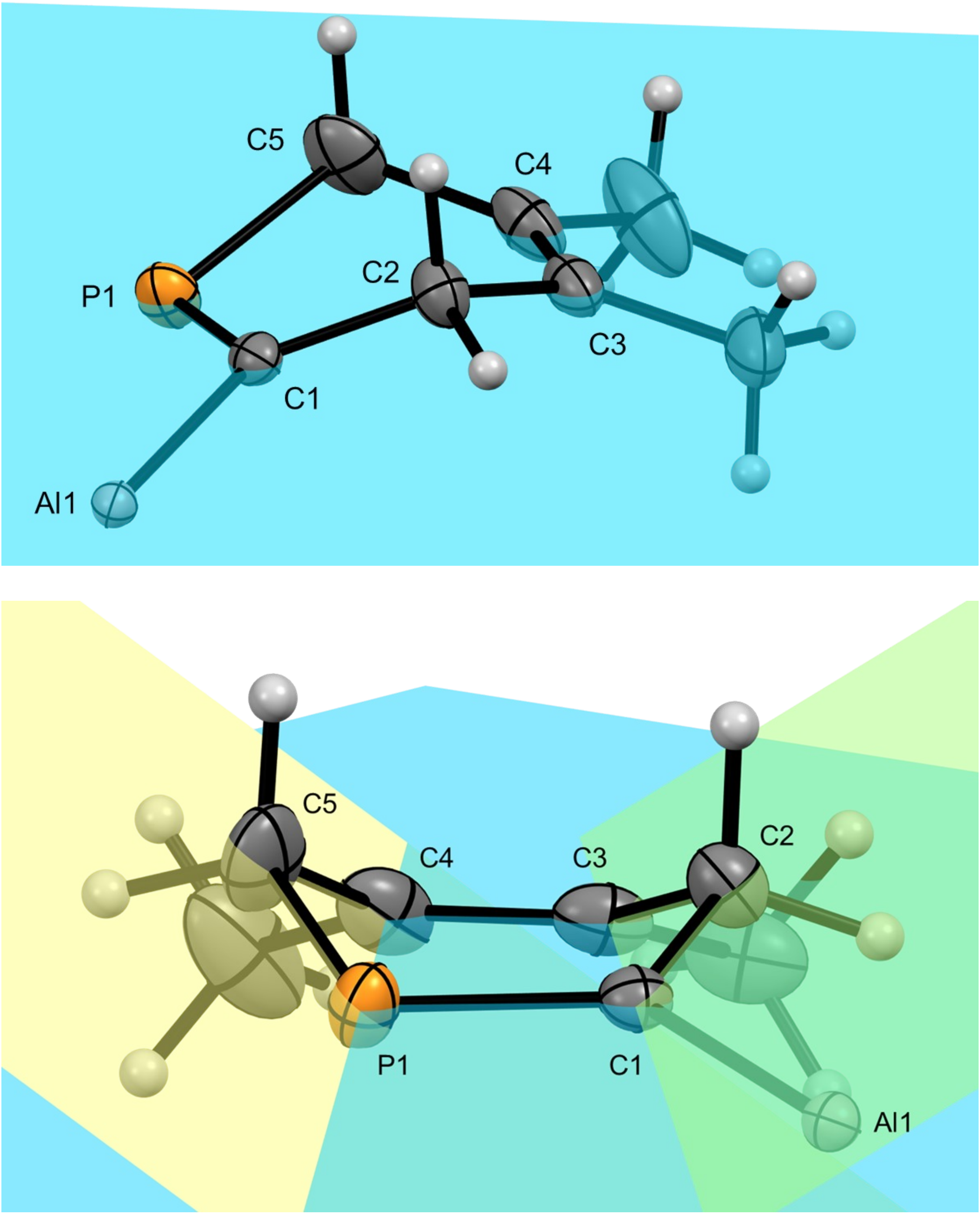

Figure S81. Molecular structure of the heterocyclic fragment in **6** (thermal ellipsoids are set at 50% probability; ligand environment on Al metallocentre is omitted for clarity).

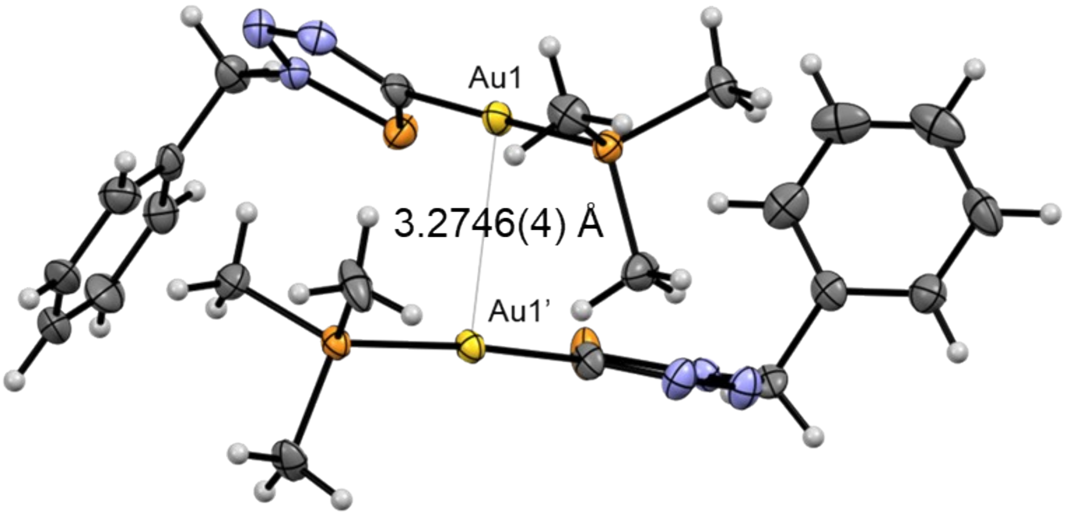

Figure S82. Aurophilic interaction in the molecular structure of **8** (thermal ellipsoids are set at 50% probability).

5. Computational Details

All the calculations reported in this paper were obtained with the Gaussian 16 suite of programs.[10] All species were optimized at the BP86[11,12]/def2-SVP[13] level of theory using the D3 dispersion correction suggested by Grimme et al.[14] and including solvent effects (solvent = n-hexane) with the polarizable continuum model (PCM).[15–17] All stationary points were characterized by frequency calculations.[18] Reactants and products have positive definite Hessian matrices, whereas transition structures show only one negative eigenvalue in their diagonalized force constant matrices, and their associated eigenvectors were confirmed to correspond to the motion along the reaction coordinate under consideration using the intrinsic reaction coordinate (IRC) method.[19] Single-point energy refinements were carried out at the same DFT level using the much larger def2-TZVPP basis-set This level is denoted PCM-BP86-D3/def2-TZVPP//BP86-D3/def2-SVP level. The computed thermochemistry data were corrected following Grimme's quasi-harmonic (QHA) model for entropy[20] with a frequency cutoff value of 100.0 cm^{-1} using the GoodVibes[21] program at the specified temperature and a standard concentration of 1 M.

The bonding situation in compounds **1** and **E** was analyzed with the Energy Decomposition Analysis (EDA) method.[22,23] Within this approach, the interaction between the selected fragments ($[M]^+$ and $[CP]^-$ in this case), ΔE_{int} can be decomposed into the following physically meaningful terms:

$$\Delta E_{int} = \Delta E_{elstat} + \Delta E_{Pauli} + \Delta E_{orb} + \Delta E_{disp}$$

The term ΔE_{elstat} corresponds to the classical electrostatic interaction between the unperturbed charge distributions of the deformed reactants and is usually attractive. The Pauli repulsion ΔE_{Pauli} comprises the destabilizing interactions between occupied orbitals and is responsible for any steric

repulsion. The orbital interaction ΔE_{orb} accounts for electron-pair bonding, charge transfer (interaction between occupied orbitals on one moiety with unoccupied orbitals on the other, including HOMO–LUMO interactions), and polarization (empty-occupied orbital mixing on one fragment due to the presence of another fragment). Finally, the ΔE_{disp} term takes into account the interactions which are due to dispersion forces.

The program package AMS 2023.101[24] was used for the EDA-NOCV calculations at the same BP86-D3 level, in conjunction with a triple-ζ-quality basis set using uncontracted Slater-type orbitals (STOs) augmented by two sets of polarization functions with a frozen-core approximation for the core electrons.[25] Auxiliary sets of s, p, d, f, and g STOs were used to fit the molecular densities and to represent the Coulomb and exchange potentials accurately in each SCF cycle.[26] Scalar relativistic effects were incorporated by applying the zeroth-order regular approximation (ZORA).[27–29] This level of theory is denoted ZORA-BP86-D3/TZ2P//PCM-BP86-D3/def2-SVP.

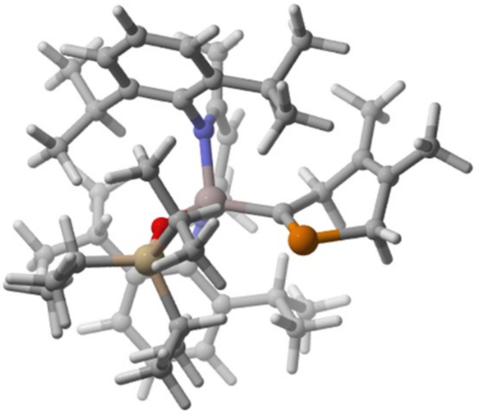

6–I (X-Ray)
ΔG_{rel} = 0.0 kcal/mol

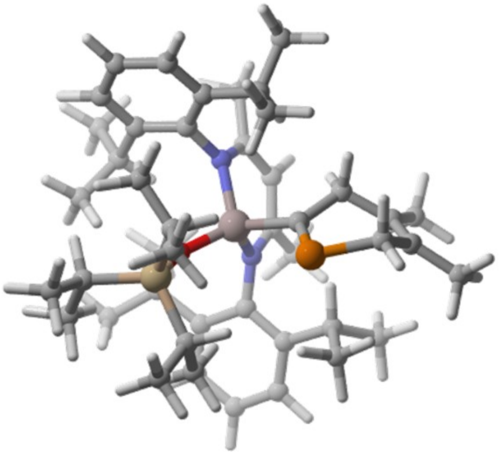

6–II
ΔG_{rel} = 0.5 kcal/mol

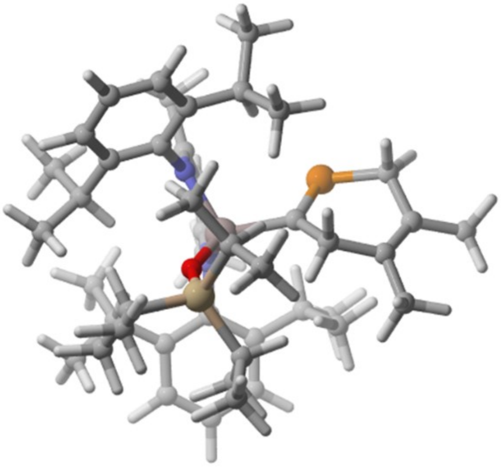

6–III
ΔG_{rel} = 1.6 kcal/mol

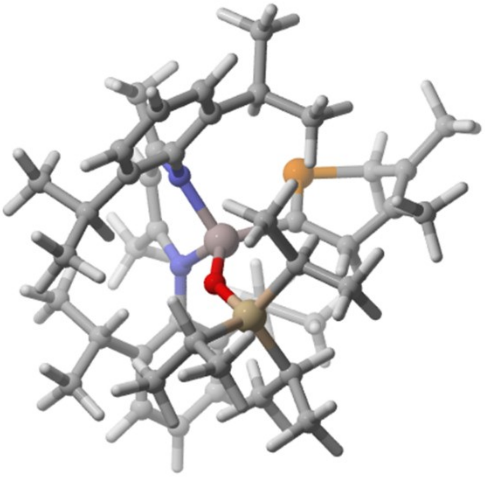

6–IV
ΔG_{rel} = 2.4 kcal/mol

Figure S83. Possible conformers of species **6**. All data were computed at the PCM-BP86-D3/def2-SVP level.

Cartesian coordinates (in Å) and free energies (in a.u.) of all the stationary points discussed in the text.

Al(^{DiPP}NacNac): G= -1481.767251

Al	-0.014642000	-0.433688000	-1.051584000
N	1.391586000	-0.368434000	0.405111000
N	-1.424083000	-0.269860000	0.396082000
C	1.236160000	-0.497009000	1.741091000
C	-0.031532000	-0.584170000	2.356326000
H	-0.039526000	-0.708924000	3.447418000
C	-1.287431000	-0.403377000	1.733470000
C	2.463488000	-0.512319000	2.627420000
H	2.199087000	-0.666067000	3.690212000
H	3.165664000	-1.310106000	2.309626000
H	3.022435000	0.441372000	2.531678000
C	-2.513402000	-0.338453000	2.622301000
H	-2.237130000	-0.335651000	3.693141000
H	-3.114018000	0.567185000	2.400634000
H	-3.181274000	-1.203685000	2.432925000
C	2.708867000	-0.070716000	-0.101201000
C	3.575439000	-1.122815000	-0.507863000
C	4.827395000	-0.781112000	-1.058285000
H	5.509404000	-1.582031000	-1.385840000
C	5.218818000	0.558225000	-1.199414000
H	6.201527000	0.803869000	-1.632403000
C	4.353430000	1.583687000	-0.791976000
H	4.663845000	2.634417000	-0.910864000
C	3.089391000	1.294670000	-0.239394000
C	3.162340000	-2.588736000	-0.388097000
H	2.240638000	-2.624200000	0.230237000
C	2.807376000	-3.165798000	-1.774859000
H	2.460497000	-4.217599000	-1.689560000
H	2.002495000	-2.572087000	-2.258210000
H	3.690917000	-3.149476000	-2.448445000
C	4.227712000	-3.447985000	0.320653000
H	3.850995000	-4.480139000	0.479671000
H	5.159244000	-3.527680000	-0.278836000
H	4.502344000	-3.026779000	1.310379000
C	2.158131000	2.435977000	0.172541000
H	1.253037000	1.986767000	0.631568000
C	1.692814000	3.237415000	-1.059972000
H	0.982547000	4.036950000	-0.761989000
H	2.549860000	3.720091000	-1.576022000
H	1.183802000	2.578321000	-1.794600000
C	2.796924000	3.355455000	1.232290000
H	3.122896000	2.782515000	2.125064000
H	3.685850000	3.887360000	0.831669000
H	2.070801000	4.125716000	1.567876000
C	-2.729096000	0.047166000	-0.126733000
C	-3.686706000	-0.983551000	-0.334099000
C	-4.954659000	-0.624686000	-0.834984000
H	-5.710869000	-1.407525000	-1.003219000
C	-5.264824000	0.709224000	-1.131489000
H	-6.263978000	0.972148000	-1.513788000
C	-4.292121000	1.707533000	-0.968141000

H	-4.539856000	2.744755000	-1.237708000
C	-3.005162000	1.401543000	-0.481588000
C	-3.341545000	-2.453322000	-0.095314000
H	-2.422267000	-2.488649000	0.525739000
C	-3.004630000	-3.137569000	-1.438311000
H	-2.704200000	-4.195276000	-1.280240000
H	-3.884179000	-3.126812000	-2.117034000
H	-2.173017000	-2.613414000	-1.955900000
C	-4.442954000	-3.220339000	0.660725000
H	-4.099283000	-4.245945000	0.909778000
H	-4.722749000	-2.711867000	1.607202000
H	-5.366368000	-3.324735000	0.052734000
C	-1.923580000	2.479854000	-0.362302000
H	-0.967070000	1.994806000	-0.676834000
C	-2.142561000	3.668893000	-1.310620000
H	-2.305152000	3.332887000	-2.355038000
H	-3.013216000	4.288603000	-1.006684000
H	-1.254058000	4.332562000	-1.300327000
C	-1.717860000	2.967423000	1.086804000
H	-0.935909000	3.755603000	1.121098000
H	-2.657019000	3.398029000	1.495115000
H	-1.390252000	2.150040000	1.758147000

PCOSi^{i}Pr$_3$: G= -1099.854156

P	3.836215000	0.020709000	-0.505350000
C	2.301152000	-0.008644000	-0.891640000
O	1.065997000	-0.047266000	-1.227899000
Si	-0.345908000	-0.008210000	-0.136327000
C	0.190410000	-1.038861000	1.361818000
H	1.171475000	-0.586324000	1.642422000
C	0.443169000	-2.509187000	0.971253000
H	1.169897000	-2.599472000	0.137105000
H	0.854856000	-3.079800000	1.832071000
H	-0.495791000	-3.016463000	0.660739000
C	-0.759177000	-0.911778000	2.571120000
H	-0.881665000	0.139529000	2.904077000
H	-1.771740000	-1.311393000	2.349667000
H	-0.365860000	-1.487558000	3.437379000
C	-0.595978000	1.841394000	0.216094000
H	-0.498927000	2.310366000	-0.791490000
C	0.528259000	2.399985000	1.114736000
H	1.539967000	2.175283000	0.716481000
H	0.442560000	3.504558000	1.210137000
H	0.475158000	1.979740000	2.142157000
C	-1.998370000	2.179880000	0.764589000
H	-2.807613000	1.884297000	0.065153000
H	-2.198706000	1.680415000	1.736540000
H	-2.095837000	3.274346000	0.936283000
C	-1.655172000	-0.803412000	-1.252001000
H	-1.153367000	-1.732779000	-1.611007000
C	-1.957487000	0.080179000	-2.479810000
H	-1.033180000	0.355401000	-3.029025000
H	-2.628057000	-0.447651000	-3.192562000
H	-2.469090000	1.022447000	-2.187292000
C	-2.936552000	-1.214112000	-0.497573000

H	-2.729938000	-1.930405000	0.324488000
H	-3.457870000	-0.339263000	-0.054562000
H	-3.656298000	-1.705500000	-1.188244000

TS1: G= -2581.615115 (i = -168 cm-1)

Al	-0.634370000	0.428109000	-0.688276000
P	-0.009798000	-2.194450000	-2.766142000
N	-2.448927000	0.802793000	-0.316301000
N	0.034304000	2.182626000	-0.604531000
C	0.099821000	-1.137710000	-1.485792000
C	-2.964984000	2.059801000	-0.317619000
C	-2.191132000	3.179463000	-0.708395000
H	-2.738462000	4.120845000	-0.853220000
C	-0.789102000	3.251214000	-0.845612000
C	-4.387083000	2.274859000	0.139888000
H	-4.768056000	3.259338000	-0.190549000
H	-5.062673000	1.475514000	-0.222853000
H	-4.435049000	2.246037000	1.250494000
C	-0.165712000	4.553016000	-1.292323000
H	0.259628000	4.418008000	-2.310225000
H	-0.910177000	5.369515000	-1.327138000
H	0.679729000	4.858385000	-0.644892000
C	-3.236548000	-0.311526000	0.163289000
C	-3.829213000	-1.199151000	-0.778351000
C	-4.566366000	-2.287881000	-0.268067000
H	-5.038873000	-2.993644000	-0.966337000
C	-4.699211000	-2.498882000	1.111888000
H	-5.278124000	-3.359697000	1.482233000
C	-4.080854000	-1.628363000	2.020306000
H	-4.169269000	-1.819395000	3.100534000
C	-3.335382000	-0.522946000	1.566418000
C	-3.677328000	-0.982553000	-2.284163000
H	-2.643193000	-0.608202000	-2.454862000
C	-3.792978000	-2.280344000	-3.098139000
H	-3.496503000	-2.089399000	-4.149448000
H	-3.113830000	-3.062039000	-2.702353000
H	-4.831630000	-2.676162000	-3.111005000
C	-4.660533000	0.084323000	-2.812297000
H	-4.553316000	0.196281000	-3.911633000
H	-5.711777000	-0.205850000	-2.599484000
H	-4.480531000	1.079384000	-2.360550000
C	-2.564120000	0.356082000	2.551427000
H	-2.405391000	1.350582000	2.083479000
C	-1.168765000	-0.254085000	2.791057000
H	-0.564832000	0.369155000	3.478292000
H	-1.250721000	-1.273280000	3.219652000
H	-0.594971000	-0.358413000	1.839849000
C	-3.299365000	0.597667000	3.880255000
H	-4.326776000	0.982275000	3.712329000
H	-3.378739000	-0.329062000	4.486342000
H	-2.747511000	1.339677000	4.493683000
C	1.428683000	2.438496000	-0.326044000
C	2.419996000	2.256401000	-1.326031000
C	3.752713000	2.588103000	-1.001032000
H	4.537711000	2.460086000	-1.763026000

C	4.093847000	3.084320000	0.263615000
H	5.138446000	3.350598000	0.489226000
C	3.107536000	3.209634000	1.253305000
H	3.391989000	3.557898000	2.257987000
C	1.767086000	2.866424000	0.992384000
C	2.096669000	1.723029000	-2.719877000
H	1.014583000	1.470460000	-2.745926000
C	2.868910000	0.422112000	-3.011599000
H	2.551719000	-0.006773000	-3.984001000
H	3.964511000	0.600058000	-3.057263000
H	2.663652000	-0.331559000	-2.228266000
C	2.352466000	2.784623000	-3.809575000
H	2.062522000	2.395957000	-4.808001000
H	1.777013000	3.715152000	-3.622959000
H	3.427077000	3.062185000	-3.855487000
C	0.720074000	2.939185000	2.108197000
H	-0.131358000	2.296590000	1.797059000
C	1.251456000	2.382498000	3.442782000
H	1.664407000	1.361834000	3.318777000
H	2.049391000	3.023624000	3.871978000
H	0.434636000	2.341661000	4.193391000
C	0.168850000	4.367633000	2.304480000
H	-0.559327000	4.392741000	3.142601000
H	0.990964000	5.075072000	2.544883000
H	-0.350760000	4.741132000	1.401224000
O	1.120212000	-1.009543000	-0.375631000
Si	2.062918000	-2.274489000	0.375194000
C	0.927136000	-3.799631000	0.538945000
H	0.687903000	-4.030639000	-0.525889000
C	-0.398175000	-3.460323000	1.244946000
H	-0.936354000	-2.631406000	0.736934000
H	-1.083557000	-4.337014000	1.251858000
H	-0.237663000	-3.164562000	2.304699000
C	1.614138000	-5.035746000	1.152050000
H	2.513334000	-5.342614000	0.578254000
H	1.934864000	-4.861859000	2.201259000
H	0.921452000	-5.907107000	1.164064000
C	3.660153000	-2.641939000	-0.620973000
H	3.995192000	-1.627307000	-0.942415000
C	3.410151000	-3.488515000	-1.882750000
H	2.563403000	-3.095139000	-2.487939000
H	4.320881000	-3.531151000	-2.521477000
H	3.150898000	-4.536343000	-1.614930000
C	4.789116000	-3.259477000	0.233641000
H	5.093759000	-2.613383000	1.082086000
H	4.494721000	-4.243255000	0.657089000
H	5.697138000	-3.435252000	-0.385811000
C	2.475873000	-1.388076000	2.023238000
H	1.502467000	-0.919820000	2.297276000
C	3.478195000	-0.245594000	1.762466000
H	3.152837000	0.404897000	0.925267000
H	3.600830000	0.405616000	2.656273000
H	4.487365000	-0.634406000	1.509940000
C	2.903227000	-2.288676000	3.196538000
H	2.107961000	-3.010873000	3.475001000
H	3.816193000	-2.876705000	2.965109000
H	3.128375000	-1.680432000	4.101846000

1: G= -2581.748411

Al	0.012508000	-0.474740000	0.824910000
P	0.157243000	-0.674535000	4.367418000
N	-1.459368000	-1.597947000	0.322048000
N	1.412726000	-1.616306000	0.179401000
C	0.104578000	-0.537459000	2.797539000
C	-1.295022000	-2.926093000	0.514338000
C	-0.018109000	-3.522390000	0.667722000
H	-0.013645000	-4.597618000	0.889863000
C	1.247212000	-2.942127000	0.390978000
C	-2.517923000	-3.812877000	0.544774000
H	-2.257127000	-4.862792000	0.771431000
H	-3.239682000	-3.441067000	1.301746000
H	-3.049961000	-3.778723000	-0.427482000
C	2.451306000	-3.852836000	0.313966000
H	2.168637000	-4.914499000	0.433828000
H	2.980583000	-3.725097000	-0.652167000
H	3.182384000	-3.581904000	1.103686000
C	-2.736510000	-1.092004000	-0.116204000
C	-3.656150000	-0.555283000	0.826980000
C	-4.882472000	-0.046212000	0.353350000
H	-5.597244000	0.384179000	1.072182000
C	-5.211383000	-0.085574000	-1.008090000
H	-6.178688000	0.309522000	-1.356491000
C	-4.297245000	-0.621134000	-1.924451000
H	-4.551342000	-0.638725000	-2.996345000
C	-3.045774000	-1.116433000	-1.505285000
C	-3.336281000	-0.494239000	2.316474000
H	-2.435301000	-1.115986000	2.494097000
C	-2.982042000	0.949801000	2.725990000
H	-2.686492000	0.998436000	3.794235000
H	-2.130138000	1.334629000	2.129914000
H	-3.844920000	1.631051000	2.564547000
C	-4.462911000	-1.069677000	3.195709000
H	-4.136829000	-1.112161000	4.255914000
H	-5.381428000	-0.446305000	3.158597000
H	-4.740720000	-2.097209000	2.880239000
C	-2.056172000	-1.626964000	-2.549184000
H	-1.127346000	-1.919178000	-2.019151000
C	-1.688395000	-0.509938000	-3.545430000
H	-0.983468000	-0.891071000	-4.311601000
H	-2.583131000	-0.127238000	-4.079285000
H	-1.211422000	0.348435000	-3.030898000
C	-2.582407000	-2.875828000	-3.284825000
H	-2.832953000	-3.692546000	-2.577223000
H	-3.497192000	-2.647291000	-3.871837000
H	-1.818909000	-3.264176000	-3.991446000
C	2.685128000	-1.129854000	-0.300087000
C	3.723134000	-0.817046000	0.620237000
C	4.966329000	-0.389960000	0.108204000
H	5.777928000	-0.138619000	0.808948000
C	5.184077000	-0.279963000	-1.269719000
H	6.165386000	0.042541000	-1.652298000
C	4.137423000	-0.552590000	-2.162076000

H	4.308509000	-0.422132000	-3.239861000
C	2.867759000	-0.956264000	-1.703409000
C	3.512450000	-0.864485000	2.130657000
H	2.563014000	-1.401145000	2.330772000
C	3.344109000	0.571051000	2.671917000
H	3.127942000	0.561264000	3.760258000
H	4.264032000	1.169649000	2.498943000
H	2.499915000	1.090491000	2.174608000
C	4.630507000	-1.614510000	2.879338000
H	4.369612000	-1.716712000	3.953276000
H	4.790290000	-2.632950000	2.467217000
H	5.601040000	-1.077028000	2.827306000
C	1.709491000	-1.153568000	-2.683116000
H	0.810354000	-0.736093000	-2.177961000
C	1.903057000	-0.378624000	-3.998662000
H	2.128990000	0.690892000	-3.817614000
H	2.723132000	-0.806630000	-4.613232000
H	0.982413000	-0.430387000	-4.611894000
C	1.429953000	-2.642038000	-2.986151000
H	0.634270000	-2.738043000	-3.754279000
H	2.341594000	-3.140235000	-3.379109000
H	1.087909000	-3.197548000	-2.091898000
O	-0.022099000	1.068603000	0.082860000
Si	0.035233000	2.710295000	-0.227977000
C	-1.783663000	3.301850000	-0.269842000
H	-2.171855000	2.947670000	0.714147000
C	-2.573377000	2.557249000	-1.362041000
H	-2.465490000	1.459058000	-1.262683000
H	-3.661470000	2.782940000	-1.306595000
H	-2.231857000	2.838352000	-2.382862000
C	-2.001607000	4.825668000	-0.330195000
H	-1.539493000	5.350129000	0.531771000
H	-1.579735000	5.275304000	-1.254053000
H	-3.087597000	5.072018000	-0.318524000
C	1.028997000	3.524558000	1.190242000
H	1.964774000	2.916722000	1.210852000
C	0.320305000	3.340724000	2.545862000
H	0.091806000	2.274952000	2.754654000
H	0.949234000	3.712851000	3.385962000
H	-0.638536000	3.903332000	2.582913000
C	1.434103000	4.991563000	0.950524000
H	2.007769000	5.122790000	0.009694000
H	0.550498000	5.661178000	0.890339000
H	2.071466000	5.371866000	1.780488000
C	0.952469000	2.863470000	-1.900045000
H	0.527056000	2.009901000	-2.481239000
C	2.458983000	2.593676000	-1.707623000
H	2.648397000	1.663065000	-1.134146000
H	2.985090000	2.487571000	-2.683017000
H	2.956199000	3.427938000	-1.167417000
C	0.700368000	4.154952000	-2.698568000
H	-0.373789000	4.292884000	-2.941326000
H	1.031763000	5.057810000	-2.141647000
H	1.258783000	4.146588000	-3.662070000

TS0: G= -2581.606275 (i = -22 cm-1)

Al	-1.583517000	0.651041000	-1.142966000
N	-2.935305000	-0.111249000	0.158944000
N	-1.269953000	2.161415000	0.163235000
P	-0.109168000	-1.844056000	-1.487944000
C	1.184195000	-1.755670000	-0.562395000
O	2.207497000	-1.508265000	0.188836000
Si	3.838602000	-1.446089000	-0.511124000
C	-3.758886000	0.673436000	0.881353000
C	-3.511974000	2.054859000	1.082738000
H	-4.292545000	2.621862000	1.607458000
C	-2.298999000	2.734112000	0.827561000
C	-5.009324000	0.077748000	1.493839000
H	-5.706600000	-0.246834000	0.693872000
H	-4.773012000	-0.832747000	2.080039000
H	-5.529333000	0.803357000	2.146306000
C	-2.174495000	4.173119000	1.286430000
H	-3.015358000	4.470026000	1.940298000
H	-1.220079000	4.361453000	1.814977000
H	-2.168553000	4.836454000	0.394664000
C	-3.188964000	-1.525872000	0.109752000
C	-4.085323000	-2.053707000	-0.861040000
C	-4.295306000	-3.448321000	-0.889627000
H	-4.982276000	-3.874319000	-1.638247000
C	-3.643885000	-4.298362000	0.011604000
H	-3.826399000	-5.384325000	-0.020681000
C	-2.738568000	-3.765890000	0.942286000
H	-2.211346000	-4.447779000	1.625172000
C	-2.476451000	-2.383197000	1.002217000
C	-4.780135000	-1.169597000	-1.896817000
H	-4.608157000	-0.109476000	-1.613244000
C	-4.144930000	-1.377386000	-3.288547000
H	-3.057058000	-1.156776000	-3.263001000
H	-4.275268000	-2.426062000	-3.631356000
H	-4.613616000	-0.707741000	-4.040632000
C	-6.304879000	-1.395880000	-1.945641000
H	-6.773017000	-1.279896000	-0.945609000
H	-6.783640000	-0.668726000	-2.634447000
H	-6.557038000	-2.412739000	-2.314250000
C	-1.435572000	-1.825762000	1.976653000
H	-0.914854000	-1.003924000	1.437911000
C	-0.362382000	-2.857504000	2.366193000
H	0.465493000	-2.358493000	2.908024000
H	-0.767945000	-3.644103000	3.038145000
H	0.068414000	-3.348296000	1.470284000
C	-2.075350000	-1.208825000	3.239099000
H	-1.288740000	-0.893464000	3.956461000
H	-2.679827000	-0.311168000	3.004785000
H	-2.727916000	-1.945832000	3.753675000
C	0.029058000	2.765818000	0.258956000
C	0.514265000	3.630043000	-0.762041000
C	1.799399000	4.190823000	-0.607134000
H	2.193474000	4.856088000	-1.392113000
C	2.577183000	3.929924000	0.528660000
H	3.571997000	4.390426000	0.636713000
C	2.087225000	3.071064000	1.523919000
H	2.699999000	2.872674000	2.417541000
C	0.825522000	2.456894000	1.402307000

C	-0.319295000	3.977283000	-1.993828000
H	-1.324417000	3.523489000	-1.852931000
C	0.284345000	3.355740000	-3.269342000
H	-0.337925000	3.598477000	-4.156641000
H	1.310812000	3.739006000	-3.455007000
H	0.335249000	2.250573000	-3.179852000
C	-0.512633000	5.499674000	-2.150065000
H	-0.921868000	5.957219000	-1.225325000
H	0.445515000	6.012594000	-2.379482000
H	-1.212737000	5.719650000	-2.983189000
C	0.318610000	1.518274000	2.498341000
H	-0.566543000	0.989513000	2.090539000
C	1.359256000	0.449478000	2.874822000
H	0.936078000	-0.253297000	3.621834000
H	1.669227000	-0.137832000	1.989085000
H	2.268244000	0.894424000	3.331793000
C	-0.147361000	2.293997000	3.748064000
H	-0.520461000	1.592547000	4.523897000
H	0.689268000	2.877622000	4.188997000
H	-0.967721000	3.000232000	3.513271000
C	4.915854000	-0.919182000	0.959739000
H	5.954411000	-1.163767000	0.633352000
C	4.595872000	-1.767322000	2.209968000
H	4.711735000	-2.855282000	2.023398000
H	5.272848000	-1.499667000	3.050700000
H	3.554019000	-1.598400000	2.552706000
C	4.852070000	0.591040000	1.260427000
H	5.239384000	1.201631000	0.419428000
H	3.814137000	0.925144000	1.456061000
H	5.458389000	0.839798000	2.159377000
C	4.219977000	-3.211928000	-1.133927000
H	3.903605000	-3.192803000	-2.202939000
C	3.398333000	-4.307256000	-0.422177000
H	2.306409000	-4.146825000	-0.537947000
H	3.638379000	-5.308120000	-0.843907000
H	3.618414000	-4.348153000	0.665736000
C	5.732916000	-3.518840000	-1.090899000
H	6.345358000	-2.748501000	-1.606740000
H	6.102784000	-3.591975000	-0.045376000
H	5.950892000	-4.493394000	-1.580199000
C	3.700673000	-0.226983000	-1.959764000
H	3.102418000	-0.803796000	-2.705009000
C	5.082897000	0.067373000	-2.580993000
H	5.608587000	-0.854310000	-2.909875000
H	4.981175000	0.723095000	-3.473412000
H	5.749134000	0.595630000	-1.864738000
C	2.918857000	1.061056000	-1.637049000
H	1.918592000	0.855470000	-1.202581000
H	3.454794000	1.715236000	-0.922223000
H	2.753872000	1.657023000	-2.559867000

INT1: G= -2581.607998

Al	-1.717190000	0.802358000	-1.182484000
N	-2.950167000	-0.003000000	0.197735000
N	-1.129416000	2.151957000	0.185187000

P	-0.163527000	-1.362468000	-1.776245000
C	1.075919000	-1.349478000	-0.763312000
O	2.058580000	-1.195774000	0.062187000
Si	3.711285000	-1.531907000	-0.512775000
C	-3.682511000	0.805493000	0.982594000
C	-3.350684000	2.175317000	1.168594000
H	-4.076784000	2.783925000	1.724473000
C	-2.107765000	2.782999000	0.878816000
C	-4.923381000	0.272347000	1.667265000
H	-5.753903000	0.205018000	0.932305000
H	-4.763403000	-0.753566000	2.053011000
H	-5.247051000	0.929877000	2.495599000
C	-1.888385000	4.215475000	1.323949000
H	-2.713307000	4.576269000	1.965935000
H	-0.928922000	4.347343000	1.860606000
H	-1.828306000	4.863833000	0.423191000
C	-3.257204000	-1.401845000	0.100302000
C	-4.260427000	-1.849975000	-0.804834000
C	-4.500272000	-3.236675000	-0.902666000
H	-5.271022000	-3.601481000	-1.600257000
C	-3.771194000	-4.156066000	-0.139029000
H	-3.975236000	-5.234987000	-0.228415000
C	-2.766641000	-3.700128000	0.728647000
H	-2.187292000	-4.432406000	1.310015000
C	-2.480409000	-2.327512000	0.859345000
C	-5.039365000	-0.889297000	-1.703514000
H	-4.812307000	0.147695000	-1.376987000
C	-4.560398000	-1.015838000	-3.165686000
H	-3.472993000	-0.804427000	-3.242864000
H	-4.742165000	-2.039956000	-3.556116000
H	-5.096438000	-0.296232000	-3.820405000
C	-6.565367000	-1.086784000	-1.600060000
H	-6.915762000	-1.041167000	-0.547822000
H	-7.098581000	-0.301649000	-2.175989000
H	-6.881428000	-2.068113000	-2.012976000
C	-1.380485000	-1.840342000	1.802978000
H	-0.914294000	-0.957030000	1.315086000
C	-0.267404000	-2.877575000	2.027074000
H	0.584893000	-2.413268000	2.560882000
H	-0.615298000	-3.734489000	2.642677000
H	0.113981000	-3.271361000	1.064226000
C	-1.963421000	-1.364250000	3.151004000
H	-1.151051000	-1.069356000	3.847538000
H	-2.624485000	-0.484273000	3.028581000
H	-2.549661000	-2.174584000	3.634302000
C	0.206704000	2.665157000	0.283020000
C	0.754640000	3.492791000	-0.736359000
C	2.058427000	4.001442000	-0.557779000
H	2.497865000	4.639064000	-1.341476000
C	2.798987000	3.719609000	0.597081000
H	3.809877000	4.138415000	0.723975000
C	2.252168000	2.887972000	1.586047000
H	2.837442000	2.670025000	2.493230000
C	0.967097000	2.329751000	1.444261000
C	-0.024151000	3.844988000	-2.001258000
H	-1.058063000	3.451983000	-1.877895000
C	0.581221000	3.140060000	-3.232282000

H	-0.015010000	3.359601000	-4.143040000
H	1.624917000	3.476414000	-3.412898000
H	0.594624000	2.039053000	-3.092012000
C	-0.128626000	5.367667000	-2.218830000
H	-0.549433000	5.878421000	-1.327751000
H	0.863430000	5.822402000	-2.426258000
H	-0.783373000	5.592821000	-3.086637000
C	0.399326000	1.416447000	2.532894000
H	-0.469371000	0.890033000	2.087015000
C	1.412859000	0.347048000	2.974975000
H	0.946905000	-0.349612000	3.701918000
H	1.764254000	-0.240454000	2.106254000
H	2.298002000	0.790396000	3.478130000
C	-0.118564000	2.206738000	3.753010000
H	-0.504153000	1.510968000	4.527912000
H	0.695152000	2.810138000	4.210018000
H	-0.943718000	2.893927000	3.484593000
C	4.786045000	-1.229542000	1.019214000
H	5.791764000	-1.602163000	0.709433000
C	4.309829000	-2.085976000	2.213060000
H	4.314933000	-3.170910000	1.981861000
H	4.968379000	-1.931550000	3.095701000
H	3.277672000	-1.817317000	2.518025000
C	4.924163000	0.259396000	1.396610000
H	5.445498000	0.842592000	0.610113000
H	3.937108000	0.736217000	1.559755000
H	5.509483000	0.372617000	2.335739000
C	3.753451000	-3.348825000	-1.102898000
H	3.496851000	-3.285722000	-2.185930000
C	2.700634000	-4.251974000	-0.427114000
H	1.669594000	-3.889744000	-0.617299000
H	2.770067000	-5.289477000	-0.821892000
H	2.841918000	-4.308875000	0.672778000
C	5.173421000	-3.946781000	-0.986043000
H	5.947999000	-3.325817000	-1.484418000
H	5.479155000	-4.068107000	0.075504000
H	5.212202000	-4.955823000	-1.451722000
C	3.933588000	-0.328268000	-1.965388000
H	3.273867000	-0.769478000	-2.750106000
C	5.382986000	-0.333195000	-2.493877000
H	5.723384000	-1.346559000	-2.795339000
H	5.480208000	0.323630000	-3.386095000
H	6.097140000	0.047501000	-1.731358000
C	3.419990000	1.094701000	-1.674332000
H	2.377707000	1.103676000	-1.296295000
H	4.038543000	1.618286000	-0.919126000
H	3.436013000	1.713932000	-2.597020000

TS2: G= -2581.605417 (i = -70 cm-1)

Al	-1.494644000	0.634026000	-0.913088000
N	-2.915677000	0.010469000	0.275605000
N	-1.114685000	2.184107000	0.211996000
P	-0.255990000	-1.186164000	-2.028823000
C	1.092699000	-1.553206000	-1.131375000
O	1.875738000	-1.159429000	-0.128741000

Si	3.548186000	-1.607481000	-0.480428000
C	-3.663101000	0.831634000	1.038647000
C	-3.325116000	2.195948000	1.226658000
H	-4.046821000	2.806635000	1.785676000
C	-2.096565000	2.813597000	0.904605000
C	-4.916863000	0.302891000	1.698569000
H	-5.724767000	0.196978000	0.943611000
H	-4.751782000	-0.707750000	2.121447000
H	-5.273717000	0.981520000	2.495343000
C	-1.861048000	4.232138000	1.372887000
H	-2.795261000	4.695174000	1.741696000
H	-1.109569000	4.263066000	2.187085000
H	-1.445533000	4.845352000	0.547362000
C	-3.230954000	-1.391518000	0.174853000
C	-4.232028000	-1.832982000	-0.731965000
C	-4.480498000	-3.218244000	-0.820947000
H	-5.249586000	-3.584686000	-1.519058000
C	-3.752749000	-4.135591000	-0.052661000
H	-3.961403000	-5.213927000	-0.136791000
C	-2.738305000	-3.683061000	0.805137000
H	-2.153867000	-4.416125000	1.380039000
C	-2.446105000	-2.311291000	0.928424000
C	-4.968747000	-0.866775000	-1.658719000
H	-4.765745000	0.167410000	-1.309328000
C	-4.401848000	-0.978620000	-3.091258000
H	-3.307497000	-0.789938000	-3.104033000
H	-4.570489000	-1.996207000	-3.503195000
H	-4.889533000	-0.245865000	-3.768652000
C	-6.496509000	-1.069488000	-1.645720000
H	-6.908322000	-1.032606000	-0.615394000
H	-6.997649000	-0.282054000	-2.246532000
H	-6.784524000	-2.048179000	-2.084224000
C	-1.331672000	-1.818284000	1.852706000
H	-0.867786000	-0.932669000	1.361671000
C	-0.209770000	-2.847494000	2.054346000
H	0.638740000	-2.385017000	2.596176000
H	-0.547193000	-3.718867000	2.654909000
H	0.173447000	-3.214468000	1.082600000
C	-1.904528000	-1.345844000	3.205978000
H	-1.090224000	-1.026137000	3.888644000
H	-2.594163000	-0.486751000	3.088007000
H	-2.462340000	-2.168956000	3.701160000
C	0.214376000	2.738854000	0.233049000
C	0.737431000	3.400160000	-0.912980000
C	2.055518000	3.898266000	-0.851783000
H	2.479192000	4.404800000	-1.733410000
C	2.832932000	3.762352000	0.304996000
H	3.859467000	4.160328000	0.332252000
C	2.301852000	3.108332000	1.426108000
H	2.918491000	2.998103000	2.332200000
C	0.998286000	2.575353000	1.413326000
C	-0.083496000	3.600654000	-2.185501000
H	-1.092633000	3.167667000	-2.003708000
C	0.529160000	2.838737000	-3.377872000
H	-0.104946000	2.954555000	-4.281925000
H	1.544116000	3.218316000	-3.622273000
H	0.615336000	1.754833000	-3.156065000

C	-0.278494000	5.096552000	-2.506791000
H	-0.735886000	5.640744000	-1.654272000
H	0.688369000	5.591157000	-2.739824000
H	-0.939614000	5.223079000	-3.389706000
C	0.483323000	1.816514000	2.639254000
H	-0.570411000	1.526607000	2.450160000
C	1.282736000	0.514953000	2.834281000
H	0.846154000	-0.097532000	3.650015000
H	1.298665000	-0.088199000	1.904555000
H	2.334648000	0.730265000	3.112852000
C	0.510098000	2.669721000	3.923359000
H	0.069923000	2.104993000	4.771738000
H	1.548376000	2.941567000	4.208842000
H	-0.062657000	3.613340000	3.811564000
C	4.525542000	-1.195791000	1.103326000
H	5.543830000	-1.604820000	0.898581000
C	3.958282000	-1.947119000	2.326467000
H	3.986514000	-3.047887000	2.189906000
H	4.540614000	-1.711638000	3.245075000
H	2.903439000	-1.668962000	2.522769000
C	4.670861000	0.316749000	1.360888000
H	5.290762000	0.811451000	0.585110000
H	3.689949000	0.830520000	1.368476000
H	5.156564000	0.509673000	2.343970000
C	3.662167000	-3.472224000	-0.896695000
H	3.486076000	-3.510088000	-1.996256000
C	2.575647000	-4.336263000	-0.227414000
H	1.564687000	-4.008966000	-0.547151000
H	2.696779000	-5.405878000	-0.509660000
H	2.619456000	-4.281577000	0.881053000
C	5.077149000	-4.025402000	-0.617773000
H	5.879390000	-3.430128000	-1.103545000
H	5.298607000	-4.051147000	0.470890000
H	5.171856000	-5.069233000	-0.990779000
C	4.010858000	-0.532607000	-1.986709000
H	3.461397000	-1.037787000	-2.816030000
C	5.518075000	-0.572086000	-2.305647000
H	5.889031000	-1.605047000	-2.474732000
H	5.748380000	0.012912000	-3.223714000
H	6.121670000	-0.132834000	-1.481534000
C	3.472718000	0.909188000	-1.890488000
H	2.394218000	0.939429000	-1.634486000
H	4.001833000	1.504016000	-1.119281000
H	3.598291000	1.443457000	-2.858058000

TS3: G= -2581.603912 (i = -135 cm-1)

Al	-1.630442000	0.993937000	-1.183073000
N	-2.759730000	0.150137000	0.227333000
N	-0.861975000	2.253691000	0.147218000
P	-0.091819000	-0.733272000	-2.175895000
C	0.758167000	-0.803362000	-0.799811000
O	1.653852000	-1.051356000	0.105530000
Si	3.154399000	-1.830292000	-0.485424000
C	-3.490309000	1.025035000	0.935696000
C	-3.111369000	2.395472000	1.047667000

H	-3.836346000	3.058573000	1.540327000
C	-1.826737000	2.940648000	0.803702000
C	-4.782568000	0.592366000	1.593871000
H	-5.626226000	0.810575000	0.903096000
H	-4.797054000	-0.495053000	1.797884000
H	-4.966613000	1.148478000	2.533182000
C	-1.568737000	4.374461000	1.221391000
H	-2.242363000	4.697887000	2.037250000
H	-0.517874000	4.543088000	1.523041000
H	-1.756210000	5.028591000	0.340797000
C	-3.073047000	-1.247542000	0.172582000
C	-4.031187000	-1.724653000	-0.765806000
C	-4.251199000	-3.116137000	-0.847558000
H	-4.988813000	-3.502398000	-1.569268000
C	-3.544881000	-4.011947000	-0.037213000
H	-3.727556000	-5.095329000	-0.118761000
C	-2.595280000	-3.526187000	0.876228000
H	-2.041620000	-4.241362000	1.501546000
C	-2.337227000	-2.147308000	0.999653000
C	-4.805376000	-0.791141000	-1.695875000
H	-4.538984000	0.254926000	-1.432895000
C	-4.381930000	-1.001324000	-3.164216000
H	-3.290762000	-0.840573000	-3.286376000
H	-4.619153000	-2.030803000	-3.508593000
H	-4.909561000	-0.286864000	-3.830917000
C	-6.331068000	-0.942439000	-1.525775000
H	-6.637692000	-0.813464000	-0.467306000
H	-6.868827000	-0.187084000	-2.136571000
H	-6.682253000	-1.944039000	-1.853581000
C	-1.311832000	-1.616683000	2.000745000
H	-0.759092000	-0.803243000	1.478260000
C	-0.285166000	-2.669156000	2.442742000
H	0.483572000	-2.200081000	3.085690000
H	-0.753768000	-3.480518000	3.039637000
H	0.232717000	-3.124240000	1.578543000
C	-1.992399000	-0.997479000	3.242569000
H	-1.229942000	-0.673221000	3.981189000
H	-2.600086000	-0.108436000	2.990479000
H	-2.652730000	-1.740525000	3.738024000
C	0.503796000	2.677100000	0.251167000
C	1.112587000	3.429379000	-0.791103000
C	2.446108000	3.856956000	-0.622082000
H	2.928242000	4.441062000	-1.422211000
C	3.167690000	3.548803000	0.537919000
H	4.208280000	3.891765000	0.651493000
C	2.561889000	2.787799000	1.549267000
H	3.134431000	2.545177000	2.458328000
C	1.233274000	2.335611000	1.430125000
C	0.365763000	3.786361000	-2.072811000
H	-0.677900000	3.411318000	-1.969912000
C	0.982827000	3.063492000	-3.287014000
H	0.389604000	3.260007000	-4.204841000
H	2.025086000	3.402252000	-3.471521000
H	1.007507000	1.966178000	-3.122649000
C	0.287723000	5.309896000	-2.292846000
H	-0.163336000	5.820919000	-1.416849000
H	1.293604000	5.752186000	-2.457536000

H	-0.329464000	5.544899000	-3.185380000
C	0.582719000	1.547799000	2.567734000
H	-0.319033000	1.060095000	2.144211000
C	1.499460000	0.441229000	3.111966000
H	0.972706000	-0.145922000	3.891965000
H	1.798583000	-0.245881000	2.299274000
H	2.416182000	0.850305000	3.586258000
C	0.118193000	2.469514000	3.715429000
H	-0.344504000	1.870381000	4.528212000
H	0.978339000	3.025345000	4.146898000
H	-0.630790000	3.211458000	3.379699000
C	4.260457000	-1.980412000	1.048456000
H	5.116883000	-2.590297000	0.671939000
C	3.577858000	-2.774443000	2.181827000
H	3.233273000	-3.775614000	1.850246000
H	4.276864000	-2.927259000	3.033113000
H	2.691948000	-2.235563000	2.573014000
C	4.822133000	-0.632087000	1.546568000
H	5.470342000	-0.144748000	0.790322000
H	4.015142000	0.085076000	1.800077000
H	5.435870000	-0.779037000	2.462648000
C	2.673463000	-3.518770000	-1.243452000
H	2.430905000	-3.266893000	-2.301861000
C	1.428223000	-4.189552000	-0.630376000
H	0.533012000	-3.535882000	-0.667164000
H	1.179673000	-5.121215000	-1.184663000
H	1.597167000	-4.479382000	0.428168000
C	3.879246000	-4.485732000	-1.248282000
H	4.785012000	-4.050321000	-1.720357000
H	4.157886000	-4.791807000	-0.216918000
H	3.633610000	-5.415217000	-1.807247000
C	3.814297000	-0.666375000	-1.840905000
H	3.148842000	-0.881135000	-2.709394000
C	5.258076000	-1.030399000	-2.247735000
H	5.355560000	-2.085772000	-2.578564000
H	5.600944000	-0.390628000	-3.090382000
H	5.973588000	-0.875027000	-1.411404000
C	3.663189000	0.827696000	-1.501063000
H	2.621666000	1.099655000	-1.238839000
H	4.299392000	1.132138000	-0.646400000
H	3.953720000	1.459612000	-2.368531000

2: G= -2581.703351

Al	-0.306384000	-0.755414000	-0.588844000
P	0.169185000	0.918514000	-2.122219000
C	0.437852000	1.441561000	-0.466063000
O	0.190354000	0.582822000	0.573251000
Si	1.171776000	3.123036000	0.057540000
C	3.003390000	2.768825000	0.533611000
H	3.434399000	3.757613000	0.822100000
C	3.793122000	2.219085000	-0.671739000
H	3.767321000	2.897886000	-1.549938000
H	4.861597000	2.052674000	-0.407499000
H	3.383555000	1.238598000	-0.997562000
C	3.115026000	1.809541000	1.731677000

H	2.594458000	2.188082000	2.636261000
H	2.674120000	0.823343000	1.483032000
H	4.178990000	1.630969000	2.006198000
C	1.126807000	4.332236000	-1.430354000
H	1.492083000	3.698106000	-2.273277000
C	-0.294768000	4.805105000	-1.789743000
H	-1.001929000	3.955298000	-1.888948000
H	-0.696944000	5.493946000	-1.016118000
H	-0.299671000	5.361270000	-2.753913000
C	2.093141000	5.523331000	-1.273005000
H	3.141154000	5.200753000	-1.100310000
H	2.091202000	6.161699000	-2.185258000
H	1.804847000	6.176591000	-0.421291000
C	0.167562000	3.673895000	1.596035000
H	0.411689000	2.877636000	2.337918000
C	-1.357738000	3.638927000	1.371290000
H	-1.698671000	2.674745000	0.940330000
H	-1.904624000	3.780968000	2.330268000
H	-1.688468000	4.444711000	0.684249000
C	0.633771000	5.024316000	2.171195000
H	1.728019000	5.047386000	2.362811000
H	0.401330000	5.860429000	1.476330000
H	0.123026000	5.253776000	3.133806000
N	0.710125000	-2.350923000	-0.310868000
N	-2.009131000	-1.494914000	-0.181824000
C	0.164518000	-3.578766000	-0.194652000
C	-1.236026000	-3.795583000	-0.179530000
H	-1.559298000	-4.843955000	-0.120956000
C	-2.256275000	-2.821479000	-0.091851000
C	1.055385000	-4.786282000	-0.003726000
H	0.706440000	-5.625641000	-0.637789000
H	2.114220000	-4.565886000	-0.232827000
H	1.001553000	-5.128881000	1.051019000
C	-3.677138000	-3.276004000	0.137065000
H	-3.750322000	-4.376153000	0.212227000
H	-4.082482000	-2.822343000	1.064255000
H	-4.332328000	-2.924611000	-0.685136000
C	2.114442000	-2.115877000	-0.072053000
C	2.976254000	-1.861053000	-1.176331000
C	4.317126000	-1.522683000	-0.903404000
H	5.001404000	-1.317345000	-1.740167000
C	4.793166000	-1.437437000	0.412431000
H	5.841380000	-1.157962000	0.602333000
C	3.931390000	-1.700878000	1.484924000
H	4.308516000	-1.622767000	2.516888000
C	2.580699000	-2.041407000	1.270831000
C	2.499193000	-2.025516000	-2.618592000
H	1.395702000	-1.899629000	-2.619628000
C	3.075998000	-0.965626000	-3.572525000
H	2.588423000	-1.041572000	-4.566383000
H	2.896496000	0.057060000	-3.183006000
H	4.167254000	-1.098232000	-3.732621000
C	2.800635000	-3.453528000	-3.123479000
H	2.443155000	-3.585468000	-4.166635000
H	3.893629000	-3.652687000	-3.107396000
H	2.308418000	-4.223270000	-2.495765000
C	1.669310000	-2.301819000	2.470026000

H	0.672404000	-2.601065000	2.085612000
C	1.456805000	-1.024793000	3.305799000
H	0.772620000	-1.230328000	4.155746000
H	2.415445000	-0.653920000	3.726185000
H	1.018605000	-0.219593000	2.685537000
C	2.199130000	-3.460122000	3.341055000
H	1.472489000	-3.708697000	4.142806000
H	2.381856000	-4.375698000	2.741780000
H	3.156873000	-3.189525000	3.833861000
C	-3.047884000	-0.519121000	0.057319000
C	-3.833149000	-0.038447000	-1.029301000
C	-4.779635000	0.973494000	-0.768112000
H	-5.394501000	1.359856000	-1.596362000
C	-4.945887000	1.500853000	0.519109000
H	-5.686965000	2.295359000	0.700179000
C	-4.161277000	1.017829000	1.575016000
H	-4.294372000	1.439090000	2.583937000
C	-3.198105000	0.008812000	1.372337000
C	-3.694990000	-0.580869000	-2.452443000
H	-2.877460000	-1.332205000	-2.451104000
C	-3.303850000	0.529074000	-3.449316000
H	-3.136803000	0.101959000	-4.460596000
H	-4.104443000	1.294155000	-3.537061000
H	-2.370952000	1.039173000	-3.132288000
C	-4.986179000	-1.289959000	-2.917092000
H	-5.295845000	-2.096824000	-2.221007000
H	-5.832596000	-0.574563000	-2.989646000
H	-4.843275000	-1.742275000	-3.920881000
C	-2.384671000	-0.500257000	2.564655000
H	-1.595109000	-1.179020000	2.178363000
C	-1.674791000	0.646044000	3.308835000
H	-0.964864000	1.153979000	2.629911000
H	-2.395615000	1.395967000	3.696050000
H	-1.108942000	0.250844000	4.176547000
C	-3.273619000	-1.317950000	3.526558000
H	-3.770720000	-2.166314000	3.012790000
H	-2.669489000	-1.732373000	4.361047000
H	-4.069879000	-0.681481000	3.967766000

3: G= -2581.672909

Al	-0.912408000	0.545759000	-0.462861000
N	-2.671737000	0.224569000	0.215566000
N	-0.785896000	2.362711000	0.101635000
P	0.179787000	-0.598150000	-2.131643000
C	0.589989000	-0.614883000	-0.415586000
O	1.581225000	-1.227858000	0.258835000
Si	2.943958000	-2.040752000	-0.465600000
C	-3.497716000	1.192667000	0.664637000
C	-3.116560000	2.556496000	0.739732000
H	-3.890731000	3.255054000	1.083813000
C	-1.827407000	3.102070000	0.539166000
C	-4.891686000	0.819771000	1.116972000
H	-5.338060000	1.615654000	1.741323000
H	-5.550729000	0.660786000	0.237884000
H	-4.886070000	-0.133593000	1.681714000

C	-1.592771000	4.558428000	0.863971000
H	-2.517690000	5.057646000	1.206044000
H	-0.815868000	4.662981000	1.648459000
H	-1.200158000	5.089838000	-0.027175000
C	-3.077412000	-1.159738000	0.209016000
C	-3.925541000	-1.647290000	-0.822373000
C	-4.264374000	-3.016205000	-0.809175000
H	-4.918766000	-3.416287000	-1.599760000
C	-3.768324000	-3.877335000	0.177807000
H	-4.044434000	-4.943702000	0.169710000
C	-2.900105000	-3.385299000	1.164273000
H	-2.496658000	-4.077317000	1.917682000
C	-2.527974000	-2.027018000	1.198845000
C	-4.410317000	-0.759626000	-1.968561000
H	-4.179968000	0.294393000	-1.708688000
C	-3.631675000	-1.087056000	-3.261103000
H	-2.539132000	-0.976151000	-3.105745000
H	-3.825546000	-2.132337000	-3.583345000
H	-3.938862000	-0.411351000	-4.087415000
C	-5.931689000	-0.861706000	-2.197626000
H	-6.505160000	-0.681771000	-1.264330000
H	-6.260646000	-0.118874000	-2.954020000
H	-6.221877000	-1.864425000	-2.576335000
C	-1.558389000	-1.496642000	2.256457000
H	-0.918805000	-0.740022000	1.746944000
C	-0.595322000	-2.568873000	2.786498000
H	0.173742000	-2.098971000	3.431559000
H	-1.112756000	-3.336357000	3.400835000
H	-0.070295000	-3.075795000	1.952601000
C	-2.292890000	-0.780867000	3.408429000
H	-1.564250000	-0.396816000	4.153394000
H	-2.883552000	0.085458000	3.049136000
H	-2.983966000	-1.476314000	3.931027000
C	0.565206000	2.857463000	0.222125000
C	1.199984000	3.495295000	-0.876352000
C	2.535715000	3.918938000	-0.717363000
H	3.047578000	4.412802000	-1.558406000
C	3.227440000	3.707435000	0.482338000
H	4.273047000	4.038584000	0.582839000
C	2.591311000	3.055708000	1.548912000
H	3.146174000	2.877740000	2.483933000
C	1.257365000	2.615295000	1.444809000
C	0.488405000	3.709992000	-2.210301000
H	-0.550381000	3.333730000	-2.097977000
C	1.158025000	2.888866000	-3.331111000
H	0.606191000	3.008493000	-4.287324000
H	2.205462000	3.218288000	-3.500350000
H	1.173473000	1.808576000	-3.075625000
C	0.407669000	5.204300000	-2.585335000
H	-0.066251000	5.806270000	-1.781982000
H	1.416558000	5.631168000	-2.768385000
H	-0.185559000	5.342956000	-3.513452000
C	0.597151000	1.904803000	2.628362000
H	-0.408781000	1.563602000	2.306060000
C	1.385816000	0.651478000	3.053670000
H	0.850120000	0.121339000	3.868848000
H	1.514073000	-0.048805000	2.204490000

H	2.391253000	0.917112000	3.444386000
C	0.389750000	2.862973000	3.820273000
H	-0.129563000	2.341988000	4.652261000
H	1.361276000	3.235618000	4.209321000
H	-0.220698000	3.745892000	3.540013000
C	4.007254000	-2.487812000	1.055121000
H	4.828988000	-3.130447000	0.658525000
C	3.198100000	-3.312436000	2.077128000
H	2.794006000	-4.249495000	1.640881000
H	3.826780000	-3.596442000	2.950510000
H	2.336201000	-2.728359000	2.460486000
C	4.643989000	-1.253287000	1.725017000
H	5.346565000	-0.724977000	1.049037000
H	3.871092000	-0.521660000	2.040104000
H	5.215397000	-1.545227000	2.634575000
C	2.299158000	-3.589559000	-1.386065000
H	2.078184000	-3.214292000	-2.412435000
C	0.978921000	-4.137436000	-0.803034000
H	0.182285000	-3.365954000	-0.775587000
H	0.603243000	-4.985872000	-1.417228000
H	1.111612000	-4.519432000	0.231526000
C	3.370321000	-4.695662000	-1.494911000
H	4.317308000	-4.333164000	-1.948592000
H	3.622971000	-5.117941000	-0.498005000
H	3.007588000	-5.540219000	-2.122090000
C	3.795302000	-0.812577000	-1.658174000
H	3.162135000	-0.860961000	-2.574317000
C	5.218294000	-1.270849000	-2.040242000
H	5.229081000	-2.286083000	-2.490904000
H	5.672231000	-0.578387000	-2.783828000
H	5.897636000	-1.292162000	-1.160491000
C	3.767406000	0.646141000	-1.159275000
H	2.746767000	0.980094000	-0.884358000
H	4.408560000	0.798128000	-0.267241000
H	4.135812000	1.339798000	-1.947210000

E: G= -2124.745857

Mg	0.031936000	0.737136000	-0.193676000
C	0.072533000	2.864502000	-0.199923000
P	0.023396000	4.448801000	-0.230954000
N	1.527319000	-0.424364000	-0.999908000
N	-1.465682000	-0.324840000	-1.165669000
C	1.358093000	-0.956434000	-2.227729000
C	0.099494000	-1.100694000	-2.866572000
H	0.135892000	-1.535093000	-3.875775000
C	-1.208323000	-0.944363000	-2.327067000
C	2.576261000	-1.470866000	-2.975161000
H	3.290940000	-0.643486000	-3.161272000
H	2.303801000	-1.930844000	-3.942986000
H	3.126249000	-2.217948000	-2.366557000
C	-2.336631000	-1.582067000	-3.121709000
H	-2.323160000	-2.683549000	-2.982488000
H	-2.203323000	-1.390634000	-4.205327000
H	-3.332181000	-1.214766000	-2.807829000
C	2.793281000	-0.551148000	-0.351420000

C	3.804080000	0.438333000	-0.516759000
C	5.046264000	0.246072000	0.124779000
H	5.842314000	0.996654000	-0.008199000
C	5.282818000	-0.874104000	0.931522000
H	6.263439000	-1.013895000	1.413709000
C	4.251713000	-1.802124000	1.150820000
H	4.433062000	-2.654324000	1.822619000
C	2.992021000	-1.654306000	0.537135000
C	3.554479000	1.718841000	-1.311229000
H	2.523017000	1.662963000	-1.720514000
C	4.528343000	1.886690000	-2.494423000
H	5.577737000	1.990790000	-2.144820000
H	4.279532000	2.797215000	-3.078795000
H	4.493202000	1.019235000	-3.185385000
C	3.613152000	2.942353000	-0.373509000
H	2.902510000	2.830245000	0.469834000
H	3.330224000	3.868127000	-0.915837000
H	4.633720000	3.081647000	0.043200000
C	1.837525000	-2.609644000	0.851949000
H	0.914887000	-1.986901000	0.845966000
C	1.640528000	-3.698542000	-0.224609000
H	1.360567000	-3.265431000	-1.203800000
H	0.825504000	-4.392203000	0.072483000
H	2.567082000	-4.297058000	-0.355701000
C	1.964282000	-3.254448000	2.243779000
H	2.787839000	-3.998847000	2.281013000
H	1.031748000	-3.792502000	2.506093000
H	2.156751000	-2.499268000	3.033645000
C	-2.738402000	-0.428120000	-0.533866000
C	-3.581664000	0.725182000	-0.471078000
C	-4.785445000	0.639589000	0.256191000
H	-5.447531000	1.516987000	0.310435000
C	-5.159000000	-0.544398000	0.912100000
H	-6.106034000	-0.591216000	1.472873000
C	-4.317121000	-1.663250000	0.855075000
H	-4.606933000	-2.585418000	1.384735000
C	-3.097992000	-1.629327000	0.146367000
C	-3.189450000	2.007661000	-1.198050000
H	-2.079913000	2.073296000	-1.172927000
C	-3.597428000	1.950051000	-2.685047000
H	-3.131250000	1.086953000	-3.199740000
H	-3.277582000	2.872494000	-3.213860000
H	-4.699863000	1.855726000	-2.788432000
C	-3.710009000	3.287454000	-0.527843000
H	-4.808793000	3.407771000	-0.642722000
H	-3.228754000	4.175073000	-0.987905000
H	-3.470307000	3.304346000	0.555577000
C	-2.174382000	-2.846535000	0.185663000
H	-1.289726000	-2.628803000	-0.447712000
C	-2.855030000	-4.111882000	-0.374042000
H	-3.692173000	-4.446022000	0.275394000
H	-2.129309000	-4.949874000	-0.438023000
H	-3.270221000	-3.936830000	-1.387251000
C	-1.655273000	-3.088371000	1.617702000
H	-1.070501000	-2.219795000	1.980341000
H	-0.996407000	-3.980698000	1.647928000
H	-2.490437000	-3.264873000	2.327927000

O	-0.156702000	0.213636000	1.825729000
O	-0.191425000	2.099171000	3.947852000
C	1.010075000	0.410046000	2.676103000
H	1.900109000	0.200988000	2.048018000
H	0.962105000	-0.339393000	3.497029000
C	1.010080000	1.833054000	3.225653000
H	1.858517000	1.969571000	3.927161000
H	1.124310000	2.549416000	2.375386000
C	-1.328893000	1.928245000	3.101300000
H	-1.295935000	2.646151000	2.245752000
H	-2.232063000	2.136144000	3.710883000
C	-1.387102000	0.508121000	2.552860000
H	-1.481139000	-0.236109000	3.374335000
H	-2.219383000	0.364874000	1.836558000

N_3TMS G= -573.588435

N	3.107902000	0.000048000	-0.026694000
N	2.014747000	-0.000067000	0.349823000
N	0.895015000	-0.000223000	0.843054000
Si	-0.683695000	-0.000007000	-0.022057000
C	-0.774472000	1.556225000	-1.083062000
H	-0.679114000	2.468906000	-0.459059000
H	0.035649000	1.568295000	-1.842341000
H	-1.745936000	1.603500000	-1.619967000
C	-0.773563000	-1.554644000	-1.085485000
H	-1.744499000	-1.600936000	-1.623438000
H	0.037279000	-1.565525000	-1.844012000
H	-0.678888000	-2.468343000	-0.462878000
C	-1.964424000	-0.001411000	1.350438000
H	-1.856331000	0.897018000	1.992577000
H	-2.989041000	-0.000999000	0.922550000
H	-1.856291000	-0.901141000	1.990744000

TS-[2+3]: G= -3155.304455 (i = -232 cm-1)

Al	0.270385000	0.119306000	-0.458275000
C	-1.673129000	0.412185000	-0.446900000
P	-2.951223000	0.644655000	-1.386847000
N	-2.602140000	0.352956000	1.798559000
N	-3.705320000	0.487080000	1.428205000
N	-4.519826000	0.694513000	0.511768000
N	0.948134000	1.592465000	-1.500912000
N	0.537092000	-1.262444000	-1.773020000
O	1.279334000	-0.181948000	0.911675000
Si	1.374479000	-0.524991000	2.549754000
C	0.755224000	1.575162000	-2.833441000
C	0.400113000	0.397675000	-3.543202000
H	0.242124000	0.525473000	-4.622596000
C	0.404278000	-0.941016000	-3.077511000
C	0.904842000	2.849994000	-3.634940000
H	1.265655000	2.642955000	-4.660261000
H	-0.088163000	3.341481000	-3.719295000
H	1.587849000	3.568034000	-3.142610000
C	0.287883000	-2.048967000	-4.099898000
H	0.328196000	-1.656206000	-5.132330000
H	1.097218000	-2.794737000	-3.963729000

H	-0.666330000	-2.598197000	-3.964865000
C	1.451400000	2.753352000	-0.808998000
C	0.573198000	3.803255000	-0.417953000
C	1.099519000	4.861150000	0.354111000
H	0.428573000	5.672877000	0.677010000
C	2.449847000	4.896221000	0.715539000
H	2.840232000	5.727915000	1.323131000
C	3.308506000	3.867243000	0.299539000
H	4.369265000	3.911682000	0.584207000
C	2.834797000	2.778706000	-0.458333000
C	-0.901724000	3.840508000	-0.812945000
H	-1.103430000	2.957085000	-1.449765000
C	-1.829686000	3.726455000	0.413054000
H	-2.892164000	3.692358000	0.091320000
H	-1.629757000	2.799238000	0.982091000
H	-1.702650000	4.591741000	1.098617000
C	-1.234917000	5.103375000	-1.635329000
H	-2.275647000	5.050862000	-2.019044000
H	-1.154627000	6.024105000	-1.019123000
H	-0.552599000	5.224026000	-2.501449000
C	3.782487000	1.671742000	-0.921430000
H	3.239938000	0.711894000	-0.778991000
C	5.086936000	1.598661000	-0.110231000
H	5.639302000	0.670778000	-0.365153000
H	5.759742000	2.452658000	-0.339333000
H	4.899598000	1.590179000	0.981046000
C	4.120971000	1.807502000	-2.423617000
H	4.893818000	1.066237000	-2.714350000
H	3.243032000	1.632957000	-3.073430000
H	4.521310000	2.819983000	-2.643417000
C	0.706432000	-2.638430000	-1.373907000
C	-0.426905000	-3.486316000	-1.221914000
C	-0.218194000	-4.821132000	-0.815355000
H	-1.087507000	-5.484648000	-0.684607000
C	1.066729000	-5.307545000	-0.556850000
H	1.211666000	-6.351711000	-0.237226000
C	2.172585000	-4.454107000	-0.688138000
H	3.173750000	-4.849254000	-0.467201000
C	2.024402000	-3.110607000	-1.088929000
C	-1.857094000	-2.990637000	-1.417719000
H	-1.810640000	-1.979957000	-1.869183000
C	-2.558859000	-2.837274000	-0.052433000
H	-3.573878000	-2.406914000	-0.180125000
H	-2.654243000	-3.819170000	0.458638000
H	-1.995672000	-2.155493000	0.613538000
C	-2.682729000	-3.887177000	-2.361085000
H	-3.672984000	-3.425344000	-2.558476000
H	-2.176010000	-4.040762000	-3.336895000
H	-2.869665000	-4.890520000	-1.922988000
C	3.240796000	-2.185555000	-1.191061000
H	2.960591000	-1.262096000	-0.635674000
C	3.581894000	-1.785726000	-2.644156000
H	4.539673000	-1.226038000	-2.667215000
H	3.704229000	-2.686747000	-3.282389000
H	2.816362000	-1.131113000	-3.100183000
C	4.501140000	-2.762094000	-0.524674000
H	5.293037000	-1.987230000	-0.482758000

H	4.310342000	-3.100892000	0.512248000
H	4.910270000	-3.620312000	-1.099996000
C	-0.154401000	-1.595395000	2.990319000
H	-0.947901000	-1.131422000	2.363038000
C	-0.633479000	-1.511689000	4.450683000
H	-0.873227000	-0.469799000	4.750945000
H	-1.555557000	-2.117534000	4.603785000
H	0.125938000	-1.895964000	5.166633000
C	0.020992000	-3.052390000	2.519313000
H	0.355593000	-3.113066000	1.462080000
H	0.767323000	-3.598938000	3.136197000
H	-0.934839000	-3.618069000	2.590114000
C	1.349716000	1.126613000	3.520720000
H	1.146746000	0.845075000	4.581023000
C	0.195418000	2.014383000	3.016385000
H	-0.784720000	1.495539000	3.013345000
H	0.093194000	2.936129000	3.632264000
H	0.395528000	2.348839000	1.975843000
C	2.675721000	1.908632000	3.469489000
H	3.508191000	1.367851000	3.963937000
H	2.975371000	2.117147000	2.419835000
H	2.576645000	2.896319000	3.974690000
C	2.966896000	-1.547618000	2.836866000
H	2.767664000	-2.474355000	2.246472000
C	3.141899000	-1.946416000	4.315262000
H	2.267509000	-2.504651000	4.709894000
H	4.037056000	-2.593200000	4.458586000
H	3.282254000	-1.053452000	4.964002000
C	4.244886000	-0.908638000	2.271514000
H	4.097711000	-0.567362000	1.229784000
H	4.562044000	-0.028018000	2.866014000
H	5.096650000	-1.624746000	2.277378000
Si	-6.283103000	0.300543000	0.493415000
C	-6.455180000	-1.569546000	0.333541000
H	-5.986192000	-1.921913000	-0.608564000
H	-5.959332000	-2.087719000	1.180689000
H	-7.524490000	-1.870496000	0.322029000
C	-7.047830000	0.940312000	2.090941000
H	-8.138855000	0.732531000	2.110701000
H	-6.588621000	0.446615000	2.972771000
H	-6.900521000	2.035649000	2.189723000
C	-6.916769000	1.193803000	-1.032188000
H	-6.380983000	0.846089000	-1.939799000
H	-8.000686000	0.999564000	-1.174202000
H	-6.767154000	2.289320000	-0.940937000

TS′-[2+3]: G= -3155.291834 (i = -206 cm-1)

Al	-0.057171000	0.100601000	-0.631554000
C	-1.725469000	0.215339000	-1.710429000
P	-2.058680000	0.460708000	-3.272232000
N	-4.010817000	0.232553000	-0.883428000
N	-4.517146000	0.457750000	-1.994467000
N	-4.313357000	0.640173000	-3.154042000
N	0.949123000	1.669867000	-1.188187000
N	1.089091000	-1.131988000	-1.599953000

O	-0.147295000	-0.207807000	1.063547000
Si	0.111171000	-0.619012000	2.669665000
C	1.180548000	1.726944000	-2.522648000
C	1.304565000	0.564770000	-3.320301000
H	1.452525000	0.727073000	-4.396103000
C	1.395311000	-0.776205000	-2.868226000
C	1.214233000	3.072070000	-3.211094000
H	1.719012000	3.026871000	-4.194110000
H	0.158376000	3.383645000	-3.378243000
H	1.685527000	3.854555000	-2.587478000
C	1.807291000	-1.838008000	-3.861951000
H	2.196030000	-1.391698000	-4.795523000
H	2.570147000	-2.519887000	-3.438367000
H	0.927556000	-2.470078000	-4.110749000
C	1.187930000	2.838729000	-0.378079000
C	0.112274000	3.706194000	-0.046238000
C	0.364835000	4.794609000	0.813528000
H	-0.465314000	5.461406000	1.095060000
C	1.651343000	5.043050000	1.305295000
H	1.832913000	5.896306000	1.977892000
C	2.714143000	4.208016000	0.928484000
H	3.723223000	4.428799000	1.305625000
C	2.515625000	3.096065000	0.084574000
C	-1.288463000	3.492871000	-0.609913000
H	-1.212120000	2.746243000	-1.424901000
C	-2.238343000	2.897274000	0.444574000
H	-3.234037000	2.705399000	-0.001155000
H	-1.858767000	1.926925000	0.825170000
H	-2.357204000	3.579834000	1.313229000
C	-1.873623000	4.774435000	-1.233269000
H	-2.827274000	4.543972000	-1.752688000
H	-2.093158000	5.549434000	-0.468163000
H	-1.176272000	5.219109000	-1.973634000
C	3.702403000	2.217775000	-0.328452000
H	3.323468000	1.174265000	-0.370056000
C	4.880686000	2.270259000	0.663406000
H	5.589304000	1.443735000	0.448197000
H	5.450377000	3.218695000	0.563027000
H	4.558589000	2.178430000	1.717515000
C	4.240935000	2.560270000	-1.737544000
H	5.185750000	2.009150000	-1.927922000
H	3.539106000	2.278413000	-2.542350000
H	4.457154000	3.646206000	-1.823817000
C	1.389881000	-2.473171000	-1.163045000
C	0.441012000	-3.511767000	-1.375444000
C	0.769333000	-4.817218000	-0.957691000
H	0.041423000	-5.629687000	-1.105648000
C	1.997053000	-5.090677000	-0.343561000
H	2.237774000	-6.115158000	-0.018381000
C	2.916488000	-4.053468000	-0.127882000
H	3.871078000	-4.284753000	0.365399000
C	2.641428000	-2.729364000	-0.528188000
C	-0.935271000	-3.220572000	-1.964601000
H	-0.880557000	-2.251648000	-2.499854000
C	-1.955951000	-3.033760000	-0.823413000
H	-2.936250000	-2.700055000	-1.220849000
H	-2.093611000	-3.979454000	-0.256411000

H	-1.616457000	-2.262667000	-0.103536000
C	-1.411255000	-4.273308000	-2.980498000
H	-2.351487000	-3.935557000	-3.463719000
H	-0.656659000	-4.442691000	-3.777134000
H	-1.623193000	-5.253514000	-2.502839000
C	3.659816000	-1.610771000	-0.284794000
H	3.077250000	-0.728993000	0.061757000
C	4.407605000	-1.180617000	-1.567873000
H	5.215956000	-0.463891000	-1.312790000
H	4.875611000	-2.055480000	-2.066572000
H	3.747565000	-0.670829000	-2.294272000
C	4.691381000	-1.948495000	0.805911000
H	5.299474000	-1.050788000	1.037932000
H	4.213474000	-2.288704000	1.746743000
H	5.396964000	-2.739034000	0.472718000
C	-0.742158000	-2.303330000	3.006473000
H	-1.721190000	-2.211374000	2.490096000
C	-1.030896000	-2.603429000	4.490594000
H	-1.648828000	-1.816232000	4.971045000
H	-1.578149000	-3.566665000	4.602834000
H	-0.098293000	-2.692884000	5.086483000
C	0.022140000	-3.457363000	2.328653000
H	0.269566000	-3.233364000	1.272026000
H	0.980366000	-3.679766000	2.844880000
H	-0.572883000	-4.397864000	2.333570000
C	-0.600439000	0.770725000	3.782522000
H	-0.298939000	0.487439000	4.819901000
C	-2.139963000	0.832523000	3.738051000
H	-2.618084000	-0.128905000	4.014144000
H	-2.531918000	1.611416000	4.430252000
H	-2.496796000	1.096103000	2.721835000
C	-0.001727000	2.156795000	3.460411000
H	1.076437000	2.223496000	3.701586000
H	-0.106045000	2.412178000	2.386078000
H	-0.517695000	2.957596000	4.036652000
C	1.988303000	-0.853372000	2.917546000
H	2.208669000	-1.622365000	2.139995000
C	2.401929000	-1.427678000	4.284724000
H	1.941630000	-2.415359000	4.491284000
H	3.506238000	-1.562145000	4.343557000
H	2.115735000	-0.746965000	5.116489000
C	2.800704000	0.402738000	2.581526000
H	2.409645000	0.925015000	1.686184000
H	2.778562000	1.134993000	3.415888000
H	3.867869000	0.157614000	2.400959000
Si	-5.007333000	-0.160428000	0.579726000
C	-3.916975000	-1.316767000	1.563501000
H	-4.223563000	-1.369990000	2.627389000
H	-3.922384000	-2.339628000	1.138091000
H	-2.880059000	-0.930906000	1.515529000
C	-5.390699000	1.425151000	1.524677000
H	-5.876911000	2.174207000	0.865830000
H	-6.085494000	1.198300000	2.361697000
H	-4.474567000	1.878033000	1.952749000
C	-6.598660000	-0.977479000	-0.018464000
H	-7.190912000	-0.282704000	-0.649813000
H	-6.374239000	-1.884570000	-0.617282000

H	-7.227060000	-1.279610000	0.846320000

5b: G= -3155.384836

Al	0.217985000	0.051078000	-0.351345000
C	-1.679544000	-0.057059000	0.232304000
P	-3.046758000	-0.052187000	-0.868767000
N	-2.098869000	-0.147652000	1.540732000
N	-3.391856000	-0.209726000	1.710717000
N	-4.066085000	-0.170988000	0.533963000
N	0.319931000	1.578933000	-1.526280000
N	0.467168000	-1.307501000	-1.694351000
O	1.512524000	0.035175000	0.795342000
Si	1.848921000	-0.211809000	2.423761000
C	-0.066627000	1.464548000	-2.810987000
C	-0.280254000	0.210339000	-3.440056000
H	-0.628794000	0.255165000	-4.480470000
C	0.064367000	-1.078756000	-2.962241000
C	-0.297421000	2.705021000	-3.645803000
H	-0.056046000	2.523771000	-4.710265000
H	-1.370183000	2.987582000	-3.587265000
H	0.292875000	3.564797000	-3.276088000
C	-0.002651000	-2.232553000	-3.937288000
H	-0.103157000	-1.878196000	-4.979432000
H	0.897564000	-2.873475000	-3.853912000
H	-0.872644000	-2.879843000	-3.702111000
C	0.662945000	2.851418000	-0.939212000
C	-0.350121000	3.723657000	-0.449384000
C	0.049161000	4.905004000	0.211821000
H	-0.722645000	5.582645000	0.609776000
C	1.400275000	5.226464000	0.373493000
H	1.691628000	6.150409000	0.897530000
C	2.386323000	4.367601000	-0.135500000
H	3.444113000	4.637630000	-0.008018000
C	2.047175000	3.168637000	-0.792164000
C	-1.842352000	3.445990000	-0.618471000
H	-1.948695000	2.499535000	-1.185951000
C	-2.548938000	3.236615000	0.735934000
H	-3.612091000	2.956827000	0.577834000
H	-2.073561000	2.428858000	1.322337000
H	-2.528662000	4.162213000	1.349726000
C	-2.539622000	4.561212000	-1.426413000
H	-3.590884000	4.279326000	-1.647016000
H	-2.564579000	5.516439000	-0.860084000
H	-2.025682000	4.760961000	-2.388530000
C	3.129794000	2.250660000	-1.363647000
H	2.832265000	1.211238000	-1.104927000
C	4.523166000	2.495432000	-0.761432000
H	5.218052000	1.691387000	-1.079737000
H	4.956589000	3.458411000	-1.107104000
H	4.501839000	2.503232000	0.345548000
C	3.210082000	2.354384000	-2.903813000
H	4.066055000	1.760450000	-3.285925000
H	2.300805000	1.970307000	-3.403309000
H	3.360805000	3.409113000	-3.217847000
C	0.950845000	-2.611068000	-1.305243000

C	0.034891000	-3.647574000	-0.968916000
C	0.552837000	-4.898388000	-0.572047000
H	-0.144479000	-5.706221000	-0.299690000
C	1.931077000	-5.122157000	-0.500718000
H	2.318378000	-6.103857000	-0.185130000
C	2.822148000	-4.085249000	-0.816778000
H	3.901973000	-4.275156000	-0.743608000
C	2.362570000	-2.814595000	-1.217968000
C	-1.477749000	-3.443060000	-0.966356000
H	-1.686249000	-2.448628000	-1.410181000
C	-2.017205000	-3.411452000	0.478310000
H	-3.093392000	-3.136807000	0.490669000
H	-1.907563000	-4.402843000	0.967407000
H	-1.475646000	-2.669193000	1.094113000
C	-2.226795000	-4.495700000	-1.807313000
H	-3.306402000	-4.243412000	-1.866272000
H	-1.831801000	-4.558953000	-2.842645000
H	-2.149093000	-5.509723000	-1.361261000
C	3.349831000	-1.687618000	-1.537572000
H	2.982527000	-0.799040000	-0.977271000
C	3.387388000	-1.327457000	-3.039723000
H	4.197244000	-0.593969000	-3.233869000
H	3.593439000	-2.227859000	-3.656929000
H	2.445723000	-0.867010000	-3.392577000
C	4.779974000	-1.973911000	-1.051011000
H	5.404479000	-1.064572000	-1.162728000
H	4.806384000	-2.268676000	0.016324000
H	5.262128000	-2.779318000	-1.645867000
C	0.623724000	-1.531383000	3.080440000
H	-0.330488000	-1.240546000	2.581901000
C	0.361027000	-1.492202000	4.597647000
H	-0.044995000	-0.511953000	4.925097000
H	-0.382060000	-2.267475000	4.892786000
H	1.281406000	-1.685207000	5.191539000
C	1.009033000	-2.944172000	2.600148000
H	1.213984000	-2.977002000	1.509316000
H	1.918057000	-3.320639000	3.118613000
H	0.195889000	-3.677283000	2.799663000
C	1.585458000	1.439851000	3.357758000
H	1.565083000	1.164215000	4.438551000
C	0.210872000	2.022044000	2.972505000
H	-0.613602000	1.282941000	3.052594000
H	-0.051306000	2.903523000	3.599750000
H	0.228403000	2.376130000	1.919359000
C	2.691639000	2.490777000	3.146800000
H	3.674190000	2.159662000	3.542318000
H	2.817921000	2.728211000	2.068346000
H	2.435402000	3.447987000	3.655398000
C	3.638266000	-0.879683000	2.524633000
H	3.560516000	-1.866452000	2.007430000
C	4.093680000	-1.132733000	3.974653000
H	3.419971000	-1.832189000	4.512182000
H	5.117779000	-1.568799000	4.009340000
H	4.124129000	-0.188581000	4.561891000
C	4.658730000	-0.033006000	1.747359000
H	4.301023000	0.185637000	0.723445000
H	4.848741000	0.939162000	2.246371000

H	5.640236000	-0.551082000	1.661149000
Si	-5.891443000	-0.233101000	0.677330000
C	-6.560916000	-0.186202000	-1.081164000
H	-6.262921000	0.747143000	-1.602178000
H	-6.198033000	-1.049199000	-1.676964000
H	-7.670962000	-0.227075000	-1.059051000
C	-6.288790000	-1.844865000	1.557976000
H	-7.380051000	-1.945230000	1.736293000
H	-5.951556000	-2.713776000	0.955293000
H	-5.765333000	-1.877043000	2.535390000
C	-6.384867000	1.281251000	1.675125000
H	-6.126672000	2.212606000	1.129385000
H	-7.475882000	1.288064000	1.880876000
H	-5.841378000	1.285022000	2.642080000

5b-iso: G= -3155.357584

Al	0.296824000	-0.129021000	-0.488727000
C	2.198670000	-0.483944000	-1.187252000
P	2.370918000	-0.948479000	-2.895611000
N	3.459596000	-0.588690000	-0.637735000
N	4.466878000	-1.031796000	-1.499290000
N	4.060007000	-1.256341000	-2.692078000
N	-0.796359000	-1.582922000	-1.207874000
N	-0.509691000	1.199557000	-1.661904000
O	0.078390000	0.213552000	1.194596000
Si	-0.488897000	0.649223000	2.722638000
C	-0.820310000	-1.645679000	-2.563790000
C	-0.670354000	-0.500991000	-3.379099000
H	-0.652792000	-0.675466000	-4.462750000
C	-0.664760000	0.851159000	-2.959721000
C	-0.899951000	-2.990679000	-3.247815000
H	-1.281383000	-2.913375000	-4.282911000
H	0.140426000	-3.386638000	-3.296088000
H	-1.504222000	-3.722765000	-2.681620000
C	-0.778913000	1.920606000	-4.021494000
H	-1.218520000	1.521168000	-4.953903000
H	-1.374239000	2.785903000	-3.674145000
H	0.238843000	2.303627000	-4.254167000
C	-1.336120000	-2.695508000	-0.461244000
C	-0.465025000	-3.699402000	0.040109000
C	-1.005354000	-4.733072000	0.831470000
H	-0.334158000	-5.502419000	1.244366000
C	-2.378916000	-4.798944000	1.088959000
H	-2.788735000	-5.609064000	1.712668000
C	-3.237573000	-3.839977000	0.531086000
H	-4.318084000	-3.924460000	0.715835000
C	-2.748792000	-2.776620000	-0.255669000
C	1.023393000	-3.691822000	-0.285082000
H	1.183071000	-2.960638000	-1.102232000
C	1.864491000	-3.214927000	0.910800000
H	2.935603000	-3.201675000	0.634144000
H	1.580059000	-2.189541000	1.227076000
H	1.734981000	-3.882050000	1.789415000
C	1.518737000	-5.053160000	-0.809539000

H	2.563367000	-4.963533000	-1.173669000
H	1.508123000	-5.834417000	-0.020094000
H	0.890636000	-5.413199000	-1.650629000
C	-3.727026000	-1.776501000	-0.884411000
H	-3.234317000	-0.782147000	-0.842187000
C	-5.076599000	-1.686568000	-0.144546000
H	-5.630033000	-0.786194000	-0.482954000
H	-5.718469000	-2.564559000	-0.371064000
H	-4.964801000	-1.624282000	0.953978000
C	-4.020284000	-2.076980000	-2.373719000
H	-4.855835000	-1.439330000	-2.730828000
H	-3.157681000	-1.868661000	-3.030573000
H	-4.318517000	-3.137788000	-2.510885000
C	-0.733293000	2.574067000	-1.282476000
C	0.339359000	3.502977000	-1.356418000
C	0.106498000	4.834027000	-0.954103000
H	0.932983000	5.560808000	-0.989227000
C	-1.154935000	5.240276000	-0.505563000
H	-1.320897000	6.281397000	-0.186872000
C	-2.211933000	4.318543000	-0.465226000
H	-3.198651000	4.658591000	-0.120601000
C	-2.034971000	2.975113000	-0.855391000
C	1.732558000	3.079817000	-1.811229000
H	1.649394000	2.084861000	-2.293922000
C	2.647379000	2.898388000	-0.587592000
H	3.638653000	2.506974000	-0.893096000
H	2.796252000	3.858529000	-0.049245000
H	2.204306000	2.180644000	0.129232000
C	2.351266000	4.039679000	-2.843466000
H	3.304705000	3.622891000	-3.229371000
H	1.672571000	4.202304000	-3.706398000
H	2.580390000	5.033597000	-2.404279000
C	-3.212930000	1.994948000	-0.838627000
H	-2.825986000	1.049516000	-0.397748000
C	-3.736949000	1.653851000	-2.253298000
H	-4.666110000	1.051624000	-2.176557000
H	-3.976398000	2.577984000	-2.820216000
H	-3.018180000	1.055025000	-2.841662000
C	-4.398061000	2.470940000	0.019682000
H	-5.148926000	1.659686000	0.104630000
H	-4.092572000	2.760466000	1.044848000
H	-4.912364000	3.339236000	-0.444244000
C	0.471096000	2.196966000	3.325900000
H	1.536601000	1.931177000	3.176146000
C	0.305014000	2.528387000	4.823076000
H	0.563227000	1.670111000	5.478743000
H	0.967581000	3.374552000	5.114383000
H	-0.730829000	2.831750000	5.077337000
C	0.173659000	3.411188000	2.424369000
H	0.294982000	3.171558000	1.348512000
H	-0.865720000	3.782832000	2.552214000
H	0.851287000	4.264544000	2.651757000
C	-0.236971000	-0.826378000	3.922930000
H	-0.786401000	-0.517622000	4.845415000
C	1.239905000	-1.055592000	4.306456000
H	1.740373000	-0.139442000	4.681906000
H	1.328644000	-1.833461000	5.097664000

H	1.824193000	-1.416515000	3.434346000
C	-0.850195000	-2.144597000	3.405940000
H	-1.950083000	-2.102054000	3.297162000
H	-0.445154000	-2.417041000	2.411280000
H	-0.619256000	-2.989142000	4.093457000
C	-2.325179000	1.132039000	2.540882000
H	-2.266555000	1.837761000	1.680433000
C	-2.949189000	1.889405000	3.727151000
H	-2.457003000	2.863948000	3.917544000
H	-4.026504000	2.099248000	3.536449000
H	-2.898877000	1.299094000	4.668329000
C	-3.214870000	-0.046188000	2.129690000
H	-2.737386000	-0.689844000	1.365407000
H	-3.449394000	-0.695829000	2.999676000
H	-4.185889000	0.306737000	1.724838000
Si	4.422728000	-0.212569000	0.909576000
C	3.322898000	0.702815000	2.106248000
H	3.626071000	0.484638000	3.149873000
H	3.382394000	1.796858000	1.947513000
H	2.270942000	0.389975000	1.972972000
C	5.041091000	-1.830103000	1.649206000
H	5.443371000	-2.499491000	0.862656000
H	5.865356000	-1.591562000	2.355695000
H	4.252882000	-2.365168000	2.215078000
C	5.884352000	0.864192000	0.407083000
H	6.587827000	0.308092000	-0.242282000
H	5.550215000	1.772333000	-0.134819000
H	6.418879000	1.193033000	1.324712000

TS-[2+4]: G= -2816.331683 (i = -227 cm-1)

Al	-0.026949000	0.288053000	-0.113350000
N	1.022239000	1.463270000	-1.235028000
N	-1.202493000	-0.372795000	-1.495278000
C	-1.101501000	1.262274000	1.208323000
P	-1.740106000	1.368944000	2.679960000
C	-2.030925000	3.185358000	-0.105809000
H	-1.433195000	3.666507000	0.676035000
H	-1.553477000	3.089529000	-1.091382000
C	-3.401862000	3.037580000	0.013197000
C	-4.035173000	3.096187000	1.310484000
C	-3.291417000	3.347605000	2.464321000
H	-3.790861000	3.361546000	3.446936000
H	-2.346927000	3.912361000	2.414945000
C	-4.217043000	2.683041000	-1.213691000
H	-3.562446000	2.545395000	-2.097250000
H	-4.808289000	1.756063000	-1.083542000
H	-4.946890000	3.487128000	-1.454419000
C	-5.488002000	2.690521000	1.433651000
H	-5.853008000	2.779215000	2.475362000
H	-6.138547000	3.320544000	0.789797000
H	-5.650911000	1.640268000	1.105551000
C	0.480348000	1.992183000	-2.355076000
C	-0.736655000	1.537241000	-2.921392000
H	-1.084671000	2.084181000	-3.807240000
C	-1.466922000	0.369243000	-2.589185000

C	1.202181000	3.117926000	-3.060664000
H	0.693254000	3.400640000	-4.000048000
H	1.257780000	4.006495000	-2.399498000
H	2.250426000	2.837656000	-3.286316000
C	-2.558364000	-0.078595000	-3.532814000
H	-2.340976000	-1.089497000	-3.933274000
H	-3.520405000	-0.162544000	-2.988455000
H	-2.680865000	0.622802000	-4.378021000
C	2.356967000	1.878325000	-0.854875000
C	2.538071000	3.031094000	-0.040872000
C	3.850391000	3.430773000	0.284628000
H	3.998551000	4.319124000	0.919082000
C	4.958213000	2.712990000	-0.174997000
H	5.978520000	3.039672000	0.081220000
C	4.764280000	1.557116000	-0.945910000
H	5.645364000	0.989005000	-1.275303000
C	3.474640000	1.103433000	-1.290560000
C	1.362963000	3.814254000	0.532048000
H	0.444034000	3.360710000	0.119688000
C	1.297037000	3.665413000	2.065781000
H	0.397714000	4.178764000	2.468389000
H	1.234403000	2.601824000	2.363549000
H	2.189775000	4.111911000	2.553345000
C	1.377443000	5.301956000	0.129004000
H	0.451801000	5.805469000	0.479199000
H	2.239529000	5.839328000	0.578147000
H	1.440004000	5.434956000	-0.970949000
C	3.292584000	-0.207256000	-2.061962000
H	2.488013000	-0.758078000	-1.525519000
C	4.552237000	-1.092148000	-2.048182000
H	4.313010000	-2.096672000	-2.451354000
H	5.358020000	-0.669202000	-2.685529000
H	4.958852000	-1.231515000	-1.027061000
C	2.842559000	-0.007057000	-3.527021000
H	2.813329000	-0.985173000	-4.050805000
H	1.831775000	0.433874000	-3.612105000
H	3.557356000	0.645048000	-4.073009000
C	-1.736096000	-1.712826000	-1.405054000
C	-2.955213000	-1.981488000	-0.723891000
C	-3.409027000	-3.316797000	-0.670123000
H	-4.349602000	-3.541295000	-0.142947000
C	-2.690158000	-4.361522000	-1.261314000
H	-3.063011000	-5.396050000	-1.200532000
C	-1.485658000	-4.081765000	-1.920612000
H	-0.914624000	-4.903832000	-2.380712000
C	-0.987555000	-2.767042000	-2.006694000
C	-3.807124000	-0.895180000	-0.069282000
H	-3.277273000	0.075561000	-0.182184000
C	-3.989236000	-1.152090000	1.439775000
H	-4.481072000	-0.283813000	1.925581000
H	-4.619267000	-2.047775000	1.626216000
H	-3.015362000	-1.310969000	1.940095000
C	-5.188041000	-0.798746000	-0.754889000
H	-5.811823000	-0.011027000	-0.283329000
H	-5.104771000	-0.566504000	-1.836435000
H	-5.745909000	-1.754145000	-0.664269000
C	0.324304000	-2.516854000	-2.747745000

H	0.563624000	-1.438790000	-2.660897000
C	1.484476000	-3.292429000	-2.098732000
H	1.611533000	-2.970973000	-1.047666000
H	1.310225000	-4.388896000	-2.115375000
H	2.433310000	-3.099985000	-2.639683000
C	0.195905000	-2.840149000	-4.250534000
H	1.141482000	-2.604327000	-4.782595000
H	-0.023062000	-3.916334000	-4.417060000
H	-0.615523000	-2.253737000	-4.728740000
O	0.959786000	-1.008913000	0.513920000
Si	1.222050000	-1.736575000	2.012631000
C	2.020877000	-0.435698000	3.175403000
H	1.160837000	0.233272000	3.424830000
C	3.085598000	0.420519000	2.462352000
H	2.718938000	0.851409000	1.511644000
H	3.418149000	1.268492000	3.101813000
H	3.989531000	-0.174412000	2.213861000
C	2.584266000	-1.006613000	4.492277000
H	1.865597000	-1.658198000	5.029685000
H	3.500890000	-1.607766000	4.306481000
H	2.876623000	-0.188955000	5.189256000
C	-0.497240000	-2.300788000	2.644603000
H	-1.125286000	-1.430944000	2.341348000
C	-0.675368000	-2.476668000	4.161339000
H	-0.437181000	-1.546028000	4.717040000
H	-1.727600000	-2.746324000	4.408108000
H	-0.030777000	-3.285816000	4.568779000
C	-0.992489000	-3.525576000	1.854644000
H	-0.860544000	-3.393326000	0.761304000
H	-0.449960000	-4.449953000	2.148913000
H	-2.075350000	-3.712353000	2.028868000
C	2.324331000	-3.268392000	1.692856000
H	1.745060000	-3.812235000	0.909078000
C	2.477798000	-4.219988000	2.895066000
H	1.499753000	-4.541833000	3.309083000
H	3.032707000	-5.141715000	2.607070000
H	3.045387000	-3.748625000	3.724272000
C	3.687239000	-2.889779000	1.089601000
H	3.574753000	-2.158282000	0.265452000
H	4.351334000	-2.429710000	1.852922000
H	4.223114000	-3.777484000	0.683822000

Conformers of species 6 (PCM-BP86-D3/def2-SVP level)

6-I: G= -2813.980641

Al	-0.006514000	0.348591000	-0.182545000
N	1.250679000	1.301156000	-1.317486000
N	-1.137144000	-0.300674000	-1.624840000
C	-1.105578000	1.688592000	0.791833000
P	-1.572935000	1.542260000	2.413080000
C	-1.662895000	2.855018000	-0.039488000
H	-1.134963000	3.780362000	0.304345000
H	-1.398338000	2.754698000	-1.112790000
C	-3.161074000	3.088883000	0.094028000
C	-3.700015000	3.140741000	1.343530000

C	-2.767919000	3.023609000	2.533195000
H	-3.319284000	2.975081000	3.494847000
H	-2.097903000	3.916624000	2.592158000
C	-3.897930000	3.256559000	-1.209397000
H	-3.707512000	2.393045000	-1.884014000
H	-4.993344000	3.364060000	-1.091753000
H	-3.528919000	4.156314000	-1.752182000
C	-5.166285000	3.303455000	1.661222000
H	-5.518855000	2.477100000	2.318461000
H	-5.353383000	4.246185000	2.222950000
H	-5.810167000	3.313217000	0.760283000
C	0.841244000	1.819193000	-2.496562000
C	-0.377294000	1.456767000	-3.122687000
H	-0.606253000	1.976273000	-4.062254000
C	-1.237364000	0.382522000	-2.783252000
C	1.727705000	2.812784000	-3.213751000
H	1.345554000	3.042470000	-4.224993000
H	1.789212000	3.756307000	-2.634140000
H	2.765100000	2.431836000	-3.295186000
C	-2.265442000	-0.046398000	-3.805332000
H	-2.348823000	0.685610000	-4.629603000
H	-1.987576000	-1.030320000	-4.236362000
H	-3.258441000	-0.187920000	-3.335642000
C	2.595256000	1.577540000	-0.859858000
C	2.869754000	2.763903000	-0.122953000
C	4.187616000	2.991715000	0.324937000
H	4.407822000	3.901420000	0.905688000
C	5.212086000	2.081782000	0.050143000
H	6.236912000	2.273960000	0.405325000
C	4.926963000	0.913213000	-0.671654000
H	5.742420000	0.204040000	-0.869730000
C	3.626617000	0.626502000	-1.134083000
C	1.789853000	3.782586000	0.230817000
H	0.860420000	3.469247000	-0.281121000
C	1.507076000	3.785963000	1.746922000
H	0.669950000	4.476342000	1.986264000
H	1.225128000	2.778323000	2.104967000
H	2.398136000	4.121695000	2.318744000
C	2.134792000	5.205223000	-0.256580000
H	1.278885000	5.890834000	-0.082746000
H	3.008602000	5.623149000	0.286461000
H	2.377400000	5.228024000	-1.339354000
C	3.337384000	-0.683461000	-1.875191000
H	2.439654000	-1.113854000	-1.378594000
C	4.479005000	-1.709918000	-1.762278000
H	4.149838000	-2.687077000	-2.169578000
H	5.367934000	-1.395120000	-2.349838000
H	4.801100000	-1.879020000	-0.716407000
C	3.006376000	-0.490021000	-3.373012000
H	2.887968000	-1.478885000	-3.863524000
H	2.066473000	0.066706000	-3.541641000
H	3.829790000	0.045664000	-3.891800000
C	-1.845556000	-1.553419000	-1.495541000
C	-3.125644000	-1.609995000	-0.875240000
C	-3.772151000	-2.861124000	-0.792583000
H	-4.763677000	-2.919416000	-0.316761000
C	-3.180239000	-4.026782000	-1.293182000

H	-3.702221000	-4.993241000	-1.211898000
C	-1.912062000	-3.957140000	-1.885602000
H	-1.439548000	-4.876595000	-2.266885000
C	-1.225392000	-2.732193000	-2.002302000
C	-3.829508000	-0.378402000	-0.311872000
H	-3.128374000	0.476519000	-0.386237000
C	-4.175196000	-0.549390000	1.178396000
H	-4.553683000	0.408852000	1.589114000
H	-4.958046000	-1.321859000	1.335344000
H	-3.283332000	-0.840750000	1.766019000
C	-5.100743000	-0.025082000	-1.110989000
H	-5.578659000	0.883473000	-0.690482000
H	-4.881998000	0.172489000	-2.180474000
H	-5.843227000	-0.850061000	-1.068444000
C	0.157796000	-2.711580000	-2.649874000
H	0.505634000	-1.659732000	-2.677880000
C	1.168047000	-3.500021000	-1.796069000
H	1.260712000	-3.037655000	-0.794905000
H	0.856255000	-4.557874000	-1.666700000
H	2.168887000	-3.502297000	-2.274107000
C	0.125202000	-3.225708000	-4.103053000
H	1.125191000	-3.121537000	-4.574287000
H	-0.155200000	-4.299292000	-4.151722000
H	-0.601725000	-2.661777000	-4.723532000
O	0.764784000	-0.971396000	0.647837000
Si	0.870286000	-1.697645000	2.165554000
C	1.692455000	-0.436356000	3.354690000
H	0.858790000	0.272677000	3.579199000
C	2.816926000	0.369004000	2.674643000
H	2.505131000	0.797183000	1.702475000
H	3.155236000	1.213721000	3.315416000
H	3.707837000	-0.261065000	2.468991000
C	2.181781000	-1.037358000	4.687359000
H	1.407245000	-1.642221000	5.202294000
H	3.063918000	-1.694947000	4.527999000
H	2.502697000	-0.238476000	5.393517000
C	-0.923243000	-2.114875000	2.687902000
H	-1.461413000	-1.194130000	2.360110000
C	-1.196179000	-2.273398000	4.192577000
H	-0.912035000	-1.363734000	4.761547000
H	-2.278787000	-2.454194000	4.382365000
H	-0.644141000	-3.132233000	4.631934000
C	-1.477023000	-3.290889000	1.862927000
H	-1.271446000	-3.169223000	0.779598000
H	-1.034970000	-4.260201000	2.180344000
H	-2.580238000	-3.382749000	1.973069000
C	1.878855000	-3.308362000	1.946715000
H	1.344449000	-3.814936000	1.109129000
C	1.852490000	-4.265975000	3.153768000
H	0.819759000	-4.545950000	3.447851000
H	2.396263000	-5.210539000	2.924900000
H	2.339383000	-3.821066000	4.046426000
C	3.316002000	-3.018544000	1.481970000
H	3.331836000	-2.279801000	0.656815000
H	3.929435000	-2.601101000	2.309630000
H	3.831916000	-3.938363000	1.124569000

6-II: G= -2813.979787

Al	0.074424000	0.301433000	-0.200847000
N	1.253263000	-0.527899000	-1.507865000
N	-1.024121000	1.254693000	-1.487547000
C	1.180174000	1.650610000	0.753115000
P	1.582896000	1.553888000	2.394799000
C	1.810993000	2.761408000	-0.102787000
H	1.578729000	2.629270000	-1.179956000
H	1.307328000	3.718028000	0.188137000
C	3.311582000	2.944815000	0.073840000
C	3.809471000	3.029772000	1.338446000
C	2.834963000	2.990599000	2.499217000
H	2.198690000	3.909788000	2.504162000
H	3.353045000	2.957511000	3.479847000
C	4.099461000	3.019850000	-1.208625000
H	3.790822000	3.907435000	-1.806296000
H	5.194042000	3.082202000	-1.056499000
H	3.892321000	2.133642000	-1.848432000
C	5.269150000	3.157086000	1.700403000
H	5.464036000	4.104319000	2.251632000
H	5.578808000	2.333376000	2.382211000
H	5.941585000	3.134735000	0.820730000
C	1.505629000	0.099915000	-2.678087000
C	0.726175000	1.177902000	-3.168942000
H	1.057823000	1.614717000	-4.119714000
C	-0.523426000	1.632963000	-2.680868000
C	2.632640000	-0.397216000	-3.556009000
H	2.722789000	0.205665000	-4.477847000
H	3.595292000	-0.369389000	-3.008410000
H	2.470658000	-1.458792000	-3.834009000
C	-1.341253000	2.549731000	-3.560578000
H	-0.782503000	2.854052000	-4.464389000
H	-2.276818000	2.043402000	-3.875228000
H	-1.655845000	3.453295000	-3.001367000
C	1.945080000	-1.772267000	-1.247261000
C	3.229982000	-1.775878000	-0.636195000
C	3.896905000	-3.010290000	-0.482688000
H	4.894430000	-3.026035000	-0.015642000
C	3.312778000	-4.209470000	-0.898000000
H	3.851324000	-5.162619000	-0.775986000
C	2.020922000	-4.196487000	-1.443883000
H	1.558491000	-5.150996000	-1.731223000
C	1.305289000	-2.995329000	-1.618094000
C	3.893936000	-0.521786000	-0.074045000
H	3.217413000	0.337988000	-0.252961000
C	4.070200000	-0.652926000	1.451833000
H	4.423525000	0.308427000	1.877632000
H	3.116214000	-0.915866000	1.948715000
H	4.816342000	-1.435111000	1.708245000
C	5.251104000	-0.209823000	-0.734401000
H	5.683277000	0.714282000	-0.299462000
H	5.980524000	-1.031511000	-0.571989000
H	5.161049000	-0.056112000	-1.829317000
C	-0.135155000	-3.022189000	-2.140025000
H	-0.703306000	-2.319714000	-1.490608000
C	-0.789543000	-4.409116000	-2.006722000

H	-1.875185000	-4.338189000	-2.217532000
H	-0.364236000	-5.135505000	-2.731786000
H	-0.673046000	-4.831735000	-0.988965000
C	-0.273786000	-2.541166000	-3.602085000
H	-1.323678000	-2.659715000	-3.943325000
H	-0.010825000	-1.474444000	-3.731774000
H	0.369227000	-3.145512000	-4.277134000
C	-2.391905000	1.580316000	-1.157638000
C	-2.697598000	2.770275000	-0.438930000
C	-4.041297000	3.020299000	-0.094352000
H	-4.289026000	3.933711000	0.469215000
C	-5.061533000	2.126781000	-0.444380000
H	-6.103887000	2.334001000	-0.155197000
C	-4.746969000	0.966686000	-1.165675000
H	-5.550950000	0.268437000	-1.448411000
C	-3.420779000	0.675165000	-1.542691000
C	-1.622521000	3.776491000	-0.042126000
H	-0.664561000	3.404846000	-0.452939000
C	-1.468049000	3.867147000	1.488411000
H	-0.618509000	4.530926000	1.756524000
H	-2.383508000	4.279906000	1.963201000
H	-1.269083000	2.872058000	1.929363000
C	-1.878305000	5.170405000	-0.651204000
H	-1.033825000	5.854319000	-0.422809000
H	-1.991628000	5.125138000	-1.754323000
H	-2.801467000	5.631695000	-0.240907000
C	-3.130198000	-0.582213000	-2.357772000
H	-2.039720000	-0.613272000	-2.551703000
C	-3.484281000	-1.850167000	-1.563202000
H	-2.884563000	-1.891034000	-0.635424000
H	-4.559728000	-1.876695000	-1.287505000
H	-3.270240000	-2.759968000	-2.160181000
C	-3.843473000	-0.553994000	-3.725016000
H	-3.552537000	-1.436581000	-4.333005000
H	-4.947915000	-0.577419000	-3.609508000
H	-3.587931000	0.356695000	-4.305611000
O	-0.855099000	-0.891827000	0.659059000
Si	-1.312674000	-1.335753000	2.219703000
C	0.219908000	-2.099680000	3.082930000
H	0.834454000	-1.203385000	3.341604000
C	1.052452000	-2.975196000	2.126245000
H	1.314462000	-2.444932000	1.191219000
H	2.004205000	-3.302058000	2.601688000
H	0.504001000	-3.893481000	1.826938000
C	-0.099973000	-2.861366000	4.383891000
H	-0.715389000	-2.269828000	5.092253000
H	-0.654260000	-3.801010000	4.169600000
H	0.832596000	-3.154382000	4.916914000
C	-1.907480000	0.270514000	3.080604000
H	-1.136888000	1.002403000	2.739918000
C	-1.885750000	0.293286000	4.618604000
H	-0.869077000	0.098942000	5.020629000
H	-2.204972000	1.287835000	5.005560000
H	-2.572690000	-0.459804000	5.062312000
C	-3.262960000	0.722027000	2.505045000
H	-3.276239000	0.685497000	1.396256000
H	-4.096440000	0.082639000	2.867903000

H	-3.503626000	1.768291000	2.796889000
C	-2.779556000	-2.554054000	2.049413000
H	-3.472781000	-1.992536000	1.379583000
C	-3.540059000	-2.843740000	3.358838000
H	-3.891288000	-1.918824000	3.860420000
H	-4.437770000	-3.473993000	3.165425000
H	-2.911710000	-3.395370000	4.088970000
C	-2.386401000	-3.859129000	1.335026000
H	-1.804833000	-3.666262000	0.412441000
H	-1.760456000	-4.502640000	1.990300000
H	-3.280573000	-4.457491000	1.048049000

6-III: G= -2813.978015

Al	0.100929000	0.171852000	-0.455552000
N	1.036299000	-1.089741000	-1.601189000
N	-1.289127000	0.616186000	-1.735817000
C	1.229941000	1.786636000	-0.094078000
P	1.727751000	2.675853000	-1.449571000
C	1.585557000	2.255386000	1.313442000
H	0.789722000	2.974787000	1.631826000
H	1.491250000	1.416165000	2.034613000
C	2.936519000	2.926768000	1.522954000
C	3.434260000	3.785896000	0.592654000
C	2.598703000	4.145876000	-0.620496000
H	3.197712000	4.685114000	-1.384524000
H	1.770864000	4.837325000	-0.331147000
C	3.614759000	2.540292000	2.813784000
H	2.938759000	2.731699000	3.677426000
H	3.838115000	1.450018000	2.834755000
H	4.560007000	3.084125000	3.003173000
C	4.795824000	4.436698000	0.648032000
H	5.392898000	4.156927000	-0.249465000
H	4.711600000	5.546545000	0.630526000
H	5.385672000	4.155198000	1.541519000
C	1.027392000	-0.893956000	-2.940708000
C	0.137395000	-0.007677000	-3.592834000
H	0.281325000	0.102491000	-4.675638000
C	-1.011180000	0.620932000	-3.055867000
C	1.987364000	-1.675768000	-3.810692000
H	1.764275000	-1.534361000	-4.883879000
H	3.031570000	-1.354103000	-3.621900000
H	1.947448000	-2.757249000	-3.572084000
C	-1.935365000	1.333364000	-4.018403000
H	-1.842401000	0.933071000	-5.045110000
H	-2.992960000	1.280017000	-3.696402000
H	-1.657557000	2.409476000	-4.044799000
C	1.883066000	-2.134279000	-1.067025000
C	3.268759000	-1.887076000	-0.853756000
C	4.070087000	-2.929106000	-0.342084000
H	5.141412000	-2.744742000	-0.164741000
C	3.527733000	-4.183433000	-0.049488000
H	4.167387000	-4.987661000	0.347475000
C	2.159345000	-4.410860000	-0.254566000
H	1.747336000	-5.400032000	-0.012367000
C	1.309367000	-3.404897000	-0.757083000

C	3.906451000	-0.520345000	-1.081352000
H	3.167230000	0.128295000	-1.592439000
C	4.207522000	0.132883000	0.279566000
H	4.549178000	1.180307000	0.155393000
H	3.295795000	0.159294000	0.907465000
H	4.983547000	-0.435877000	0.835153000
C	5.172286000	-0.573033000	-1.958129000
H	5.535155000	0.455025000	-2.167054000
H	6.000453000	-1.119741000	-1.459398000
H	4.983695000	-1.072552000	-2.931334000
C	-0.183256000	-3.683144000	-0.955222000
H	-0.719058000	-2.818264000	-0.501478000
C	-0.664098000	-4.959321000	-0.240855000
H	-1.771370000	-5.010900000	-0.263935000
H	-0.282874000	-5.874600000	-0.742139000
H	-0.351993000	-4.994564000	0.821570000
C	-0.585112000	-3.766657000	-2.445111000
H	-1.641615000	-4.093290000	-2.535348000
H	-0.501500000	-2.796547000	-2.970145000
H	0.042348000	-4.511916000	-2.978638000
C	-2.602407000	0.987296000	-1.267548000
C	-2.940509000	2.344172000	-1.016133000
C	-4.235109000	2.633809000	-0.535603000
H	-4.509109000	3.680802000	-0.330128000
C	-5.168351000	1.617262000	-0.299125000
H	-6.169775000	1.863824000	0.087609000
C	-4.820065000	0.281515000	-0.551608000
H	-5.559281000	-0.513467000	-0.370611000
C	-3.545098000	-0.058838000	-1.043823000
C	-1.943866000	3.479611000	-1.218417000
H	-1.039068000	3.055160000	-1.699864000
C	-1.497282000	4.040733000	0.145047000
H	-0.679523000	4.782124000	0.023711000
H	-2.339577000	4.536052000	0.673393000
H	-1.126218000	3.226391000	0.797239000
C	-2.487499000	4.593578000	-2.132693000
H	-1.695883000	5.344115000	-2.339393000
H	-2.840422000	4.188714000	-3.103452000
H	-3.339611000	5.131916000	-1.666071000
C	-3.214250000	-1.499573000	-1.422152000
H	-2.112983000	-1.607063000	-1.356503000
C	-3.839050000	-2.544526000	-0.486512000
H	-3.597181000	-2.329455000	0.571839000
H	-4.943923000	-2.592588000	-0.588537000
H	-3.447931000	-3.553904000	-0.728260000
C	-3.621302000	-1.765494000	-2.888374000
H	-3.422097000	-2.820225000	-3.169538000
H	-4.705208000	-1.570173000	-3.033916000
H	-3.060816000	-1.121113000	-3.593478000
O	-0.619646000	-0.603485000	0.926188000
Si	-0.857047000	-0.522201000	2.585006000
C	0.814838000	-0.935260000	3.444992000
H	1.362369000	0.038679000	3.464576000
C	1.662579000	-1.926162000	2.624797000
H	1.849924000	-1.570488000	1.593009000
H	2.652640000	-2.103921000	3.100753000
H	1.161946000	-2.912615000	2.526673000

C	0.659427000	-1.410117000	4.902774000
H	0.051956000	-0.715181000	5.518896000
H	0.168286000	-2.405733000	4.946053000
H	1.649950000	-1.517415000	5.399396000
C	-1.431535000	1.257879000	3.009218000
H	-0.794182000	1.875867000	2.338421000
C	-1.161013000	1.725461000	4.450925000
H	-0.085155000	1.657736000	4.719677000
H	-1.469957000	2.785880000	4.592893000
H	-1.725898000	1.123532000	5.195323000
C	-2.890816000	1.495031000	2.580545000
H	-3.076329000	1.152660000	1.542932000
H	-3.607202000	0.960926000	3.240559000
H	-3.155224000	2.575249000	2.617599000
C	-2.254491000	-1.763807000	2.983830000
H	-3.049982000	-1.420284000	2.278367000
C	-2.842609000	-1.708654000	4.406279000
H	-3.171444000	-0.688491000	4.693349000
H	-3.730390000	-2.375243000	4.493730000
H	-2.111564000	-2.046324000	5.169939000
C	-1.865749000	-3.194852000	2.580734000
H	-1.437451000	-3.213041000	1.561335000
H	-1.105075000	-3.621468000	3.269876000
H	-2.739720000	-3.884664000	2.591852000

6-IV: G= -2813.976804

Al	0.085088000	0.310960000	-0.393453000
N	1.750968000	0.773606000	-1.291579000
N	-0.753020000	-0.513537000	-1.933495000
C	-0.906731000	1.973173000	0.115849000
P	-1.311321000	2.993481000	-1.180393000
C	-1.303855000	2.378569000	1.536677000
H	-1.162003000	1.541563000	2.248597000
H	-0.572835000	3.153559000	1.871834000
C	-2.705071000	2.960841000	1.696953000
C	-3.122627000	3.919647000	0.825107000
C	-2.155284000	4.410687000	-0.232064000
H	-1.311445000	4.971610000	0.238089000
H	-2.637685000	5.104175000	-0.951472000
C	-3.527145000	2.375395000	2.816688000
H	-2.953036000	2.371445000	3.769175000
H	-4.474913000	2.918094000	2.997275000
H	-3.782217000	1.312115000	2.610717000
C	-4.509076000	4.513266000	0.771720000
H	-4.479560000	5.620733000	0.878646000
H	-4.977454000	4.308933000	-0.218032000
H	-5.188580000	4.113292000	1.548754000
C	1.741013000	1.033101000	-2.620076000
C	0.648314000	0.732052000	-3.467928000
H	0.748676000	1.059447000	-4.510905000
C	-0.475410000	-0.076769000	-3.179693000
C	2.960786000	1.654063000	-3.264653000
H	2.892163000	1.624590000	-4.367290000
H	3.065143000	2.711042000	-2.947108000

H	3.888255000	1.139954000	-2.943771000
C	-1.392903000	-0.449564000	-4.322799000
H	-0.912991000	-0.280151000	-5.304184000
H	-1.728877000	-1.502516000	-4.254437000
H	-2.308034000	0.178641000	-4.269430000
C	2.980137000	1.000703000	-0.561898000
C	3.320002000	2.311713000	-0.120232000
C	4.534326000	2.497835000	0.573241000
H	4.802111000	3.506996000	0.924545000
C	5.396809000	1.428200000	0.826988000
H	6.342708000	1.591594000	1.367379000
C	5.046167000	0.140561000	0.396690000
H	5.731346000	-0.691434000	0.609624000
C	3.842830000	-0.106696000	-0.294185000
C	2.403846000	3.518467000	-0.309017000
H	1.529881000	3.200234000	-0.911503000
C	1.867336000	3.982076000	1.060643000
H	1.078608000	4.754330000	0.937478000
H	1.436914000	3.132240000	1.625189000
H	2.677450000	4.418886000	1.682827000
C	3.086831000	4.692464000	-1.039157000
H	2.357161000	5.509602000	-1.218950000
H	3.921744000	5.117018000	-0.442706000
H	3.504142000	4.388949000	-2.021349000
C	3.485018000	-1.528059000	-0.737060000
H	2.425714000	-1.687687000	-0.430370000
C	4.340068000	-2.610143000	-0.053008000
H	3.927124000	-3.614740000	-0.275794000
H	5.386821000	-2.597472000	-0.425254000
H	4.365412000	-2.496861000	1.048791000
C	3.577662000	-1.717736000	-2.267912000
H	3.430420000	-2.786220000	-2.527811000
H	2.812273000	-1.141851000	-2.820878000
H	4.580812000	-1.418221000	-2.639232000
C	-1.695908000	-1.589858000	-1.748839000
C	-3.087834000	-1.334236000	-1.622534000
C	-3.954573000	-2.432082000	-1.438439000
H	-5.035797000	-2.250445000	-1.332881000
C	-3.464882000	-3.742257000	-1.369932000
H	-4.157632000	-4.584697000	-1.215787000
C	-2.086714000	-3.977947000	-1.490416000
H	-1.708077000	-5.010228000	-1.439265000
C	-1.180492000	-2.917572000	-1.686990000
C	-3.654590000	0.080552000	-1.638287000
H	-2.837036000	0.770635000	-1.933456000
C	-4.085634000	0.492713000	-0.220083000
H	-4.398785000	1.556120000	-0.189563000
H	-4.920689000	-0.140156000	0.148857000
H	-3.239781000	0.385635000	0.486531000
C	-4.804435000	0.255995000	-2.648213000
H	-5.103277000	1.323504000	-2.707977000
H	-4.511766000	-0.077764000	-3.665519000
H	-5.706124000	-0.322224000	-2.354476000
C	0.303549000	-3.193995000	-1.913550000
H	0.852209000	-2.272260000	-1.635306000
C	0.852578000	-4.338567000	-1.048548000
H	0.602518000	-4.187933000	0.019096000

H	0.458296000	-5.328493000	-1.361924000
H	1.957222000	-4.386979000	-1.136800000
C	0.573294000	-3.461016000	-3.410769000
H	1.640638000	-3.711898000	-3.581462000
H	-0.040997000	-4.312641000	-3.773585000
H	0.335428000	-2.577544000	-4.035015000
O	0.312621000	-0.894788000	0.843354000
Si	-0.066987000	-1.310922000	2.423261000
C	0.878696000	-0.109949000	3.592683000
H	0.221180000	0.791250000	3.649056000
C	2.227784000	0.340054000	2.998186000
H	2.116826000	0.808808000	2.000604000
H	2.734852000	1.078873000	3.658150000
H	2.925095000	-0.514474000	2.864172000
C	1.056031000	-0.644110000	5.027851000
H	0.101734000	-0.983366000	5.481782000
H	1.757500000	-1.505498000	5.049877000
H	1.484210000	0.135853000	5.696966000
C	-1.964366000	-1.123903000	2.640599000
H	-2.171987000	-0.181693000	2.083627000
C	-2.459739000	-0.921264000	4.082632000
H	-1.988167000	-0.041125000	4.568009000
H	-3.561029000	-0.757718000	4.109793000
H	-2.249838000	-1.805355000	4.722339000
C	-2.725066000	-2.246158000	1.912290000
H	-2.354135000	-2.389158000	0.877702000
H	-2.627838000	-3.219514000	2.439138000
H	-3.812034000	-2.020252000	1.839968000
C	0.408858000	-3.149710000	2.641174000
H	-0.151542000	-3.618462000	1.796032000
C	-0.068661000	-3.822402000	3.941589000
H	-1.158788000	-3.700444000	4.108012000
H	0.140150000	-4.915994000	3.922320000
H	0.448273000	-3.411815000	4.833395000
C	1.907007000	-3.380132000	2.393204000
H	2.228039000	-2.900890000	1.450134000
H	2.525446000	-2.952642000	3.212022000
H	2.157688000	-4.462855000	2.323915000

6. References

[1] D. Heift, Z. Benko, H. Grützmacher, "Coulomb repulsion versus cycloaddition: formation of anionic four-membered rings from sodium phosphaethynolate, Na(OCP)" *Dalton Transactions 43* (2014): 831–840.

[2] J. Feldman, S. J. McLain, A. Parthasarathy, W. J. Marshall, J. C. Calabrese, S. D. Arthur, "Electrophilic Metal Precursors and a β-Diimine Ligand for Nickel(II)- and Palladium(II)-Catalyzed Ethylene Polymerization". *Organometallics* **16**, (1997): 1514–1516.

[3] O. Kysliak, H. Görls, R. Kretschmer, “Salt metathesis as an alternative approach to access aluminium(I) and gallium(I) β-diketiminates”. *Dalton Transactions* **49**, (2020): 6377–6383.

[4] G. Rabe, H. W. Roesky, D. Stalke, F. Pauer, G. M. Sheldrick, “The preparation and crystal structures of sodium and potassium pentamethylcyclopentadienyl pyridine solvates” *Journal of Organometallic Chemistry* **403**, (1991): 11–19.

[5] C. Ganesamoorthy, S. Loerke, C. Gemel, P. Jerabek, M. Winter, G. Frenking, R. A. Fischer, “Reductive elimination: a pathway to low-valent aluminium species”. *Chemical Communications 48*, (2013) 2858–2860.

[6] Bruker, *SAINT*, *V8.41*, Bruker AXS Inc., Madison, Wisconsin, USA.

[7] L. Krause, R. Herbst-Irmer, G. M. Sheldrick, D. Stalke, “Comparison of silver and molybdenum microfocus X-ray sources for single-crystal structure determination”. *Journal of Applied Crystallography* **48**, (2015): 3–10.

[8] G. M. Sheldrick, “A short history of SHELX” *Acta Crystallographica A* **A64**, (2008): 112–122.

[9] G. M. Sheldrick, “Crystal structure refinement with SHELXL” *Acta Crystallographica C* **C71**, (2015): 3–8.

[10] Gaussian 16, Revision C.01, M. J. Frisch, G. W. Trucks, H. B. Schlegel, G. E. Scuseria, M. A. Robb, J. R. Cheeseman, G. Scalmani, V. Barone, G. A. Petersson, H. Nakatsuji, X. Li, M. Caricato, A. V. Marenich, J. Bloino, B. G. Janesko, R. Gomperts, B. Mennucci, H. P. Hratchian, J. V. Ortiz, A. F. Izmaylov, J. L. Sonnenberg, D. Williams-Young, F. Ding, F. Lipparini, F. Egidi, J. Goings, B. Peng, A. Petrone, T. Henderson, D. Ranasinghe, V. G. Zakrzewski, J. Gao, N. Rega, G. Zheng, W. Liang, M. Hada, M. Ehara, K. Toyota, R. Fukuda, J. Hasegawa, M. Ishida, T. Nakajima, Y. Honda, O. Kitao, H. Nakai, T. Vreven, K.

Throssell, J. A. Montgomery, Jr., J. E. Peralta, F. Ogliaro, M. J. Bearpark, J. J. Heyd, E. N. Brothers, K. N. Kudin, V. N. Staroverov, T. A. Keith, R. Kobayashi, J. Normand, K. Raghavachari, A. P. Rendell, J. C. Burant, S. S. Iyengar, J. Tomasi, M. Cossi, J. M. Millam, M. Klene, C. Adamo, R. Cammi, J. W. Ochterski, R. L. Martin, K. Morokuma, O. Farkas, J. B. Foresman, and D. J. Fox, Gaussian, Inc., Wallingford CT, 2016.

[11] A. D. Becke, "Density-functional exchange-energy approximation with correct asymptotic behavior". *Physical Review A* **38**, (1988): 3098–3100.

[12] J. P. Perdew, "Density-functional approximation for the correlation energy of the inhomogeneous electron gas". *Physical Review B* **33**, (1986): 8822–8824.

[13] F. Weigend, R. Ahlrichs, "Balanced basis sets of split valence, triple zeta valence and quadruple zeta valence quality for H to Rn: Design and assessment of accuracy". *Physical Chemistry Chemical Physics* **7**, (2005): 3297–3305.

[14] S. Grimme, J. Antony, S. Ehrlich, H. Krieg, "A consistent and accurate ab initio parametrization of density functional dispersion correction (DFT-D) for the 94 elements H-Pu". *Journal of Chemical Physics* **132**, (2010): 154104–19.

[15] S. Miertuš, E. Scrocco, J. Tomasi, "Electrostatic interaction of a solute with a continuum. A direct utilizaion of AB initio molecular potentials for the prevision of solvent effects". *Chemical Physics* **55**, (1981): 117–129.

[16] J. L. Pascual-Ahuir, E. Silla, I. Tuñón, "GEPOL: An improved description of molecular surfaces. III. A new algorithm for the computation of a solvent-excluding surface". *Journal of Computational Chemistry* **15**, (1994): 1127–1138.

[17] V. Barone, M. Cossi, M. “Quantum Calculation of Molecular Energies and Energy Gradients in Solution by a Conductor Solvent Model” *Journal of Physical Chemistry A* **102**, (1998): 1995–2001.

[18] J. W. McIver, A. K. Komornicki, “Structure of transition states in organic reactions. General theory and an application to the cyclobutene-butadiene isomerization using a semiempirical molecular orbital method” *Journal of the American Chemical Society* **94**, (1972): 2625–2633.

[19] C. González, H. B. Schlegel, “Reaction path following in mass-weighted internal coordinates”. *Journal of Physical Chemistry* **94**, (1990): 5523–5527.

[20] S. Grimme, “Supramolecular Binding Thermodynamics by Dispersion-Corrected Density Functional Theory”. *Chemistry – A European Journal* **18**, (2012): 9955–9964.

[21] G. Luchini, J. V. Alegre-Requena, I. Funes-Ardoiz, R. S. Paton, “GoodVibes: automated thermochemistry for heterogeneous computational chemistry data”. *F1000 Research* 9, (2020): 291.

[22] F. M. Bickelhaupt, E. J. Baerends, E. J. in *Reviews in Computational Chemistry* (Eds. K. B. Lipkowitz, D. B. Boyd), Wiley-VCH: New York, 2000, Vol. 15, pp. 1–86.

[23] M. von Hopffgarten, G. Frenking, “Energy decomposition analysis”. *WIREs Computational Molecular Science* **2**, (2012): 43–62.

[24] ADF2020, SCM, Theoretical Chemistry, Vrije Universiteit, Amsterdam, The Netherlands, http://www.scm.com.

[25] J. G. Snijders, P. Vernooijs, E. J. Baerends, “Roothaan-Hartree-Fock-Slater atomic wave functions: Single-zeta, double-zeta, and extended Slater-type basis sets for ${}_{87}$Fr–${}_{103}$Lr”. *Atomic Data and Nuclear Data Tables* **26**, (1981): 483–509.

[26] J. Krijn, E. J. Baerends in *Fit Functions in the HFS-Method, Internal Report (in Dutch)*, Vrije Universiteit Amsterdam, The Netherlands, 1984.

[27] E. van Lenthe, E. J. Baerends, J. G. Snijders, "Relativistic regular two-component Hamiltonians". *Journal of Chemical Physics* **99**, (1993): 4597–4610.

[28] E. van Lenthe, E. J. Baerends, J. G. Snijders, "Relativistic total energy using regular approximations". *Journal of Chemical Physics* **101**, (1994): 9783–9792.

[29] E. van Lenthe, A. Ehlers, E. J. Baerends, "Geometry optimizations in the zero order regular approximation for relativistic effects". *Journal of Chemical Physics* **110** (1999): 8943–8953.
